# Genome Wide Association Study Identifies *L3MBTL4* as a Novel Susceptibility Gene for Hypertension

**DOI:** 10.1038/srep30811

**Published:** 2016-08-02

**Authors:** Xin Liu, Cheng Hu, Minghui Bao, Jing Li, Xiaoyan Liu, Xuerui Tan, Yong Zhou, Yequn Chen, Shouling Wu, Shuohua Chen, Rong Zhang, Feng Jiang, Weiping Jia, Xingyu Wang, Xinchun Yang, Jun Cai

**Affiliations:** 1 National Research Institute for Family Planning, Beijing, China; 2 Beijing Hypertension League Institute, Beijing, China; 3 Shanghai Diabetes Institute, Shanghai Jiao Tong University Affiliated Sixth People’s Hospital, Shanghai, China; 4 Institute for Metabolic Diseases, Shanghai Jiao Tong University Affiliated Sixth People’s Hospital South Campus, Shanghai, China; 5 Department of Cardiology, Beijing Chaoyang Hospital, Capital Medical University, Beijing, China; 6 Beijing Key Laboratory of Hypertension, Beijing, China; 7 Medical Research Center, Beijing Chaoyang Hospital, Capital Medical University, Beijing, China; 8 Department of Cardiology, The First Affiliated Hospital of Shantou University Medical College, Shantou, Guangdong, China; 9 Department of Cardiology, Beijing Friendship Hospital, Capital Medical University, Beijing, China; 10 Department of Cardiology, Kailuan General Hospital, Hebei Union University, Tangshan, Hebei, China; 11 Department of Hypertension, Fuwai Hospital, Beijing, China; 12 State Key Laboratory of Cardiovascular Disease, Beijing, China; 13 National Center for Cardiovascular Diseases, Chinese Academy of Medical Sciences and Peking Union Medical College, Beijing, China

## Abstract

Hypertension is a major global health burden and a leading risk factor for cardiovascular diseases. Although its heritability has been documented previously, contributing loci identified to date account for only a small fraction of blood pressure (BP) variation, which strongly suggests the existence of undiscovered variants. To identify novel variants, we conducted a three staged genetic study in 21,990 hypertensive cases and normotensive controls. Four single nucleotide polymorphisms (SNPs) at three new genes (*L3MBTL4* rs403814, *P*_meta_ = 6.128 × 10^−9^; *LOC729251*, and *TCEANC*) and seven SNPs at five previously reported genes were identified as being significantly associated with hypertension. Through functional analysis, we found that *L3MBTL4* is predominantly expressed in vascular smooth muscle cells and up-regulated in spontaneously hypertensive rats. Rats with ubiquitous over-expression of *L3MBTL4* exhibited significantly elevated BP, increased thickness of the vascular media layer and cardiac hypertrophy. Mechanistically, *L3MBTL4* over-expression could lead to down-regulation of latent transforming growth factor-β binding protein 1 (*LTBP1*), and phosphorylation activation of the mitogen-activated protein kinases (MAPK) signaling pathway, which is known to trigger the pathological progression of vascular remodeling and BP elevation. These findings pinpointed *L3MBTL4* as a critical contributor to the development and progression of hypertension and uncovers a novel target for therapeutic intervention.

Due to its high prevalence, hypertension is a major global health burden and represents an increased risk for cardiovascular diseases and premature death^1^,^2^,^3^,^4^,^5^. Although lifestyle is known to influence blood pressure (BP) in previous studies^6^,^7^, a substantial contribution of genetic factors has been documented by a number of genome-wide association studies (GWASs)^8^,^9^. However, the contributing loci identified to date account for only a small fraction of BP variation in the population and point to the existence of additional susceptibility loci.

Therefore, we performed a GWAS and two-staged follow-up study to identify novel genetic variants contributing to essential hypertension in the Chinese population. We first report a genome-wide significant locus in *L3MBTL4* gene (rs403814) and comprehensively illustrate its pathogenic effects and probable mechanisms as a potential inducer of hypertension. We estimated the expression level of *L3MBTL4* in spontaneously hypertensive rats (SHRs) and Wistar-Kyoto rats (WKYs) and found that *L3MBTL4* is predominantly expressed in vascular smooth muscle cells (SMCs) and upregulated in SHRs. Furthermore, rats over-expressing *L3MBTL4* exhibited significantly elevated BP and cardiac hypertrophy. We observed phosphorylation activation of the mitogen-activated protein kinases (MAPK) signaling pathway in the *L3MBTL4* over-expression model, which may be a trigger of vascular remodeling and BP elevation. A direct binding of L3MBTL4 with *LTBP1* was also detected, leading to decrease of the target gene. Moreover, copy number variation (CNV) burden analysis suggested a pathogenic role of 16q24.2 for hypertension.

## Results

### GWAS of Hypertensive Cases and Normotensive Controls

In stage 1, a genome-wide association analysis was performed in 528 Chinese individuals (276 cases and 252 controls) to identify genomic loci associated with BP using an extreme case-control strategy (Supplementary Methods, Supplementary Fig. S1, and Supplementary Table S1)^10^. In this stage, hypertensive cases were defined as having SBP ≥150 mmHg and/or DBP ≥ 90 mmHg in untreated subjects with an age of onset of ≤50 years. Normotensive controls were defined as having SBP ≤ 125 mmHg and DBP ≤ 80 mmHg without antihypertensive treatment. All controls were ≥65 years of age. Following a clean-up of the data, 518 samples (271 cases and 247 controls) and 727,172 SNPs remained for subsequent analyses. From this filtered data set, we found the genomic inflation factor λ value to be 1.02, indicating the absence of systemic confounding factors across the GWAS samples (Supplementary Fig. S2). We set a threshold of *P* < 5 × 10^−4^ in this stage. The association of genomic loci with hypertension and/or SBP and DBP identified a total of 358 SNPs within 152 chromosome regions beyond the significance threshold (Supplementary Fig. S3).

### Two Follow-up Studies and Combined Analyses

In stage 2, the 358 SNPs and 26 additional SNPs previously reported to be associated with SBP/DBP or hypertension^8^,^9^,^11^ were selected as candidate loci and genotyped in 4,608 Chinese individuals (2,044 cases and 2,564 controls) (Supplementary Methods). In this stage, hypertension and normotension were defined as described for stage 1, while the normotensive controls were ≥55 years of age. After data clean-up, 371 SNPs and 4,502 samples (1,994 cases and 2,508 controls) remained. We used a false discovery rate (FDR) threshold of 0.2^12,13^ and therefore expected a maximum of only 20% of declared discoveries to be false. After adjustment for multiple testing with FDR, 18 SNPs reached the level of FDR < 0.2 and were selected for stage 3 (Supplementary Table S2). These loci identified by these 18 SNPs consisted of several previously reported genes including *FGF5*, *ATP2B1*, *MTHFR*, *CASZ1*, *CYP11B2*, *CYP17A1*, *ADRB1*, and *HECTD4*^8^,^9^,^11^, as well as seven new genes (*L3MBTL4*, *KIRREL*, *C9orf98, GALNT18*, *LOC729251*, *NLRP1* and *TCEANC*) that may be associated with hypertension. We also generated haplotype analysis in stage 2, and no haplotype was associated with hypertension (Supplementary Table S3).

In stage 3, 17,318 samples (7,796 cases and 9,522 controls) selected from three cohorts (Shantou, Shanghai, and Jidong) were genotyped for the 18 SNPs identified from stage 2 (Supplementary Methods). In this stage, hypertension and normotension were defined as described for stage 1 but without age limitation. Following data clean-up, 16,970 samples (7,639 cases and 9,331 controls) were retained. We next performed a combined analysis of data from stages 1 to 3 and thereby identified *L3MBTL4* as a novel susceptibility gene significantly associated with hypertension (meta-analyses odds ratio (OR) = 1.15, 95% confidence interval (CI) = 1.07–1.23, *P*_*meta*_ = 6.128 × 10^−9^). In addition, we identified three SNPs in two novel genes (*LOC729251* and *TCEANC*) and seven SNPs in five previously reported genes (*FGF5, ATP2B1, CYP17A1, MTHFR* and *CASZ1*) nominally associated with hypertension (*P*_*meta*_ < 0.05) (Table 1).

Basing on GWAS data of stage 1 control group, 5 SNPs on *L3MBTL4* gene were genotyped in Shantou, Shanghai and Jidong cohort, which were in linkage disequilibrium with rs403815 (r^2^ > 0.5) and MAF > 0.05. Association analysis of each cohort and combined results implied these 5 SNPs did not associated with hypertension (Supplementary Table S4).

### CNV Burden Analyses

The total CNV burden in hypertensive cases was significantly greater than that in controls (Supplementary Methods and Supplementary Table S5). Eleven CNV regions were implicated by PLINK 1.07^14^ and further validated across 989 cases and 1,022 controls randomly selected from stage 2 by multiplex ligation-dependent probe amplification (MLPA) (Supplementary Table S6 and Supplementary Table S7). Among these, only 16q24.2 emerged as having a statistically significant association with hypertension (*P* = 0.048) (Supplementary Table S8).

### Functional Analyses of the *L3MBTL4* Locus

**Figure 1:**
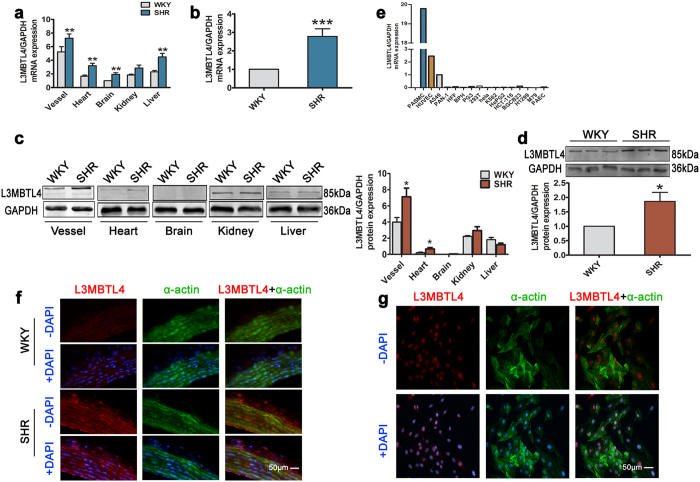
Expression and localization of *L3MBTL4*. **(a)** Relative mRNA expression of *L3MBTL4* in Wistar-Kyoto rats (WKYs) and spontaneously hypertensive rats (SHRs) (n = 8 each group). **(b)** Quantification of *L3MBTL4* mRNA level in the blood vessels (n = 12 per group). **(c)** Western blot analysis of *L3MBTL4* protein expression relative to GAPDH from tissues (n = 3–6 each group). **(d)** Increased protein levels of *L3MBTL4* in the vessel from SHR are verified by western blot (n = 4 each group). **(e)** Expression of *L3MBTL4* mRNA in different cell lines. **(f–g)** Representative immunofluorescence images of *L3MBTL4* (red) and α-actin (green) in the vessels from WKY and SHR **(f)**, as well as cultured vascular smooth muscle cells **(g)**. Nuclei are stained with DAPI (blue). Scale bars are 50 μm. n = 3 independent experiments for **(e–g)**, *p < 0.05, **p < 0.01, ***p < 0.001 compared to WKY. All data represent mean ± s.e.m.

The newly identified SNP rs403814 is located in an intron of the *L3MBTL4* gene at 18p11.31 (Supplementary Fig. S4). *L3MBTL4* is known as lethal(3) malignant brain tumor-like protein 4. To determine the expression and distribution of *L3MBTL4* in hypertension, we performed q-PCR and Western blotting, and compared *L3MBTL4* mRNA and protein expression from different tissues of SHRs, the most widely-used animal model for hypertension, and WKYs, a normotensive reference group. We found more abundant *L3MBTL4* mRNA and protein expression in the blood vessels of SHRs (Fig. 1a,c) and further validated the enhancement in vessels using another rats (Fig. 1b,d). In different human cell lines, we found *L3MBTL4* to be highly expressed in SMCs and endothelial cells (Fig. 1e). Double immunofluorescence staining revealed co-localization of *L3MBTL4* with α-actin, indicating that *L3MBTL4* is predominant in the medial layer of the vasculature (Fig. 1f). Due to the limitation of frozen sections staining in clarity and the complex structure of aortic tissue, we are not sure about the subcellular localization of *L3MBTL4*, although *L3MBTL4* mainly co-localized with α-actin, and distributed in the medial layer of the vasculature. To address this issue, further analysis was performed using vascular SMCs (Fig. 1g). According to our findings, the cellular localization of *L3MBTL4* is mainly in nucleus, which is consistent with the product datasheet of *L3MBTL4* antibody.

**Figure 2:**
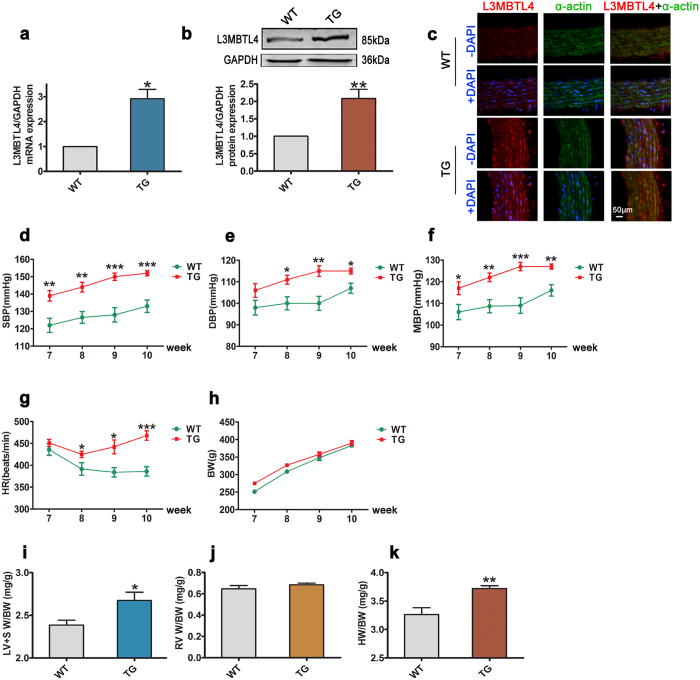
Blood pressure and cardiac parameters of *L3MBTL4* transgenic rats. **(a)** Relative mRNA expression of *L3MBTL4* in the blood vessels from wild type rats (WTs) and transgenic rats (TGs) (n = 7 each group). **(b)** *L3MBTL4* protein levels in the vasculature are measured by western blot analysis (n = 7 per group). (**c**) Representative immunofluorescence images of *L3MBTL4* (red) and α-actin (green) in the vessels from WTs and TGs. Nuclei are stained with DAPI (blue). Scale bars are 50 μm. n = 3 independent experiments. **(d–h)** Systolic blood pressure (SBP), diastolic blood pressure (DBP), mean blood pressure (MBP), heart rate (HR) and body weight (BW) of WTs and TGs (n = 8 each group). **(j,k)** Quantitative analysis of left ventricle + septum weight/body weight (LV + SW/BW), right ventricle weight/body weight (RVW/BW) and heart weight/body weight (HW/BW) ratios between groups (n = 6 each group). *p < 0.05, **p < 0.01, ***p < 0.001 compared to WT. All data represent mean ± s.e.m.

**Figure 3:**
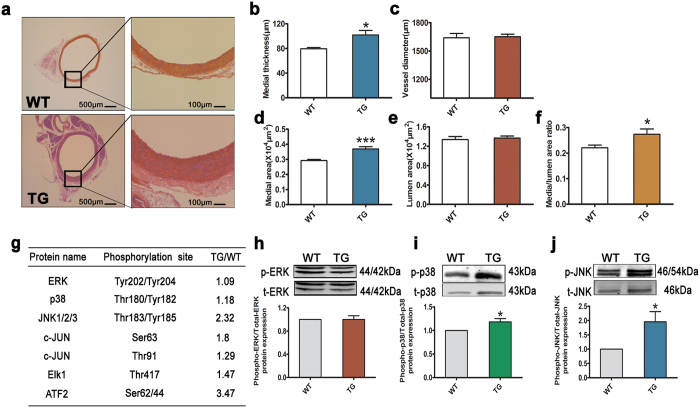
*L3MBTL4* induces vascular remodeling via MAPK signaling pathway. **(a)** Representative photomicrographs of hematoxylin-eosin staining in blood vessels from wild type rats (WTs) and transgenic rats (TGs) (n = 7 each group). **(b–f)** The media thickness, vessel diameter, media area, lumen area and media/lumen area ratio of aortas is quantified (n = 7 per group). Scale bars are 500 μm and 100 μm. **(g)** Changed phosphorylated proteins in the vessels of TGs compared to WTs are identified by phospho-antibody microarray. Listed are proteins in mitogen-activated protein kinases (MAPK) family. **(h–j)** Western Blot analysis validate the phosphorylation levels of extracellular signal-regulated kinase (ERK), p38MAPK and c-Jun N-terminal kinase (JNK) in the aortas from WTs and TGs; n = 7 per group for **(h,j)**, n = 6 each group for **(i)**. *p < 0.05, ***p < 0.001 compared to WT. All data represent mean ± s.e.m.

To investigate whether *L3MBTL4* participates causally in the development of hypertension, we constructed *L3MBTL4* transgenic rats (TGs) and confirmed increased *L3MBTL4* expression, at both the mRNA and protein levels in the vasculature (Fig. 2a–c). When compared to wild-type rats (WTs), TGs had similar body weights (Fig. 2h) but higher SBP, DBP, MBP and HR (Fig. 2d–g). TGs also had higher left ventricle and septum weight/body weight ratios (LV + SW/BW) and heart weight/body weight ratios (HW/BW), but no substantial changes were observed in the right ventricle weight/body weight ratio (RVW/BW) (Fig. 2i–k). These findings suggest that *L3MBTL4* might be an important inducer of elevated BP and cardiac hypertrophy. To explore the functional role of *L3MBTL4* more specifically in the vasculature, we undertook a series of histological analyses. The results of these showed an increase in the thickness and area of the vasculature media layer in TGs compared to WTs, as well as a higher media/lumen area ratio in TGs, without any difference in vessel diameter or lumen area between TGs and WTs (Fig. 3a–f).

To identify the signaling pathways involved in vascular remodeling mediated by *L3MBTL4*, we performed high-throughput protein phosphorylation profiling on cell lysates from the aortas of WTs versus TGs using the phospho-antibody microarray. The spectrum of proteins whose phosphorylation levels were changed for more than 20% was shown in Supplementary Table S9. Extracellular signal-regulated kinase (ERK), p38MAPK and c-Jun N-terminal kinase (JNK) comprise three key families of MAPK, that play central roles in cellular growth and proliferation. We found the phosphorylation levels of p38MAPK and JNK to be upregulated in TGs compared to WTs, while phospho-ERK hardly differed between the two. Moreover, downstream transcription factors of p38MAPK and JNK, including c-JUN, Elk1, and ATF2 were phosphorylated by more than 20% in TG (Fig. 3g). We further confirmed that *L3MBTL4* did affect the phosphorylation of p38MAPK and JNK, but not ERK (Fig. 3h–j). Together, these results led us to speculate that activation of the MAPK pathway may be a central mechanism by which *L3MBTL4* affects vascular remodeling.

**Figure 4:**
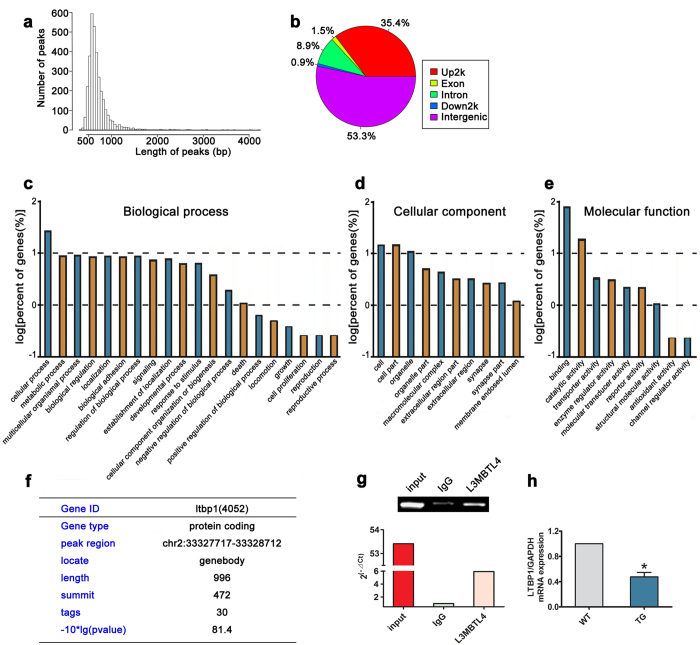
ChIP sequencing data of DNA bound by L3MBTL4 in HASMCs. **(a)** The distribution of peak length (bp) is indicated. **(b)** The location of ChIP-sequencing peaks in genome regions. **(c–e)** Gene Ontology categories of L3MBTL4 binding genes in biological process, cellular component and molecular function. **(f)** LTBP1 is a target gene interacting with L3MBTL4. **(g)** The enrichment of *LTBP1* peak regions in immunoprecipitated DNA fragments were determined by q-PCR; “input” indicates total DNA, “IgG” represents DNA fragments binding with control IgG, “L3MBTL4” means DNA fragments binding with anti-L3MBTL4 antibody. Data shown are from three independent experiments. **(h)** Relative mRNA expression of LTBP1 in the vasculature of WTs and TGs (n = 7 for each group). *p < 0.05 compared to WT. All data represent mean ± s.e.m.

Chromatin DNA from human aortic smooth muscle cells (HASMCs) was immunoprecipitated with *L3MBTL4* antibody and sequenced to search for downstream targets of *L3MBTL4*. 3,289 peaks were yielded and annotated using the UCSC database (University of California Santa Cruz), and 1362 of them were successfully located to a gene (Fig. 4a, Supplementary Table S10). Based on their location in genome elements, these peaks were classified into exon, intron, upstream, intergenic, and downstream regions (Fig. 4b). Gene Ontology analyses of genes mapping to the sequenced peaks indicated enrichment of genes playing crucial roles in biological processes, cellular components and molecular functions (Fig. 4c–e). Among the 1362 genes, we speculated that *LTBP1* might participate in the effect of *L3MBTL4* on vascular remodeling and hypertension, as there are evidence that anomalous expression of *LTBP1* is detected in thoracic aortic aneurysm, and may promote the development of arterial diseases^15^,^16^, Therefore the protein coding gene *LTBP1* (Gene ID 4052) was further focused (Fig. 4f). *LTBP1* was validated for *L3MBTL4* association, which showed higher abundance in immunoprecipitated samples compared to the control IgG (Fig. 4g). Repressed transcription activities of *LTBP1* was confirmed in the blood vessels of TGs (Fig. 4h). Together, these results strongly suggest that *L3MBTL4* directly targets to *LTBP1*.

Given altered MAPK family upon *LTBP2* knockdown^17^, we speculate the increased activity of MAPKs by *L3MBTL4* is possibly due to the down-regulation of *LTBP1*. To further reveal the direct effects of *LTBP1* on MAPKs activation, we inhibited the expression of *LTBP1* by siRNA (Supplementary Fig. S5a). It was noted that siRNA target to *LTBP1* conduced to a increase of p38MAPK and JNK phosphorylation (Supplementary Fig. S5b,c). Thus *LTBP1* is responsible for the activation of MAPKs, and repressed *LTBP1* may be a primary factor in the effect of L3MBTL4 on vascular remodeling and hypertension.

## Discussion

By using an extreme case-control strategy in a three-staged genetic study of up to 21,990 individuals of Chinese population, we identified eleven loci associated with hypertension (*P* < 0.05). Seven of these loci in five genes, *FGF5*, *ATP2B1*, *CYP17A1*, *MTHFR* and *CASZ1*, were reported as having an association with SBP, DBP and/or hypertension in European populations^9^,^11^,^17^. Replication studies in Asians^8^,^18^,^19^,^20^ confirmed the relationship between SNPs and BP or hypertension.

In this study, we also identified four new hypertension-associated variants in three genes, *LOC729251*, *L3MBTL4*, and *TCEANC*. Distribution of SNP rs403814 in *L3MBTL4* was significantly different between hypertensive participants and controls in the three stages and in the meta-analysis (*P*_*meta*_ = 6.128 × 10^−9^, OR = 1.15, 95% CI = 1.07–1.23). SNP rs403814 lies within the intron of the conserved domain on *L3MBTL4* gene. We did not find other SNPs on or near gene *L3MBTL4* or haplotypes to be associated with hypertension, indicated the important role of rs403814. Moreover, SNP rs403814 in intron of L3MBTL4 might be a binding site for a repressor, and a C to A transition abrogates the interaction with the repressor and thus leads to an up-regulation of L3MBTL4 expression, illustrating the importance of rs403814 in regulation of the gene expression. The three other loci (rs4243170 of *LOC729251* gene, rs2361159 and rs5935649 of *TCEANC* gene) nominally associated with hypertension (5 × 10^−4^ < *P*_*meta*_ < 0.05). Larger cohort and more genetic studies are needed to identified the association of these three SNPs and hypertension.

Very few studies have investigated associations between CNVs and hypertension^17^. To address this question, we sought to identify loci with significant associations with hypertension using a CNV burden analysis. The locus we identified by this approach is 16q24.2. This region has been shown to be associated with a number of diseases^21^,^22^ and low HDL-C^23^, but potential associations with BP have not been uncovered. Chromosome region 16q24.2 includes 9 genes that had no relationship with hypertension or blood pressure were reported. Mechanism that locus on 16q24.2 modulates blood pressure and generation of hypertension is unclear. This novel result promises to shed new light on the association between CNVs and hypertension. Refined analysis and functional investigations are required to understand its role in hypertension.

Previous research has revealed associations of *L3MBTL4* with several malignancies^24^,^25^,^26^. Recently, *L3MBTL4* has been found to be decreased in breast tumors, suggesting that it may act as a tumor suppressor^27^. To further understand the biological and cellular functions of *L3MBTL4*, especially within the context of hypertension, we performed a number of functional studies. Our finding that *L3MBTL4* over-expression results in increased BP and heart hypertrophy supports a pathogenic role for *L3MBTL4* in the context of hypertension that extends beyond its putative function as a tumor suppressor.

*L3MBTL4* over-expression was correlated with aorta thickening and activation of the MAPK signaling pathway. Alterations in the structure of blood vessels are thought to contribute to the development of hypertension by promoting increased vascular resistance^28^. In the present study, we found evidence of hypertrophic remodeling in the arterial structure, as indicated by an increased medial to lumen area ratio^29^. Activation of MAPK pathway components including p38MAPK, JNK and other downstream targets are known to drive cellular growth and proliferation, and several previously published studies support the association between activity of the MAPK signaling pathway and vascular remodeling^30^,^31^,^32^,^33^. These data propose a mechanistic model wherein *L3MBTL4* activates the MAPK pathway, triggering vascular remodeling and the eventual development of hypertension. Further studies are still required to understand the contribution of the cell cycle- and cell adhesion-regulatory functions of *L3MBTL4*^34^,^35^.

Moreover, the targeting of *LTBP1* by L3MBTL4 was demonstrated in the current work. *LTBP1* belongs to the fibrillin-LTBP superfamily^36^. There are evidence that anomalous expression of LTBP1 may promote the development of arterial diseases^15^. Decreased expression of LTBP1 is also detected in fibroblasts from thoracic aortic aneurysm^16^. In L3MBTL4 TGs, LTBP1 was significantly down-regulated whereas the activity of MAPK signaling was apparently up-regulated. Notably, the latest research findings identify altered MAPK family upon LTBP2 knockdown^37^. Consistently, siRNA specific to *LTBP1* induced elevated phosphorylation of p38MAPK and JNK. Therefore, the mechanism underlying L3MBTL4-induced MAPKs activation is depending on depressed expression of *LTBP1*. LTBP1 may be a primary factor in the effect of L3MBTL4 on vascular remodeling and hypertension. Taken together, the underlying mechanism of *L3MBTL4*-induced hypertension was summarized in Supplementary Fig. S6. Our data indicated that L3MBTL4 targets the *LTBP1* gene, and thereby represses its expression, which results in phosphorylation of p38MAPK and JNK, as well as their downstream factors. The activation of MAPK signaling is engaged in the progression of vascular remodeling associated with pathological hypertension.

In summary, the GWAS described in this paper, along with the two-staged follow-up study, identified *L3MBTL4* as a novel susceptibility gene in hypertension. Through i*n vitro* and *in vivo* experiments, we have shed further light on the biological function of *L3MBTL4* and the molecular mechanisms underlying BP regulation. We have also identified the locus, 16q24.2, as a potential hypertension-modulating locus given its great CNV burden. These findings yield new insights into the genetic and biological basis of hypertension and offer potential targets for future antihypertensive therapies.

## Methods

Supplementary methods (including complete methods for genetic study design, ethics and subjects, genotyping, quality control, imputation, SNP selection at each stage, CNV analyses and MLPA, statistical analyses, and functional study), Supplementary tables, and Supplementary figures are available in the on-line supplementary.

### Ethics Statement

The study was approved by the Institutional Ethical Committee of each hospital and was performed according to the Declaration of Helsinki principles. Peripheral blood and clinical information were collected from subjects who provided written informed consent.

### Subjects and BP Phenotyping

The study was designed in three stages using an extreme case-control strategy^10^. (Supplementary Methods and Supplementary Table S1). Five independent cohorts were recruited. In stage 1, Hypertensive cases were defined as having SBP ≥ 150 mmHg and/or DBP ≥ 90 mmHg in untreated subjects with an age of onset of ≤50 years. Normotensive controls were defined as having SBP ≤ 125 mmHg and DBP ≤ 80 mmHg without antihypertensive treatment. All controls were ≥65 years of age. In stage 2, hypertension and normotension were defined as described for stage 1, while the normotensive controls were ≥55 years of age. In stage 3, hypertension and normotension were defined as described for stage 1 but without age limitation. Individuals would be excluded who had secondary form of hypertension as evaluated by an extensive workup that included serum creatinine and electrolytes, urinalysis, and other hematologic screening tests (Supplementary Methods, Supplementary Fig. S1, and Supplementary Table S1).

### Genotyping and Quality Control

Genomic DNA was extracted from peripheral blood samples using the QIAGEN QIAamp DNA Mini Blood Kit (Hilden, Germany). Genotyping was performed using the HumanOmniZhongHua-8 Beadchip (Illumina, San Diego, California, U.S.) in stage 1, the Illumina GoldenGate assay (custom, 384 SNPs) in stage 2 and the MassARRAY iPLEX (Sequenom, San Diego, California, U.S.) in stage 3. Quality control and data filtering were performed according to the call rate (CR), minor allele frequency (MAF), and Hardy-Weinberg equilibrium test (HWE) (Supplementary Methods).

### CNV burden Analyses and MLPA

CNV burden was indicated by PLINK 1.07^14^ based on the genotyping results of stage 1 and then replicated in an additional cohort (989 cases and 1,022 controls randomly selected from stage 2) by multiplex ligation-dependent probe amplification (MLPA) method^38^ (Supplementary Methods and Supplementary Table S5–S9).

### Statistics

The associations between SNPs and SBP, DBP as well as hypertension were assessed using the Cochran-Armitage trend test. Manhattan plots were generated using Haploview software (V4.2). METAL software^39^ was used for the meta-analyses. Heterogeneity was examined using Cochran’s Q and I^2^ statistics to assess diversity across different studies. A fixed-effects model was applied if *P*_het_ for Q was > 0.05; a random-effects model was adopted if *P*_het_ for Q was < 0.05. The significance threshold was set at *P* < 0.05. For quantitative trait analyses, the genetic effects estimated in each of the multistage panels were combined using the inverse variance method. Regional association plots were generated using Locus Zoom v1.3^40^. Haplotypes were estimated using the software PLINK 1.07^14^. We used Pearson correlation to estimate the CNV rate differences between cases and controls. The significance threshold was set at *P* < 0.05. Details of statistics are showed in Supplementary Methods.

### Gene Expression Analysis

Quantitative real-time PCR (q-PCR) and Western blotting were performed to estimate mRNA and protein concentrations of the susceptibility genes selected from this genetic study on tissues from spontaneously hypertensive rats (SHRs) and Wistar-Kyoto rats (WKYs). Double immunofluorescence staining was used to localize the protein encoded by susceptibility gene in vasculature and smooth muscle cells (SMCs) (Supplementary Methods).

### Rats Model Construction and Phenotype Analysis

Transgenic rats were constructed to explore functional roles of susceptibility gene. Systolic BP (SBP), diastolic BP (DBP), mean BP (MBP) of rats were measured by no-invasive and invasive methods. Weight of different parts of heart were measured to estimate cardiac hypertrophy. Thickness and area of vasculature media layer were measured by histological analyses (Supplementary Methods).

### Pathway Analysis and Target Gene Identification

Phospho-antibody microarray was used to analysis high-throughput protein phosphorylation profiling on cell lysates from the aortas and identify probable signaling pathways. Chromatin immunoprecipitation (ChIP) and Gene Ontology analyses were used to indicated downstream targets of susceptibility gene (Supplementary Methods, Supplementary Table S12 and Supplementary Table S13).

## Additional Information

**How to cite this article**: Liu, X. *et al*. Genome Wide Association Study Identifies *L3MBTL4* as a Novel Susceptibility Gene for Hypertension. *Sci. Rep*. **6**, 30811; doi: 10.1038/srep30811 (2016).

## Supplementary Material

Supplementary Information

## Figures and Tables

**Table 1:** Combined results of the 18 SNPs genotyped in stage 1, 2 and 3.

chr	SNP ID	position	minor/major allele	gene	location	group	OR(95% CI)	*P*
1	rs12749808*	158065762	C/T	*KIRREL*	UTR	stage1	1.47 (1.15, 1.88)	1.315 × 10^−4^
						stage2	1.09 (1.00, 1.18)	0.004
						Jidong	1.02 (0.93, 1.12)	0.678
						Shanghai	0.82 (0.69,1.02)	0.541
						Shantou	0.99 (0.92, 1.06)	0.776
						meta	1.03 (0.86, 1.20)	0.687
1	**rs17367504**	11862778	G/A	*MTHFR*	INTRON	stage2	1.42 (1.20, 1.67)	3.918 × 10^−5^
						Jidong	1.20 (0.67, 2.17)	0.197
						Shanghai	1.05 (0.93, 1.20)	0.404
						Shantou	1.22 (1.03, 1.45)	0.009
						meta	1.21 (1.09, 1.30)	3.810 × 10^−4^
1	**rs880315**	10796866	A/G	*CASZ1*	INTRON	stage2	0.88 (0.80, 0.96)	0.003
						Jidong	0.98 (0.89, 1.08)	0.771
						Shanghai	0.93 (0.86, 0.99)	0.009
						Shantou	0.86 (0.80, 0.93)	0.000924
						meta	0.91 (0.86, 0.95)	1.814 × 10^−4^
4	**rs1458038**	81164723	A/G	*FGF5*	UTR	stage2	1.30 (1.20, 1.42)	8.853 × 10^−10^
						Jidong	0.96 (0.88, 1.06)	0.524
						Shanghai	1.12 (1.05, 1.20)	0.005
						Shantou	1.10 (1.03, 1.18)	5.235 × 10^−5^
						meta	1.12 (1.01, 1.24)	0.037
4	**rs16998073**	81184341	A/T	*FGF5*	UTR	stage2	1.32 (1.21, 1.44)	1.799 × 10^−10^
						Jidong	0.99 (0.90, 1.09)	0.847
						Shanghai	1.14 (1.06, 1.22)	1.495 × 10^−4^
						Shantou	1.12 (1.04, 1.20)	3.182 × 10^−5^
						meta	1.14 (1.03, 1.26)	0.007
8	rs1799998	143999600	C/T	*CYP11B2*	UTR	stage2	0.80 (0.73, 0.87)	1.099 × 10^−6^
						Jidong	1.04 (0.94, 1.16)	0.418
						Shanghai	0.93 (0.86, 1.00)	0.048
						Shantou	0.94 (0.87, 1.01)	0.469
						meta	0.92 (0.84, 1.02)	0.166
9	kgp9568150*	66749500	A/G	*C9orf98*	INTRON	stage1	1.85 (1.31, 2.61)	4.385 × 10^−4^
						stage2	0.82 (0.73, 0.92)	5.812 × 10^−4^
						Jidong	1.03 (0.91, 1.17)	0.645
						Shanghai	1.00 (0.91,1.10)	0.127
						Shantou	1.01 (0.92, 1.11)	0.846
						meta	1.03 (0.90, 1.18)	0.714
10	**rs11191548**	104846178	C/T	*CYP17A1*	UTR	stage2	0.85 (0.77, 0.93)	5.388 × 10^−4^
						Jidong	0.92 (0.83, 1.02)	0.160
						Shanghai	0.95 (0.87, 1.02)	0.345
						Shantou	0.92 (0.85, 0.99)	0.027
						meta	0.91 (0.85, 0.95)	7.584 × 10^−5^
10	rs1801253	115805056	C/G	*ADRB1*	CODING	stage2	0.89 (0.81, 0.98)	0.008
						Jidong	1.01 (0.91, 1.13)	0.644
						Shanghai	0.99 (0.91, 1.07)	0.363
						Shantou	0.99 (0.92, 1.07)	0.564
						meta	0.97 (0.92, 1.02)	0.251
11	rs12421938*	11422750	G/A	*GALNT18*	INTRON	stage1	0.63 (0.42, 0.96)	3.522 × 10^−4^
						stage2	0.81 (0.71, 0.93)	0.002
						Jidong	0.85 (0.73, 1.00)	0.051
						Shanghai	0.91 (0.81, 1.01)	0.166
						Shantou	1.12 (1.08, 1.23)	0.034
						meta	0.89 (0.77, 1.02)	0.124
12	**rs17249754**	90060586	A/G	*ATP2B1*	INTERGENIC	stage2	0.85 (0.78, 0.92)	1.785 × 10^−4^
						Jidong	0.99 (0.90, 1.10)	0.967
						Shanghai	0.94 (0.88, 1.01)	0.139
						Shantou	0.85 (0.79, 0.91)	8.582 × 10^−4^
						meta	0.90 (0.84, 0.97)	0.007
12	**rs2681472**	90008959	C/T	*ATP2B1*	INTRON	stage2	0.87 (0.80, 0.95)	0.001
						Jidong	1.01 (0.91, 1.11)	0.950
						Shanghai	0.94 (0.87, 1.01)	0.157
						Shantou	0.87 (0.81, 0.94)	4.243 × 10^−4^
						meta	0.91 (0.85, 0.98)	1.443 × 10^−5^
12	rs2074356	112645401	T/C	*HECTD4*	INTRON	stage2	0.78 (0.69, 0.89)	1.632 × 10^−4^
						Jidong	0.99 (0.86, 1.15)	0.920
						Shanghai	0.92 (0.83, 1.01)	0.099
						Shantou	1.03 (0.94, 1.13)	0.224
						meta	0.93 (0.83, 1.04)	0.288
16	rs4243170*	79437209	A/G	*LOC729251*	INTERGENIC	stage1	0.59 (0.44, 0.78)	2.782 × 10^−4^
						stage2	0.87 (0.79, 0.96)	0.005
						Jidong	0.95 (0.85, 1.06)	0.379
						Shanghai	0.95 (0.88, 1.03)	0.199
						Shantou	0.98 (0.91, 1.06)	0.839
						meta	0.88 (0.78, 1.00)	0.029
17	rs17225738*	5861557	A/G	*NLRP1*	INTERGENIC	stage1	0.63 (0.49, 0.82)	3.614 × 10^−4^
						stage2	0.89 (0.81, 0.97)	0.008
						Jidong	1.01 (0.91, 1.12)	0.655
						Shanghai	1.02 (0.95, 1.09)	0.180
						Shantou	0.99 (0.92, 1.06)	0.561
						meta	0.91 (0.81, 1.02)	0.210
18	rs403814*	6282593	G/T	*L3MBTL4*	INTRON	stage1	1.39(1.01, 1.94)	4.699 × 10^−4^
						stage2	1.24 (1.12, 1.36)	1.321 × 10^−5^
						Jidong	1.14 (1.01, 1.29)	0.006
						Shanghai	1.15 (1.06, 1.25)	0.003
						Shantou	1.06 (0.98, 1.14)	0.177
						meta	1.15 (1.07, 1.23)	6.128 × 10^−9^
23	rs2361159*	13681115	C/T	*TCEANC*	CODING	stage1	1.21 (1.12, 1.29)	2.813 × 10^−4^
						stage2	1.10 (1.02, 1.15)	0.008
						Jidong	1.20 (0.89, 1.52)	0.991
						Shanghai	1.03 (1.01, 1.10)	0.034
						Shantou	1.04 (0.98, 1.14)	0.383
						meta	1.07 (1.02, 1.12)	0.002
23	rs5935649*	13681638	C/T	*TCEANC*	CODING	stage1	1.24 (1.10, 1.30)	4.687 × 10^−4^
						stage2	1.11 (0.89, 1.09)	0.006
						Jidong	1.21 (1.04, 1.55)	0.825
						Shanghai	1.02 (1.00, 1.06)	0.021
						Shantou	1.06 (0.97, 1.15)	0.226
						meta	1.08 (1.03, 1.14)	7.911 × 10^−4^

CHR, chromosome; OR, odds ratio; 95% CI, 95% confidence interval; stage 1, the genome-wide association study; stage 2, the follow-up study; stage 3, the replication study; meta, meta-analysis of stages 1, 2 and 3. SNPs with bold font are significantly associated with hypertension (P < 0.05). Newly identified loci are indicated by asterisks.
