# Supplementary material for: Genome Wide Association Study Identifies L3MBTL4 as a Novel Susceptibility Gene for Hypertension
Source: Sci Rep. 2016 Aug 2;6:30811. doi: 10.1038/srep30811 (PMC4969609; doi:10.1038/srep30811)
Supplement: Supplementary Information [file srep30811-s1.pdf]

Supplementary Information

Genome Wide Association Study Identifies *L3MBTL4* as a Novel Susceptibility Gene for Hypertension

Xin Liu, PhD[1,2§]; Cheng Hu, MD, PhD[3,4§]; Minghui Bao, MD[5§]; Jing Li, PhD[5,6§]; Xiaoyan Liu, PhD[7§]; Xuerui Tan, MD[8]; Yong Zhou, MD[9]; Yequn Chen, MD[8]; Shouling Wu, MD[10]; Shuohua Chen, MD[10]; Rong Zhang, MD[3]; Feng Jiang, MD[3]; Weiping Jia, MD[3]; Xingyu Wang, MD, PhD[1,2]*; Xinchun Yang, MD[5]*; Jun Cai, MD, PhD[11,12,13]*

[1]National Research Institute for Family Planning, Beijing, China;

[2]Beijing Hypertension League Institute, Beijing, China;

[3]Shanghai Diabetes Institute, Shanghai Jiao Tong University Affiliated Sixth People's Hospital, Shanghai, China;

[4]Institute for Metabolic Diseases, Shanghai Jiao Tong University Affiliated Sixth People's Hospital South Campus, Shanghai, China;

[5]Department of Cardiology, Beijing Chaoyang Hospital, Capital Medical University, Beijing, China;

[6]Beijing Key Laboratory of Hypertension, Beijing, China;

[7]Medical Research Center, Beijing Chaoyang Hospital, Capital Medical University, Beijing, China;

[8]Department of Cardiology, The First Affiliated Hospital of Shantou University Medical College, Shantou, Guangdong, China;

[9]Department of Cardiology, Beijing Friendship Hospital, Capital Medical University, Beijing, China;
[10]Department of Cardiology, Kailuan General Hospital, Hebei Union University, Tangshan, Hebei, China;
[11]Department of Hypertension, Fuwai Hospital, Beijing, China;
[12]State Key Laboratory of Cardiovascular Disease, Beijing, China;
[13]National Center for Cardiovascular Diseases, Chinese Academy of Medical Sciences and Peking Union Medical College, Beijing, China.

Correspondence to: Jun Cai, MD, PhD, Department of Hypertension, Fuwai Hospital, State Key Laboratory of Cardiovascular Disease, National Center for Cardiovascular Diseases, Chinese Academy of Medical Sciences and Peking Union Medical College, Xicheng District, North Lishi Road No. 167, Beijing, China, 100037. Tel: 86-10-88398530; Fax: 86-10-88398530; E-mail: caijun@fuwaihospital.org.

[§]Drs Xin Liu, Cheng Hu, Minghui Bao, Jing Li, and Xiaoyan Liu contributed equally as co-first authors.
*Drs Jun Cai, Xinchun Yang, and Xingyu Wang contributed equally as co-corresponding authors.

Supplementary Methods

Ethics and subjects. Peripheral blood and clinical information were collected from subjects who provided written informed consent. The study was approved by the Institutional Ethical Committee of each hospital and was performed according to the Declaration of Helsinki principles. Peripheral blood (4 ml per person) and clinical information were collected from subjects who provided written informed consent. Blood pressure levels were classified according to the Hypertension Guidelines for the Management of Hypertension.[1]

Genetic study design. Five independent cohorts were recruited for the three-stage genetic study. The detailed characteristics of the subjects analyzed at each stage are described in **Supplementary Table S1 online**. In stage 1, we conducted a case-control study of the genome wide association study (GWAS) scan. A total of 528 hypertensive cases and normotensive controls were recruited. Cases were enrolled from clinical practices or annual medical checkups at medical institutions according to uniformly defined criteria. To increase the power of discovery stage, we conducted an extreme case-control strategy which using cases and controls with dramatically distinct distributed blood pressure phenotype. Hypertensive cases were defined as: (i) SBP ≥150 mmHg and/or DBP ≥90 mmHg for untreated subjects; (ii) no secondary form of hypertension as evaluated by an extensive workup that included serum creatinine and electrolytes, urinalysis, and other hematologic screening tests; (iii) age of onset ≤50 years. Normotensive controls were defined as: (i) SBP ≤125 mmHg and DBP ≤80 mmHg without antihypertensive treatments; (ii) age of onset

≥65 years. In stage 2, 4608 hypertensive cases and normotensive controls were selected for follow-up study from clinical practices or annual medical checkups at medical institutions. Hypertension and normotension were defined as described for stage 1, while the normotensive controls were ≥55 years of age. For CNV analysis, we randomly selected 989 cases and 1022 controls to be further tested by MLPA method. In stage 3, a replication study was conducted and included subjects from 3 independent cohorts. A total of 17,318 hypertensive cases and normotensive control from Jidong, Shanghai and Shantou study. hypertension and normotension were defined as described for stage 1 but without age limitation. Subjects of Jidong cohort were participants in the baseline survey of the Jidong Cohort Study, which was recruited from annual medical checkups of Jidong oil field company (located in Tangshan, an industrial city in Northern China) from 2009 to 2010, aged 25 to 65 years. The cohort comprised 6,423 persons and 5,648 of them have DNA available to be genotyped. Shanghai cohort recruited about 8,000 participants from 3 communities of Shanghai in 2012. Information about demographic factors, lifestyle, and history of disease (such as hypertension) was obtained using structured questionnaires. Participants of Shantou cohort were recruited from clinic and medical examination center of The First Affiliated Hospital of Shantou University Medical College in 2012-2013. Combined analyses of data from across all three stages were used in a meta-analyses to confirm the candidate loci across all subjects.

Three clinic blood pressure (BP) measurements were obtained from each participant in the sitting position after 5 min of rest. In addition, participants were advised to avoid alcohol, cigarette smoking, coffee/tea, and exercise for at least 30 min before their BP

measurements. The average of three measures of BP was used in this study.

Genotyping, quality control, and imputation. For stage 1, genome-wide genotyping was performed using the Beadchip (Illumina, San Diego, California, U.S.), which covers ~900,000 genetic markers and contains both common and rare loci discovered by the HapMap Project and 1000 Genomes Project. Data clean-up and analyses were performed using the PLINK 1.07 software. Before the association analyses, quality control was performed on the raw genotyping data to filter out SNPs and samples of low quality. SNPs were excluded based on the following criteria: minor allele frequency (MAF)≤1%, significant deviation from Hardy-Weinberg equilibrium test (HWE, $P<10^{-4}$), SNP call rate (CR)<97%, no-random missing haplotype ($P<10^{-9}$) or mismatched positions between Illumina, dbSNP build 126, and/or HapMap phase II+III (CHB+JPT). After data filtering and imputation, 727,172 SNPs and 518 samples (271 cases and 247 controls) remained for subsequent analyses. The population stratification was checked by multidimensional scaling analyses of the pair-wise distances between samples measured over all SNPs.

For stage 2, genotyping was performed using the Illumina GoldenGate assay (custom, 384 SNPs) according to manufacturer's instructions. A total of 358 SNPs associated with SBP, DBP, and hypertension in stage 1 and 26 SNPs previously reported to be associated with elevated SBP, DBP, or hypertension were genotyped. SNPs were excluded based on the following criteria: MAF≤5%, $P<10^{-3}$ in HWE test, or SNP CR<97%. After data filtering, 371 SNPs and 4,502 samples were retained for further analyses.

For stage 3, samples were genotyped using MassARRAY iPLEX (Sequenom, San Diego,

California, U.S.) according to the manufacturer's instructions. A total of 18 SNPs in stage 2 were selected. The overall CR exceeded 98%.

Additive 5 SNPs on L3MBTL4 gene were genotyped also by using MassARRAY iPLEX (Sequenom, San Diego, California, U.S.). The overall CR exceeded 99%.

CNV analyses and MLPA. Based on the genotyping results of stage 1, we analyzed the potential loci containing CNVs using PLINK 1.07. A total of 11 chromosomal regions (1p36.33, 1q31.3, 7q36.3, 17p13.1, 4p16.1, 5q21.3, 5q34, 8q24.23, 2q32.1, 16q24.2 and 20p12.1) were suggested to contain CNVs. These 11 loci were further analyzed in 989 cases and 1,022 controls. According to the principle of MLPA[2] we designed three pairs of probes matching highly conserved segments of the target CNVs and reference genome **(Supplementary Table S11 online)**. The peak areas in capillary electrophoresis of the three different products of each CNV were calculated to ensure accuracy of the experimental results.

Statistics. The associations between SNPs and SBP, DBP as well as hypertension were assessed using the Cochran-Armitage trend test. Manhattan plots were generated using Haploview software (V4.2). METAL software[2] was used for the meta-analyses. Heterogeneity was examined using Cochran's Q and I^2 statistics to assess diversity across different studies. A fixed-effects model was applied if P_{het} for Q was >0.05; a random-effects model was adopted if P_{het} for Q was <0.05. The significance threshold was set at $P<0.05$. For quantitative trait analyses, the genetic effects estimated in each of the multistage panels were combined using the inverse variance method. Regional association plots were

generated using Locus Zoom v1.3.[3] We used Pearson correlation to estimate the CNV rate differences between cases and controls. The significance threshold was set at $P<0.05$. Except for mentioned software, calculations were performed by PLINK 1.07.[4]

Functional study. L3MBTL4 and LTBP1 antibodies were purchased from Abcam (Cambridge, UK). Antibodies specific to GAPDH, α-actin, β-actin, phospho-ERK, ERK, phospho-p38, p38, phospho-JNK, and JNK were purchased from the Beyotime Institute of Biotechnology. siRNA was synthesize by RiboBio Co. Ltd.

Quantitative real-time PCR. Total RNA was extracted from frozen rat tissue and cultured cell lines using Trizol reagent (Invitrogen). Isolated RNA was reverse-transcribed using the PrimeScript 1st Strand cDNA Synthesis Kit (Takara) according to manufacturer's instructions. Subsequently, diluted cDNA was used as a template in real-time PCR with the SYBR Premix Ex Taq kit (Takara) on an ABI Prism 7500 sequence detection system (Applied Biosystems, Foster, CA, USA). Gene-specific primers are listed in **Supplementary Table S13 online.** GAPDH was used as an endogenous control, and the relative mRNA level of each sample was analyzed and calculated using the $2^{-\Delta\Delta Ct}$ method.

Western blot analyses. Rat tissues were homogenized with cold lysis buffer, sonicated, and centrifuged to obtain the supernatants. The protein concentration in the supernatant was determined by the bicinchoninic acid protein assay. Equal amounts of protein were fractionated on SDS-PAGE and transferred to nitrocellulose membranes (Millipore, USA). The membranes were blocked and incubated with primary antibodies against L3MBTL4, phospho-ERK, ERK, phospho-p38, p38, phospho-JNK, JNK, GAPDH, or β-actin. After binding

with secondary antibodies, the protein bands were detected using the Odyssey infrared imaging system (LI-COR).

Immunofluorescence staining. After being fixed with 4% paraformaldehyde and dehydrated with 20-30% sucrose, rat aortas were embedded in OCT and sectioned (10 μm). Paraformaldehyde (4%) was also applied to fixed cultured vascular smooth muscle cells. Frozen sections and fixed vascular smooth muscle cells were permeabilized with Triton X-100 and double-stained with antibodies specific to L3MBTL4 and α-actin overnight at 4°C. The samples were then incubated with Cy3- and FITC-coupled secondary antibodies and nuclei counterstained with DAPI. Images were captured with a fluorescence microscope (Nikon).

Animal experiments. Animal experiments were performed according to the guidelines of the Animal Ethics Committee of University. WKYs and SHRs aged 8-12 weeks were purchased from Charles River Laboratories. The *L3MBTL4* transgenic rats were constructed by Cyagen Bioscience Inc. Briefly, the DNA fragment containing pRP.ExBi-EF1α-L3MBTL4-IRES-eGFP was prepared and microinjected into the eggs of female SD rats. Genomic DNA was isolated using tail digestion buffer (KCl 50 mM, Tris-HCl 10 mM, Triton X-100 0.1 %, proteinase K 0.4 mg/mL). The genotype was verified by PCR analyses. The sequences of specific primers are listed in **Supplementary Table S12 online.** In the end, we anesthetized the male rats aged 12 weeks in each group with pentobarbital injection (120 mg/kg, i.p.), opened the thorax, collected the heart and dissected the aorta for further examination.

Blood pressure measurement. Rat SBP and DBP were measured once per week using

the tail-cuff method and the BP-98A system (Softron, Tokyo, Japan). At the end of this study, the animals were anesthetized with isoflurane and invasive blood pressure measured by inserting a polyethylene tube pre-filled with heparinized normal saline into the carotid artery. These data were recorded in the physiological data acquisition and analyses system.

HE staining. The rat aortas were fixed with 4% paraformaldehyde and embedded in paraffin. Sections were stained with hematoxylin-eosin to determine the morphology of the aorta. The staining was examined with a fluorescence microscope (Nikon).

Phosphoprotein profiling by PhosphoExplorer antibody microarray. Cell lysates obtained from the aortas of WT and TG rats were applied to the Pathway Phosphorylation Antibody Array (PEX100) designed by Full Moon Biosystems Inc. The array contains 1,318 antibodies. Each of the antibodies has two replicates printed on coated glass microscope slides, along with multiple positive and negative controls. The antibody array experiment was performed by Wayen Biotechnology. Briefly, cell lysates were biotinylated using the AntibodyArray Assay Kit (Full Moon Biosystems, Inc.). The antibody microarray slides were blocked in a blocking solution, rinsed and dried with compressed nitrogen, then incubated with the biotin-labeled cell lysates in coupling solution, washed and rinsed before detecting bound biotinylated proteins using Cy3-conjugated streptavidin. The slides were scanned on a GenePix 4000 scanner and the images analyzed using GenePix Pro 6.0 (Molecular Devices, Sunnyvale, CA). The fluorescence signal (I) of each antibody was obtained from the fluorescence intensity of the antibody spot. A ratio was computed to measure the extent of protein

phosphorylation as follows: phosphorylation ratio = phospho value /unphospho value.

Chromatin immunoprecipitation (ChIP). Approximately 10^7 h*uman* aortic smooth muscle cells (*HASMCs*) were cross-linked using 1% formaldehyde and lysed with IP buffer (150 mM NaCl, 5 mM EDTA, 1% Triton X-100, 0.5% NP-40, 50 mM Tris–HCl (pH 7.5) and 0.5 mM DTT) containing 0.5 mM phenylmethlysulfonyl fluoride. The DNA fragments were sheared into 100-500 bp fragments using a sonicator microprobe (Misonix 3000). The sheared chromatin fraction was incubated overnight with L3MBTL4 antibody. Protein A beads were added to pull down the protein-DNA complexes. After washing and elution, the proteins were digested with proteinase K, and DNA was extracted and purified. Sequencing was performed by Tianjin GenoBio Technology Co., Ltd. First, the DNA-end was repaired to have a 3'-dA overhang, and then adapters were ligated to the end DNA fragments. A qualified library for sequencing was constructed and DNA fragments with the proper size (usually 100-300 bp, including adaptor sequence) selected. The reads enrichment region (peak) was scanned from the whole genome to obtain related genes. Gene Ontology analyses was performed. Finally, the enrichment of *LTBP1* peak regions in immunoprecipitated DNA fragments were determined by q-PCR using the primers listed in **Supplementary Table S14**, and the relative expression of *LTBP1* in TGs was confirmed with primers shown in **Supplementary Table S12 online**.

Supplementary Tables Indexes

Supplementary Table S1. Baseline characteristics of subjects. 12

Supplementary Table S2. SNPs genotyped in the follow-up study (stage 2). 13

Supplementary Table S3. Haplotype analysis in the follow-up study (stage 2). 27

Supplementary Table S4. Five SNPs linkage disequilibrium with rs403815 did not associated with hypertension. 29

Supplementary Table S5. Total CNV burden among cases and controls. 30

Supplementary Table S6. 11 CNV regions implicated by PLINK. 31

Supplementary Table S7. Baseline characteristics of participants for CNV analysis. 32

Supplementary Table S8. CNV discovery of 16q24.2 locus. 33

Supplementary Table S9. The spectrum of phosphorylation proteins as detected by phospho-antibody microarray. 34

Supplementary Table S10. The 3,289 peaks obtained by ChIP sequencing. 46

Supplementary Table S11. Probe design for MLPA. 264

Supplementary Table S12. The sequence of specific primers for *L3MBTL4*, *LTBP1* and *GAPDH* in quantitative real-time PCR. 268

Supplementary Table S13. The gene specific primers for genotype identification of *L3MBTL4* transgenic rats. 270

Supplementary Table S14. The primer of q-PCR determing the enrichment of *LTBP1* peaks in immunoprecipitated DNA fragments. 271

Supplementary Table S1. Baseline Characteristics of subjects.

	stage 1		stage 2		Stage 3					
					Jidong		Shanghai		Shantou	
	case [a]	control [b]	case [c]	control [d]	case [e]	control [f]	case [e]	control [f]	case [e]	cont[illegible]
**N**	271	247	1994	2508	1427	2145	2796	4016	3416	31[illegible]
**Age (S.D.)**	43.4(3.06)	73.5(4.56)	46.2(4.9)	59.2(4.8)	49.5(12.4)	38.8(11.6)	57.46(10.68)	48.45(11.54)	59.8(11.9)	45.3([illegible]
**Gender (% female)**	23.9	35	41.9	42.2	36	58.5	50	66.5	71.2	76.[illegible]
**BMI, kg/m^2 (S.D.)**	26.7(3.5)	24.7(3.5)	26.3(3.9)	23.2(3.6)	25.9(2.8)	23.3(2.6)	25.4(3.4)	23.0(3.0)	25.1(3.4)	22.3([illegible]
**SBP (S.D.)**	144.2(15.57)	114.7(7.73)	170.2(17.5)	108.6(7.0)	146.0(16.7)	111.5(7.9)	142.9(14.8)	111.1(8.9)	149.4(19.2)	109.9[illegible]
**DBP (S.D.)**	98.32(8.05)	71.6(6.67)	105.1(10.7)	69.8(5.7)	94.6(10.6)	70.6(6.4)	90.3(8.6)	72.3(6.0)	91.7(10.8)	71.3([illegible]

N, sample size; S.D., standard deviation; BMI, body mass index; SBP, systolic blood pressure; DBP, diastolic blood pressure.

stage 1, the genome-wide association study scan; stage 2, the follow-up study; stage 3, the replication study.

[a] Case is defined as (i) SBP ≥150 mmHg and/or DBP ≥90 mmHg for untreated subjects; (ii) no secondary form of hypertension; (iii) age of onset ≤50 years.

[b] Control is defined as (i) SBP ≤125 mmHg and DBP ≤80 mmHg without antihypertensive treatments; (ii) age of onset ≥65 years.

[c] Case is defined as (i) SBP ≥150 mmHg and/or DBP ≥90 mmHg for untreated subjects; (ii) no secondary form of hypertension; (iii) age of onset ≤50 years.

[d] Control is defined as (i) SBP ≤125 mmHg and DBP ≤80 mmHg without antihypertensive treatments; (ii) age of onset ≥55 years.

[e] Case is defined as (i) SBP ≥150 mmHg and/or DBP ≥90 mmHg for untreated subjects or subjects had a history of hypertension; (ii) no secondary form of hypertension.

[f] Control is defined as (i) SBP ≤125 mmHg and DBP ≤80 mmHg without antihypertensive treatments.

Supplementary Table S2. SNPs genotyped in the follow-up study (stage 2).

select	CHR	SNP	position	Gene Symbol	Gene Location	Allele 1	Allele 2	MAF	CHISQ	OR	*P*	FDR
**GWAS**	1	rs1157688	4636767	LOC284661	INTERGENIC	A	G	0.3703	0.1426	1.017	0.7057	0.9098
**GWAS**	1	rs164771	4653765	LOC284661	INTERGENIC	A	G	0.5	0.6441	0.7241	0.4222	
**GWAS**	1	rs537761	4662408	LOC284661	INTERGENIC	A	C	0.2283	0.9783	1.051	0.3226	0.8326
**GWAS**	1	rs164781	4665232	LOC284661	INTERGENIC	A	G	0.3341	0.6116	1.036	0.4342	0.8811
**GWAS**	1	kgp12526299	8777141	CAMSAP1L1	UTR	G	A	0.2122	2.935	1.093	0.08669	0.7185
**GWAS**	1	kgp8989849	11703502	NFIA	INTERGENIC	A	G	0.3564	2.203	1.068	0.1378	0.7742
**GWAS**	1	kgp12465996	19752305	PLA2G4A	INTERGENIC	G	A	0.2808	1.595	0.942	0.2067	0.7742
**GWAS**	1	rs12116935	36789546	C1orf113	COMPLEX	G	A	0.06956	0.07343	0.9776	0.7864	0.9098
**GWAS**	1	kgp729617	40635333	GPR161	INTRON	A	G	0.3724	0.4822	0.97	0.4874	0.8811
**GWAS**	1	kgp7705709	45641485	NGF	INTRON	G	A	0.07297	0.006725	1.007	0.9346	0.9465
**GWAS**	1	kgp2589023	46708460	PDZK1IP1	INTRON	A	G	0.3782	0.145	0.9835	0.7034	0.9098
**GWAS**	1	kgp6411634	49652399	RYR2	INTRON	A	G	0.4077	0.02497	1.007	0.8744	0.9974
**GWAS**	1	kgp10225862	49678311	NFASC	INTRON	G	A	0.212	0.3204	0.971	0.5714	0.9098
**GWAS**	1	rs1047635	53074532	GPX7	UTR	C	A	0.2749	0.9353	1.047	0.3335	0.8187
**GWAS**	1	kgp12438615	53623543	CAMSAP1L1	INTRON	A	G	0.2118	2.778	1.09	0.09555	0.7185
**GWAS**	1	rs12039843	53874063	LRP8	INTERGENIC	A	G	0.4606	2.32	0.9372	0.1277	0.7185
**GWAS**	1	rs11206154	53877199	LRP8	INTERGENIC	A	C	0.4572	2.035	0.941	0.1537	0.745
**GWAS**	1	rs2994570	56801686	LOC100288320	INTERGENIC	A	G	0.1365	1.715	0.922	0.1904	0.7742
**GWAS**	1	kgp3394712	61726393	LRP8	INTERGENIC	G	A	0.4901	3.4	1.081	0.06521	0.6036
**GWAS**	1	rs1890265	61998177	NFIA	INTERGENIC	C	A	0.3561	2.247	1.069	0.1339	0.7742
**GWAS**	1	rs1933299	62000281	NFIA	INTERGENIC	C	A	0.3777	1.108	1.047	0.2926	0.82
**GWAS**	1	rs1933314	62022914	NFIA	INTERGENIC	G	A	0.3854	0.6055	1.034	0.4365	0.8811
**GWAS**	1	rs4915899	63844736	ALG6	INTRON	G	A	0.1667	0.2813	0.9702	0.5958	0.8999
**GWAS**	1	rs10493345	63900901	ALG6	INTRON	A	G	0.1619	0.4366	0.9626	0.5088	0.8999
**GWAS**	1	rs2036867	63906880	ITGB3BP	INTRON	A	C	0.1624	0.5205	0.9593	0.4706	0.8815

**GWAS**	1	rs6671446	63908980	ITGB3BP	INTRON	G	A	0.1626	0.3694	0.9656	0.5433	0.9046
**GWAS**	1	rs1057815	63918388	ITGB3BP	INTRON	G	A	0.1614	0.4413	0.9624	0.5065	0.8916
**GWAS**	1	rs6657480	63999868	EFCAB7	CODING	G	A	0.168	0.5431	0.959	0.4612	0.8811
**GWAS**	1	rs2273367	64021096	EFCAB7	CODING	A	G	0.1625	0.5875	0.9568	0.4434	0.8811
**GWAS**	1	rs4649932	71348176	PTGER3	INTRON	A	G	0.3401	0.05673	1.011	0.8117	0.9419
**GWAS**	1	kgp8706079	72603384	RGL1	INTRON	A	C	0.2166	0.1185	0.9824	0.7307	0.9419
**GWAS**	1	rs483532	91980447	CDC7	CODING	A	G	0.2631	1.449	1.06	0.2286	0.7742
**GWAS**	1	rs12082544	115839595	NGF	INTRON	C	A	0.07333	1.985	0.891	0.1589	0.7742
**GWAS**	1	rs12058927	115846221	NGF	INTRON	A	G	0.2403	0.07495	1.014	0.7843	0.9098
**GWAS**	1	rs12749808	158065762	KIRREL	UTR	A	G	0.4964	4.293	0.9158	0.007828	0.1592
**GWAS**	1	rs4540637	168103545	GPR161	INTRON	A	G	0.4218	2.779	1.074	0.09552	0.745
**GWAS**	1	rs2902570	168118292	GPR161	INTERGENIC	A	G	0.3495	0.04261	1.009	0.8365	0.9392
**GWAS**	1	rs1543358	168131592	GPR161	INTERGENIC	G	A	0.3784	0.03842	0.9915	0.8446	0.9419
**GWAS**	1	rs4657739	168142489	GPR161	INTERGENIC	A	G	0.44	1.256	1.049	0.2624	0.7901
**GWAS**	1	rs2281016	168147892	GPR161	INTERGENIC	A	G	0.4393	1.125	1.046	0.2888	0.7901
**GWAS**	1	rs10918838	168148829	TIPRL	INTRON	A	G	0.4403	1.043	1.045	0.3072	0.7902
**GWAS**	1	rs4657741	168149142	TIPRL	INTRON	A	G	0.4592	0.6259	1.034	0.4289	0.8376
**GWAS**	1	rs2235207	168159262	TIPRL	INTRON	A	G	0.4425	0.6523	1.035	0.4193	0.8524
**GWAS**	1	rs10753766	168166616	TIPRL	INTRON	A	G	0.4606	0.5752	1.033	0.4482	0.8612
**GWAS**	1	rs4534357	168167139	TIPRL	INTRON	G	A	0.4429	0.8811	1.042	0.3479	0.8387
**GWAS**	1	rs6703330	187604765	PLA2G4A	INTERGENIC	A	G	0.3101	1.727	0.9414	0.1887	0.7742
**GWAS**	1	rs955835	187609850	PLA2G4A	INTERGENIC	G	A	0.3154	3.362	0.9195	0.06671	0.6107
**GWAS**	1	rs4498762	187689619	PLA2G4A	INTERGENIC	A	G	0.2664	2.557	0.9259	0.1098	0.7399
**GWAS**	1	rs4233131	187693731	PLA2G4A	INTERGENIC	A	C	0.2988	1.42	0.9462	0.2335	0.7742
**GWAS**	1	rs10798137	187699152	PLA2G4A	INTERGENIC	A	G	0.2438	3.378	0.9129	0.06606	0.6107
**GWAS**	1	rs6661294	187700076	PLA2G4A	INTERGENIC	C	A	0.2741	3.798	0.9112	0.05131	0.5958
**GWAS**	1	rs2595961	204913652	NFASC	INTRON	G	A	0.4206	0.01007	0.9957	0.9201	0.9974
**GWAS**	1	rs2278795	204954993	NFASC	INTRON	A	G	0.4545	0.01389	0.9503	0.9062	

**GWAS**	1	rs11240325	204955329	NFASC	INTRON	G	A	0.2585	0.01045	1.005	0.9186	0.9419
**GWAS**	1	rs9660802	227017031	ITPKB	INTERGENIC	G	A	0.3148	0.2759	0.9763	0.5994	0.8811
**GWAS**	1	rs2485564	237280629	RYR2	INTRON	A	G	0.4094	0.03768	1.008	0.8461	0.9974
**GWAS**	1	rs2820037	239436542	LOC102724174	INTERGENIC	A	T	0.08265	0.2559	1.04	0.613	0.8811
**GWAS**	2	kgp8845185	24644383	NT5C1B	INTERGENIC	G	A	0.3008	0.3344	1.027	0.5631	0.8811
**GWAS**	2	kgp6315043	42799491	MLPH	INTERGENIC	G	A	0.4576	0.9789	1.043	0.3225	0.82
**GWAS**	2	kgp1666873	46735355	LOC100132413	INTRON	A	C	0.358	0.6558	1.036	0.418	0.8552
**GWAS**	2	kgp10266633	48739988	MLPH	INTERGENIC	A	C	0.3735	0.9184	1.043	0.3379	0.8376
**GWAS**	2	rs12616608	51821908	LOC730100	INTERGENIC	G	A	0.278	2.431	0.9244	0.119	
**GWAS**	2	rs2953300	51852373	LOC730100	INTERGENIC	A	G	0.256	3.052	0.9184	0.08066	0.6107
**GWAS**	2	kgp7978797	54604349	TPO	INTRON	A	G	0.4294	1.585	1.055	0.2081	0.7742
**GWAS**	2	rs4852856	72353138	DYSF	INTERGENIC	A	G	0.3858	0.3985	0.9728	0.5279	0.902
**GWAS**	2	rs2041666	72353573	DYSF	INTERGENIC	A	C	0.3699	0.3788	0.9733	0.5382	0.8999
**GWAS**	2	rs1046139	73300661	RAB11FIP5	UTR	C	A	0.2603	0.2328	0.9769	0.6294	0.8999
**GWAS**	2	rs4848495	118928678	INSIG2	INTERGENIC	A	C	0.08313	0.1121	0.9746	0.7378	0.9446
**GWAS**	2	rs4088767	118930311	INSIG2	INTERGENIC	A	G	0.08343	0.000521	1.002	0.9818	0.9876
**GWAS**	2	rs3849332	118934355	INSIG2	INTERGENIC	A	G	0.08232	0.03077	0.9865	0.8607	0.9729
**GWAS**	2	rs10186364	119006422	INSIG2	INTERGENIC	A	G	0.1669	0.003464	0.9967	0.9531	0.9954
**GWAS**	2	rs12711901	119007927	INSIG2	INTERGENIC	G	A	0.1658	0.1023	0.9819	0.7491	0.9227
**GWAS**	2	rs13005524	119010201	INSIG2	INTERGENIC	A	C	0.169	0.000833	1.002	0.977	0.9974
**GWAS**	2	rs13002573	164915208	FIGN	INTERGENIC	G	A	0.3672	0.825	0.9608	0.3637	0.8772
**GWAS**	2	rs13008224	192989148	TMEFF2	INTRON	A	G	0.1924	3.513	0.9036	0.06089	0.6107
**GWAS**	2	rs6434538	192991640	TMEFF2	INTRON	A	G	0.2449	2.845	0.9198	0.09167	0.6107
**GWAS**	2	rs2325814	238462725	MLPH	UTR	A	G	0.3763	0.7815	1.039	0.3767	0.8552
**GWAS**	2	rs1320203	238467914	MLPH	INTERGENIC	G	A	0.3739	0.6379	1.036	0.4245	0.8811
**GWAS**	3	kgp2392502	22644501	CDCP1	INTRON	C	A	0.3904	0.3757	0.9737	0.5399	0.8999
**GWAS**	3	rs1349265	24159387	THRB	UTR	A	G	0.2996	0.9202	1.045	0.3374	0.8326
**GWAS**	3	rs11716163	31807576	OSBPL10	INTRON	G	A	0.4177	0.7619	0.9631	0.3827	0.8811

**GWAS**	3	kgp11008091	43709304	C3orf70	INTERGENIC	A	G	0.39	0.6352	0.9659	0.4254	0.881
**GWAS**	3	rs4682769	45170095	CDCP1	INTRON	C	A	0.3886	0.4736	0.9705	0.4913	0.881
**GWAS**	3	rs4683055	45171167	CDCP1	INTRON	G	A	0.3934	0.5266	0.969	0.468	0.881
**GWAS**	3	kgp2541350	48661162	SERPINI1	INTRON	A	G	0.06064	1.17	1.1	0.2794	0.802
**GWAS**	3	kgp3755293	57714416	ZMAT3	INTERGENIC	A	G	0.0547	0.06947	0.9756	0.7921	0.957
**GWAS**	3	kgp11592138	66806449	TFRC	INTERGENIC	G	A	0.2425	0.2031	0.9779	0.6522	0.881
**GWAS**	3	kgp8440441	71725482	TFRC	INTERGENIC	A	G	0.1676	0.151	0.9781	0.6976	0.909
**GWAS**	3	rs6804537	184897801	C3orf70	INTERGENIC	A	G	0.3076	0.002483	1.002	0.9603	0.995
**GWAS**	3	rs721814	184925384	EHHADH	INTRON	A	G	0.4916	0.0391	1.008	0.8432	0.997
**GWAS**	3	rs2269228	184930238	EHHADH	INTRON	A	G	0.2884	0.1174	0.9841	0.7318	0.921
**GWAS**	3	rs6779662	184940498	EHHADH	INTRON	G	A	0.294	0.3832	0.9716	0.5359	0.881
**GWAS**	3	rs12490036	195816195	TFRC	INTERGENIC	A	G	0.1996	3.184	0.9093	0.07437	0.610
**GWAS**	4	rs4689519	4425620	STX18	INTRON	A	G	0.2062	0.01213	0.9942	0.9123	0.995
**GWAS**	4	rs2369705	5436590	STK32B	INTRON	A	G	0.4387	1.008	1.044	0.3154	0.818
**GWAS**	4	rs3733182	5448429	STK32B	CODING	G	A	0.4912	1.413	1.052	0.2346	0.790
**GWAS**	4	rs3733180	5450124	STK32B	INTRON	A	G	0.4899	1.444	1.052	0.2295	0.790
**GWAS**	4	kgp1200023	19697331	ABLIM2	INTRON	T	A	0.2255	0.04408	1.011	0.8337	0.954
**GWAS**	4	kgp4225342	24673305	GPRIN3	INTERGENIC	G	A	0.436	0.5793	0.9679	0.4466	0.881
GWAS	4	kgp10702591	27809358	ABLIM2	INTRON	C	G	0.1745	0.3331	0.9673	0.5639	
**GWAS**	4	kgp4832055	45663431	GPRIN3	INTERGENIC	C	A	0.4376	0.4347	0.9722	0.5097	0.881
**GWAS**	4	kgp7680058	71702479	STK32B	INTRON	G	A	0.4889	1.152	1.047	0.2831	0.818
**GWAS**	4	rs4694683	75243702	EREG	INTRON	A	G	0.3206	0.3111	1.026	0.577	0.899
**GWAS**	4	rs2367707	75248434	EREG	CODING	A	G	0.3163	0.0174	1.006	0.895	0.972
**GWAS**	4	rs2367708	75248544	EREG	INTRON	G	A	0.3277	0.000442	0.999	0.9832	0.997
GWAS	4	rs1017733	75252350	EREG	UTR	A	G	0.375	0.6095	1.357	0.435	
**GWAS**	4	rs1460013	75258429	EREG	INTERGENIC	G	A	0.2883	0.1273	0.9834	0.7212	0.909
**GWAS**	4	rs2703137	77854979	ANKRD56	INTERGENIC	C	A	0.153	0.08954	0.9825	0.7648	0.941
**GWAS**	4	rs4106153	90244476	GPRIN3	INTERGENIC	C	A	0.437	0.5865	0.9678	0.4438	0.875

**GWAS**	4	rs6854616	183761427	ODZ3	INTERGENIC	A	G	0.4607	0.09441	0.987	0.7586	0.9465
**GWAS**	5	rs3797937	9191362	SEMA5A	INTRON	G	A	0.4303	2.818	0.9305	0.09321	0.7185
**GWAS**	5	rs1806125	9192881	SEMA5A	INTRON	C	A	0.3189	1.638	1.06	0.2006	0.7742
**GWAS**	5	rs32270	11197025	CTNND2	INTRON	A	C	0.2996	0.6936	0.9621	0.4049	0.8811
**GWAS**	5	rs27720	11201861	CTNND2	INTRON	G	A	0.2583	0.3113	1.027	0.5769	0.8811
**GWAS**	5	kgp10335597	19617378	ODZ2	INTRON	G	A	0.4366	1.178	1.048	0.2778	0.7902
**GWAS**	5	kgp9981262	22651339	ODZ2	INTRON	A	G	0.4183	0.2004	0.9809	0.6544	0.8999
**GWAS**	5	kgp5310817	32808375	STC2	INTERGENIC	G	A	0.1669	1.314	1.067	0.2517	0.7893
**GWAS**	5	kgp4131619	44734401	STC2	INTERGENIC	A	G	0.1132	0.7086	1.058	0.3999	0.8524
**GWAS**	5	kgp1843608	64688429	ODZ2	INTRON	T	A	0.4852	0.1438	1.016	0.7045	0.9028
**GWAS**	5	kgp1616247	68625452	ODZ2	INTRON	G	A	0.2705	0.3721	1.03	0.5418	0.8811
**GWAS**	5	kgp11234410	90777318	SEMA5A	INTRON	G	A	0.4113	2.503	1.071	0.1137	0.751
**GWAS**	5	rs4307107	162841338	GABRG2	INTERGENIC	G	A	0.08454	0.1532	0.9705	0.6955	0.9335
**GWAS**	5	rs7713448	167146649	ODZ2	INTRON	C	A	0.4804	1.128	1.046	0.2882	0.8025
**GWAS**	5	rs10057680	167147874	ODZ2	INTRON	G	A	0.2912	0.1616	1.019	0.6877	0.9046
**GWAS**	6	kgp12425976	16621386	SLC16A10	INTRON	C	A	0.07316	0.03148	0.9856	0.8592	0.9465
**GWAS**	6	rs6921343	25311461	LRRC16A	INTRON	A	G	0.3724	0.5441	1.033	0.4607	0.8612
**GWAS**	6	rs9295654	25312755	LRRC16A	INTRON	A	G	0.372	0.5144	1.032	0.4733	0.8772
**GWAS**	6	rs9467466	25312915	LRRC16A	INTRON	A	G	0.373	0.6572	1.036	0.4175	0.8326
**GWAS**	6	rs2744303	25327149	LRRC16A	INTRON	C	A	0.3847	0.01081	0.9955	0.9172	0.9592
**GWAS**	6	rs10456044	25367710	LRRC16A	INTRON	A	C	0.3607	1.302	0.9508	0.2539	0.7901
**GWAS**	6	rs215012	25383814	LRRC16A	INTRON	G	A	0.334	1.512	0.9461	0.2188	0.7742
**GWAS**	6	rs9461924	33854816	MLN	INTERGENIC	A	C	0.2438	0.3939	0.9694	0.5303	0.8999
**GWAS**	6	rs1776888	34158331	GRM4	INTERGENIC	A	C	0.2964	1.532	1.059	0.2159	0.7742
**GWAS**	6	kgp8818949	36780434	MLN	INTERGENIC	A	G	0.2751	1.656	1.063	0.1981	0.7742
**GWAS**	6	rs2076472	41029342	APOBEC2	CODING	G	A	0.2349	1.626	0.938	0.2022	0.7742
**GWAS**	6	rs9394761	41033640	APOBEC2	INTERGENIC	C	A	0.2356	1.516	0.9402	0.2182	0.7893
**GWAS**	6	rs11280	41034858	C6orf130	UTR	G	A	0.2461	1.151	0.9484	0.2833	0.8134

**GWAS**	6	rs9381034	41049508	NFYA	INTRON	G	A	0.2405	0.5636	0.9634	0.4528	0.8811
**GWAS**	6	rs943084	43770150	VEGFA	INTERGENIC	A	G	0.2524	0.001337	0.9982	0.9708	0.9974
**GWAS**	6	kgp187992	59662436	MLN	INTERGENIC	C	G	0.3449	0.004995	1.003	0.9437	0.9592
**GWAS**	6	rs12525751	109337723	LOC100287407	INTERGENIC	A	G	0.08831	0.05095	0.9832	0.8214	0.9419
**GWAS**	7	kgp11041212	18679501	SEMA3C	INTERGENIC	G	A	0.3959	0.1174	0.9852	0.7319	0.9098
GWAS	7	rs2215934	78112731	MAGI2	INTRON	A	G	0.463	0.09124	1.124	0.7626	
**GWAS**	7	rs4726937	147954909	CNTNAP2	INTRON	A	G	0.07138	1.824	0.894	0.1768	0.7742
**GWAS**	8	rs2959592	17002725	EFHA2	INTERGENIC	A	G	0.3732	0.0829	1.013	0.7734	0.9098
**GWAS**	8	kgp2914505	21707531	OXR1	INTRON	A	G	0.2333	0.1392	1.019	0.7091	0.9046
**GWAS**	8	kgp463300	37742338	EFHA2	INTERGENIC	G	A	0.289	0.1387	0.9827	0.7096	0.9227
**GWAS**	8	kgp280249	45607306	SLC18A1	INTRON	C	G	0.4191	3.202	0.9259	0.07356	0.6107
**GWAS**	8	kgp9407592	53688427	FAM84B	INTERGENIC	A	G	0.1292	0.1014	1.02	0.7502	0.9227
**GWAS**	8	kgp4935987	60746457	FAM84B	INTERGENIC	G	A	0.3434	1.967	1.065	0.1608	0.7742
**GWAS**	8	kgp565395	64713338	LOC157381	UTR	C	A	0.1771	0.004173	0.9964	0.9485	0.997
**GWAS**	8	rs12679456	90827701	RIPK2	INTERGENIC	C	A	0.1883	0.07499	0.9852	0.7842	0.9465
**GWAS**	8	rs40638	90828052	RIPK2	INTERGENIC	G	A	0.4927	1.705	0.9461	0.1917	0.7742
**GWAS**	8	rs1621663	107462505	OXR1	INTRON	G	A	0.2326	0.1433	1.019	0.705	0.9097
**GWAS**	8	rs1670387	107474139	OXR1	INTRON	G	A	0.2354	0.1414	1.019	0.7069	0.9098
**GWAS**	8	rs7817974	107477037	OXR1	INTRON	A	G	0.3516	2.133	1.067	0.1442	0.7742
GWAS	8	rs6469756	119556430	SAMD12	INTRON	A	G	0.4179	1.218	0.9473	0.2698	
**GWAS**	8	rs4870982	127740139	FAM84B	INTERGENIC	A	G	0.4408	0.4682	0.9712	0.4938	0.8811
**GWAS**	8	rs2029424	127746957	FAM84B	INTERGENIC	G	A	0.448	0.5655	0.9684	0.452	0.8811
**GWAS**	8	rs4871739	127768683	FAM84B	INTERGENIC	G	A	0.3944	1.627	0.9461	0.2021	0.7742
**GWAS**	9	rs2183472	4487374	LOC100287493	INTERGENIC	G	A	0.1409	1.609	0.9247	0.2047	0.7742
**GWAS**	9	rs10757288	22159416	LOC729983	INTERGENIC	G	A	0.09344	0.001496	1.003	0.9691	0.9592
**GWAS**	9	rs1411373	27746785	C9orf72	INTERGENIC	A	G	0.1625	0.3081	0.9685	0.5789	0.8811
**GWAS**	9	kgp18585905	48622205	FRMPD1	CODING	A	G	0.3617	2.907	0.9274	0.08821	0.7185
**GWAS**	9	kgp7431060	60674415	GNAQ	INTRON	A	G	0.2022	0.9597	0.9495	0.3273	0.82

**GWAS**	9	kgp9568150	66749500	C9orf98	INTRON	A	G	0.1547	11.84	0.8154	0.000579	0.0376
**GWAS**	9	rs7043420	79189090	LOC392352	INTERGENIC	A	G	0.09429	0.6338	1.059	0.426	0.8811
**GWAS**	9	rs9409449	94412540	LOC100132701	INTERGENIC	A	C	0.1213	3.9	1.136	0.04829	0.5329
**GWAS**	9	rs10821278	96740079	BARX1	INTERGENIC	A	C	0.4473	0.00195	1.002	0.9648	0.9954
**GWAS**	9	rs10761312	96769237	BARX1	INTERGENIC	A	G	0.449	0.0585	0.9897	0.8089	0.9465
**GWAS**	9	rs4149274	107639414	ABCA1	INTRON	A	G	0.2886	0.239	0.9773	0.6249	0.9098
**GWAS**	9	rs10901212	135647021	C9orf98	INTRON	C	A	0.1206	0.04607	0.9861	0.83	0.9766
**GWAS**	10	rs11818932	12288366	CDC123	INTRON	A	C	0.2239	2.117	1.077	0.1457	0.7624
**GWAS**	10	kgp10363410	12796400	KCNMA1	INTRON	A	G	0.01771	1.317	1.202	0.2512	0.7742
**GWAS**	10	kgp3103529	13766326	FAM53B	INTRON	A	G	0.152	0.4195	1.039	0.5172	0.8811
**GWAS**	10	kgp2928342	17803477	TMEM26	INTRON	A	G	0.1913	1.518	1.069	0.2179	0.7742
**GWAS**	10	rs4354609	24316134	KIAA1217	INTRON	G	A	0.4483	2.104	1.064	0.1469	0.7742
**GWAS**	10	rs7912789	24691079	KIAA1217	INTRON	G	A	0.3103	0.263	1.024	0.608	0.9098
**GWAS**	10	kgp5400193	24718378	KCNMA1	INTRON	A	G	0.1767	0.01011	1.006	0.9199	0.9974
**GWAS**	10	kgp12449774	29720388	SEC61A2	INTRON	A	G	0.1145	0.3317	0.9623	0.5646	0.8999
**GWAS**	10	kgp6245100	58712343	TMEM26	INTRON	G	A	0.1965	1.129	1.058	0.2879	0.7901
**GWAS**	10	rs1907217	63172966	TMEM26	INTRON	A	G	0.1968	0.8212	1.049	0.3648	0.8187
**GWAS**	10	rs12572590	63186686	TMEM26	INTRON	A	G	0.1951	1.122	1.058	0.2895	0.7902
**GWAS**	10	rs4948281	63248358	TMEM26	INTERGENIC	A	G	0.1935	1.091	1.058	0.2964	0.7901
**GWAS**	10	rs4454676	63355099	TMEM26	INTERGENIC	A	G	0.1064	0.1741	1.029	0.6765	0.9419
**GWAS**	10	rs16918582	65304328	REEP3	INTRON	C	A	0.05553	3.278	1.181	0.07022	0.6107
**GWAS**	10	kgp21672435	66727240	LRRC20	INTRON	A	G	0.1174	0.1204	0.9774	0.7286	0.9227
**GWAS**	10	rs2579787	78063891	C10orf11	INTRON	G	A	0.4705	1.92	0.9428	0.1659	0.7742
**GWAS**	10	rs10824393	78072371	C10orf11	INTRON	A	C	0.3064	1.174	0.9513	0.2786	0.7901
**GWAS**	10	rs10824403	78096526	C10orf11	INTRON	G	A	0.3126	2.208	0.9341	0.1373	0.745
**GWAS**	10	rs11001742	78107150	C10orf11	INTRON	G	A	0.3107	1.947	0.9379	0.1629	0.7742
**GWAS**	10	rs11002139	79129757	KCNMA1	INTRON	A	G	0.1626	0.3273	0.9676	0.5673	0.8811
**GWAS**	10	rs10824534	79137838	KCNMA1	INTRON	G	A	0.1668	0.4104	0.9641	0.5218	0.8811

**GWAS**	10	rs42455	79162342	KCNMA1	INTRON	G	A	0.2269	0.6104	1.04	0.4346	0.8811
**GWAS**	10	rs673195	79164857	KCNMA1	INTRON	G	A	0.1949	0.9299	1.053	0.3349	0.8326
**GWAS**	10	rs17112147	98551647	RPL13AP5	INTERGENIC	A	G	0.07021	1.349	0.9076	0.2455	0.7764
**GWAS**	10	rs11190245	101451259	ENTPD7	CODING	G	A	0.3011	0.05717	1.011	0.811	0.9419
**GWAS**	10	rs767844	101500472	CUTC	INTRON	G	A	0.3309	0.04239	1.009	0.8369	0.9227
**GWAS**	10	rs4148399	101592689	ABCC2	INTRON	C	A	0.03199	0.7022	1.115	0.402	
**GWAS**	10	rs3758395	101602004	ABCC2	INTRON	G	A	0.3509	0.01213	0.9951	0.9123	0.9974
**GWAS**	10	rs3740065	101605693	ABCC2	INTRON	G	A	0.3531	0.007292	1.004	0.9319	0.957
**GWAS**	10	rs3750821	116055417	AFAP1L2	UTR	G	A	0.3051	1.048	1.048	0.3059	0.8187
**GWAS**	10	rs11597613	126383857	FAM53B	INTRON	A	G	0.1933	1.173	1.06	0.2787	0.7901
**GWAS**	11	rs10741735	2920283	SLC22A18AS	INTRON	A	G	0.397	0.6494	1.036	0.4203	0.8376
**GWAS**	11	rs7924382	11227291	ZBED5	INTERGENIC	G	A	0.051	0.2488	0.9527	0.6179	0.9046
**GWAS**	11	rs12421938	11422750	GALNTL4	INTRON	G	A	0.1166	9.27	0.8158	0.00233	0.0659
**GWAS**	11	rs7935882	11427577	GALNTL4	INTRON	G	A	0.1399	6.005	0.8599	0.01426	0.346
**GWAS**	11	kgp8692495	21777303	METT5D1	INTERGENIC	A	G	0.0972	1.978	0.9037	0.1596	0.7742
**GWAS**	11	kgp1029052	31661350	LOC100288816	INTERGENIC	G	A	0.1871	0.1474	1.021	0.701	0.9098
**GWAS**	11	kgp916075	34632421	SCN4B	INTRON	A	G	0.1475	0.003767	0.9963	0.9511	0.9465
**GWAS**	11	rs7940927	36785787	C11orf74	INTERGENIC	A	G	0.4175	1.727	1.058	0.1889	0.7742
**GWAS**	11	rs428215	37126189	C11orf74	INTERGENIC	G	A	0.2911	1.957	0.9366	0.1618	0.7742
**GWAS**	11	kgp2718916	42606489	METT5D1	INTERGENIC	A	G	0.09682	0.9169	0.9333	0.3383	0.8376
**GWAS**	11	kgp2009561	48655304	ZBED5	INTERGENIC	G	A	0.2464	0.01314	0.994	0.9087	0.9592
**GWAS**	11	kgp10671407	48682413	ZBED5	INTERGENIC	G	A	0.2507	0.2473	0.9759	0.6189	0.8999
**GWAS**	11	kgp11213976	52717500	LOC100288816	INTERGENIC	A	G	0.201	0.2069	1.024	0.6492	0.9098
**GWAS**	11	kgp214437	58647447	TSPAN32	INTRON	A	G	0.2145	2.563	0.9203	0.1094	0.7742
**GWAS**	11	kgp7665589	60701495	C11orf74	INTERGENIC	A	G	0.4091	1.823	1.06	0.177	0.7742
**GWAS**	11	rs3017670	62744899	SLC22A6	INTRON	A	G	0.2056	0.2624	0.9734	0.6085	0.8811
**GWAS**	11	rs4944336	81466717	LOC100288816	INTERGENIC	A	C	0.4937	2.568	0.9342	0.1091	0.745
**GWAS**	11	rs17142932	81490264	LOC100288816	INTERGENIC	A	G	0.4168	1.433	1.053	0.2313	0.7901

**GWAS**	11	rs6591999	81492541	LOC100288816	INTERGENIC	A	G	0.2091	0.3288	1.03	0.5663	0.8811
**GWAS**	11	rs7104475	81492649	LOC100288816	INTERGENIC	A	G	0.1785	0.9016	1.056	0.3424	
**GWAS**	11	rs7948962	81494943	LOC100288816	INTERGENIC	C	A	0.2087	0.3655	1.032	0.5455	0.8811
**GWAS**	11	rs7950775	81498152	LOC100288816	INTERGENIC	G	A	0.1832	0.1849	1.024	0.6672	0.9097
**GWAS**	11	rs1459937	81525713	LOC100288816	INTERGENIC	A	G	0.1862	0.2259	1.026	0.6346	0.8999
**GWAS**	11	rs4944360	81531111	LOC100288816	INTERGENIC	A	G	0.186	0.06305	1.014	0.8017	0.9419
**GWAS**	11	rs10897889	81532550	LOC100288816	INTERGENIC	C	A	0.4309	1.603	1.056	0.2054	0.7893
**GWAS**	11	rs1013273	81534503	LOC100288816	INTERGENIC	G	A	0.1788	0.2433	1.028	0.6219	0.8999
**GWAS**	11	rs7934819	81538168	LOC100288816	INTERGENIC	A	G	0.2263	0.4094	0.968	0.5223	0.8811
**GWAS**	11	rs12577924	81539154	LOC100288816	INTERGENIC	G	A	0.1815	0.2609	1.028	0.6095	0.8999
**GWAS**	11	rs7121014	81540109	LOC100288816	INTERGENIC	A	C	0.2214	0.3348	1.03	0.5628	0.8835
**GWAS**	11	rs10437580	95862546	MAML2	INTRON	G	A	0.2467	0.2089	1.023	0.6476	0.9046
**GWAS**	11	rs10790778	99406350	CNTN5	INTRON	A	G	0.1266	0.9064	0.9409	0.3411	0.8772
**GWAS**	11	rs7925532	99416115	CNTN5	INTRON	G	A	0.1374	1.222	0.9339	0.269	0.8134
**GWAS**	11	rs643281	102386076	LOC727869	INTERGENIC	A	G	0.005414	0.2363	1.151	0.6269	0.8811
**GWAS**	11	rs2510087	102948592	DCUN1D5	INTRON	A	G	0.04926	1.257	0.8947	0.2622	0.8326
**GWAS**	11	rs2513996	102949969	DCUN1D5	INTRON	G	A	0.2444	1.112	0.9492	0.2917	0.82
**GWAS**	11	rs1893073	102968093	DCUN1D5	INTERGENIC	C	A	0.2443	0.9455	0.953	0.3309	0.8326
**GWAS**	12	rs2239083	2573043	CACNA1C	INTRON	G	A	0.1314	0.04227	1.013	0.8371	0.9465
**GWAS**	12	rs2239084	2577104	CACNA1C	INTRON	A	G	0.1266	0.000358	1.001	0.9849	0.9974
**GWAS**	12	kgp5769230	16617334	TMEM132C	INTRON	C	A	0.1127	0.0947	1.021	0.7583	0.9227
**GWAS**	12	kgp11206447	26635466	RIMBP2	INTRON	A	G	0.3475	0.4621	1.031	0.4966	0.8811
**GWAS**	12	kgp18883701	52738539	TMEM132C	INTRON	A	G	0.1068	0.2256	1.033	0.6348	0.8916
**GWAS**	12	rs1983313	130963148	RIMBP2	INTRON	A	G	0.3316	0.8231	1.042	0.3643	0.8376
**GWAS**	13	kgp7580276	26706488	DACH1	INTERGENIC	G	A	0.09129	1.233	0.9211	0.2668	0.7893
**GWAS**	13	kgp12014499	33806384	ENOX1	INTRON	A	G	0.333	0.1521	1.018	0.6965	0.9227
**GWAS**	13	rs9603532	39983072	LHFP	INTRON	A	C	0.07533	0.2116	1.038	0.6455	0.902
**GWAS**	13	rs9315683	39984967	LHFP	INTRON	G	A	0.07252	1.092	1.089	0.2959	0.8187

**GWAS**	13	rs2325033	43929302	ENOX1	INTRON	A	G	0.4899	0.4433	0.9721	0.5056	0.881
**GWAS**	13	rs9567161	43932798	ENOX1	INTRON	A	G	0.1535	0.1581	1.024	0.6909	0.917
**GWAS**	13	rs9525762	43943415	ENOX1	INTRON	G	A	0.4605	0.6312	0.9667	0.4269	0.881
**GWAS**	13	kgp16823924	44730181	LOC647264	INTERGENIC	A	C	0.0569	1.69	1.126	0.1936	0.774
**GWAS**	13	rs17088537	65156017	LOC647264	INTERGENIC	G	A	0.0896	0.2805	0.9613	0.5964	0.911
**GWAS**	13	rs12428541	65158529	LOC647264	INTERGENIC	G	A	0.09076	0.1332	0.9734	0.7151	0.957
**GWAS**	13	rs12428506	65160146	LOC647264	INTERGENIC	G	A	0.09265	0.00149	1.003	0.9692	0.946
**GWAS**	13	rs634685	65178318	LOC647264	INTERGENIC	G	A	0.09007	0.007954	0.9934	0.9289	0.959
**GWAS**	13	rs9555700	111102495	COL4A2	INTRON	C	A	0.4125	0.3258	0.9757	0.5682	0.881
**GWAS**	14	kgp11331807	21693465	FOS	INTERGENIC	A	G	0.1362	0.0281	0.9897	0.8669	0.941
**GWAS**	14	kgp19469873	58698289	HECTD1	CODING	A	G	0.136	0.3277	0.9651	0.567	0.904
**GWAS**	14	rs7146378	75756803	FOS	INTERGENIC	A	G	0.1431	0.005459	0.9955	0.9411	0.957
**GWAS**	14	rs11159133	75759172	FOS	INTERGENIC	G	A	0.1409	0.008842	1.006	0.9251	0.997
**GWAS**	14	rs2430340	92337774	FBLN5	INTRON	A	G	0.08376	0.09535	0.9766	0.7575	0.941
**GWAS**	15	kgp5725677	10760388	THSD4	INTRON	A	G	0.4637	0.1198	0.9854	0.7293	0.909
**GWAS**	15	kgp6080674	19748468	KIAA1024	INTERGENIC	A	G	0.4205	0.3361	0.7782	0.5621	
**GWAS**	15	rs4906676	26681670	LOC100128714	INTERGENIC	G	A	0.1123	0.5578	1.051	0.4551	0.881
**GWAS**	15	rs8041610	27008264	GABRB3	INTRON	C	A	0.4024	0.4294	0.972	0.5123	0.881
**GWAS**	15	rs11629509	27035868	GABRB3	INTERGENIC	G	A	0.4714	0.06714	0.989	0.7955	0.933
**GWAS**	15	kgp19985034	36641846	MGA	INTRON	C	A	0.08404	0.2461	0.9626	0.6198	0.902
**GWAS**	15	kgp2367376	47626378	ADAMTS17	CODING	G	A	0.07219	0.5316	0.9418	0.4659	0.881
**GWAS**	15	kgp11460984	63772467	ADAMTS17	INTRON	C	A	0.07833	0.1324	1.029	0.716	0.909
**GWAS**	15	rs7169899	68633534	ITGA11	INTRON	A	G	0.4012	0.09221	1.013	0.7614	0.909
**GWAS**	15	rs720251	70938445	TLE3	INTERGENIC	A	G	0.0934	3.188	0.877	0.07417	0.610
**GWAS**	15	rs2114716	80454745	FAH	INTRON	G	A	0.4768	0.6117	0.9673	0.4342	0.881
**GWAS**	15	rs9806684	100701154	ADAMTS17	INTRON	A	C	0.3463	1.614	1.06	0.204	0.774
**GWAS**	16	rs7202727	12707944	SNX29	INTERGENIC	A	G	0.1089	1.023	1.071	0.3118	0.82
**GWAS**	16	rs7188223	12709531	SNX29	INTERGENIC	A	G	0.4143	0.01569	0.9408	0.9003	

**GWAS**	16	rs12708757	12710581	SNX29	INTERGENIC	A	G	0.107	1.004	1.071	0.3164	0.82
**GWAS**	16	kgp8442661	18658429	SNX29	INTERGENIC	C	A	0.1113	0.1477	1.026	0.7008	0.9098
**GWAS**	16	kgp1044218	26715441	HSD17B2	INTERGENIC	C	A	0.284	0.02761	0.9922	0.868	0.9584
**GWAS**	16	kgp1892236	29623309	WFDC1	INTRON	A	T	0.4066	0.468	0.9709	0.4939	0.8916
**GWAS**	16	kgp6079816	32746365	A2BP1	INTRON	A	G	0.4313	2.535	1.071	0.1113	0.745
**GWAS**	16	kgp2946421	44651497	SLC6A2	INTRON	A	G	0.3701	2.13	1.066	0.1445	0.6678
**GWAS**	16	kgp2397163	75777527	IRF8	INTERGENIC	A	G	0.3608	0.3841	0.973	0.5354	0.8811
**GWAS**	16	rs11648121	78181986	WWOX	INTRON	A	G	0.22	1.139	1.056	0.2859	0.8025
**GWAS**	16	rs4887941	78200278	WWOX	INTRON	G	A	0.2202	2.644	1.087	0.104	0.7185
**GWAS**	16	rs11150047	78201753	WWOX	INTRON	A	C	0.2212	3.332	1.098	0.06793	0.6107
**GWAS**	16	rs4888957	79398547	LOC729251	INTERGENIC	G	A	0.2178	6.279	0.8786	0.01222	0.2856
**GWAS**	16	rs4243170	79437209	LOC729251	INTERGENIC	A	G	0.268	8.069	0.8722	0.004502	0.1322
**GWAS**	16	rs1064208	82181977	MPHOSPH6	UTR	G	A	0.477	0.6784	1.036	0.4101	0.8811
**GWAS**	16	rs12445041	82194190	MPHOSPH6	INTRON	A	G	0.4784	1.511	1.054	0.2189	0.7901
**GWAS**	16	rs2967383	82217153	MPHOSPH6	INTERGENIC	C	A	0.1894	0.2847	0.9715	0.5936	0.8999
**GWAS**	16	rs904201	86248387	IRF8	INTERGENIC	G	A	0.3756	2.068	0.9389	0.1504	0.7742
**GWAS**	16	rs7200646	86335351	IRF8	INTERGENIC	A	G	0.3823	3.307	0.9236	0.06899	0.6107
**GWAS**	16	rs2665313	86342373	IRF8	INTERGENIC	G	A	0.4731	0.5333	1.032	0.4652	0.8631
**GWAS**	17	rs17225738	5861557	NLRP1	INTERGENIC	A	G	0.3178	7.128	0.8851	0.007591	0.1487
**GWAS**	17	rs8070912	5865080	NLRP1	INTERGENIC	G	A	0.4547	4.791	0.9109	0.02861	0.4928
**GWAS**	17	rs3786103	9859408	GAS7	INTRON	A	G	0.496	0.3562	0.975	0.5506	0.8815
**GWAS**	17	rs11078825	9861160	GAS7	INTRON	A	G	0.3629	0.6596	0.9648	0.4167	0.8707
**GWAS**	17	rs11658790	9862216	GAS7	INTRON	A	C	0.4872	0.04403	1.009	0.8338	0.9465
**GWAS**	17	kgp6576302	30679331	ABR	INTRON	T	A	0.4809	0.02607	0.9932	0.8717	0.9954
**GWAS**	17	kgp6547754	34795335	SDK2	INTRON	A	G	0.2903	0.7656	1.042	0.3816	0.8326
**GWAS**	17	rs2716213	67497563	MAP2K6	INTRON	A	C	0.4641	0.5926	1.033	0.4414	0.8811
**GWAS**	17	rs2521348	67499717	MAP2K6	INTRON	G	A	0.4608	0.6148	1.034	0.433	0.8811
**GWAS**	17	rs2251862	67513003	MAP2K6	CODING	A	C	0.4667	1.531	0.9487	0.2159	0.8134

**GWAS**	17	rs2074028	67517549	MAP2K6	INTRON	A	G	0.471	0.5027	1.031	0.4783	0.8811
**GWAS**	17	rs2251160	67519542	MAP2K6	INTRON	G	A	0.4946	0.6305	1.034	0.4272	0.8811
**GWAS**	17	rs2074031	67522975	MAP2K6	INTRON	G	A	0.4936	0.3686	0.9746	0.5438	0.8999
**GWAS**	17	rs2715833	67527626	MAP2K6	INTRON	A	G	0.472	0.4417	1.029	0.5063	0.8811
**GWAS**	17	rs10438701	71395173	SDK2	INTRON	A	G	0.2763	0.05668	0.988	0.8118	0.9465
**GWAS**	17	rs7226251	71397375	SDK2	INTRON	C	A	0.2873	0.01045	1.005	0.9186	0.9779
**GWAS**	18	rs403814	6282593	L3MBTL4	INTRON	C	A	0.2497	19.08	1.238	0.000013	0.0011
**GWAS**	18	rs2729741	6283038	L3MBTL4	INTRON	G	A	0.1961	2.999	1.097	0.0833	0.6107
**GWAS**	18	rs1452539	6287726	L3MBTL4	INTRON	G	A	0.1746	1.663	1.075	0.1972	0.7742
**GWAS**	18	rs4561576	20819090	CABLES1	INTRON	G	A	0.2002	0.09382	0.9839	0.7594	0.9419
**GWAS**	18	rs774788	49839327	LOC100287225	INTERGENIC	A	G	0.06599	1.078	0.9147	0.2991	0.82
**GWAS**	18	rs9304421	49842838	LOC100287225	INTERGENIC	A	G	0.08032	1.587	0.9058	0.2077	0.7901
**GWAS**	18	rs8084171	49853145	LOC100287225	INTERGENIC	A	G	0.2533	1.571	0.8796	0.21	
**GWAS**	18	rs1443570	49878620	DCC	INTRON	G	A	0.09244	3.486	0.8713	0.0619	0.6107
**GWAS**	18	kgp8313589	53675508	DCC	INTRON	A	G	0.07708	0.9851	0.9237	0.3209	0.8187
**GWAS**	18	rs4566279	61724709	SERPINB8	INTERGENIC	G	A	0.1004	0.7697	1.064	0.3803	0.8387
**GWAS**	19	rs17604735	1807018	ATP8B3	INTRON	G	A	0.1508	0.00032	0.9989	0.9857	0.9954
**GWAS**	19	rs7249302	1808683	ATP8B3	INTRON	A	G	0.1469	0.2312	1.029	0.6306	0.9098
**GWAS**	19	rs107251	4176085	SIRT6	INTRON	A	G	0.2783	0.2216	0.978	0.6379	0.8999
**GWAS**	19	rs12461296	33774591	SLC7A10	INTERGENIC	A	C	0.2008	2.795	0.915	0.09456	0.7185
**GWAS**	19	rs887804	40357123	FCGBP	INTRON	G	A	0.3032	1.152	0.9516	0.2831	0.82
**GWAS**	19	rs3745489	50412650	NUP62	CODING	A	G	0.1181	0.002892	0.9965	0.9571	0.9974
**GWAS**	19	rs7246004	51216909	SHANK1	INTRON	C	A	0.375	0.1868	0.9812	0.6656	0.8999
**GWAS**	19	kgp21549796	84717203	ZNF570	INTERGENIC	G	A	0.05932	0.8779	1.087	0.3488	0.8723
**GWAS**	19	kgp21368512	98772417	HKR1	CODING	G	A	0.03157	3.388	1.248	0.06567	0.7608
**GWAS**	20	rs2206698	1937475	SIRPA	INTERGENIC	A	G	0.2317	0.1284	0.9821	0.7201	0.9419
**GWAS**	20	rs12626044	1942718	SIRPA	INTERGENIC	G	A	0.4997	0.2965	0.9769	0.5861	0.8811
**GWAS**	20	rs11087269	1944510	SIRPA	INTERGENIC	A	G	0.2606	0.007618	0.9958	0.9304	0.9924

**GWAS**	20	rs6045697	1945406	SIRPA	INTERGENIC	A	G	0.2389	0.1186	0.983	0.7306	0.9403
**GWAS**	20	rs2064279	42094474	SFRS6	INTERGENIC	A	C	0.02267	1.424	0.8419	0.2328	0.815
**GWAS**	20	kgp3621958	44752372	LOC100130589	INTERGENIC	A	G	0.2124	5.274	0.8872	0.02165	0.4877
**GWAS**	20	rs3003145	62868948	MYT1	INTRON	G	A	0.08935	0.2588	1.039	0.6109	0.8811
**GWAS**	21	rs11702531	41477207	DSCAM	INTRON	G	A	0.0926	0.004435	1.005	0.9469	0.9465
**GWAS**	21	rs11701451	41481302	DSCAM	INTRON	G	A	0.09237	0.04199	1.015	0.8376	0.9098
**GWAS**	22	kgp8715947	36641486	APOBEC3H	INTRON	A	G	0.286	4.416	0.9058	0.0356	0.4928
**GWAS**	22	rs139314	39499604	APOBEC3H	INTRON	G	A	0.2801	4.038	0.9092	0.04449	0.5329
**GWAS**	22	rs138843	50183162	BRD1	INTRON	A	G	0.1288	0.6308	0.9508	0.4271	0.8811
**GWAS**	22	rs138845	50185192	BRD1	INTRON	A	G	0.1124	0.9103	0.9377	0.34	0.8811
**GWAS**	22	rs138850	50193337	BRD1	INTRON	G	A	0.3908	1.229	0.9529	0.2676	0.7902
**GWAS**	22	kgp8776661	57728345	CECR1	UTR	G	C	0.3321	1.41	1.055	0.235	0.7902
**GWAS**	23	rs1974522	3238733	MXRA5	CODING	A	G	0.3979	0.0391	1.01	0.8433	0.9465
**GWAS**	23	rs1635246	3240343	MXRA5	CODING	A	G	0.3989	0.04881	1.011	0.8251	0.9518
**GWAS**	23	rs1635248	3241284	MXRA5	CODING	G	A	0.4014	0.1601	1.02	0.689	0.9098
**GWAS**	23	rs4830872	13643346	EGFL6	INTRON	C	A	0.3792	6.04	1.129	0.01399	0.2856
**GWAS**	23	rs12835909	13647506	EGFL6	INTRON	G	A	0.4046	1.559	0.9405	0.2119	0.7742
**GWAS**	23	rs2361159	13681115	TCEANC	CODING	G	A	0.3788	4.134	1.106	0.007920	0.1629
**GWAS**	23	rs5935649	13681638	TCEANC	CODING	G	A	0.3812	4.454	1.11	0.005483	0.1329
**GWAS**	23	rs5935651	13682066	TCEANC	UTR	G	A	0.3804	3.707	1.1	0.05418	0.6107
**GWAS**	23	kgp22776189	19779469	LOC392452	INTERGENIC	C	A	0.1771	0.3325	0.9316	0.5642	0.882
**GWAS**	23	kgp22752839	25726435	DMD	INTRON	A	C	0.04992	1.912	1.163	0.1667	0.7742
**GWAS**	23	rs1456739	32228514	DMD	INTRON	A	C	0.0524	2.577	1.187	0.1084	0.6976
**GWAS**	23	kgp22795745	48763349	DMD	INTRON	G	A	0.054	3.1	1.203	0.07829	0.6107
**GWAS**	23	rs28582134	90480280	LOC100288789	INTERGENIC	A	G	0.3261	1.101	1.061	0.294	
**ref**	1	rs880315	10796866	CASZ1	INTRON	A	G	0.3816	9.281	0.8751	0.002315	0.0881
**ref**	1	rs17367504	11862778	MTHFR	INTRON	G	A	0.07356	17.32	0.707	0.000032	0.0029
**ref**	1	rs699	230845794	AGT	CODING	A	G	0.201	1.949	0.9286	0.1627	0.7742

**ref**	2	rs35929607	169035736	STK39	INTERGENIC	A	G	0.4634	1.027	1.044	0.3108	0.8376
**ref**	2	rs6749447	169041386	STK39	INTERGENIC	A	C	0.3454	0.5335	1.033	0.4651	0.8811
**ref**	2	rs7571613	190805662	C2orf88	INTRON	G	A	0.09587	0.009049	0.9932	0.9242	0.9974
**ref**	3	rs155524	37562141	ITGA9	INTRON	G	A	0.1602	4.67	1.133	0.03069	0.4928
**ref**	3	rs3774372	41877414	ULK4	CODING	G	A	0.2036	0.9221	1.052	0.3369	0.815
**ref**	4	rs871606	54799245	RP11-317M11	INTERGENIC	G	A	0.232	0.1523	0.9805	0.6963	0.9227
**ref**	4	rs1458038	81164723	FGF5	UTR	A	G	0.4208	36.18	1.295	8.85E-10	1.64E-[illegible]
**ref**	4	rs16998073	81184341	FGF5	UTR	A	T	0.3899	38.17	1.308	1.79E-10	6.68E-[illegible]
**ref**	6	rs805303	31616366	BAG6	INTRON	A	G	0.4122	0.09005	1.013	0.7641	0.9335
**ref**	7	rs17477177	106411858	RNA5SP236	INTERGENIC	G	A	0.1454	0.5155	1.044	0.4728	0.8811
**ref**	8	rs2071518	120435812	NOV	UTR	A	G	0.1666	0.6798	1.048	0.4096	0.8376
**ref**	8	rs1799998	143999600	CYP11B2	UTR	G	A	0.3113	23.86	0.7983	0.000001	0.0001
**ref**	9	rs4977950	24732482	IZUMO3	INTERGENIC	C	G	0.2644	0.433	0.9687	0.5105	0.8811
**ref**	10	rs11191548	104846178	CYP17A1	UTR	G	A	0.2712	11.78	0.8482	0.000599	0.0251
**ref**	10	rs1801253	115805056	ADRB1	CODING	C	G	0.2677	6.999	0.880	0.008153	0.1856
**ref**	11	rs381815	16902268	PLEKHA7	INTRON	A	G	0.1914	1.185	1.06	0.2764	0.7742
**ref**	12	rs2681472	90008959	ATP2B1	INTRON	G	A	0.3701	10.57	0.8664	0.001151	0.0513
**ref**	12	rs17249754	90060586	ATP2B1	INTERGENIC	A	G	0.3616	13.71	0.8486	0.000214	0.0096
**ref**	12	rs2074356	112645401	HECTD4	INTRON	A	G	0.126	15.33	0.7757	0.00009	0.0096
**ref**	12	rs10850411	115387796	LOC102723639	INTERGENIC	A	G	0.3182	0.2798	1.024	0.5969	0.8999
**ref**	15	rs1378942	75077367	CSK	INTRON	A	C	0.1648	5.048	0.8788	0.02466	0.4877
**ref**	16	rs13333226	20365654	UMOD	INTERGENIC	G	A	0.09511	1.16	1.081	0.2815	0.8187
**ref**	17	rs16948048	47440466	ZNF652	INTERGENIC	G	A	0.188	3.745	1.11	0.05296	0.6107

SNP IDs and chromosomal positions are based on NCBI Build 36 of the genome. CHR, chromosome; MAF, minor allele frequency; CHISQ, chi square; OR, odds ratio; FDR, false discovery rate. GWAS, indicates the SNP was selected from genome-wide association study; ref, indicates the SNP was selected from previous published references. A total of 384 SNPs are listed in this table, 13 SNPs grayed out indicates the SNPs were filtered out by quality control. Therefore, 371 SNPs were genotyped in stage 2.

Supplementary Table S3. Haplotype analysis in the follow-up study (stage 2).

chr	SNPs	ref haplotype	effect haplotype	OR (95% CI)	P
1	rs12039843,rs11206154	AA	GC	1.060 (0.975; 1.152)	0.173
1	rs1890265,rs1933299	CC	AA	0.954 (0.876; 1.041)	0.227
1	rs10493345,rs2036867,rs6671446,rs1057815,rs6657480,rs2273367	AAGGGA	GCAAAG	1.046 (0.937; 1.170)	0.420
1	rs4540637,rs2902570,rs1543358	GAG	AGA	1.053 (0.958; 1.164)	0.094
1	rs4657739,rs2281016,rs10918838,rs4657741,rs2235207,rs10753766,rs4534357	GGGGGGA	AAAAAAG	1.042 (0.957; 1.136)	0.340
1	rs4498762,rs4233131,rs10798137	AAA	GCG	1.063 (0.970; 1.167)	0.190
2	rs4852856,rs2041666	AA	GC	1.032 (0.948; 1.122)	0.464
2	rs4848495,rs4088767,rs3849332	AAA	CGG	1.003 (0.864; 1.161)	0.972
2	rs10186364,rs12711901,rs13005524	AGA	GAC	1.000 (0.897; 1.123)	0.993
2	rs13008224,rs6434538	AA	GG	1.108 (0.995; 1.235)	0.174
2	rs2325814,rs1320203	AG	GA	0.962 (0.884; 1.057)	0.384
3	rs4682769,rs4683055	CG	AA	1.032 (0.948; 1.122)	0.470
3	rs2269228,rs6779662	AG	GA	1.035 (0.946; 1.135)	0.457
4	rs3733182,rs3733180	GA	AG	0.947 (0.872; 1.034)	0.200
4	rs4694683,rs2367707	AA	GG	0.984 (0.902; 1.076)	0.722
5	rs3797937,rs1806125	AC	GA	0.924 (0.840; 1.024)	0.246
6	rs6921343,rs9295654,rs9467466	AAA	GGG	0.966 (0.887; 1.058)	0.443
6	rs10456044,rs215012	AG	CA	1.047 (0.967; 1.148)	0.297
6	rs2076472,rs9394761,rs11280,rs9381034	GCGG	AAAA	1.040 (0.947; 1.141)	0.413
8	rs12679456,rs40638	CG	AA	1.041 (0.933; 1.161)	0.376
8	rs1621663,rs1670387,rs7817974	AAA	AAG	0.921 (0.839; 1.011)	0.226
8	rs4870982,rs2029424	AG	GA	1.033 (0.949; 1.124)	0.453
10	rs1907217,rs12572590	AA	GG	0.947 (0.854; 1.055)	0.307

**10**	rs2579787,rs10824393	GA	AC	1.065 (0.975; 1.178)	0.354
**10**	rs10824403,rs11001742	GG	AA	1.065 (0.975; 1.168)	0.162
**10**	rs11002139,rs10824534	AG	GA	1.030 (0.921; 1.154)	0.603
**10**	rs42455,rs673195	GG	AA	0.934 (0.849; 1.033)	0.169
**10**	rs3758395,rs3740065	GG	AA	0.994 (0.911; 1.096)	0.903
**11**	rs6591999,rs7104475,rs7948962,rs7950775,rs1459937,rs4944360,rs10897889,rs1013273,rs7934819,rs12577924,rs7121014	GGAAGGAAGAC	GGAAGGCAGAC	1.082 (0.972; 1.230)	0.307
**11**	rs10790778,rs7925532	AG	GA	1.062 (0.946; 1.191)	0.306
**11**	rs2513996,rs1893073	GC	AA	1.052 (0.951; 1.168)	0.335
**12**	rs2239083,rs2239084	GA	AG	0.989 (0.876; 1.125)	0.858
**13**	rs9567161,rs9525762	GG	GA	1.032 (0.944; 1.134)	0.731
**13**	rs17088537,rs12428541,rs12428506	GGG	AAA	0.992 (0.862; 1.145)	0.918
**14**	rs7146378,rs11159133	AG	GA	0.997 (0.892; 1.121)	0.970
**16**	rs4887941,rs11150047	GA	AC	0.916 (0.834; 1.017)	0.087
**17**	rs17225738,rs8070912	AG	GA	1.092 (0.934; 1.144)	0.059
**17**	rs3786103,rs11078825,rs11658790	GAA	AGC	1.005 (0.921; 1.100)	0.560
**17**	rs2716213,rs2521348	AG	CA	0.970 (0.893; 1.056)	0.475
**17**	rs2251862,rs2074028,rs2251160,rs2074031,rs2715833	CAGAA	AGAGG	0.956 (0.88; 1.045)	0.298
**18**	rs2729741,rs1452539	GG	AA	0.921 (0.832; 1.028)	0.121
**18**	rs9304421,rs8084171	GA	AG	1.069 (0.855; 1.343)	0.658
**20**	rs2206698,rs12626044,rs11087269,rs6045697	AGAA	GAGG	1.000 (0.908; 1.101)	0.977
**21**	rs11702531,rs11701451	GG	AA	0.996 (0.865; 1.152)	0.958
**22**	rs138843,rs138845	AA	GG	1.057 (0.934; 1.205)	0.380
**23**	rs1974522,rs1635246,rs1635248	AAG	GGA	0.927 (0.851; 1.017)	0.089
**23**	rs2361159,rs5935649,rs5935651	GGG	AAA	0.899 (0.857; 1.002)	0.056

Supplementary Table S4. Five SNPs linkage disequilibrium with rs403815 did not associated with hypertension.

SNP ID	position (chr18)	coded/other allele	locat	group	OR(95% CI)	p
**rs9959352**	5998962	C/T	INTRON	Jidong	0.92 (0.82, 1.03)	0.164
				Shanghai	0.98 (0.90, 1.06)	0.235
				Shantou	1.01 (0.93, 1.06)	0.771
				meta	0.98 (0.93, 1.03)	0.420
**rs9967429**	6008208	A/C	INTRON	Jidong	1.07 (0.95, 1.21)	0.250
				Shanghai	0.97 (0.89, 1.05)	0.195
				Shantou	0.96 (0.89, 1.05)	0.472
				meta	0.99 (0.93, 1.04)	0.630
**rs4798428**	6022320	C/T	INTRON	Jidong	0.88 (0.77, 1.00)	0.064
				Shanghai	0.99 (0.90, 1.09)	0.595
				Shantou	0.99 (0.89, 1.09)	0.860
				meta	0.96 (0.90, 1.03)	0.270
**rs1818871**	6117638	A/C	INTRON	Jidong	0.96 (0.87, 1.05)	0.417
				Shanghai	1.08 (1.01, 1.16)	0.514
				Shantou	1.00 (0.93, 1.08)	0.858
				meta	1.02 (0.96, 1.09)	0.540
**rs636589**	6274886	G/T	INTRON	Jidong	1.02 (0.91, 1.13)	0.708
				Shanghai	1.05 (0.97, 1.13)	0.215
				Shantou	0.98 (0.91, 1.06)	0.696
				meta	1.02 (0.97, 1.07)	0.500

OR (95% CI) and *P* value of each SNP in group Jingdong, Shanghai and Shantou were adjusted by age, sex and body mass index (BMI).

Supplementary Table S5. Total CNV burden among cases and controls (stage 1).

	Cases	Controls	*P*
**Number of Deletions**	53	66	-
**Number of Duplications**	385	278	-
**PROP**	0.5277	0.4008	0.003

PROP, proportion of sample with one or more CNVs.

P values were calculated by 10000 permutations.

Supplementary Table S6. 11 CNV regions implicated by PLINK.

Locus	Type	Genomic coordinates	Case	Control
**1q31.3**	Duplication	195,672,512-195,752,242	3	0
**2q32.1**	Duplication	184,700,135-186,100,554	2	0
**4p16.1**	Duplication	9,352,164-10,116,974	2	0
**5q21.3**	Duplication	106,512,777-107,922,654	2	0
**5q34**	Duplication	161,214,115-163,000,327	2	1
**8q24.23**	Duplication	136,812,004-138,278,663	2	0
**17p13.1**	Duplication	6,904,240-10,885,246	2	0
**1p36.33**	Deletion	1,105,935-2,201,787	2	0
**7q36.3**	Deletion	155,701,250-157,223,560	2	0
**16q24.2**	Deletion	88,539,204-90,214,527	2	0
**20p12.1**	Deletion	16,552,014-17,600,268	3	0

Supplementary Table S7. Baseline characteristics of participants for CNV analysis.

	case	control
**N**	989	1022
**Age (S.D.)**	45.7(3.50)	70.5(4.3)
**Gender (% female)**	25.2	32.7
**BMI, kg/m2 (S.D.)**	26.8(3.5)	24.5(3.7)
**SBP (S.D.)**	143.9(15.7)	115.0(8.4)
**DBP (S.D.)**	97.87(9.0)	71.3(6.7)

N, sample size; S.D., standard deviation; BMI, body mass index; SBP, systolic blood pressure; DBP, diastolic blood pressure.

Supplementary Table S8. CNV discovery of 16q24.2 locus.

	**duplications**	**deletions**	**total CNV sample**	***P***
**cases (n=989)**	22	3	26	0.048
**controls (n=1022)**	15	0	14	

P, P value of Pearson correlation test.

Bold font indicates CNV rates between cases and controls achieved significant difference (*P*<0.05).

Supplementary Table S9. The spectrum of phosphorylation proteins as detected by phospho-antibody microarray.

Protein Name (phosphorylation site)	WT-Phos/ Unphos	TG-Phos/ Unphos	TG/WT
14-3-3 theta/tau (Phospho-Ser232)	2.32	1.49	0.64
14-3-3 zeta/delta (Phospho-Thr232)	0.06	0.17	2.76
4E-BP1 (Phospho-Ser65)	0.05	0.04	0.77
4E-BP1 (Phospho-Thr36)	0.99	1.29	1.30
4E-BP1 (Phospho-Thr70)	0.86	0.57	0.66
Abl1 (Phospho-Thr754/735)	1.51	0.90	0.60
ACC1 (Phospho-Ser79)	0.98	0.60	0.62
ACC1 (Phospho-Ser80)	0.09	0.06	0.75
AFX/FOXO4 (Phospho-Ser197)	1.18	0.84	0.71
AKT (Phospho-Ser473)	1.15	0.86	0.75
AKT (Phospho-Tyr326)	0.82	1.63	2.00
AKT1 (Phospho-Thr450)	1.28	0.76	0.59
AKT1 (Phospho-Thr72)	0.46	0.63	1.37
AKT1S1 (Phospho-Thr246)	0.06	0.10	1.63
ALK (Phospho-Tyr1507)	1.71	3.66	2.14
ALK (Phospho-Tyr1604)	0.75	2.11	2.83
AMPK1 (Phospho-Thr172)	1.73	1.37	0.79
Amyloid beta A4 (Phospho-Thr743/668)	0.85	0.41	0.48
Arrestin-1 (Phospho-Ser412)	0.32	0.49	1.51
ASK1 (Phospho-Ser966)	1.35	1.04	0.77
ATF2 (Phospho-Ser62/44)	0.05	0.19	3.47
ATP1A1/Na+K+ ATPase1 (Phospho-Ser23)	1.73	1.30	0.75
ATRIP (Phospho-Ser68/72)	0.02	0.06	3.56
AurA (Phospho-Thr288)	2.12	2.91	1.37

BAD (Phospho-Ser91/128)	0.61	0.88	1.44
BCL-2 (Phospho-Ser70)	2.15	1.40	0.65
BCL-XL (Phospho-Thr47)	0.03	0.05	1.62
BCR (Phospho-Tyr360)	1.74	3.28	1.88
BID (Phospho-Ser78)	0.60	0.46	0.77
BLNK (Phospho-Tyr96)	0.16	0.36	2.24
B-RAF (Phospho-Ser446)	2.59	1.79	0.69
BRCA1 (Phospho-Ser1423)	0.22	0.33	1.49
BRCA1 (Phospho-Ser1457)	1.37	0.76	0.55
c-Abl (Phospho-Tyr412)	0.58	0.71	1.24
Calmodulin (Phospho-Thr79/Ser81)	0.05	0.69	14.50
CaMK1-alpha (Phospho-Thr177)	1.27	0.67	0.53
CASP1 (Phospho-Ser376)	0.83	1.10	1.32
CASP2 (Phospho-Ser157)	0.94	1.20	1.27
CASP6 (Phospho-Ser257)	0.21	0.34	1.62
CASP9 (Phospho-Thr125)	0.72	0.39	0.54
Caspase 9 (Phospho-Ser144)	1.59	1.13	0.71
Caspase 9 (Phospho-Tyr153)	0.13	0.24	1.87
Caspase-3 (Phospho-Ser150)	1.29	0.76	0.59
Catalase (Phospho-Tyr385)	0.25	0.33	1.30
Catenin beta (Phospho-Ser33)	0.61	1.08	1.77
Caveolin-1 (Phospho-Tyr14)	1.47	0.86	0.59
CD19 (Phospho-Tyr531)	4.49	1.41	0.31
CD227/mucin 1 (Phospho-Tyr1243)	1.86	1.41	0.76
CD3Z (Phospho-Tyr142)	1.35	0.60	0.45
CD5 (Phospho-Tyr453)	0.31	0.77	2.53
CDC2 (Phospho-Tyr15)	3.62	2.07	0.57
CDC25A (Phospho-Ser75)	0.71	1.05	1.47

CDC25B (Phospho-Ser353)	3.24	1.95	0.60
CDK1/CDC2 (Phospho-Thr14)	0.63	1.09	1.74
CDK2 (Phospho-Thr160)	0.86	0.64	0.75
CDK5 (Phospho-Tyr15)	0.17	0.32	1.87
CDK7 (Phospho-Thr170)	1.72	1.28	0.74
Chk1 (Phospho-Ser280)	1.59	1.21	0.76
Chk1 (Phospho-Ser317)	0.48	0.57	1.20
Chk1 (Phospho-Ser345)	0.85	1.10	1.30
Chk2 (Phospho-Ser516)	0.51	0.74	1.46
Chk2 (Phospho-Thr383)	1.94	0.88	0.45
c-Jun (Phospho-Ser63)	0.81	1.46	1.80
c-Jun (Phospho-Thr91)	0.93	1.20	1.29
c-Jun (Phospho-Thr93)	2.25	1.61	0.72
c-Kit (Phospho-Tyr721)	4.72	8.24	1.74
claudin 3 (Phospho-Tyr219)	1.09	2.65	2.44
claudin 7 (Phospho-Tyr210)	2.23	2.93	1.32
Cofilin (Phospho-Ser3)	0.74	0.55	0.74
Connexin 43 (Phospho-Ser367)	1.10	0.85	0.77
Cortactin (Phospho-Tyr421)	2.36	1.02	0.43
Cortactin (Phospho-Tyr466)	0.93	0.73	0.78
CPI17 alpha (Phospho-Thr38)	1.23	0.95	0.77
c-PLA2 (Phospho-Ser505)	0.75	0.99	1.33
CREB (Phospho-Ser121)	0.88	1.44	1.63
CREB (Phospho-Ser129)	2.76	1.45	0.53
CREB (Phospho-Ser142)	1.32	0.69	0.53
Cyclin B1 (Phospho-Ser126)	0.33	0.22	0.68
Cyclin E1 (Phospho-Thr77)	0.08	0.04	0.47
DAB1 (Phospho-Tyr220)	0.80	1.11	1.39

DAPP1 (Phospho-Tyr139)	0.63	2.42	3.82
DARPP-32 (Phospho-Thr34)	0.38	0.60	1.56
DAXX (Phospho-Ser668)	0.02	0.05	2.05
DDX5/DEAD-box protein 5 (Phospho-Tyr593)	0.64	2.65	4.14
DNA-PK (Phospho-Thr2647)	1.17	0.88	0.75
Dok-2 (Phospho-Tyr299)	0.76	0.95	1.26
DYN1 (Phospho-Ser774)	0.03	0.06	1.70
EGFR (Phospho-Ser1070)	0.05	0.10	1.96
EGFR (Phospho-Tyr1016)	0.54	0.74	1.36
EGFR (Phospho-Tyr1069)	1.68	1.28	0.76
EGFR (Phospho-Tyr1092)	0.71	0.38	0.53
EGFR (Phospho-Tyr1172)	0.40	0.28	0.68
EGFR (Phospho-Tyr869)	0.04	0.07	1.73
eIF2A (Phospho-Ser51)	0.86	0.54	0.63
Elk1 (Phospho-Thr417)	1.35	1.99	1.47
EPHB1/2 (Phospho-Tyr594/604)	1.27	1.01	0.80
Epo-R (Phospho-Tyr368)	0.82	1.14	1.39
ERK3 (Phospho-Ser189)	2.66	0.82	0.31
Estrogen Receptor-alpha (Phospho-Ser104)	0.75	0.44	0.59
Estrogen Receptor-alpha (Phospho-Ser118)	1.85	1.17	0.63
Estrogen Receptor-alpha (Phospho-Ser167)	1.73	1.04	0.60
ETK (Phospho-Tyr40)	0.85	2.81	3.29
ETK (Phospho-Tyr566)	13.41	28.59	2.13
Ezrin (Phospho-Thr566)	0.11	0.14	1.30
Ezrin (Phospho-Tyr478)	0.76	1.02	1.34
FAK (Phospho-Ser910)	0.17	0.10	0.55
FAK (Phospho-Tyr397)	1.00	0.56	0.56
FAK (Phospho-Tyr576)	0.85	1.09	1.28

FAK (Phospho-Tyr861)	1.66	1.27	0.77
FER (Phospho-Tyr402)	2.86	6.54	2.28
FGFR1 (Phospho-Tyr766)	0.80	0.29	0.36
FKHR (Phospho-Ser256)	0.73	0.91	1.25
FKHR/FOXO1A (Phospho-Ser329)	0.63	1.04	1.65
FKHRL1/FOXO3A (Phospho-Ser253)	0.41	0.27	0.65
FosB (Phospho-Ser27)	0.82	1.23	1.49
FOXO1/3/4-pan (Phospho-Thr24/32)	0.58	0.91	1.57
G3BP-1 (Phospho-Ser232)	0.76	0.59	0.77
GAP43 (Phospho-Ser41)	1.05	1.26	1.20
GATA1 (Phospho-Ser142)	1.65	1.21	0.73
GATA1 (Phospho-Ser310)	0.54	0.72	1.34
GluR1 (Phospho-Ser849)	0.04	0.03	0.75
GluR2 (Phospho-Ser880)	0.06	0.08	1.23
GRK2 (Phospho-Ser29)	0.11	0.22	1.97
GTPase activating protein (Phospho-Ser387)	0.72	0.92	1.27
HCK (Phospho-Tyr410)	0.02	0.06	3.07
HDAC1 (Phospho-Ser421)	0.02	0.03	1.30
HDAC2 (Phospho-Ser394)	0.70	1.49	2.14
HDAC3 (Phospho-Ser424)	0.38	0.62	1.64
HDAC5 (Phospho-Ser259)	0.56	0.91	1.61
HDAC5 (Phospho-Ser498)	1.01	1.55	1.54
HDAC8 (Phospho-Ser39)	0.73	1.04	1.43
HER2 (Phospho-Tyr1221/Tyr1222)	0.38	0.58	1.53
HER2 (Phospho-Tyr877)	1.80	1.38	0.77
HER3/ErbB3 (Phospho-Tyr1289)	0.76	1.00	1.30
HER4/ErbB4 (Phospho-Tyr1284)	0.64	0.87	1.36
HNF4 alpha (Phospho-Ser313)	2.04	1.61	0.79

HRS (Phospho-Tyr334)	0.83	1.48	1.79
HSL (Phospho-Ser554)	0.48	0.24	0.51
HSP27 (Phospho-Ser15)	1.44	3.16	2.20
HSP27 (Phospho-Ser15/Ser78/Ser82)	4.88	6.73	1.38
HSP90 co-chaperone Cdc37 (Phospho-Ser13)	0.63	1.00	1.59
HSP90B (Phospho-Ser226)	7.60	9.62	1.27
IGF1R (Phospho-Tyr1161)	0.98	1.26	1.28
IKK-alpha/beta (Phospho-Ser180/181)	0.90	0.67	0.74
IKK-beta (Phospho-Tyr188)	0.20	0.28	1.44
IKK-beta (Phospho-Tyr199)	0.05	0.10	2.00
IKK-gamma (Phospho-Ser31)	0.06	0.04	0.72
IKK-gamma (Phospho-Ser85)	0.56	0.73	1.31
IL-10R-alpha (Phospho-Tyr496)	2.34	2.81	1.20
IL-13R/CD213a1 (Phospho-Tyr405)	2.31	4.23	1.83
IL3RB (Phospho-Tyr593)	1.00	1.81	1.81
IL-4R/CD124 (Phospho-Tyr497)	0.90	1.38	1.53
Integrin beta-1 (Phospho-Thr788)	0.81	1.09	1.35
Integrin beta-3 (Phospho-Tyr773)	1.53	1.95	1.27
Integrin beta-3 (Phospho-Tyr785)	1.50	0.99	0.66
IR (Phospho-Tyr1361)	1.25	3.12	2.50
IRS-1 (Phospho-Ser307)	1.93	1.16	0.60
IRS-1 (Phospho-Ser312)	0.40	0.77	1.96
IRS-1 (Phospho-Ser323)	0.27	0.39	1.41
IRS-1 (Phospho-Ser636)	1.40	1.69	1.21
IRS-1 (Phospho-Ser639)	0.12	0.07	0.56
IRS-1 (Phospho-Ser794)	1.76	1.21	0.69
JAK2 (Phospho-Tyr1007)	0.38	0.48	1.25
JAK2 (Phospho-Tyr221)	0.82	1.45	1.76

JNK1/2/3 (Phospho-Thr183/Tyr185)	0.52	1.20	2.32
JunB (Phospho-Ser259)	1.68	1.26	0.75
JunB (Phospho-Ser79)	1.22	0.94	0.77
Keratin 8 (Phospho-Ser73)	0.86	0.64	0.75
Kv1.3/KCNA3 (Phospho-Tyr135)	1.38	5.73	4.15
LAT (Phospho-Tyr191)	0.80	0.61	0.76
LCK (Phospho-Ser59)	1.08	1.37	1.28
LCK (Phospho-Tyr192)	0.72	1.03	1.43
LIMK1 (Phospho-Thr508)	0.92	1.38	1.50
LKB1 (Phospho-Thr189)	1.35	0.99	0.74
MAPKAPK2 (Phospho-Thr334)	0.02	0.21	10.35
MARCKS (Phospho-Ser158)	0.75	1.01	1.35
MDM2 (Phospho-Ser166)	0.02	0.03	1.74
MEF2A (Phospho-Ser408)	0.34	0.24	0.70
MEF2A (Phospho-Thr312)	0.14	0.35	2.43
MEK1 (Phospho-Ser217)	1.67	1.23	0.74
MEK1 (Phospho-Ser221)	3.25	1.73	0.53
MEK1 (Phospho-Thr286)	0.05	0.45	8.25
MEK2 (Phospho-Thr394)	3.14	2.47	0.79
Merlin (Phospho-Ser10)	0.20	2.83	13.91
Merlin (Phospho-Ser518)	1.82	0.93	0.51
MKK6 (Phospho-Ser207)	1.03	0.35	0.34
MKK7/MAP2K7 (Phospho-Ser271)	1.89	1.34	0.71
MKP-1 (Phospho-Ser359)	0.61	1.04	1.69
Mnk1 (Phospho-Thr385)	2.13	1.46	0.68
MSK1 (Phospho-Ser360)	0.03	0.08	2.37
MSK1 (Phospho-Thr581)	0.80	0.54	0.68
Mst1/Mst2 (Phospho-Thr183)	1.04	0.81	0.78

mTOR (Phospho-Ser2481)	1.05	0.73	0.69
mTOR (Phospho-Thr2446)	1.40	2.27	1.62
Myc (Phospho-Ser373)	0.56	0.85	1.52
Myc (Phospho-Ser62)	0.88	1.08	1.22
Myc (Phospho-Thr358)	0.03	0.04	1.59
NFAT4 (Phospho-Ser165)	1.00	1.38	1.38
NFkB-p100/p52 (Phospho-Ser869)	0.52	0.66	1.26
NFkB-p105/p50 (Phospho-Ser337)	6.44	4.27	0.66
NFkB-p105/p50 (Phospho-Ser893)	1.29	0.59	0.46
NFkB-p65 (Phospho-Ser276)	0.63	1.11	1.76
NFkB-p65 (Phospho-Ser468)	3.61	1.30	0.36
NFkB-p65 (Phospho-Ser536)	1.78	1.06	0.60
NFkB-p65 (Phospho-Thr435)	1.22	1.67	1.37
p130Cas (Phospho-Tyr410)	0.69	1.50	2.17
p21Cip1 (Phospho-Thr145)	0.93	1.22	1.32
p27Kip1 (Phospho-Ser10)	1.29	1.58	1.22
p27Kip1 (Phospho-Thr187)	0.93	2.42	2.60
p38 MAPK (Phospho-Thr180/Tyr182)	1.23	1.45	1.18
p44/42 MAP Kinase (Phospho-Thr202/Tyr204)	4.98	5.44	1.09
p53 (Phospho-Ser315)	0.29	0.16	0.56
p53 (Phospho-Ser378)	1.53	1.99	1.30
p53 (Phospho-Ser46)	3.92	2.46	0.63
p53 (Phospho-Ser6)	0.81	1.06	1.31
P70S6K (Phospho-Ser371)	1.00	1.44	1.44
P70S6K (Phospho-Ser418)	1.27	1.88	1.48
P70S6K (Phospho-Thr229)	0.63	0.86	1.38
P73 (Phospho-Tyr99)	1.13	0.80	0.71
P90RSK (Phospho-Thr359/Ser363)	0.05	0.07	1.41

P90RSK (Phospho-Thr573)	0.31	0.41	1.33
P95/NBS1 (Phospho-Ser343)	0.12	0.23	1.99
PAK1 (Phospho-Ser204)	1.27	1.61	1.27
PAK1/2 (Phospho-Ser199)	0.71	1.22	1.72
PAK2 (Phospho-Ser192)	0.96	1.16	1.21
PDGF R alpha (Phospho-Tyr849)	2.05	1.17	0.57
PDGF R beta (Phospho-Tyr740)	2.40	3.12	1.30
PDK1 (Phospho-Ser241)	0.14	0.28	2.01
PEA-15 (Phospho-Ser116)	1.00	1.33	1.33
PECAM-1 (Phospho-Tyr713)	0.03	0.08	2.33
PI3-kinase p85-subunit alpha/gamma (Phospho-Tyr467/Tyr199)	0.74	0.95	1.28
PKA CAT (Phospho-Thr197)	0.58	0.87	1.49
PKC alpha (Phospho-Tyr657)	2.29	1.69	0.74
PKC alpha/beta II (Phospho-Thr638)	1.08	0.76	0.70
PKC delta (Phospho-Thr505)	1.04	0.66	0.63
PKC epsilon (Phospho-Ser729)	1.87	1.14	0.61
PKC theta (Phospho-Ser676)	1.50	0.87	0.58
PKC theta (Phospho-Thr538)	0.01	0.03	2.90
PKC zeta (Phospho-Thr560)	1.18	0.92	0.78
PKD1/PKC mu (Phospho-Ser910)	1.20	0.84	0.70
PKD2 (Phospho-Ser876)	1.95	1.46	0.75
PKR (Phospho-Thr451)	1.15	0.76	0.66
PLC beta3 (Phospho-Ser537)	1.52	1.21	0.80
PLCG1 (Phospho-Tyr771)	0.98	1.47	1.50
PLCG2 (Phospho-Tyr1217)	0.87	0.67	0.76
PLCG2 (Phospho-Tyr753)	0.36	0.63	1.77
PLD1 (Phospho-Ser561)	0.93	1.69	1.81

PPAR-BP (Phospho-Thr1457)	1.23	0.81	0.65
Progesterone Receptor (Phospho-Ser190)	0.50	0.87	1.76
Pyk2 (Phospho-Tyr580)	0.32	0.13	0.40
Rac1/cdc42 (Phospho-Ser71)	1.62	1.20	0.74
Raf1 (Phospho-Ser338)	0.87	1.34	1.53
Raf1 (Phospho-Tyr341)	0.75	1.04	1.39
Rb (Phospho-Ser795)	0.72	0.45	0.63
Rb (Phospho-Ser807)	0.34	0.21	0.60
Rel (Phospho-Ser503)	0.89	0.57	0.65
Ret (Phospho-Tyr905)	2.02	1.50	0.74
Rho/Rac guanine nucleotide exchange factor 2 (Phospho-Ser885)	2.01	1.44	0.71
RyR2 (Phospho-Ser2808)	1.61	0.84	0.52
SEK1/MKK4 (Phospho-Ser80)	0.10	0.20	2.05
Shc (Phospho-Tyr349)	0.56	0.09	0.15
Shc (Phospho-Tyr427)	0.24	0.15	0.61
SHP-2 (Phospho-Tyr580)	1.22	0.94	0.77
SLP-76 (Phospho-Tyr128)	0.56	1.33	2.38
Smad1 (Phospho-Ser187)	1.54	0.84	0.55
Smad1 (Phospho-Ser465)	0.60	0.44	0.74
Smad2 (Phospho-Ser250)	0.87	1.18	1.36
Smad2 (Phospho-Ser467)	0.90	0.55	0.62
Smad2 (Phospho-Thr220)	0.67	0.83	1.23
Smad2/3 (Phospho-Thr8)	1.35	1.73	1.28
Smad3 (Phospho-Ser204)	0.57	0.80	1.42
SMC1 (Phospho-Ser957)	0.87	1.58	1.83
Src (Phospho-Ser75)	0.79	0.95	1.20
Src (Phospho-Tyr529)	0.52	0.94	1.80

SRF (Phospho-Ser77)	1.33	0.75	0.56
SRF (Phospho-Ser99)	0.12	0.16	1.25
STAM2 (Phospho-Tyr192)	1.79	2.83	1.58
STAT1 (Phospho-Ser727)	0.96	0.67	0.70
STAT1 (Phospho-Tyr701)	3.15	2.50	0.79
STAT3 (Phospho-Ser727)	1.05	0.79	0.75
STAT3 (Phospho-Tyr705)	0.60	0.89	1.49
STAT4 (Phospho-Tyr693)	1.64	1.30	0.79
STAT5A (Phospho-Ser780)	0.03	0.07	2.47
STAT6 (Phospho-Thr645)	0.06	0.08	1.41
STAT6 (Phospho-Tyr641)	1.20	1.63	1.36
Survivin (Phospho-Thr117)	1.38	0.96	0.69
SYK (Phospho-Tyr348)	0.54	0.36	0.67
Synuclein alpha (Phospho-Tyr125)	1.09	1.34	1.24
Synuclein alpha (Phospho-Tyr133)	2.56	1.67	0.65
TAK1 (Phospho-Thr184)	0.70	1.06	1.53
Tau (Phospho-Ser214)	0.47	0.20	0.43
Tau (Phospho-Ser356)	0.02	0.22	9.19
Tau (Phospho-Ser422)	1.44	0.93	0.65
Tau (Phospho-Thr205)	0.42	0.25	0.60
TOP2A/DNA topoisomerase II (Phospho-Ser1106)	0.11	0.23	2.19
Tuberin/TSC2 (Phospho-Ser939)	0.17	0.24	1.39
Tyrosine Hydroxylase (Phospho-Ser19)	0.37	0.25	0.66
Tyrosine Hydroxylase (Phospho-Ser40)	0.83	0.59	0.72
Tyrosine Hydroxylase (Phospho-Ser8)	0.32	0.52	1.62
VASP (Phospho-Ser238)	1.71	1.08	0.63
VAV1 (Phospho-Tyr174)	1.29	1.55	1.20
VAV2 (Phospho-Tyr142)	1.45	2.15	1.48

VEGFR2 (Phospho-Tyr1054)	1.11	2.24	2.02
Vinculin (Phospho-Tyr821)	2.01	6.40	3.18
WASP (Phospho-Tyr290)	1.10	0.36	0.33
WAVE1 (Phospho-Tyr125)	0.89	1.72	1.93
Zap-70 (Phospho-Tyr493)	0.91	1.85	2.03

Supplementary Table S10. The 3,289 peaks obtained by ChIP sequencing.

Peak region			length	summit	tags	-10*LOG10(pvalue)	fold_enrichment	Gene:Start-End:Locate
chr	start	end						
chr1	228757723	228758406	684	490	37	200.94	21.62	-
chr8	43793463	43794259	797	513	120	487.88	19.55	-
chr8	86823178	86823829	652	199	54	285.88	18.52	-
chr5	49409807	49410542	736	488	39	178.67	17.78	-
chrY	9201093	9201605	513	242	25	134.36	16.04	728395:9195451-9218479:Genebody
chr21	9826880	9827543	664	239	113	445.5	15.85	-
chr9_gl000199_random	87790	88265	476	263	37	201.39	15.09	-
chr2	92291235	92292012	778	273	58	189.24	15.06	-
chr22	21539137	21539822	686	468	25	106.7	14.65	-
chr2	92312707	92313586	880	277	82	331.43	14.53	-
chr7	23942	24802	861	466	33	120.2	14.53	-
chr4	9370297	9370999	703	285	60	218.86	14.37	391622:9369599-9370794:Genebody
chr2	92306850	92308136	1287	530	143	254.36	13.75	-
chr4	167501335	167501960	626	158	23	90.8	13.75	-
chr8	86559626	86560323	698	439	32	159.67	13.25	-
chr9	141122344	141122949	606	409	22	84.56	12.8	100132403:141106636-141134172:Geneb

								ody
chr19	8885337	8885772	436	250	20	106.07	12.56	-
chr4	9245228	9245641	414	216	19	101.61	12.56	391627:9245604-9247196:Upstream
chrY	58904811	58905548	738	507	136	435.23	12.56	-
chr8	6856859	6857387	529	343	27	142.46	12.5	1667:6854288-6856724:Upstream
chr9_gl000199_random	72392	73140	749	496	58	179.6	12.5	-
chrUn_gl000239	27195	27871	677	190	32	133.42	12.5	-
chrY	10089727	10090497	771	379	36	130.68	12.5	-
chr19_gl000208_random	2	425	424	199	19	97.47	12.16	-
chr9_gl000199_random	155501	156425	925	455	88	276.8	11.95	-
chr17	41645926	41646367	442	187	22	112.64	11.88	-
chr16	90183479	90183808	330	166	17	112.87	11.86	-
chr19	2233984	2234541	558	194	21	94.61	11.86	84444:2164147-2232576:Downstream
chr4	52676704	52677245	542	347	20	89.35	11.86	-
chr5	49414178	49414736	559	198	23	109.9	11.86	-
chr9_gl000199_random	34317	34656	340	171	17	112.87	11.86	-
chrY	13108955	13109740	786	420	24	81.1	11.86	-

chr1	148010271	148011288	1018	663	49	161.79	11.84	25832:148003642-148025848:Genebody
chrUn_gl000226	9280	9952	673	384	38	126.14	11.54	-
chr19	48453090	48453873	784	239	40	161.73	11.5	100170226:48453552-48453671:Upstream
chr10	42395467	42396701	1235	633	270	450.17	11.44	-
chrY	13694478	13695468	991	532	47	107.49	11.36	-
chr1	145356171	145356790	620	227	22	80.86	11.31	100132406:145293370-145368682:Genebody
chr9_gl000199_random	41866	42431	566	326	20	78.02	11.25	-
chrY	58826712	58828025	1314	643	288	578.87	11.2	-
chr2	52066941	52067714	774	391	18	52.18	11.16	-
chr5	17599004	17599517	514	318	20	85.76	11.16	-
chr7	158129536	158130446	911	443	22	63.7	11.16	5799:157331750-158380482:Genebody
chr8	86781652	86782088	437	199	17	81.63	11.16	254958:86758550-86789306:Genebody
chrUn_gl000225	87950	88445	496	212	19	88.33	11.16	-
chrY	13105961	13106452	492	317	18	81.33	11.16	-

chr2	21023937	21024373	437	244	17	81.34	11.11	60526:20884818-21022827:Upstream
chr3	196625184	196625924	741	502	29	86.74	11.11	205564:196594726-196661582:Genebody
chr17	61993679	61995031	1353	610	234	538.47	11.09	2688:61994563-61996198:Downstream
chr4	9249687	9250619	933	685	47	184.62	10.94	-
chr4	49318685	49319406	722	269	38	100.44	10.9	-
chrY	58865630	58867752	2123	442	122	152.41	10.89	-
chr2	171681610	171682447	838	361	22	67.51	10.81	2571:171673199-171699268:Genebody
chr5	11354	12155	802	265	50	149.11	10.8	-
chr7	56440988	56441543	556	295	41	149.08	10.71	-
chr3	41208663	41209333	671	319	22	78.5	10.62	-
chr4	125933663	125934267	605	414	21	79.85	10.62	-
chr22	51081825	51082689	865	444	30	79.3	10.5	-
chr14	66098526	66099143	618	423	21	86.62	10.46	2530:65877839-66209961:Genebody
chr15	92272785	92273322	538	360	16	61.58	10.46	-
chr17	36329795	36330297	503	217	17	72.56	10.46	-
chr18	40658329	40658890	562	371	18	72.41	10.46	6014:40323192-40695657:Genebody

chr2	90489333	90489742	410	218	16	77.92	10.46	-
chr4	112866262	112866738	477	195	16	68.69	10.46	-
chr8	7599185	7599680	496	231	21	104.03	10.46	441324:7595908-7598917:Downstream
chr9_gl000199_random	149049	149552	504	197	19	87.17	10.46	-
chrUn_gl000219	110368	110979	612	418	19	73.48	10.46	-
chr2	148012490	148013223	734	414	21	73.27	10.42	-
chr4	49293311	49294259	949	494	28	69.22	10.42	-
chr4	49103194	49103862	669	369	37	130.94	10.32	-
chr3	188766957	188767796	840	466	20	56.03	10.14	-
chr4	49121690	49122633	944	524	27	50.68	10.09	-
chrY	58820893	58821357	465	188	46	159.99	10.04	-
chrUn_gl000220	101364	102453	1090	450	133	306.47	10.03	-
chr18	73576713	73577362	650	278	29	104.96	9.9	-
chr2	226316368	226317208	841	429	21	59.64	9.87	57624:226265601-226518734:Genebody
chr3	100305733	100306287	555	323	17	62.79	9.87	-
chr8	29629045	29629794	750	369	18	50.56	9.87	-
chr10	42538851	42539684	834	531	53	177.74	9.86	-
chr1	33110919	33111302	384	193	14	64.15	9.77	339487:33087308-33116185:Genebody
chr1	57183412	57183987	576	191	18	70.78	9.77	199920:57184

								477-57285369:Downstream
chr1	145351963	145352693	731	375	19	61.53	9.77	100132406:145293370-145368682:Genebody
chr1	231370384	231371140	757	274	24	90.84	9.77	128061:231359509-231376924:Genebody
chr10	32634862	32635290	429	188	15	67.65	9.77	80314:32557858-32636113:Genebody
chr10	129443125	129443742	618	199	16	53.66	9.77	-
chr11	3675541	3675908	368	185	14	71.67	9.77	417:3666360-3685646:Genebody
chr11	68265756	68266344	589	397	15	50.24	9.77	NM_001164160:68228185-68382799:Genebody
chr11	127845704	127846221	518	260	18	77.85	9.77	-
chr14	19872833	19873335	503	314	18	79.83	9.77	-
chr2	60807728	60808185	458	198	15	63.94	9.77	-
chr2	121130236	121130920	685	378	21	78.76	9.77	-
chr2	143465333	143465894	562	369	18	72.41	9.77	-
chr2	157945410	157945926	517	196	16	63.91	9.77	-
chr3	22429822	22430483	662	360	19	68.12	9.77	-
chr3	106564183	106564689	507	317	16	65.06	9.77	-

chr4	138797069	138797600	532	256	19	83.3	9.77	-
chr6	14345307	14345917	611	198	16	54.3	9.77	-
chr6	36220865	36221536	672	264	18	60.87	9.77	285848:36210944-36276372:Genebody
chr7	143890715	143891286	572	249	18	71.24	9.77	445328:143883176-143892791:Genebody
chr8	25861199	25861767	569	199	17	64.85	9.77	64641:25701572-25902392:Genebody
chr3	11398652	11399231	580	391	20	74.47	9.76	10533:11314009-11599132:Genebody
chr19	27731864	27733141	1278	226	223	514.75	9.75	-
chr1	1865212	1866008	797	484	22	73.35	9.72	-
chr3	128947193	128947752	560	318	18	72.35	9.72	-
chr4	116331620	116332199	580	200	16	56.99	9.72	-
chr5	130714791	130715342	552	360	15	53.45	9.72	56990:130599701-130730380:Genebody
chrX	58566766	58567567	802	463	25	74.76	9.66	-
chr14	61977475	61978079	605	189	20	75.03	9.62	5583:61788514-62017695:Genebody
chr19	10519216	10519807	592	200	17	57.33	9.62	-
chr2	3584260	3584987	728	371	23	80.47	9.62	-
chr3	184991390	184992217	828	429	21	58.92	9.62	-

chr8	29745668	29746382	715	467	25	95.22	9.62	-
chr1	54689504	54690135	632	413	24	92.73	9.52	-
chr1	247603380	247603850	471	198	17	66.73	9.52	114548:247579457-247612404:Genebody
chr15	65313098	65313852	755	282	20	54.71	9.52	123263:65293850-65321977:Genebody
chr2	103877775	103878457	683	414	19	55.68	9.52	-
chr2	122817607	122818062	456	258	19	82.98	9.52	-
chr3	50068249	50069001	753	391	20	54.88	9.52	10180:49977476-50114684:Genebody
chrX	61691369	61692416	1048	344	117	277.35	9.49	-
chr1	216721537	216722155	619	438	18	64.06	9.46	2104:216676587-216896807:Genebody
chr10	99067264	99067840	577	319	18	68.59	9.46	-
chr10	110483301	110483914	614	178	19	71.07	9.46	-
chr4	132656122	132656608	487	198	17	72.6	9.46	-
chr5	80983072	80983561	490	197	15	58.42	9.46	23635:80715672-81047072:Genebody
chr6	25588740	25589439	700	252	18	56.35	9.46	55604:25279655-25620756:Genebody
chr6	37809523	37810441	919	499	25	77.88	9.46	60685:37787306-38122397:

								Genebody
chr6	80965576	80966144	569	370	16	50.39	9.46	594:80816343-81055987:Genebody
chr6	120337628	120337997	370	186	14	69.83	9.46	-
chr2	45641388	45642170	783	535	31	94.9	9.43	55133:45615819-45838433:Genebody
chr10	70089213	70089838	626	267	19	64.51	9.38	64081:70042416-70092684:Genebody
chr14	20045420	20046001	582	193	17	56.84	9.38	-
chr14	102190585	102191211	627	200	20	70.68	9.38	-
chr9	34777625	34778225	601	262	18	60.98	9.38	-
chr13	62841110	62841675	566	181	16	52.44	9.37	-
chr9_gl000199_random	109337	110158	822	389	20	52.36	9.37	-
chr16	57422399	57423473	1075	536	45	123.63	9.32	-
chr2	97799657	97800351	695	482	23	76.36	9.3	375248:97779232-97930255:Genebody
chr2	228873999	228874952	954	479	24	57.41	9.3	80309:228844669-229046361:Genebody
chr1	143282787	143283780	994	441	37	59.98	9.29	-
chrY	58893275	58894029	755	417	42	111.92	9.26	-
chr2	33818778	33819452	675	350	20	56.09	9.24	25940:33808728-33824362:

								Genebody
chr1	2832011	2832575	565	379	19	74.96	9.21	-
chr1	106784490	106785247	758	363	21	66.99	9.21	-
chr1	142951838	142952881	1044	696	36	132.02	9.21	-
chr19	11342726	11343570	845	387	21	59.31	9.21	57572:11309972-11373157:Genebody
chr19	48459181	48459682	502	189	16	62.2	9.21	100170218:48458938-48459058:Downstream
chr19	51223454	51224283	830	332	24	78.14	9.21	-
chr2	152848225	152849053	829	496	22	66.33	9.21	785:152689287-152955235:Genebody
chr3	170038190	170038765	576	380	16	54.32	9.21	-
chr5	17519512	17520037	526	334	24	76.1	9.21	-
chr9	33360305	33361171	867	470	22	63.04	9.21	441394:33219069-33511047:Genebody
chrY	58868597	58869523	927	550	56	100.66	9.21	-
chrY	58872328	58872925	598	334	35	104.34	9.18	-
chr1	47696890	47697486	597	362	19	66.03	9.15	6886:47681962-47695443:Upstream
chr2	60615318	60615887	570	190	18	62.77	9.15	-
chr2	16513541	16514201	661	317	22	72.32	9.09	-
chr2	168920697	168921249	553	197	18	60.23	9.09	27347:168810

								530-16910410 5:Genebody
chr22	16166371	16166881	511	242	19	71.78	9.09	-
chr1	27342762	27343239	478	274	15	61.55	9.07	-
chr1	40688875	40689442	568	374	17	64.96	9.07	6018:4062704 0-40706592:G enebody
chr1	149316631	149317056	426	240	14	60.8	9.07	-
chr1	150463028	150463543	516	243	16	64.02	9.07	80222:150459 919-15047974 7:Genebody
chr1	201543703	201544483	781	350	20	63.05	9.07	-
chr10	37466801	37467390	590	324	20	83.02	9.07	91074:374147 84-37521495: Genebody
chr10	58416366	58417034	669	477	20	73.88	9.07	-
chr10	66019365	66019876	512	211	16	64.48	9.07	-
chr10	67945479	67946133	655	333	16	50.44	9.07	29119:676797 24-69425416: Genebody
chr10	127597216	127597803	588	345	18	69.43	9.07	92565:127585 107-12769815 9:Genebody
chr11	56556434	56556950	517	319	15	57.23	9.07	-
chr11	73698874	73699490	617	248	16	53.75	9.07	-
chr11	76080652	76081279	628	431	19	71.7	9.07	5612:7606100 3-76091880:G enebody

chr11	89640173	89640946	774	381	18	52.18	9.07	-
chr11	109868841	109869377	537	221	18	75.43	9.07	-
chr12	100406804	100407369	566	191	17	65.18	9.07	-
chr13	34927063	34927606	544	372	15	54.47	9.07	-
chr13	96763137	96763640	504	315	14	52.09	9.07	266722:96743092-97491811:Genebody
chr13	110032001	110032457	457	264	16	71.27	9.07	-
chr14	46683511	46684265	755	382	19	59.42	9.07	-
chr14	65940442	65941010	569	199	19	78.54	9.07	2530:65877839-66209961:Genebody
chr15	25988874	25989511	638	274	16	51.89	9.07	57194:25923861-26108349:Genebody
chr15	28690703	28691366	664	475	18	61.62	9.07	-
chr19	8860569	8861047	479	200	14	54.68	9.07	-
chr19	48429961	48430416	456	259	14	57.23	9.07	100170218:48432074-48432194:Upstream
chr2	10906634	10907134	501	324	14	52.39	9.07	245973:10861774-10925228:Genebody
chr2	27389468	27390082	615	213	16	53.93	9.07	-
chr2	28033857	28034459	603	244	17	61.3	9.07	64080:28004265-28113223:Genebody
chr2	111101919	111102445	527	194	20	91.46	9.07	-

chr2	145346316	145346788	473	277	15	62.14	9.07	-
chr20	34303338	34303773	436	246	14	59.57	9.07	9584:34291531-34330193:Genebody
chr20	50639811	50640423	613	430	18	66.73	9.07	-
chr20	61553150	61553789	640	157	19	70.41	9.07	11083:61509089-61569274:Genebody
chr22	45313316	45313741	426	170	14	60.8	9.07	112885:45277044-45405581:Genebody
chr3	153802599	153803033	435	239	15	66.86	9.07	-
chr4	85011245	85011648	404	199	14	63.62	9.07	-
chr4	107012983	107013587	605	270	18	67.58	9.07	93627:106967232-107237423:Genebody
chr4	186351499	186352018	520	199	14	50.51	9.07	441054:186350544-186370821:Genebody
chr5	70079631	70080299	669	270	17	55.1	9.07	-
chr5	164553673	164554297	625	199	18	65.48	9.07	-
chr6	67599107	67599647	541	358	16	61.25	9.07	-
chr6	75468738	75469450	713	362	17	51.4	9.07	-
chr6	95585427	95586099	673	250	18	60.77	9.07	-
chr6	108804937	108805489	553	339	18	73.47	9.07	246269:108616097-108844249:Genebody
chr7_gl00019	165239	165878	640	361	17	57.72	9.07	-

5_random								
chr8	7585347	7585935	589	237	16	56.37	9.07	-
chr8	47705970	47706711	742	397	18	54.73	9.07	-
chr9	4163278	4163733	456	268	16	71.4	9.07	169792:3824127-4300035:Genebody
chr9	67952546	67953327	782	473	24	88.05	9.07	84210:67926760-67969839:Genebody
chrY	22268649	22269279	631	433	20	78.1	9.07	-
chrY	28805352	28805989	638	360	16	51.89	9.07	-
chr10	94896874	94897495	622	432	17	59.16	9.03	-
chr11	12478001	12478611	611	290	16	54.05	9.03	55742:12399145-12551410:Genebody
chr11	18454018	18454678	661	469	21	81.12	9.03	3948:18433852-18472792:Genebody
chr19	12342446	12343043	598	203	19	74.77	9.03	-
chr19	14941645	14942114	470	189	14	55.43	9.03	-
chr2	16857173	16857659	487	297	15	60.27	9.03	-
chr22	22690644	22691389	746	274	20	65.9	9.03	-
chr4	36583878	36584506	629	433	20	78.01	9.03	-
chr4	43398949	43399508	560	197	16	59	9.03	-
chr4	142555781	142556297	517	263	16	63.65	9.03	3600:142557753-142654611:Upstream
chr8	2712792	2713259	468	240	16	69.57	9.03	-

chr9	9711409	9712036	628	192	17	58.59	9.03	-
chr1	150140110	150140778	669	451	23	67.55	9	-
chr15	79210018	79210756	739	315	22	54.86	9	-
chrUn_gl000 225	6532	7278	747	360	23	59.26	9	-
chr1	28815393	28816238	846	384	23	68.6	8.97	65979:28696092-28826880: Genebody
chr12	40221311	40222109	799	480	23	73.05	8.97	114134:40148824-40499661 :Genebody
chr15	65780997	65781450	454	257	15	59.72	8.97	54878:65737998-65809609: Genebody
chr18	18510909	18511642	734	434	23	79.81	8.97	-
chr2	107913340	107914030	691	253	21	71.83	8.97	-
chr4	188271013	188271606	594	253	16	51.17	8.97	-
chr5	133736743	133737627	885	459	21	54.38	8.97	91368:133737757-133747598:Downstrea m
chr7	9802729	9803183	455	200	17	73.57	8.97	-
chr12	76401064	76401480	417	199	16	60.33	8.93	-
chr2	43761363	43762043	681	391	18	50.35	8.93	63892:43457975-43823113: Genebody
chr2	110915698	110916499	802	397	21	56.03	8.93	4867:110880915-110962639

								:Genebody
chr3	195217548	195218813	1266	784	90	221.56	8.89	-
chr5	144142033	144142530	498	252	18	65.45	8.89	-
chr17	6735673	6736447	775	291	24	64.88	8.85	83659:6703300-6735060:Upstream
chr22	16229570	16230289	720	432	21	54.29	8.85	-
chr14	47880241	47880932	692	447	18	57.05	8.78	161357:47308829-48143988:Genebody
chr15	64848467	64849219	753	398	20	63.37	8.78	23060:64791618-64978264:Genebody
chr19	1776954	1777682	729	357	19	59.63	8.78	390874:1753661-1775443:Downstream
chr21	39259403	39259943	541	336	16	59.41	8.78	3763:38996785-39288696:Genebody
chr3	64798769	64799581	813	380	19	52.69	8.78	693126:64705682-64941858:Genebody
chr3	170643167	170643684	518	193	16	61.93	8.78	-
chr5	112739005	112739671	667	317	18	59.34	8.78	4163:112357796-112824527:Genebody
chr5	127709956	127710617	662	467	18	59.81	8.78	2201:127593601-127873735

								:Genebody
chr5	152415423	152416096	674	209	17	52.81	8.78	-
chr7	114277737	114278384	648	326	17	55.1	8.78	93986:113726364-114333825:Genebody
chr1	198209992	198210782	791	544	22	66.01	8.75	140609:198126107-198291546:Genebody
chr1	229356207	229356939	733	298	20	59.91	8.75	-
chr1	229848006	229848498	493	322	16	60.27	8.75	-
chr10	134962805	134963328	524	167	15	50.62	8.75	-
chr15	97764800	97765197	398	200	14	58.34	8.75	-
chr18	44540431	44540827	397	199	14	58.34	8.75	83473:44526786-44628613:Genebody
chr3	49640703	49641427	725	200	26	98.44	8.75	8927:49591921-49708981:Genebody
chr3	88318004	88318683	680	483	21	71.15	8.75	-
chr3	111202442	111202991	550	353	18	66.64	8.75	-
chr4	6762054	6762862	809	421	21	58.78	8.75	-
chr4	48429147	48429754	608	320	20	72.87	8.75	57606:48343612-48428213:Downstream
chr4	146159315	146159888	574	215	20	77	8.75	-
chr6	11491156	11491722	567	374	18	64.68	8.75	-
chr6	120144084	120144726	643	258	18	56.78	8.75	-
chr8	71756473	71757310	838	415	20	51.12	8.75	-

chrY	13719052	13719781	730	417	44	129.89	8.75	-
chr1	73939392	73939837	446	194	16	61.89	8.72	-
chr6	73310102	73310768	667	271	19	55.69	8.72	-
chr1	231326	231915	590	217	18	53.49	8.7	-
chr7	87767604	87768377	774	521	29	91.98	8.67	53616:87563701-87811428:Genebody
chr9	46891374	46892131	758	449	27	62.88	8.61	-
chrY	58832692	58834380	1689	1320	75	100.75	8.59	-
chrY	58912166	58912878	713	311	76	178.06	8.59	-
chr8	86753193	86754403	1211	723	61	92.57	8.58	-
chr1	58767530	58768104	575	200	16	54.42	8.55	-
chr1	149894768	149895351	584	392	16	53.55	8.55	10262:149895210-149899702:Downstream
chr11	36899219	36899803	585	386	16	53.46	8.55	-
chr13	113527191	113527698	508	313	16	61.51	8.55	23250:113344642-113541480:Genebody
chr15	88976203	88976626	424	185	14	57.97	8.55	-
chr16	63017243	63017838	596	424	16	52.42	8.55	-
chr16	84384807	84385244	438	245	14	56.28	8.55	-
chr16	89276420	89276987	568	190	16	55.11	8.55	-
chr18	31941767	31942274	508	195	15	55.01	8.55	-
chr18	50638496	50639232	737	314	18	51.61	8.55	1630:49866541-51062273:Genebody

chr22	24271208	24271835	628	353	17	55.38	8.55	-
chr5	123848574	123849362	789	290	20	58.41	8.55	-
chr7	34408920	34409333	414	219	14	52.37	8.55	100329167:34386125-34797884:Genebody
chr9	138582782	138583524	743	433	21	68.43	8.55	-
chr1	205331253	205331859	607	270	19	64.92	8.54	-
chr12	58921159	58921573	415	219	15	61.93	8.54	-
chr14	95518675	95519567	893	444	21	50.45	8.54	-
chr20	62167944	62168708	765	498	23	72.54	8.54	5753:62159777-62168707:Genebody
chr4	20020865	20021419	555	340	19	70.96	8.54	-
chr6	154456230	154456888	659	220	21	71.64	8.54	4988:154360442-154568001:Genebody
chr7	44286038	44286653	616	284	20	63.94	8.54	816:44256748-44365230:Genebody
chr8	45611	46533	923	586	29	86.17	8.54	-
chr4	15407	15957	551	359	21	79.71	8.52	-
chr6	36403305	36403854	550	322	22	86.66	8.52	222659:36358328-36410666:Genebody
chr9	25844793	25845265	473	275	18	70.25	8.52	-
chr14	21651541	21652066	526	200	18	59.23	8.51	-
chr4	21333834	21334447	614	187	22	72.91	8.51	80333:207302

								38-21546279:<br>Genebody
chr4	190154687	190155651	965	475	39	51.95	8.51	-
chr8	43827677	43828504	828	390	68	110.32	8.49	-
chrY	58883206	58885405	2200	462	243	232.92	8.49	-
chr13	111358978	111359827	850	290	38	121.6	8.48	79587:111293<br>756-11135848<br>0:Upstream
chr2	162137683	162139038	1356	473	47	71.82	8.45	-
chr3	74707988	74709009	1022	506	37	66.77	8.45	-
chr1	1232178	1232671	494	198	14	53.11	8.37	116983:12277<br>63-1243269:G<br>enebody
chr1	50265261	50265821	561	365	17	65.72	8.37	84871:489985<br>26-50489626:<br>Genebody
chr1	91579541	91580069	529	349	16	62.56	8.37	-
chr1	100093480	100093914	435	198	14	59.7	8.37	-
chr1	101000693	101001181	489	189	14	53.63	8.37	-
chr1	102192784	102193577	794	380	21	67.86	8.37	-
chr1	104864072	104864676	605	197	16	54.85	8.37	-
chr1	145330179	145330751	573	296	15	51.7	8.37	100132406:14<br>5293370-1453<br>68682:Geneb<br>ody
chr1	150240007	150240507	501	226	16	58.95	8.37	51107:150237<br>798-15024153<br>2:Genebody

chr1	246946589	246947208	620	298	18	66	8.37	-
chr10	12529222	12529726	505	351	14	51.99	8.37	57118:12391582-12868135:Genebody
chr10	31421574	31422403	830	334	20	58.94	8.37	-
chr10	53274828	53275426	599	203	16	55.41	8.37	5592:52750944-54055274:Genebody
chr10	58317351	58318107	757	427	20	65.19	8.37	-
chr10	59327812	59328363	552	314	16	60.09	8.37	-
chr10	64788003	64788594	592	214	16	56.08	8.37	-
chr10	77662567	77663056	490	221	14	53.52	8.37	83938:77542518-78317124:Genebody
chr10	99788340	99788848	509	330	14	51.59	8.37	55118:99624757-99790585:Genebody
chr10	108534302	108534802	501	314	15	58.95	8.37	114815:108333421-108924292:Genebody
chr10	127783320	127784044	725	326	20	68.19	8.37	8038:127702901-128077127:Genebody
chr10	135389440	135390019	580	384	16	57.24	8.37	-
chr11	14456002	14456477	476	299	14	55.01	8.37	-
chr11	44192387	44192993	607	332	17	60.9	8.37	2132:44117098-44266979:Genebody

chr11	100521553	100522010	458	256	14	57	8.37	-
chr11	107713374	107714021	648	235	18	63.17	8.37	54733:107661719-107729914:Genebody
chr12	12443248	12444012	765	323	19	58.57	8.37	-
chr12	34325286	34325880	595	200	17	62.11	8.37	-
chr12	49042821	49043392	572	213	16	58.04	8.37	-
chr12	51613448	51614153	706	332	18	57.78	8.37	-
chr12	55964857	55965492	636	254	20	77.53	8.37	-
chr12	56818481	56818971	491	271	14	53.42	8.37	8914:56810156-56843200:Genebody
chr12	89320230	89320829	600	325	18	68.12	8.37	-
chr12	98767281	98767868	588	395	16	56.46	8.37	-
chr12	120310310	120311009	700	408	18	58.31	8.37	11113:120123597-120315092:Genebody
chr13	48012984	48013560	577	188	18	70.67	8.37	-
chr13	75292985	75293481	497	279	18	80.65	8.37	-
chr13	85218337	85219023	687	271	17	53.55	8.37	-
chr13	88284094	88284646	553	219	15	53.59	8.37	-
chr13	93398716	93399532	817	354	20	59.99	8.37	2262:92050934-93519485:Genebody
chr14	47542417	47543132	716	265	18	56.92	8.37	161357:47308829-47812438:Genebody
chr14	87632513	87632929	417	231	14	61.93	8.37	-

chr15	25031138	25031704	567	353	15	52.25	8.37	-
chr15	28585640	28586175	536	246	15	55.27	8.37	-
chr15	30539584	30540051	468	183	14	55.88	8.37	-
chr15	57866675	57867144	470	194	14	55.66	8.37	-
chr15	101642824	101643466	643	304	17	57.44	8.37	-
chr16	54202115	54202623	509	311	14	51.59	8.37	-
chr16	67719023	67719534	512	291	16	64.48	8.37	81577:67708436-67753273:Genebody
chr16	68682760	68683310	551	197	16	60.19	8.37	1001:68678150-68732957:Genebody
chr16	71482347	71482833	487	241	14	53.84	8.37	7571:71481512-71496117:Genebody
chr17	58102345	58102989	645	246	16	51.28	8.37	-
chr18	39191975	39192417	443	200	14	58.74	8.37	-
chr18	39404455	39405007	553	181	16	59.98	8.37	-
chr18	52340673	52341315	643	200	18	63.66	8.37	-
chr18	66802473	66802975	503	319	14	52.19	8.37	-
chr19	2524130	2524756	627	269	18	65.27	8.37	2788:2511217-2702746:Genebody
chr19	15922620	15923110	491	315	14	53.42	8.37	-
chr19	37784158	37784759	602	184	16	55.13	8.37	-
chr19	42056572	42057123	552	368	16	60.09	8.37	-
chr19	44017054	44017564	511	314	14	51.39	8.37	23474:44010871-44031396:

								Genebody
chr19	58677753	58678630	878	391	22	66.41	8.37	-
chr2	618381	618949	569	310	18	71.59	8.37	-
chr2	14315856	14316350	495	302	15	59.61	8.37	-
chr2	50599934	50600402	469	190	14	55.77	8.37	9378:50145643-51259674:Genebody
chr2	62241908	62242482	575	389	17	64.2	8.37	150684:62132802-62363204:Genebody
chr2	68293320	68293875	556	185	15	53.3	8.37	-
chr2	102083402	102083937	536	197	15	55.27	8.37	731220:102013822-102091165:Genebody
chr2	122251622	122252146	525	326	16	63	8.37	23332:122095353-122407052:Genebody
chr2	149019942	149020398	457	242	15	57.11	8.37	55777:148778579-149271042:Genebody
chr2	153583699	153584305	607	421	16	54.67	8.37	151188:153574422-153617767:Genebody
chr2	174235806	174236469	664	466	17	55.54	8.37	-
chr2	177133424	177133908	485	294	14	54.05	8.37	10651:177134122-177202751:Upstream
chr2	190149125	190149810	686	385	18	59.57	8.37	-

chr20	42721825	42722377	553	363	15	53.59	8.37	-
chr21	30945136	30945825	690	438	18	59.21	8.37	2897:30909255-31312282:Genebody
chr21	42040570	42041117	548	350	17	67.18	8.37	1826:41384342-42219039:Genebody
chr22	18667422	18668260	839	265	26	95.07	8.37	-
chr22	18737207	18737660	454	188	14	57.46	8.37	-
chr22	35246019	35246672	654	334	19	68.94	8.37	-
chr22	39951880	39952426	547	193	15	54.17	8.37	-
chr3	46065768	46066382	615	200	16	53.93	8.37	2829:46062291-46068979:Genebody
chr3	50269710	50270316	607	411	16	54.67	8.37	-
chr3	138147689	138148250	562	375	15	52.73	8.37	-
chr4	9271658	9272241	584	320	15	50.69	8.37	391627:9269344-9270936:Downstream
chr4	15234191	15234805	615	423	16	53.93	8.37	-
chr4	54934452	54934921	470	185	15	55.66	8.37	-
chr4	60705122	60705698	577	193	15	51.33	8.37	-
chr4	95015177	95015774	598	318	17	61.81	8.37	-
chr4	146567266	146567885	620	189	19	72.58	8.37	166785:146540539-146581186:Genebody
chr5	5732606	5733333	728	261	20	67.91	8.37	-
chr5	32768512	32769023	512	316	14	51.29	8.37	4883:3271166

								4-32787252:G enebody
chr5	34799170	34799628	459	263	14	56.89	8.37	26064:346564 32-34832716: Genebody
chr5	51240662	51241215	554	207	16	59.88	8.37	-
chr5	54002155	54002722	568	271	16	58.44	8.37	-
chr5	122287621	122288369	749	359	18	54.16	8.37	28966:122181 159-12234490 1:Genebody
chr5	137188495	137188958	464	278	14	56.33	8.37	-
chr5	138379490	138379978	489	192	14	53.63	8.37	64374:138282 411-13853406 5:Genebody
chr5	143679973	143680430	458	194	14	57	8.37	57528:143550 436-14385694 4:Genebody
chr6	64903459	64903941	483	291	16	67.94	8.37	346007:64429 875-66417118 :Genebody
chr6	131733368	131733951	584	389	15	50.69	8.37	-
chr7	6003561	6004179	619	212	17	59.72	8.37	222967:59657 76-6010314:G enebody
chr7	42931411	42932022	612	356	16	54.21	8.37	-
chr7	98398708	98399189	482	171	14	54.36	8.37	-
chr7	102165146	102165615	470	286	14	55.66	8.37	-
chr7	102294046	102294599	554	299	16	59.88	8.37	246721:10227

								7471-1023121<br>82:Genebody
chr7	124069950	124070463	514	200	15	57.54	8.37	-
chr7	125771499	125772295	797	479	22	73.69	8.37	-
chr7	141496775	141497240	466	271	14	56.1	8.37	-
chr7	145319609	145320334	726	302	17	50.36	8.37	-
chr8	5212892	5213379	488	195	14	53.73	8.37	-
chr8	8052460	8053091	632	264	18	64.76	8.37	-
chr8	9716199	9716721	523	349	14	50.22	8.37	-
chr8	12050201	12050845	645	191	18	63.46	8.37	85002:120396<br>13-12051624:<br>Genebody
chr8	33255028	33255587	560	377	15	52.92	8.37	84750:332283<br>45-33330664:<br>Genebody
chr8	74378525	74379025	501	310	14	52.39	8.37	27067:743326<br>05-74659162:<br>Genebody
chr8	90887806	90888393	588	394	16	56.46	8.37	-
chr8	122868894	122869600	707	297	18	57.7	8.37	-
chr8	123220544	123220979	436	248	14	59.57	8.37	-
chr8	130506592	130507158	567	376	15	52.25	8.37	-
chr9	17782486	17783112	627	242	16	52.85	8.37	6456:1757895<br>2-17797120:G<br>enebody
chr9	28724014	28724613	600	189	16	55.32	8.37	-
chr9	36848377	36849015	639	443	16	51.8	8.37	5079:3683853<br>0-37034476:G

								enebody
chr9	40092255	40092733	479	236	14	54.68	8.37	-
chr9	69777611	69778046	436	200	14	59.57	8.37	-
chr9	106621063	106621743	681	365	17	54.06	8.37	-
chr9	110030509	110031009	501	194	14	52.39	8.37	-
chrUn_gl000221	73436	73909	474	191	14	55.22	8.37	-
chrY	10095008	10095652	645	261	17	57.26	8.37	-
chrY	58898106	58898619	514	246	17	71.19	8.37	-
chr1	9537424	9538437	1014	671	41	113.9	8.33	-
chr1	29472414	29473084	671	431	20	53.7	8.33	NM_005626:29474250-29508637:Downstream
chr1	69732701	69733336	636	234	17	53.12	8.33	-
chr1	97490523	97491093	571	382	17	59.49	8.33	-
chr1	143461732	143462401	670	366	18	60.77	8.33	-
chr1	170205769	170206263	495	186	16	66.21	8.33	-
chr1	186571542	186572106	565	195	16	58.49	8.33	-
chr10	80551078	80551955	878	410	24	60.25	8.33	-
chr10	135256934	135257628	695	404	20	55.62	8.33	-
chr11	72782615	72783217	603	193	17	61.03	8.33	9873:72547790-72853143:Genebody
chr11	111819467	111819939	473	263	16	68.93	8.33	85458:111807926-111893305:Genebody
chr11	127210742	127211597	856	410	22	56.78	8.33	-

chr12	118631558	118632153	596	216	16	55.45	8.33	51347:118587606-118810750:Genebody
chr12	131421195	131421930	736	379	21	57.18	8.33	-
chr13	36597279	36597796	518	188	14	50.48	8.33	9201:36343122-36705464:Genebody
chr13	41179946	41180386	441	258	14	58.74	8.33	2308:41129802-41240734:Genebody
chr13	60871342	60872014	673	326	21	63.41	8.33	-
chr15	20066658	20067430	773	477	22	69.61	8.33	-
chr15	42218903	42219511	609	413	16	54.23	8.33	30844:42191640-42264755:Genebody
chr15	73135664	73136151	488	194	15	60.16	8.33	-
chr17	7718735	7719354	620	312	16	53.23	8.33	146754:7623038-7737058:Genebody
chr17	29164521	29165286	766	393	21	59.18	8.33	79915:29159022-29222295:Genebody
chr17	46693866	46694343	478	197	14	54.56	8.33	3218:46689708-46692301:Upstream
chr17	72933153	72933676	524	198	16	58.22	8.33	347741:72931896-72945510:Genebody

chr17	75330774	75331668	895	466	26	75.13	8.33	10801:75277491-75496676:Genebody
chr18	25724889	25725737	849	422	22	57.36	8.33	1000:25530929-25757445:Genebody
chr19	52690337	52690887	551	383	16	59.93	8.33	-
chr2	6030276	6031167	892	360	26	81.9	8.33	-
chr2	33327717	33328712	996	472	30	81.4	8.33	4052:33172368-33624573:Genebody
chr2	26825152	26825745	594	195	16	51.17	8.33	130106:26804073-26864211:Genebody
chr2	56722462	56723275	814	360	21	60.11	8.33	-
chr2	77277410	77277888	479	171	14	54.46	8.33	80059:76974857-77749502:Genebody
chr2	83015277	83016014	738	198	21	72.87	8.33	-
chr2	133391987	133392537	551	326	17	57.2	8.33	2863:133174146-133404168:Genebody
chr2	138063129	138063938	810	470	26	72.24	8.33	80731:137748461-138435287:Genebody
chr2	167057509	167058088	580	360	17	54.17	8.33	6335:167051703-167232497:Genebody

chr20	34206050	34206649	600	330	16	55.07	8.33	6676:34203808-34208965:Genebody
chr21	33415439	33416496	1058	479	43	105.69	8.33	-
chr3	15318032	15318558	527	187	16	57.89	8.33	9467:15296356-15374104:Genebody
chr3	99583565	99584321	757	290	22	65.65	8.33	100313938:99273152-99717059:Genebody
chr3	115172855	115173299	445	249	14	58.27	8.33	-
chr3	139878493	139879200	708	299	19	58.12	8.33	64084:139654026-140286917:Genebody
chr3	150415682	150416300	619	192	18	65.81	8.33	131831:150377676-150421742:Genebody
chr4	1632700	1633341	642	387	16	51.29	8.33	-
chr4	22224161	22224638	478	241	17	65.82	8.33	-
chr4	107242738	107243511	774	456	22	69.51	8.33	9255:107236766-107270379:Genebody
chr4	119901206	119901622	417	222	16	56.54	8.33	171024:119809995-119959668:Genebody
chr4	179779735	179780350	616	183	18	52.22	8.33	-
chr4	184757525	184758194	670	481	18	55.8	8.33	-

chr5	21901787	21902527	741	422	22	67.23	8.33	1010:21750972-22853731:Genebody
chr5	64963101	64963623	523	224	14	50	8.33	80006:64920557-64961953:Downstream
chr5	88424073	88424629	557	234	16	59.31	8.33	-
chr5	159385727	159386256	530	187	15	51.35	8.33	147:159343739-159400017:Genebody
chr5	159415156	159415906	751	324	20	65.44	8.33	-
chr6	3595788	3596592	805	477	23	66.73	8.33	-
chr6	12810716	12811289	574	264	15	51.37	8.33	221692:12717832-13287528:Genebody
chr6	84408056	84408626	571	383	17	64.36	8.33	9892:84262606-84419127:Genebody
chr6	91306932	91307406	475	231	17	61.97	8.33	-
chr6	94205044	94205758	715	478	22	69.9	8.33	-
chr7	2919126	2919697	572	295	15	51.55	8.33	-
chr7	93975266	93975723	458	272	14	56.77	8.33	-
chr7	108927337	108928006	670	408	18	55.8	8.33	-
chr7	139305313	139305852	540	345	20	83.32	8.33	28996:139246316-139477693:Genebody
chr7	142894958	142895536	579	218	16	57.09	8.33	-
chr8	2772707	2773224	518	194	15	56.88	8.33	-

chr8	25243070	25243832	763	301	19	53.37	8.33	80005:25042286-25270618:Genebody
chr8	43830829	43832182	1354	385	100	154.53	8.33	-
chr8	52931262	52931744	483	244	15	60.73	8.33	-
chr8	53377495	53378228	734	315	19	55.81	8.33	-
chr8	63885147	63885693	547	331	16	55.77	8.33	286183:63161500-63903627:Genebody
chr8	72853999	72854497	499	222	15	58.93	8.33	100132891:72755357-72968546:Genebody
chr9	66583254	66583834	581	382	15	50.73	8.33	-
chr9	76995393	76995758	366	184	14	62.64	8.33	-
chr9	105464578	105465167	590	394	16	51.54	8.33	-
chr9	137382069	137382923	855	429	20	56.67	8.33	-
chrUn_gl000225	99080	99551	472	275	23	76.76	8.33	-
chr11	2551485	2552232	748	473	25	58.11	8.19	3784:2466220-2870339:Genebody
chr10	42596739	42598531	1793	327	369	522.21	8.17	-
chr7	49477655	49478603	949	519	34	93.63	8.17	-
chr11	122441731	122442336	606	250	19	53.5	8.16	-
chr17	36319734	36320377	644	193	22	66.2	8.16	-
chr5	20094779	20095367	589	335	24	85.56	8.16	-
chr7	152103115	152103511	397	199	16	60.63	8.16	58508:151832

								011-15213309<br>0:Genebody
chr9	140222366	140222990	625	461	20	57	8.16	54932:140201<br>347-14031771<br>4:Genebody
chr6	140809054	140809783	730	257	20	51.01	8.15	-
chr7	784994	785578	585	226	22	78.29	8.15	54919:766337<br>-826116:Gene<br>body
chr1	121478371	121479531	1161	643	68	170.36	8.14	-
chr10	66070537	66071180	644	358	20	63.79	8.14	-
chr10	125152237	125153079	843	452	23	61.47	8.14	-
chr13	32523389	32523821	433	193	15	50.56	8.14	196549:32420<br>919-32533720<br>:Genebody
chr14	23598740	23599264	525	190	18	64.98	8.14	23428:235945<br>05-23623610:<br>Genebody
chr14	64516661	64517483	823	482	24	68.81	8.14	23224:643196<br>82-64693165:<br>Genebody
chr14	69313034	69313647	614	194	18	55.17	8.14	-
chr15	89907252	89908100	849	501	22	55.71	8.14	-
chr4	76699119	76699543	425	186	15	57.98	8.14	8615:7664982<br>8-76735364:G<br>enebody
chr1	215900148	215900730	583	219	16	50.8	8.13	7399:2157962<br>35-216596738

								:Genebody
chr1	172524084	172524686	603	330	18	60.77	8.12	51430:172502259-172580971:Genebody
chr11	26625669	26626188	520	321	16	57.21	8.12	63982:26353677-26684835:Genebody
chr12	2364887	2365627	741	208	34	78	8.12	775:2162415-2807115:Genebody
chr13	20375134	20375633	500	304	18	72.87	8.12	-
chr14	24742629	24743434	806	413	20	53.63	8.12	-
chr15	24260104	24260854	751	310	21	63.99	8.12	-
chr15	99961932	99962479	548	205	19	73.52	8.12	-
chr17	21532305	21532988	684	359	20	64.61	8.12	-
chr18	27206161	27206907	747	395	21	64.37	8.12	-
chr2	4971459	4972079	621	296	18	58.93	8.12	-
chr2	10507909	10508357	449	253	14	52.38	8.12	3241:10443039-10567742:Genebody
chr2	138619075	138619522	448	199	14	52.49	8.12	-
chr20	11602979	11603661	683	326	19	58.8	8.12	-
chr4	88606054	88606577	524	263	17	63.17	8.12	-
chr4	117423256	117423695	440	251	15	53.41	8.12	-
chr7	111636634	111637419	786	375	21	60.78	8.12	9732:111366164-111846462:Genebody
chr11	19040576	19041089	514	210	16	62.38	8.11	-

chr11	48087014	48087865	852	421	20	55.1	8.11	5795:48002109-48154264:Genebody
chr11	58644689	58645499	811	496	21	64.06	8.11	-
chr12	94015248	94016138	891	391	25	80.52	8.11	-
chr13	85939000	85939657	658	185	17	54.21	8.11	-
chr18	11247497	11247979	483	300	15	59.21	8.11	-
chr18	74500110	74500671	562	192	15	51.04	8.11	-
chr2	171563175	171563940	766	380	20	62.2	8.11	-
chr2	206663604	206664058	455	170	14	55.69	8.11	8828:206547223-206662856:Downstream
chr20	49993103	49993629	527	190	18	74.56	8.11	-
chr3	25845722	25846307	586	390	17	61.11	8.11	-
chr3	43029945	43030560	616	194	17	58.1	8.11	729085:43020758-43099205:Genebody
chr3	113850606	113851346	741	274	22	76.85	8.11	1814:113847556-113897899:Genebody
chr3	151173714	151174448	735	417	20	65.03	8.11	285313:151153779-151176497:Genebody
chr5	37336793	37337277	485	222	15	58.98	8.11	9631:37291940-37370887:Genebody
chr5	105416190	105416761	572	173	16	56.22	8.11	-
chr5	115670135	115670873	739	266	18	53.04	8.11	-

chr5	155290456	155291198	743	295	18	52.72	8.11	-
chr5	167154365	167154887	523	170	15	54.87	8.11	57451:166711842-167691161:Genebody
chr7	67617478	67618067	590	411	16	54.47	8.11	-
chr8	18431971	18432421	451	273	14	56.15	8.11	23362:18384813-18666405:Genebody
chr8	41339990	41340672	683	428	19	63.9	8.11	-
chr8	128131718	128132167	450	236	14	56.26	8.11	-
chr8	146161265	146161825	561	365	15	51.13	8.11	7564:146155745-146176274:Genebody
chr9	8716132	8716685	554	191	16	58.05	8.11	5789:8314246-10612723:Genebody
chr8	83420247	83421259	1013	516	31	69.26	8.02	-
chr14	34996636	34997170	535	294	21	72.37	8	55837:34985134-35008943:Genebody
chr2	92296949	92298231	1283	523	65	112.08	7.99	-
chr1	56133277	56133670	394	198	15	56.44	7.98	-
chr10	94080278	94081026	749	446	22	58.27	7.98	54708:94050919-94113721:Genebody
chr14	56697539	56698058	520	199	19	66.13	7.98	57161:56585092-56768030:Genebody

chr15	77961857	77962490	634	460	19	53.28	7.98	-
chr20	44682319	44682848	530	323	17	52.87	7.98	57468:44650328-44688789:Genebody
chr7	102364979	102365507	529	192	17	52.98	7.98	-
chr10	6263199	6263692	494	187	17	60.95	7.95	5209:6186842-6277505:Genebody
chr11	33978893	33979678	786	382	21	54.25	7.95	-
chr13	62577518	62578170	653	456	20	61.27	7.95	-
chr14	67685237	67686107	871	436	24	62.58	7.95	161142:67656145-67695267:Genebody
chr16	66118813	66119448	636	442	19	57.26	7.95	-
chr16	68493955	68494698	744	418	20	52.64	7.95	-
chr17	66643248	66643716	469	235	16	57.66	7.95	-
chr6	55507585	55508154	570	364	18	58.33	7.95	-
chr6	95381980	95382573	594	194	18	55.79	7.95	-
chr12	44418574	44419307	734	335	20	58.16	7.93	84216:44229961-44783540:Genebody
chr14	72010727	72011302	576	173	17	55.99	7.93	26037:71996041-72206118:Genebody
chr15	28687632	28688120	489	196	17	65.9	7.93	-
chr15	52659015	52659836	822	369	21	55.99	7.93	4644:52599479-52821247:Genebody

chr19	35502789	35503403	615	185	17	52.17	7.93	57655:35491245-35517373:Genebody
chr19	42773978	42774568	591	265	17	54.48	7.93	-
chr2	50786334	50787109	776	287	21	59.95	7.93	9378:50145643-51259674:Genebody
chr2	132273348	132273860	513	222	16	56.58	7.93	150776:132250385-132279149:Genebody
chr2	187485183	187485822	640	231	18	55.55	7.93	3685:187454789-187545627:Genebody
chr21	29921413	29922220	808	588	26	86.25	7.93	-
chr21	30232000	30232538	539	352	16	53.79	7.93	-
chr4	76799256	76800004	749	380	21	62.43	7.93	5470:76781027-76823681:Genebody
chr4	83761599	83762347	749	303	25	86.66	7.93	22872:83739814-83812400:Genebody
chr7	29834196	29835010	815	409	22	62.01	7.93	-
chr7	124887287	124887964	678	268	20	63.52	7.93	-
chr8	97647911	97648799	889	402	23	61.06	7.93	-
chr9	1014124	1014553	430	196	15	59.96	7.93	-
chr9	19236260	19236985	726	389	20	58.89	7.93	-
chr9	103711665	103712244	580	199	19	67.97	7.93	-
chr9	141123430	141123903	474	189	16	54.64	7.93	100132403:14

								1106636-141134172:Genebody
chr9_gl000199_random	83956	85150	1195	435	157	291.45	7.93	-
chr1	42963595	42964117	523	280	16	59.82	7.89	-
chr1	154291526	154292340	815	370	20	56.26	7.89	89872:154293591-154297800:Upstream
chr10	35419222	35419644	423	226	14	58.09	7.89	1390:35415800-35468774:Genebody
chr10	93688682	93689260	579	282	18	66.63	7.89	9044:93683735-93790078:Genebody
chr10	134403328	134403910	583	192	16	53.65	7.89	3632:134351352-134596983:Genebody
chr11	82887186	82887803	618	356	16	50.42	7.89	51585:82868136-82896833:Genebody
chr12	103679258	103679718	461	263	14	53.65	7.89	-
chr12	111102294	111102827	534	311	15	52.33	7.89	84329:111086490-111126946:Genebody
chr12	117606713	117607346	634	330	17	54.82	7.89	23014:117581585-117628300:Genebody

chr13	88107718	88108434	717	350	18	53.26	7.89	-
chr14	62124664	62125244	581	407	16	53.84	7.89	-
chr15	99173970	99174456	487	295	14	50.87	7.89	-
chr17	47922328	47922925	598	182	18	64.56	7.89	255061:47915670-47925379:Genebody
chr2	144127665	144128337	673	246	22	82.28	7.89	55843:143886898-144525920:Genebody
chr20	52765386	52765884	499	240	15	55.97	7.89	-
chr21	16421625	16422143	519	194	16	60.27	7.89	8204:16333555-16437126:Genebody
chr3	80111672	80112298	627	216	18	61.55	7.89	-
chr4	29069865	29070326	462	269	14	53.54	7.89	-
chr4	129945081	129945747	667	349	18	57.68	7.89	132320:129805151-130014764:Genebody
chr4	186329417	186329932	516	320	16	60.6	7.89	55325:186320693-186347139:Genebody
chr5	15925513	15926212	700	416	18	54.71	7.89	23194:15500304-15939900:Genebody
chr5	62303068	62303559	492	200	14	50.35	7.89	-
chr5	94726446	94727130	685	424	18	56.04	7.89	153643:94727047-94786144:Upstream

chr6	35340857	35341395	539	274	16	58.09	7.89	5467:35310334-35393171:Genebody
chr6	67846148	67846626	479	273	14	51.7	7.89	-
chr6	83506180	83506828	649	457	18	59.38	7.89	-
chr6	91139197	91139797	601	316	19	70.71	7.89	-
chr7	93920765	93921373	609	195	17	57.2	7.89	-
chr8	134638814	134639687	874	403	22	62.45	7.89	-
chr15	87825116	87825817	702	338	20	50.79	7.81	-
chr1_gl000192_random	527808	528540	733	284	22	58.31	7.81	-
chr3	133442513	133443147	635	253	20	57.32	7.81	-
chr3	181428827	181429247	421	222	16	58.86	7.81	347689:181328150-181459003:Genebody
chr7	153789975	153790553	579	356	18	52.06	7.81	1804:153584418-154685994:Genebody
chr8	43838271	43838802	532	312	53	121.2	7.81	-
chr10	109331258	109331912	655	194	20	59.55	7.78	-
chr13	64688196	64688750	555	193	18	58.6	7.78	-
chr13	95742248	95743020	773	320	22	59.12	7.78	10257:95672083-95953687:Genebody
chr17	5647142	5647704	563	387	17	51.89	7.78	-
chr17	61871431	61871928	498	290	17	59.11	7.78	11325:61851566-61896676:Genebody

chr19	7681114	7681716	603	354	18	53.51	7.78	57662:7660787-7683194:Genebody
chr2	49314341	49314859	519	321	17	56.65	7.78	2492:49189295-49381666:Genebody
chr3	184192661	184193255	595	199	19	60.11	7.78	-
chr6	75696643	75697178	536	300	17	54.75	7.78	-
chr8	125875688	125876546	859	528	24	61.92	7.78	-
chr9	41960372	41960975	604	261	19	59.14	7.78	654466:41958802-42019584:Genebody
chr9	105946290	105946858	569	226	18	57.05	7.78	-
chr1	223958269	223958815	547	309	20	76.96	7.74	824:223889294-223963718:Genebody
chr10	182500	183207	708	300	27	102.87	7.74	10771:180423-295199:Genebody
chr12	6205493	6206101	609	281	18	57.12	7.74	7450:6058039-6233836:Genebody
chr12	65867910	65868639	730	292	19	51.5	7.74	-
chr14	100084281	100084873	593	311	17	52.88	7.74	-
chr16	81034336	81034877	542	346	16	52.13	7.74	56942:81009700-81040502:Genebody
chr2	130277901	130278459	559	296	16	50.43	7.74	-

chr2	187027989	187028616	628	438	18	55.23	7.74	-
chr20	33311289	33312021	733	291	22	68.04	7.74	23054:33302578-33413433:Genebody
chr20	59843678	59844367	690	380	19	55.03	7.74	1002:59827558-60512299:Genebody
chr3	86034190	86034709	520	246	16	54.45	7.74	253559:85008132-86123577:Genebody
chr3	154631553	154632131	579	405	22	86.27	7.74	-
chr4	27189945	27190449	505	190	18	69.01	7.74	-
chr4	60421062	60421856	795	350	20	51.37	7.74	-
chr5	9863510	9864255	746	371	20	55.5	7.74	285692:9641427-9903936:Genebody
chr5	102470733	102471350	618	196	17	50.5	7.74	23262:102465256-102538907:Genebody
chr8	102770367	102770976	610	217	17	51.25	7.74	83988:102698770-102803439:Genebody
chr8	131819154	131819772	619	426	17	50.41	7.74	114:131792547-132052835:Genebody
chr9	4261240	4261700	461	241	16	61.34	7.74	-
chr9	29553689	29554373	685	406	18	50	7.74	-
chr7	61098883	61100611	1729	1106	94	170.1	7.73	-

chr8	86785572	86786920	1349	1063	97	262.62	7.73	100288527:86787244-86788322:Downstream
chr6	159532837	159533817	981	503	42	100.36	7.72	-
chr1	53950433	53950950	518	194	15	52.57	7.69	-
chr1	56629338	56629829	492	293	15	55.33	7.69	-
chr1	160272848	160273510	663	475	18	56.45	7.69	1314:160258377-160313354:Genebody
chr1	208805209	208805653	445	198	14	54.13	7.69	-
chr1	244229729	244230593	865	357	24	72.64	7.69	-
chr12	19716662	19717190	529	279	17	64.14	7.69	-
chr12	92055289	92056160	872	393	20	50.2	7.69	-
chr12	93839033	93839586	554	198	18	67.81	7.69	-
chr13	22773690	22774175	486	299	15	56	7.69	-
chr13	25897905	25898444	540	196	16	56.5	7.69	9818:25875665-25916560:Genebody
chr13	49990755	49991505	751	332	20	59.98	7.69	81617:49882786-50018221:Genebody
chr13	85077268	85077950	683	410	19	60.45	7.69	-
chr14	73157274	73157835	562	230	16	54.25	7.69	8110:73136659-73360809:Genebody
chr17	25276933	25277716	784	445	24	57.88	7.69	-
chr18	40039614	40040078	465	232	14	51.89	7.69	-

chr18	57779843	57780311	469	196	14	51.46	7.69	-
chr18	67879669	67880469	801	401	20	55.69	7.69	-
chr19	4972269	4972892	624	355	17	54.23	7.69	23030:4969123-5153606:Genebody
chr2	92322739	92326164	3426	2414	518	153.78	7.69	-
chr2	117588433	117589117	685	487	19	60.25	7.69	-
chr2	241951621	241952196	576	379	17	58.97	7.69	25992:241938254-242033643:Genebody
chr20	45885997	45886659	663	253	23	65.24	7.69	23613:45838380-45985474:Genebody
chr22	25574113	25574634	522	272	15	52.16	7.69	85379:25423940-25593413:Genebody
chr22	34317271	34317965	695	389	27	78.55	7.69	9215:33669062-34316416:Upstream
chr3	180260702	180261201	500	322	16	60.92	7.69	-
chr4	37238063	37238672	610	267	20	54.69	7.69	-
chr4	97324342	97324972	631	243	17	53.58	7.69	-
chr4	189664071	189664665	595	183	17	57.03	7.69	-
chr4_gl000194_random	123809	124297	489	207	15	55.66	7.69	-
chr5	5126462	5127218	757	427	19	53.86	7.69	-
chr5	100313358	100314120	763	419	19	53.37	7.69	-
chr6	66152753	66153614	862	469	28	97.57	7.69	346007:64429

								875-66417118 :Genebody
chr6	116980744	116981413	670	339	17	50.12	7.69	221302:116956780-116989973:Genebody
chr7	23326886	23327530	645	293	17	52.31	7.69	-
chr8	803980	804465	486	324	15	56	7.69	-
chr9	13984991	13985525	535	191	16	57.03	7.69	-
chr9	118330864	118331585	722	405	24	87.66	7.69	-
chr9	125597497	125598110	614	417	19	67.5	7.69	-
chr1	108981	109579	599	404	16	55.41	7.67	-
chr1	86411623	86412285	663	345	17	55.63	7.67	255631:86194917-86622121 :Genebody
chr1	89351738	89352225	488	287	14	53.73	7.67	2959:89318321-89357301:Genebody
chr1	114295232	114295984	753	389	18	53.83	7.67	10745:114239823-114301777:Genebody
chr1	143255379	143255948	570	245	16	58.24	7.67	-
chr1	170472311	170472936	626	436	16	52.94	7.67	-
chr1	193397150	193397723	574	375	16	57.84	7.67	-
chr1	208611256	208611927	672	262	18	60.87	7.67	-
chr1	234810798	234811437	640	233	16	51.71	7.67	-
chr1	246144336	246144854	519	303	14	50.61	7.67	64754:245912641-246580714:Genebody

chr1	246988828	246989272	445	189	14	58.5	7.67	-
chr10	577354	578045	692	236	17	53.13	7.67	22982:320131-735608:Genebody
chr10	7714381	7715086	706	349	17	51.97	7.67	-
chr10	36171070	36171557	488	244	14	53.73	7.67	-
chr10	44813103	44813702	600	415	16	55.32	7.67	-
chr10	50097739	50098453	715	441	17	51.24	7.67	57705:49893517-50191000:Genebody
chr10	56054033	56054655	623	213	16	53.21	7.67	65217:55562534-56561051:Genebody
chr10	80899468	80900024	557	321	15	53.2	7.67	57178:80828791-81076285:Genebody
chr10	84176103	84176723	621	178	18	65.89	7.67	10718:83635069-84746935:Genebody
chr10	111491026	111491771	746	370	18	54.4	7.67	-
chr11	7333428	7334093	666	405	18	61.43	7.67	143425:7273180-7490276:Genebody
chr11	14193586	14194231	646	200	17	57.17	7.67	10418:13983913-14289646:Genebody
chr11	31821547	31822123	577	307	16	57.54	7.67	5080:31806340-31832879:G

								enebody
chr11	37913345	37914095	751	386	19	59.77	7.67	-
chr11	42078816	42079400	585	267	18	69.77	7.67	-
chr11	57344007	57344477	471	215	15	62.37	7.67	-
chr11	62789240	62789694	455	260	14	57.34	7.67	-
chr11	71097997	71098571	575	314	15	51.51	7.67	-
chr11	74001569	74002120	552	285	15	53.69	7.67	283208:73977702-74022699:Genebody
chr11	75122660	75123172	513	191	14	51.19	7.67	-
chr11	100766639	100767285	647	377	16	51.11	7.67	143872:100558406-100861656:Genebody
chr11	116891005	116891621	617	293	16	53.75	7.67	23387:116714119-116968993:Genebody
chr11	120381392	120381895	504	208	14	52.09	7.67	-
chr12	24590669	24591249	581	196	15	50.96	7.67	6660:23685231-24715380:Genebody
chr12	42511198	42511809	612	419	16	54.21	7.67	283464:42475649-42538673:Genebody
chr12	63022473	63022909	437	187	14	59.45	7.67	-
chr12	82984039	82984678	640	398	16	51.71	7.67	-
chr12	98626406	98627008	603	405	16	55.04	7.67	-
chr12	104723653	104724271	619	382	16	53.57	7.67	7296:104609558-104744058

								:Genebody
chr12	117046448	117046949	502	192	14	52.29	7.67	-
chr12	124053560	124054059	500	253	14	52.49	7.67	-
chr13	42673021	42673493	473	280	14	55.33	7.67	160851:42622888-42803888:Genebody
chr13	45647498	45647975	478	281	16	61.55	7.67	-
chr13	53655388	53655950	563	387	16	58.95	7.67	-
chr13	63210659	63211183	525	342	15	56.39	7.67	-
chr13	65715865	65716422	558	385	15	53.11	7.67	-
chr13	105377949	105378405	457	198	14	57.11	7.67	-
chr13	105678942	105679433	492	192	15	59.95	7.67	-
chr14	26097970	26098679	710	324	17	51.64	7.67	-
chr14	31581235	31581723	489	175	14	53.63	7.67	25831:31569323-31676689:Genebody
chr14	43304039	43304613	575	366	15	51.51	7.67	-
chr14	56487192	56487948	757	399	18	53.51	7.67	-
chr14	68031757	68032437	681	482	18	60.03	7.67	57475:68000007-68056255:Genebody
chr14	80440881	80441356	476	278	14	55.01	7.67	-
chr14	82239201	82239660	460	245	14	56.77	7.67	-
chr14	99683089	99683662	574	364	15	51.6	7.67	64919:99635626-99737822:Genebody
chr15	59232933	59233445	513	325	14	51.19	7.67	-
chr15	64043332	64043853	522	197	14	50.32	7.67	8925:6390081

								7-64126147:Genebody
chr15	64460249	64460966	718	370	18	56.74	7.67	53944:64457717-64648442:Genebody
chr15	99791860	99792500	641	339	16	51.63	7.67	123355:99791651-99926496:Genebody
chr16	16328562	16329122	561	192	18	72.52	7.67	408050:16326388-16388667:Genebody
chr16	29122483	29123006	524	200	14	50.13	7.67	653390:29086162-29128036:Genebody
chr16	63702241	63702753	513	237	14	51.19	7.67	-
chr16	87235580	87236055	476	206	14	55.01	7.67	-
chr17	5209281	5209896	616	193	16	53.84	7.67	9135:5185557-5289131:Genebody
chr17	34636323	34636907	585	396	17	63.15	7.67	-
chr17	62168683	62169184	502	223	14	52.29	7.67	2081:62120389-62207502:Genebody
chr18	6895390	6895922	533	189	16	62.12	7.67	79822:6834483-6898718:Genebody
chr18	22478041	22478751	711	438	19	63.35	7.67	-
chr18	42321871	42322453	583	390	15	50.78	7.67	26040:422601

								37-42457378:<br>Genebody
chr18	58192809	58193427	619	198	17	59.72	7.67	-
chr19	4788077	4788688	612	375	19	73.48	7.67	-
chr19	9324736	9325187	452	225	14	57.69	7.67	125958:93245<br>74-9325513:G<br>enebody
chr19	15315557	15316133	577	340	15	51.33	7.67	-
chr19	36814712	36815365	654	492	19	68.94	7.67	-
chr19	45232107	45232778	672	285	18	60.87	7.67	-
chr19	54988870	54989458	589	393	16	56.37	7.67	-
chr19	58578566	58579281	716	375	18	56.92	7.67	7694:5857060<br>6-58581108:G<br>enebody
chr2	13519600	13520234	635	349	16	52.15	7.67	-
chr2	46089559	46090170	612	253	16	54.21	7.67	5581:4587904<br>2-46415128:G<br>enebody
chr2	61184643	61185239	597	190	20	82.15	7.67	150962:61167<br>549-61245365<br>:Genebody
chr2	68692788	68693295	508	196	14	51.69	7.67	554251:68689<br>506-68694390<br>:Genebody
chr2	76979524	76980085	562	333	16	59.05	7.67	80059:769748<br>57-77749502:<br>Genebody
chr2	102079733	102080441	709	344	18	57.52	7.67	731220:10201

								3822-1020911<br>65:Genebody
chr2	117638871	117639427	557	264	15	53.2	7.67	-
chr2	127167562	127168089	528	353	16	62.67	7.67	-
chr2	138974852	138975482	631	241	16	52.5	7.67	-
chr2	173943518	173944145	628	371	17	58.85	7.67	51776:173940<br>564-17409187<br>1:Genebody
chr20	38272371	38272892	522	247	15	56.7	7.67	-
chr20	56358005	56358563	559	366	15	53.01	7.67	-
chr20	59440865	59441526	662	376	17	55.72	7.67	-
chr21	43378143	43378920	778	382	19	57.49	7.67	-
chr21	46832531	46833286	756	380	19	59.34	7.67	80781:468250<br>96-46933633:<br>Genebody
chr21	48117576	48118316	741	464	19	60.64	7.67	-
chr22	16373954	16374609	656	338	18	62.39	7.67	-
chr22	22965436	22965882	447	313	14	58.27	7.67	-
chr22	31887964	31888502	539	197	16	61.47	7.67	-
chr22	42707877	42708376	500	301	14	52.49	7.67	-
chr3	19765351	19765955	605	444	17	61.1	7.67	-
chr3	36201238	36201813	576	243	15	51.42	7.67	-
chr3	68094246	68094742	497	318	14	52.8	7.67	407738:68053<br>453-68594769<br>:Genebody
chr3	68331890	68332512	623	438	18	65.68	7.67	407738:68053<br>453-68594769<br>:Genebody

chr3	79184129	79184744	616	394	16	53.84	7.67	6091:78646387-79817059:Genebody
chr3	129323237	129323750	514	320	14	51.1	7.67	23129:129274055-129325582:Genebody
chr3	137058793	137059263	471	272	14	55.55	7.67	-
chr3	142220811	142221421	611	301	16	54.3	7.67	545:142168077-142297668:Genebody
chr3	148976389	148976909	521	183	15	56.81	7.67	-
chr4	45156248	45156825	578	283	15	51.24	7.67	-
chr4	56570308	56570883	576	383	16	57.64	7.67	-
chr4	79177969	79178703	735	317	20	67.24	7.67	80144:78978723-79367785:Genebody
chr4	112824238	112824827	590	257	15	50.15	7.67	-
chr4	158182801	158183386	586	356	15	50.51	7.67	2891:158141735-158287224:Genebody
chr4	184208957	184209438	482	227	14	54.36	7.67	80014:184020462-184241927:Genebody
chr5	5123759	5124330	572	376	16	58.04	7.67	-
chr5	9688259	9688780	522	179	14	50.32	7.67	285692:9641427-9903936:Genebody
chr5	13838212	13838805	594	397	16	55.89	7.67	1767:1369043

								6-13944589:G enebody
chr5	23322002	23322524	523	267	14	50.22	7.67	-
chr5	33790929	33791492	564	195	17	65.39	7.67	81792:335272 86-33892124: Genebody
chr5	34775418	34775938	521	265	15	56.81	7.67	26064:346564 32-34832716: Genebody
chr5	57302447	57303033	587	195	17	62.94	7.67	-
chr5	60378076	60378598	523	311	16	63.23	7.67	91942:602409 55-60448863: Genebody
chr5	70526370	70527031	662	191	18	61.81	7.67	-
chr5	78544339	78545048	710	381	17	51.64	7.67	133746:78531 924-78623036 :Genebody
chr5	85073254	85073763	510	259	14	51.49	7.67	-
chr5	94190240	94190734	495	303	14	53	7.67	79772:940422 88-94417545: Genebody
chr5	98909981	98910471	491	294	14	53.42	7.67	-
chr5	103895911	103896568	658	412	16	50.18	7.67	-
chr5	133709242	133709897	656	281	18	62.39	7.67	7320:1337068 69-133727798 :Genebody
chr5	134155898	134156416	519	190	14	50.61	7.67	9879:1340944 60-134166809

								:Genebody
chr5	138257772	138258280	509	298	14	51.59	7.67	1495:138089106-138270722:Genebody
chr5	152036678	152037127	450	191	15	64.93	7.67	-
chr5	178585884	178586491	608	290	17	60.8	7.67	9509:178540850-178772329:Genebody
chr6	4502202	4502712	511	313	14	51.39	7.67	-
chr6	17386754	17387379	626	417	16	52.94	7.67	-
chr6	20223171	20223805	635	249	17	58.19	7.67	-
chr6	37150558	37151018	461	239	14	56.66	7.67	-
chr6	55118232	55118687	456	257	14	57.23	7.67	3062:55039070-55147416:Genebody
chr6	89686047	89686695	649	464	18	63.07	7.67	-
chr6	93268484	93268963	480	183	14	54.58	7.67	-
chr6	125377514	125378162	649	244	17	56.89	7.67	154214:125304513-125404657:Genebody
chr6	140081937	140082577	641	169	16	51.63	7.67	-
chr6	146507651	146508240	590	180	15	50.15	7.67	2911:146348781-146758731:Genebody
chr6	150840363	150840957	595	309	16	55.79	7.67	-
chr7	7358003	7358701	699	289	18	58.4	7.67	-
chr7	11034641	11035216	576	312	16	57.64	7.67	9678:11013498-11147376:G

								enebody
chr7	19532917	19533451	535	186	15	55.37	7.67	-
chr7	30277289	30277828	540	304	16	61.36	7.67	-
chr7	34208808	34209314	507	283	14	51.79	7.67	-
chr7	42002487	42003072	586	364	15	50.51	7.67	2737:42000549-42276618:Genebody
chr7	48752872	48753426	555	330	15	53.4	7.67	-
chr7	73495592	73496198	607	315	18	67.36	7.67	-
chr7	94729152	94729633	482	232	14	54.36	7.67	55607:94536948-94925725:Genebody
chr7	112816149	112816621	473	283	14	55.33	7.67	-
chr7	117734920	117735491	572	235	15	51.79	7.67	-
chr7	138703589	138704034	446	217	14	58.39	7.67	-
chr7	146590062	146590766	705	289	17	52.05	7.67	26047:145813452-148118086:Genebody
chr7	151749128	151749847	720	192	18	56.57	7.67	63917:151722777-151819427:Genebody
chr7	156791314	156791996	683	461	19	66.02	7.67	-
chr8	3432094	3432607	514	198	14	51.1	7.67	64478:2792875-4852328:Genebody
chr8	33897162	33897819	658	175	19	68.53	7.67	-
chr8	41285394	41285874	481	305	14	54.47	7.67	-
chr8	63526634	63527235	602	314	16	55.13	7.67	286183:63161

								500-63903627<br>:Genebody
chr8	75830959	75831555	597	181	19	75.19	7.67	-
chr8	99800089	99800684	596	196	17	62.01	7.67	6788:9946686<br>1-99837909:G<br>enebody
chr8	121236969	121237537	569	378	15	52.07	7.67	7373:1211373<br>51-121384266<br>:Genebody
chr9	4790405	4791010	606	200	16	54.76	7.67	-
chr9	14490545	14491028	484	286	14	54.15	7.67	-
chr9	86487531	86488028	498	310	14	52.7	7.67	55582:864516<br>16-86536342:<br>Genebody
chr9	112625283	112625925	643	256	18	63.66	7.67	114299:11240<br>3067-1127137<br>55:Genebody
chr9	125487487	125488051	565	189	17	65.28	7.67	254973:12548<br>6268-1254872<br>04:Downstrea<br>m
chrX	12003709	12004218	510	256	14	51.49	7.67	-
chrX	49355342	49356005	664	403	19	67.92	7.67	729447:49354<br>117-49361428<br>:Genebody
chrX	130270842	130271498	657	238	16	50.27	7.67	-
chr12	106761245	106762124	880	442	27	68.79	7.65	55703:106751<br>435-10690397

								4:Genebody
chr13	24238369	24239126	758	316	24	65.01	7.65	55504:24144722-24250231:Genebody
chr13	111526036	111526860	825	330	28	80.01	7.65	-
chr18	77383307	77384052	746	234	22	55.62	7.65	-
chr22	17138838	17139708	871	498	24	54.47	7.65	-
chr4	39041246	39041975	730	327	22	57.14	7.65	-
chr1	113009790	113010667	878	377	23	71.94	7.64	7482:113010039-113063908:Genebody
chr1	221430388	221431166	779	363	22	75.1	7.64	-
chr1	232576621	232577111	491	257	14	53.19	7.64	57568:232533713-232651243:Genebody
chr10	2090370	2090969	600	274	16	55.07	7.64	-
chr10	75010182	75010890	709	369	17	51.47	7.64	51021:75008600-75012451:Genebody
chr10	129733313	129733972	660	452	18	61.72	7.64	5791:129705324-129884162:Genebody
chr10	132185356	132185828	473	196	14	55.1	7.64	-
chr11	87685627	87686166	540	364	16	61.1	7.64	-
chr11	87787647	87788450	804	443	20	60.78	7.64	-
chr11	110312697	110313139	443	169	14	58.51	7.64	2230:110300593-110335604:Genebody

chr11	118910240	118910917	678	415	18	60.03	7.64	-
chr12	116625087	116625570	484	303	14	53.92	7.64	23389:116396382-116714991:Genebody
chr12	130619390	130619985	596	312	16	55.45	7.64	-
chr13	78470835	78471357	523	318	15	56.36	7.64	1910:78469615-78492966:Genebody
chr14	64596149	64596657	509	185	14	51.36	7.64	23224:64319682-64693165:Genebody
chr16	75721335	75721953	619	429	16	53.32	7.64	-
chr17	6981145	6981758	614	191	16	53.77	7.64	10462:6977856-6983600:Genebody
chr17	42492905	42493433	529	333	16	62.3	7.64	23131:42472649-42580802:Genebody
chr17	42681595	42682292	698	435	18	58.22	7.64	-
chr18	60309748	60310266	519	244	14	50.39	7.64	-
chr18	77409617	77410229	613	419	16	53.86	7.64	-
chr19	46533396	46534190	795	409	18	50.31	7.64	-
chr2	2851509	2852122	614	248	16	53.77	7.64	-
chr2	17330216	17330781	566	374	15	52.11	7.64	-
chr2	47420013	47420504	492	238	14	53.09	7.64	-
chr2	61926928	61927670	743	277	19	60.17	7.64	-
chr2	136487374	136488054	681	200	18	59.75	7.64	-
chr21	10204508	10205303	796	556	22	73.45	7.64	-

chr22	42412420	42412924	505	200	14	51.76	7.64	164684:42394728-42424475:Genebody
chr3	11690418	11690935	518	197	17	70.42	7.64	9686:11597543-11762220:Genebody
chr3	22001978	22002641	664	192	21	80.78	7.64	-
chr4	99407359	99408076	718	374	17	50.74	7.64	10098:99391518-99579812:Genebody
chr4	161147465	161148046	582	261	19	76.65	7.64	-
chr4	164994147	164994752	606	306	16	54.51	7.64	55016:164445449-165304407:Genebody
chr5	122436462	122437095	634	311	16	51.99	7.64	93166:122424840-122523744:Genebody
chr6	85882674	85883267	594	199	16	55.64	7.64	-
chr6	132977588	132978187	600	389	16	55.07	7.64	-
chr6	137991302	137991891	590	166	16	56.02	7.64	-
chr7	67916364	67916882	519	181	14	50.39	7.64	-
chr7	78288874	78289446	573	375	16	57.68	7.64	9863:77646374-79082890:Genebody
chr7	87530350	87530934	585	194	15	50.37	7.64	10926:87505543-87538856:Genebody
chr7	102331437	102332005	569	187	15	51.83	7.64	-

chr7	123752219	123752854	636	260	16	51.81	7.64	-
chr8	80042331	80043095	765	411	20	64.16	7.64	-
chr8	109613534	109614289	756	444	18	53.33	7.64	-
chr8	116223456	116223898	443	199	14	58.51	7.64	-
chr9	68692779	68693581	803	295	20	60.86	7.64	-
chr9	74047607	74048233	627	168	16	52.6	7.64	-
chr9	96004856	96005564	709	327	18	57.25	7.64	65268:95947211-96082853:Genebody
chrY	7076933	7077699	767	359	22	76.3	7.64	-
chr1	88054443	88054900	458	244	16	56.48	7.61	-
chr10	105362702	105363178	477	199	16	54.19	7.61	9644:105353783-105615164:Genebody
chr11	46584393	46585064	672	316	19	51	7.61	55626:46417963-46612914:Genebody
chr11	105172510	105172990	481	229	16	53.72	7.61	-
chr12	33821201	33821794	594	401	18	53.09	7.61	-
chr14	94670164	94671114	951	457	26	62.44	7.61	57718:94640648-94694180:Genebody
chr3	50277012	50277587	576	321	18	54.95	7.61	2771:50273646-50296786:Genebody
chr5	89966439	89966985	547	355	20	70.45	7.61	84059:89854616-90460032:Genebody

chr6	110772170	110772840	671	183	19	51.09	7.61	85413:110745906-110797844:Genebody
chr9	111982366	111983279	914	512	25	60.55	7.61	54566:111934253-112083021:Genebody
chr16	29182676	29183321	646	421	24	67.07	7.59	-
chr3	123110581	123111573	993	490	32	70.76	7.59	111:123003398-123167392:Genebody
chr4	132659537	132660083	547	367	21	62.61	7.59	-
chr1	27253916	27254690	775	408	21	56.76	7.56	10726:27248223-27272887:Genebody
chr1	113070641	113071575	935	517	27	74.89	7.56	54879:113066141-113162040:Genebody
chr1	221127936	221128586	651	467	19	57.24	7.56	-
chr10	32976880	32977598	719	322	19	50.99	7.56	79741:32856650-33171791:Genebody
chr12	69802539	69803205	667	290	19	55.69	7.56	-
chr14	20105203	20105944	742	474	24	77.19	7.56	-
chr15	45793472	45794164	693	436	20	58.82	7.56	7782:45774681-45815002:Genebody
chr15	91812638	91813371	734	306	20	55.02	7.56	9899:91643181-91844538:G

								enebody
chr17	15076377	15076928	552	301	17	55.7	7.56	-
chr2	1721767	1722454	688	490	19	53.73	7.56	7837:1635659-1748291:Genebody
chr20	40212729	40213263	535	351	16	51.55	7.56	84181:40031169-40247133:Genebody
chr21	18157616	18158505	890	442	25	68.06	7.56	-
chr22	42972867	42973324	458	171	15	53.93	7.56	91695:42969267-42978017:Genebody
chr4	58757180	58757839	660	462	20	62.11	7.56	-
chr5	67360691	67361360	670	389	22	73.04	7.56	-
chr6	7072254	7072985	732	297	21	60.7	7.56	-
chr6	22131127	22131746	620	399	18	54.58	7.56	401237:21666674-22194614:Genebody
chr8	23259212	23260132	921	476	23	55	7.56	4017:23154409-23261722:Genebody
chr8	96100204	96100820	617	372	18	54.87	7.56	-
chrUn_gl000224	10847	11873	1027	625	59	81.45	7.54	-
chr1	143228456	143229134	679	230	20	65.12	7.5	-
chr1	210172654	210173227	574	269	18	63.9	7.5	255928:210111537-210337631:Genebody

chr11	40240246	40240824	579	200	16	51.18	7.5	57689:40135752-40315664:Genebody
chr11	129193574	129194221	648	385	19	62.23	7.5	-
chr12	94615301	94615741	441	195	14	53.29	7.5	10154:94542498-94699224:Genebody
chr13	19108123	19108647	525	279	16	56.66	7.5	-
chr13	47410759	47411197	439	283	14	53.53	7.5	3356:47407512-47471169:Genebody
chr14	27542947	27543739	793	387	20	54.69	7.5	-
chr14	68568312	68568948	637	439	18	57.35	7.5	5890:68286508-68944809:Genebody
chr15	97382166	97382814	649	264	17	50.49	7.5	-
chr16	5580275	5580860	586	358	16	50.52	7.5	-
chr16	48216085	48216658	574	249	16	51.66	7.5	85320:48200821-48266182:Genebody
chr16	62916103	62916658	556	180	18	65.94	7.5	-
chr16	85361113	85361775	663	473	18	54.9	7.5	-
chr17	38625903	38626500	598	405	17	55.24	7.5	-
chr17	58949609	58950207	599	184	19	67.46	7.5	54828:58755171-59470198:Genebody
chr17	66953801	66954272	472	234	15	56.21	7.5	-
chr2	17291917	17292512	596	219	18	61.51	7.5	-

chr2	40569008	40569762	755	405	23	75.52	7.5	6546:40339286-40657444:Genebody
chr20	31169911	31170425	515	321	15	51.53	7.5	-
chr21	43426985	43427448	464	196	14	50.72	7.5	49854:43406940-43430496:Genebody
chr3	45813153	45813576	424	234	14	55.31	7.5	54716:45796941-45838035:Genebody
chr3	49470472	49471175	704	417	19	56.86	7.5	-
chr3	76222326	76222885	560	387	17	59.14	7.5	-
chr4	95449607	95450177	571	373	18	64.23	7.5	10611:95373037-95509369:Genebody
chr4	112238240	112239101	862	421	22	59.75	7.5	-
chr4	153029472	153030201	730	197	23	60.96	7.5	-
chr5	610937	611696	760	318	22	52.92	7.5	55722:612404-653664:Upstream
chr5	11172961	11173403	443	194	14	53.06	7.5	1501:10971951-11904110:Genebody
chr6	754033	754592	560	241	17	59.14	7.5	-
chr6	63472664	63473217	554	287	16	53.63	7.5	-
chr8	20378605	20379087	483	235	16	61.46	7.5	-
chr9	100882440	100883016	577	335	16	51.37	7.5	9830:100831568-100881488

								:Upstream
chr9	105331786	105332380	595	163	21	81.11	7.5	-
chr9	134038103	134038974	872	415	25	57.73	7.5	8021:134000980-134109090:Genebody
chr9	138688212	138689019	808	339	20	53.47	7.5	-
chr2	124701878	124702796	919	444	27	52.77	7.46	-
chr9	36184997	36185732	736	459	29	82.19	7.46	-
chr10	4190944	4191603	660	185	19	50.78	7.45	-
chr11	116382737	116383311	575	196	18	53.75	7.45	-
chr12	127776608	127777223	616	200	19	55.09	7.45	-
chr14	40263015	40263573	559	370	18	55.47	7.45	-
chr16	67934312	67935007	696	291	20	52.69	7.45	5681:67927174-67963580:Genebody
chr3	136971812	136972208	397	199	14	50.08	7.45	-
chr6	35218527	35218860	334	168	14	63.06	7.45	222663:35182189-35218609:Downstream
chr6	126237219	126237589	371	186	14	56.44	7.45	135112:126112000-126252266:Genebody
chr8	95889676	95890356	681	343	22	65.16	7.45	55656:95835533-95892720:Genebody
chr1	6434449	6435148	700	372	18	56.35	7.43	11332:6324332-6445883:Genebody

chr1	47759374	47760061	688	371	18	57.41	7.43	6491:47715810-47779819:Genebody
chr1	79721071	79721678	608	407	16	52.79	7.43	-
chr1	205604759	205605367	609	196	16	52.7	7.43	-
chr1	211495984	211496586	603	437	16	53.25	7.43	-
chr10	18798001	18798606	606	198	17	59.08	7.43	783:18429605-18830688:Genebody
chr10	25977794	25978473	680	339	19	64.2	7.43	-
chr10	36083140	36083748	609	415	16	52.7	7.43	-
chr11	33460488	33461100	613	425	18	64.68	7.43	-
chr12	114272118	114272595	478	289	14	53.16	7.43	9904:114254542-114404176:Genebody
chr13	47176242	47176727	486	176	15	58.87	7.43	23143:47127295-47319034:Genebody
chr13	70852841	70853675	835	344	21	62	7.43	-
chr14	65725670	65726276	607	408	16	52.88	7.43	-
chr16	19198968	19199605	638	329	16	50.13	7.43	51760:19179637-19278553:Genebody
chr16	20434263	20434830	568	388	15	50.48	7.43	54988:20420855-20452278:Genebody
chr17	5400439	5401054	616	437	16	52.07	7.43	728392:5402747-5404319:D

								ownstream
chr19	32938880	32939627	748	279	21	69.88	7.43	147991:32896654-32976797:Genebody
chr2	145276094	145276713	620	428	19	70.41	7.43	9839:145141941-145277958:Genebody
chr2	151368495	151369116	622	402	16	51.53	7.43	-
chr2	203042350	203043025	676	382	18	58.5	7.43	-
chr20	8530497	8531052	556	214	16	57.84	7.43	23236:8113295-8865545:Genebody
chr20	42377639	42378130	492	220	15	58.2	7.43	-
chr20	49672675	49673300	626	354	16	51.18	7.43	-
chr20	50380311	50380867	557	310	16	57.74	7.43	10079:50213313-50384908:Genebody
chr21	21004513	21005126	614	435	16	52.25	7.43	-
chr22	32298481	32299078	598	217	16	53.72	7.43	9681:32150068-32303000:Genebody
chr22	50667361	50667895	535	198	15	53.66	7.43	85378:50656117-50683400:Genebody
chr3	47396515	47397098	584	318	17	61.31	7.43	-
chr3	56948275	56948985	711	372	18	55.39	7.43	50650:56761445-57113336:Genebody

chr3	70903375	70904160	786	318	20	60.46	7.43	-
chr3	121705842	121706458	617	286	16	51.98	7.43	286676:121706170-121741030:Downstream
chr3	132192395	132192960	566	316	15	50.66	7.43	23317:132136552-132257875:Genebody
chr4	124527640	124528230	591	392	16	54.38	7.43	-
chr4	162752878	162753505	628	200	18	63.14	7.43	56884:162305050-163085186:Genebody
chr5	25148915	25149537	623	341	16	51.44	7.43	-
chr5	32372153	32372718	566	188	15	50.66	7.43	51663:32354455-32444844:Genebody
chr5	57220986	57221646	661	358	17	53.94	7.43	-
chr6	27445995	27446565	571	198	15	50.2	7.43	-
chr6	88799619	88800372	754	486	20	63.28	7.43	-
chr6	96096338	96097006	669	276	18	59.15	7.43	-
chr7	57306449	57307051	603	253	19	72.31	7.43	-
chr7	135395999	135396495	497	253	14	51.19	7.43	26266:135365992-135412933:Genebody
chr8	38966094	38966590	497	200	14	51.19	7.43	203102:38965049-39142435:Genebody
chr8	61256298	61256910	613	405	16	52.34	7.43	-

chr9	10629095	10629546	452	176	14	56.03	7.43	-
chr9	33107699	33108239	541	194	15	53.06	7.43	-
chr9	34969848	34970353	506	265	16	56.66	7.43	23349:34958191-34982542:Genebody
chr5	15219319	15219866	548	276	20	59.29	7.41	-
chr1	11954793	11955512	720	172	24	77.77	7.39	-
chr1	78072101	78072925	825	350	25	72.4	7.39	26009:78030190-78148343:Genebody
chr1	246375934	246376493	560	362	18	59.44	7.39	64754:245912641-246580714:Genebody
chr10	14547147	14547928	782	380	24	71.05	7.39	-
chr12	40145209	40145819	611	223	20	65.83	7.39	-
chr12	91856060	91856475	416	236	14	51.42	7.39	-
chr15	35021447	35022244	798	371	21	53.23	7.39	-
chr16	69850397	69851213	817	396	24	67.57	7.39	11060:69796273-69944310:Genebody
chr16	89141893	89142722	830	379	21	50.62	7.39	-
chr18	9075266	9075686	421	197	14	50.82	7.39	-
chr18	37496986	37497641	656	460	20	60.96	7.39	-
chr2	9616762	9617602	841	360	22	54.76	7.39	285148:9614669-9628590:Genebody
chr2	108718606	108719177	572	259	18	58.12	7.39	-
chr20	58242907	58243570	664	377	20	60.15	7.39	116154:58179

								602-58422764<br>:Genebody
chr3	175530	176054	525	367	16	51.32	7.39	-
chr3	30937149	30937902	754	240	23	68.18	7.39	339896:30767<br>691-30936153<br>:Upstream
chr4	164499680	164500257	578	309	21	76.12	7.39	55016:164445<br>449-16453477<br>6:Genebody
chr5	106843656	106844232	577	379	17	51.77	7.39	1946:1067125<br>90-107006596<br>:Genebody
chr6	136298901	136299587	687	349	20	57.87	7.39	27115:136172<br>833-13651670<br>8:Genebody
chr6	144968855	144969594	740	282	24	75.52	7.39	7402:1446128<br>72-145174168<br>:Genebody
chr6	151783381	151783928	548	320	17	54.77	7.39	79624:151773<br>421-15179123<br>2:Genebody
chr8	65669581	65670329	749	327	20	52.21	7.39	9420:6550852<br>8-65711348:G<br>enebody
chr9	76208622	76209161	540	189	18	61.72	7.39	-
chr9	86865491	86866179	689	416	21	63.34	7.39	-
chr9	129432168	129433075	908	499	24	59.4	7.39	4010:1293767<br>21-129463311

								:Genebody
chr5	17595724	17596764	1041	513	31	54.07	7.38	-
chr17	27295986	27296811	826	387	25	60.4	7.35	124925:27281946-27333081:Genebody
chr17	36609044	36609701	658	350	24	73.07	7.35	-
chr2	90460499	90460916	418	200	16	55.81	7.35	-
chr5	33431368	33431867	500	312	18	57.31	7.35	-
chr9	45128131	45129191	1061	494	41	122.82	7.35	-
chr1	104022876	104023488	613	199	17	52.36	7.32	-
chr1	142833029	142833860	832	373	20	50.01	7.32	-
chr11	132573050	132573656	607	211	20	71.23	7.32	4978:132284876-132813037:Genebody
chr12	26781218	26781824	607	354	17	52.93	7.32	3709:26488286-26986131:Genebody
chr14	82699233	82699876	644	414	20	67.08	7.32	-
chr15	64423489	64423914	426	196	14	53.8	7.32	6642:64388176-64436431:Genebody
chr15	65948126	65948734	609	416	17	52.73	7.32	9187:65914269-65948597:Genebody
chr17	29107116	29107620	505	293	17	63.92	7.32	-
chr19	11464273	11464877	605	197	17	53.12	7.32	126075:11457180-11465619:Genebody

chr2	27948207	27948703	497	180	15	52.11	7.32	-
chr2	164997410	164997831	422	182	14	54.28	7.32	-
chr22	21915338	21916031	694	267	20	61.93	7.32	-
chr22	38242079	38242844	766	392	19	50.02	7.32	724029:38243684-38243781:Downstream
chr3	125169128	125169927	800	323	21	57.85	7.32	8723:125165494-125239058:Genebody
chr5	141029594	141030295	702	271	20	61.16	7.32	89848:141018869-141030986:Genebody
chr6	69002036	69002772	737	347	20	57.89	7.32	-
chr7	42240760	42241323	564	321	16	51.27	7.32	2737:42000549-42276618:Genebody
chr7	57123822	57124266	445	248	15	58.07	7.32	-
chr9	16474965	16475504	540	164	17	59.84	7.32	54796:16409501-16870786:Genebody
chr9	18095865	18096580	716	401	19	54.22	7.32	-
chr9	84366075	84366768	694	309	21	67.87	7.32	-
chrUn_gl000214	72	929	858	281	34	80.39	7.31	-
chr1	23004923	23005463	541	200	18	56.19	7.29	-
chr1	94292711	94293369	659	403	20	54.87	7.29	-
chr10	130180991	130181891	901	460	30	84.89	7.29	-
chr12	90616778	90617354	577	386	18	52.27	7.29	-

chr12	124316511	124317075	565	172	20	65.22	7.29	196385:124247041-124420265:Genebody
chr3	81063471	81064407	937	494	24	50.67	7.29	-
chr4	3816285	3817385	1101	552	44	87.75	7.29	-
chr5	77035784	77036660	877	384	23	50.68	7.29	6902:76986994-77072185:Genebody
chr8	108314323	108314818	496	315	17	55.52	7.29	284:108261710-108510254:Genebody
chr8	120943482	120943898	417	176	15	53.06	7.29	64798:120885899-121063156:Genebody
chr1	214517204	214517930	727	241	24	54.31	7.27	-
chr12	130111606	130112364	759	427	29	77.05	7.27	121256:129556270-130388212:Genebody
chr22	50776098	50776716	619	345	21	55.31	7.27	-
chr4	7622859	7623334	476	282	18	55.69	7.27	57537:7194373-7744563:Genebody
chr8	129415593	129416085	493	258	18	53.55	7.27	-
chr9_gl000199_random	120024	121660	1637	1285	122	222.89	7.25	-
chr1	27720029	27720848	820	328	19	50.5	7.24	2827:27719151-27722315:Genebody

chr1	41748713	41749355	643	291	17	53.99	7.24	-
chr1	55920978	55921599	622	366	16	50.06	7.24	-
chr1	115991190	115991867	678	389	19	62.63	7.24	-
chr1	208515592	208516177	586	391	16	53.36	7.24	-
chr1	211957261	211957802	542	197	15	51.54	7.24	9926:211916799-212004114:Genebody
chr1	230864204	230864946	743	386	19	56.69	7.24	-
chr1	232244600	232245199	600	320	16	52.05	7.24	-
chr1	236771577	236772217	641	210	17	54.18	7.24	-
chr10	17722521	17723124	604	396	16	51.68	7.24	8027:17686123-17757907:Genebody
chr10	52368126	52368859	734	267	19	57.47	7.24	259230:52065345-52383737:Genebody
chr10	72816486	72817142	657	409	17	52.74	7.24	-
chr10	127803125	127803730	606	370	16	51.5	7.24	8038:127702901-128077127:Genebody
chr11	109793718	109794392	675	375	17	51.18	7.24	-
chr11	119994282	119994776	495	177	14	50.05	7.24	23650:119981994-120008863:Genebody
chr12	121720923	121721512	590	200	16	52.98	7.24	10645:121675495-121734556:Genebody
chr13	24182450	24183178	729	450	20	63.76	7.24	55504:241447

								22-24250231: Genebody
chr13	88573262	88573836	575	183	16	54.42	7.24	-
chr13	94595180	94595848	669	286	17	51.7	7.24	10082:93879077-95060267: Genebody
chr15	82257186	82257664	479	225	14	51.7	7.24	-
chr15	93158308	93158904	597	258	16	52.32	7.24	-
chr16	62989365	62989893	529	198	16	59.17	7.24	-
chr16	80245901	80246485	585	400	16	53.46	7.24	-
chr17	9432693	9433417	725	450	19	58.26	7.24	9482:9153788-9479275:Genebody
chr17	12228427	12228886	460	267	14	53.76	7.24	-
chr17	26392905	26393381	477	194	15	58.43	7.24	51701:26369687-26523402: Genebody
chr17	30200363	30201034	672	484	18	57.22	7.24	55813:30190190-30228729: Genebody
chr18	14234881	14235423	543	173	15	51.45	7.24	-
chr18	20449430	20450210	781	315	23	76.91	7.24	-
chr18	27917368	27917920	553	372	16	56.63	7.24	-
chr18	71686742	71687480	739	541	19	57.03	7.24	-
chr2	49744156	49744750	595	425	16	52.51	7.24	-
chr2	61858618	61859351	734	504	19	57.47	7.24	-
chr2	74563318	74564130	813	461	19	51.03	7.24	57835:74443369-74570534:

								Genebody
chr2	134336045	134336649	605	422	16	51.59	7.24	-
chr2	240001173	240001862	690	289	20	67.56	7.24	9759:2399698 64-240322643 :Genebody
chr20	29536532	29537195	664	259	17	52.13	7.24	-
chr20	41792234	41792962	729	284	18	52.26	7.24	11122:407013 92-41818557: Genebody
chr20	62762541	62763329	789	387	20	58.41	7.24	-
chr21	36342162	36342773	612	224	18	63.08	7.24	861:36160098 -36421595:Ge nebody
chr3	113179163	113179744	582	196	17	59.91	7.24	152185:11316 1566-1132340 34:Genebody
chr4	67462656	67463356	701	335	20	66.46	7.24	-
chr4	187049707	187050395	689	498	19	61.57	7.24	-
chr5	52239509	52240135	627	296	18	61.55	7.24	3672:5208413 5-52249484:G enebody
chr5	78050270	78050737	468	273	14	52.88	7.24	-
chr5	120640698	120641277	580	322	18	66.52	7.24	-
chr5	135463531	135464087	557	207	16	56.22	7.24	9597:1354652 04-135470579 :Downstream
chr5	180900090	180901314	1225	790	35	100.16	7.24	-
chr6	8948245	8948798	554	191	15	50.39	7.24	-

chr7	63679906	63680518	613	416	16	50.86	7.24	730291:63667580-63680666:Genebody
chr8	37772715	37773456	742	374	20	62.55	7.24	-
chr8	47540312	47540917	606	382	16	51.5	7.24	-
chr8	62421882	62422408	527	276	15	53.04	7.24	444:62413115-62602408:Genebody
chr8	63291713	63292257	545	351	16	57.46	7.24	286183:63161500-63903627:Genebody
chr8	96005286	96005924	639	328	17	54.36	7.24	-
chr9	94673059	94673661	603	416	17	57.78	7.24	4920:94484883-94712444:Genebody
chr1	2539665	2540475	811	460	21	50.67	7.22	79258:2522080-2564481:Genebody
chr1	16876658	16877244	587	196	21	73.3	7.22	-
chr10	79888214	79888665	452	266	15	52.21	7.22	-
chr11	65771720	65772242	523	280	17	56.2	7.22	8815:65769549-65771616:Downstream
chr12	131328767	131329745	979	503	25	56.93	7.22	-
chr14	23611671	23612565	895	489	23	53.8	7.22	23428:23594505-23623610:Genebody
chr2	241704421	241704989	569	200	18	57.05	7.22	547:24165318

								4-241759624: Genebody
chr3	178594144	178594890	747	401	21	56.17	7.22	-
chr4	6795411	6796042	632	199	20	61.97	7.22	9778:6784458-6885897:Genebody
chr4	63748712	63749541	830	436	27	81.48	7.22	-
chr4	79255661	79256680	1020	633	31	79.09	7.22	80144:78978723-79367785: Genebody
chr5	9996404	9996829	426	220	15	55.38	7.22	-
chr5	98026111	98026822	712	485	24	76.84	7.22	-
chr5	109799355	109800022	668	344	20	58.24	7.22	642987:109755199-110062450:Genebody
chr6	33829480	33830059	580	388	18	55.87	7.22	-
chr1	106743921	106744428	508	334	18	55.14	7.21	-
chr1	162408574	162409427	854	454	26	61.22	7.21	-
chr11	12837065	12837734	670	265	25	70.06	7.21	7003:12695968-12966283:Genebody
chr2	240071877	240072830	954	505	29	66.7	7.21	9759:239969864-240322643:Genebody
chr1	1829443	1830490	1048	560	35	74.46	7.2	-
chr1	120612978	120613713	736	439	25	58.04	7.2	4853:120454177-120612276:Upstream

chrY	58890533	58891525	993	275	45	87.48	7.19	-
chr1	161029442	161029979	538	194	16	52.55	7.14	257106:161016732-161039760:Genebody
chr1	174118066	174118545	480	195	16	59	7.14	-
chr1	229760478	229761133	656	199	23	82.91	7.14	27097:229728867-229761794:Genebody
chr10	19375063	19375859	797	379	20	51.21	7.14	-
chr10	80367003	80367666	664	182	19	57.48	7.14	-
chr10	130236605	130237375	771	487	24	63.68	7.14	-
chr11	40204972	40205525	554	200	17	56.87	7.14	57689:40135752-40315664:Genebody
chr12	65610346	65610859	514	331	17	52.23	7.14	23592:65563350-65642139:Genebody
chr12	122379261	122380029	769	453	21	58.91	7.14	144406:122356462-122409972:Genebody
chr13	102132176	102132910	735	397	20	56.47	7.14	9358:102104965-102368793:Genebody
chr13	106547662	106548146	485	240	15	52.13	7.14	-
chr14	93635650	93636463	814	432	22	60.36	7.14	-
chr15	20120197	20121197	1001	497	38	64.67	7.14	-
chr15	51940810	51941256	447	209	17	60.49	7.14	-
chr17	63786499	63787255	757	348	21	60	7.14	201134:63631

								658-63822661:Genebody
chr19	16038424	16039181	758	332	24	65.01	7.14	57834:16023179-16045676:Genebody
chr2	67899282	67900330	1049	626	38	95.33	7.14	-
chr2	120161931	120162659	729	289	20	57.02	7.14	-
chr2	187386059	187386842	784	492	23	57.2	7.14	-
chr2	200227955	200228470	516	210	16	54.88	7.14	23314:200134223-200322819:Genebody
chr20	40544652	40545226	575	257	17	54.68	7.14	-
chr20	59080549	59081133	585	192	18	59.62	7.14	-
chr20	59625963	59626520	558	343	18	62.59	7.14	-
chr20	59777374	59778058	685	375	26	85.08	7.14	-
chr3	90327881	90328501	621	200	18	55.92	7.14	-
chr3	107217318	107218097	780	451	26	53.25	7.14	-
chr3	133366823	133367538	716	518	22	58.51	7.14	11073:133319449-133380737:Genebody
chr3	139782319	139782961	643	422	22	56.67	7.14	64084:139654026-140286917:Genebody
chr3	157794312	157794889	578	200	18	60.37	7.14	-
chr4	14976259	14977112	854	467	23	62.26	7.14	-
chr4	29645705	29646502	798	516	24	73.17	7.14	-
chr4	55069021	55069779	759	428	22	65.45	7.14	-
chr4	159058244	159058800	557	313	16	50.63	7.14	51313:159045

								732-15909371<br>8:Genebody
chr5	10204982	10205627	646	304	18	53.51	7.14	-
chr5	31313068	31313782	715	425	19	52.79	7.14	1004:3119379<br>5-31325237:G<br>enebody
chr5	44771637	44772279	643	461	22	78.02	7.14	-
chr5	80843656	80844530	875	487	22	55.24	7.14	23635:807156<br>72-81047072:<br>Genebody
chr5	108922786	108923333	548	200	16	51.52	7.14	-
chr6	10483125	10483712	588	383	19	65.43	7.14	-
chr6	43001076	43001984	909	336	32	79.51	7.14	-
chr6	102948669	102949262	594	199	20	60.4	7.14	-
chr6	141875667	141876489	823	513	23	65.04	7.14	-
chr6	155670006	155670920	915	497	26	60.45	7.14	-
chr8	7797400	7798253	854	343	21	51.81	7.14	-
chr8	97448219	97448873	655	432	20	64.24	7.14	-
chr9	118924053	118924629	577	378	18	60.48	7.14	5069:1189160<br>70-119164599<br>:Genebody
chr9	130276701	130277131	431	183	15	58.5	7.14	64855:130267<br>618-13033135<br>5:Genebody
chrUn_gl000<br>226	-13	1344	1358	192	142	251.03	7.14	-
chrY	58861843	58863365	1523	882	113	226.27	7.11	-
chr16	32102720	32103633	914	414	27	59.03	7.08	-

chr1	109741863	109742457	595	273	18	52.98	7.07	57535:109656584-109749401:Genebody
chr1	145721455	145721856	402	215	14	50.9	7.07	-
chr1	177380648	177381630	983	607	30	80.54	7.07	-
chr10	66027550	66028166	617	196	20	62.11	7.07	-
chr11	57990969	57991712	744	454	21	54.95	7.07	-
chr15	37193828	37194677	850	429	22	50.97	7.07	4212:37183232-37390503:Genebody
chr17	15620476	15620982	507	300	17	56.74	7.07	57335:15602890-15624098:Genebody
chr18	42791901	42792639	739	433	21	55.41	7.07	-
chr2	227257127	227257739	613	259	18	51.2	7.07	-
chr3	185144094	185144778	685	392	20	55.12	7.07	9175:185080969-185200624:Genebody
chr3	185786637	185787227	591	304	21	71.18	7.07	2119:185764107-185826901:Genebody
chr5	171691971	171692548	578	182	18	54.74	7.07	92181:171636649-171710795:Genebody
chr5	179790293	179790900	608	417	18	51.69	7.07	-
chr6	148999444	148999990	547	307	17	52.29	7.07	-
chr1	104894250	104894844	595	188	16	51.07	7.05	-
chr10	47147992	47148620	629	213	19	65.88	7.05	-

chr10	80269465	80269941	477	190	15	57.01	7.05	-
chr10	91943579	91944204	626	387	17	54.04	7.05	-
chr11	4366602	4367217	616	352	17	54.99	7.05	-
chr11	39041145	39041605	461	189	14	52.33	7.05	-
chr13	77054233	77054709	477	284	14	50.6	7.05	-
chr13	108123134	108123837	704	442	19	58.48	7.05	728215:107820879-108519460:Genebody
chr15	56334784	56335241	458	220	14	52.66	7.05	-
chr16	67417880	67418572	693	327	19	59.5	7.05	55282:67360746-67419108:Genebody
chr2	29716757	29717519	763	501	20	58.91	7.05	238:29415640-30144432:Genebody
chr2	59523544	59524189	646	352	18	58.05	7.05	-
chr2	126007183	126007893	711	310	21	69.74	7.05	-
chr2	173157888	173158366	479	252	14	50.39	7.05	-
chr2	202677653	202678320	668	413	17	50.29	7.05	65061:202671197-202758263:Genebody
chr2	213994174	213994796	623	427	17	54.32	7.05	22807:213864412-214015058:Genebody
chr22	20343050	20343866	817	485	25	83.45	7.05	-
chr22	33833722	33834514	793	259	24	79.76	7.05	9215:33669062-34316416:Genebody

chr3	2143685	2144273	589	163	17	57.64	7.05	152330:2142246-3099644:Genebody
chr3	20327118	20327713	596	357	16	50.98	7.05	-
chr3	145763039	145763669	631	433	17	53.58	7.05	-
chr3	178629759	178630376	618	420	17	54.8	7.05	-
chr4	6272571	6273136	566	199	16	53.85	7.05	7466:6271576-6304991:Genebody
chr4	61389950	61390532	583	187	16	52.2	7.05	-
chr4	110399839	110400444	606	197	17	55.95	7.05	10427:110354970-110461614:Genebody
chr4	128301462	128302064	603	140	16	50.34	7.05	-
chr4	138747329	138747938	610	440	17	55.56	7.05	-
chr5	56728140	56728717	578	195	16	52.68	7.05	-
chr6	1085736	1086493	758	399	19	53.78	7.05	285768:961241-1101567:Genebody
chr6	4022850	4023490	641	458	17	52.67	7.05	8899:4021568-4065216:Genebody
chr6	81213239	81213957	719	323	18	51.53	7.05	-
chr7	55179200	55179949	750	462	19	54.45	7.05	1956:55086724-55224642:Genebody
chr7	134329235	134329906	672	370	18	55.62	7.05	669:134331530-134364565:

								Upstream
chr8	126792977	126793675	699	402	19	58.94	7.05	-
chr8	141364011	141364643	633	473	19	65.46	7.05	83696:140742585-141467861:Genebody
chr9	22527104	22527694	591	245	17	57.43	7.05	-
chr9	86964587	86965366	780	395	19	51.99	7.05	64078:86893091-86983413:Genebody
chr9	130982251	130983044	794	374	19	50.89	7.05	1759:130965662-131017527:Genebody
chr1	149015996	149016844	849	423	24	54.87	7	-
chr1	227296124	227296894	771	376	23	56.95	7	8476:227177566-227505826:Genebody
chr10	60694623	60695182	560	235	19	57.22	7	-
chr14	20161989	20162793	805	488	27	69.32	7	-
chr17	77920250	77921006	757	287	24	63.54	7	125058:77913820-78009647:Genebody
chr21	44888057	44888723	667	248	21	56.71	7	114038:44881973-44898103:Genebody
chr5	4585816	4586650	835	380	25	61.1	7	-
chr1	246335265	246336439	1175	565	42	77.74	6.99	64754:245912641-246580714:Genebody

chr1	28653210	28653813	604	197	16	54.95	6.98	54797:28655512-28662477:Upstream
chr1	30377446	30377952	507	269	14	51.79	6.98	-
chr1	46196923	46197606	684	433	17	53.8	6.98	3652:46159999-46216485:Genebody
chr1	102938550	102939202	653	460	16	50.6	6.98	-
chr1	105307520	105308013	494	197	14	53.11	6.98	-
chr1	113002125	113002753	629	196	16	52.67	6.98	55917:112938799-113003785:Genebody
chr1	117569703	117570291	589	393	19	76.13	6.98	9398:117544381-117579166:Genebody
chr1	120825990	120826572	583	155	15	50.78	6.98	-
chr1	148233119	148233708	590	377	15	50.15	6.98	-
chr1	159232018	159232589	572	197	15	51.79	6.98	-
chr1	163686070	163686562	493	149	16	66.72	6.98	-
chr1	197481218	197481741	524	333	14	50.13	6.98	163486:197479508-197744623:Genebody
chr1	202477370	202477825	456	299	14	57.23	6.98	4660:202317829-202557697:Genebody
chr1	220301465	220302078	614	315	16	54.02	6.98	55699:220267454-220321376:Genebody

chr1	223111350	223111873	524	334	14	50.13	6.98	84976:223101782-223179335:Genebody
chr10	1954466	1955073	608	424	16	54.57	6.98	-
chr10	2416225	2416787	563	373	16	58.95	6.98	-
chr10	28779217	28779821	605	182	16	54.85	6.98	-
chr10	37170735	37171255	521	336	14	50.42	6.98	-
chr10	38890295	38890908	614	337	16	54.02	6.98	-
chr10	45376146	45376654	509	256	14	51.59	6.98	-
chr10	63806772	63807510	739	311	19	60.82	6.98	84159:63661012-63856707:Genebody
chr10	65559303	65559777	475	233	15	51.99	6.98	-
chr10	68173966	68174536	571	217	17	64.63	6.98	29119:67679724-69425416:Genebody
chr10	72212076	72212600	525	269	14	50.03	6.98	-
chr10	88700619	88701280	662	467	19	68.12	6.98	79812:88695299-88717425:Genebody
chr10	98706091	98706813	723	477	21	74.72	6.98	84458:98592016-98724198:Genebody
chr10	100319127	100319645	519	180	16	53.24	6.98	60495:100216834-100995632:Genebody
chr10	109755598	109756215	618	426	16	53.66	6.98	-
chr11	49567634	49568508	875	398	25	69.45	6.98	-

chr11	60978716	60979330	615	235	17	60.11	6.98	643834:60970983-60980349:Genebody
chr11	66066783	66067363	581	205	16	57.15	6.98	-
chr11	99294620	99295204	585	387	15	50.6	6.98	53942:98891870-100227472:Genebody
chr11	114258893	114259394	502	145	14	52.29	6.98	-
chr11	128793897	128794476	580	168	15	51.05	6.98	-
chr12	12370170	12370769	600	413	16	55.32	6.98	4040:12268962-12419811:Genebody
chr12	17781586	17782097	512	242	14	51.29	6.98	-
chr12	38231055	38231740	686	196	18	59.57	6.98	-
chr12	56138446	56138963	518	169	14	50.71	6.98	10220:56137063-56146664:Genebody
chr12	65943048	65943630	583	386	16	56.95	6.98	-
chr12	75664622	75665244	623	247	16	53.21	6.98	-
chr12	79498845	79499481	637	438	17	58	6.98	6857:79257772-79845787:Genebody
chr12	98654649	98655209	561	391	16	52.82	6.98	-
chr12	110712710	110713221	512	166	15	57.76	6.98	-
chr12	115523269	115523856	588	177	15	50.33	6.98	-
chr13	38608021	38608615	595	322	16	55.79	6.98	-
chr13	47720670	47721240	571	249	16	58.14	6.98	-
chr13	49351272	49351861	590	423	15	50.15	6.98	-

chr13	67897477	67898099	623	395	18	65.68	6.98	-
chr13	83975679	83976306	628	445	16	52.76	6.98	-
chr13	85180296	85180901	606	365	16	54.76	6.98	-
chr13	93829905	93830491	587	195	16	56.56	6.98	-
chr13	114158786	114159287	502	159	14	52.29	6.98	55002:114145307-114204542:Genebody
chr14	35224287	35224850	564	232	15	52.54	6.98	11177:35221938-35344853:Genebody
chr14	45794265	45794875	611	192	16	54.3	6.98	-
chr14	57614621	57615168	548	309	16	60.51	6.98	-
chr14	72684347	72684957	611	176	16	54.3	6.98	9628:72399785-73033237:Genebody
chr14	74228022	74228688	667	243	19	67.61	6.98	91748:74181825-74227001:Upstream
chr14	75109249	75109895	647	387	18	63.27	6.98	-
chr14	86878496	86879191	696	206	19	64.77	6.98	-
chr14	91917821	91918419	599	293	16	55.41	6.98	-
chr14	104850486	104851021	536	349	16	61.79	6.98	-
chr14	106014478	106015053	576	392	16	57.64	6.98	-
chr15	21183477	21183994	518	365	14	50.71	6.98	-
chr15	40837016	40837540	525	196	14	50.03	6.98	-
chr15	61354092	61354738	647	344	17	57.08	6.98	6095:60780484-61521502:Genebody

chr15	61896736	61897316	581	161	15	50.96	6.98	-
chr15	87240929	87241424	496	278	14	52.9	6.98	123624:86685241-87572283:Genebody
chr15	96511579	96512062	484	266	14	54.15	6.98	-
chr15	97794387	97794935	549	354	15	53.98	6.98	-
chr16	24996804	24997435	632	436	17	58.47	6.98	55114:24930713-25026675:Genebody
chr16	52092582	52093195	614	449	16	54.02	6.98	-
chr16	62385464	62386129	666	478	18	50.28	6.98	-
chr16	65265888	65266522	635	463	16	52.15	6.98	-
chr16	67448288	67448876	589	286	15	50.24	6.98	29800:67428322-67450339:Genebody
chr16	70963550	70964130	581	246	15	50.96	6.98	54768:70841289-71264569:Genebody
chr17	715997	716502	506	198	14	51.89	6.98	64359:702585-883010:Genebody
chr17	10345796	10346319	524	329	14	50.13	6.98	4622:10346609-10372876:Downstream
chr17	35578409	35579033	625	385	17	59.14	6.98	31:35441927-35656692:Genebody
chr17	38576298	38576822	525	188	14	50.03	6.98	-

chr17	50127128	50127631	504	165	14	52.09	6.98	56934:49707674-50236132:Genebody
chr17	54534922	54535577	656	169	16	50.35	6.98	162282:54230835-54560007:Genebody
chr17	70665706	70666221	516	177	16	53.56	6.98	201266:70642085-71088853:Genebody
chr18	4748646	4749260	615	373	16	53.93	6.98	-
chr18	7847372	7847956	585	427	16	56.75	6.98	5797:7567313-8406858:Genebody
chr18	29841242	29841831	590	170	16	56.27	6.98	-
chr18	46872060	46872664	605	415	16	54.85	6.98	54808:46570171-46987079:Genebody
chr18	48899213	48899761	549	363	16	60.4	6.98	-
chr19	11500895	11501406	512	183	14	51.29	6.98	-
chr19	12571925	12572635	711	393	17	51.56	6.98	163051:12571998-12595632:Genebody
chr19	20603462	20603957	496	195	14	52.9	6.98	664701:20574520-20607762:Genebody
chr19	37726297	37726831	535	197	16	61.9	6.98	163087:37717365-37734574:Genebody

chr19	56225270	56225776	507	193	14	51.79	6.98	338321:56219798-56249768:Genebody
chr2	1645703	1646303	601	422	18	56.48	6.98	7837:1635659-1748291:Genebody
chr2	13407281	13407875	595	182	18	68.66	6.98	-
chr2	26306564	26307098	535	183	15	55.37	6.98	10890:26256728-26360322:Genebody
chr2	28175661	28176225	565	194	16	58.74	6.98	9577:28113481-28561765:Genebody
chr2	28850724	28851240	517	277	16	63.91	6.98	151056:28718937-28866651:Genebody
chr2	32943733	32944416	684	418	17	53.8	6.98	55622:32853128-33046116:Genebody
chr2	41825241	41825673	433	173	15	50.56	6.98	-
chr2	90435985	90436575	591	232	15	50.07	6.98	-
chr2	90474382	90475024	643	425	18	63.66	6.98	-
chr2	119173353	119173940	588	200	15	50.33	6.98	-
chr2	137611344	137611878	535	310	16	61.9	6.98	-
chr2	149195942	149196540	599	243	16	55.41	6.98	55777:148778579-149271042:Genebody
chr2	162833811	162834401	591	402	17	62.52	6.98	57282:162480

								844-16284178<br>5:Genebody
chr2	167912879	167913514	636	233	16	52.06	6.98	129446:16775<br>9974-1681162<br>59:Genebody
chr2	168488376	168488924	549	186	16	50.13	6.98	-
chr2	187881137	187881704	568	373	16	58.44	6.98	-
chr2	188618448	188619047	600	365	16	55.32	6.98	-
chr2	200846424	200846984	561	205	16	52.82	6.98	-
chr2	201214345	201214917	573	187	17	53.51	6.98	26010:201170<br>603-20134698<br>2:Genebody
chr2	226045036	226045555	520	189	14	50.51	6.98	-
chr2	237950501	237951055	555	317	15	53.4	6.98	-
chr2	241867997	241868622	626	250	16	52.94	6.98	-
chr20	2437600	2438257	658	327	18	62.2	6.98	-
chr20	10007046	10007666	621	383	17	59.53	6.98	-
chr20	43523481	43523941	461	193	14	56.66	6.98	7529:4351434<br>3-43537160:G<br>enebody
chr20	52769074	52769502	429	199	14	51.03	6.98	1591:5276998<br>7-52790516:D<br>ownstream
chr20	53854887	53855508	622	247	16	53.3	6.98	-
chr21	22764418	22765049	632	184	18	64.76	6.98	4685:2237063<br>2-22911214:G<br>enebody
chr21	35338042	35338527	486	216	15	50.77	6.98	-

chr22	19239630	19240153	524	325	14	50.13	6.98	8218:19166988-19279239:Genebody
chr22	21535913	21536468	556	290	15	53.3	6.98	-
chr22	29355551	29356100	550	323	18	62.02	6.98	84133:29279889-29453475:Genebody
chr22	39487598	39488250	653	341	16	50.6	6.98	-
chr3	440450	441023	574	172	15	51.6	6.98	10752:238649-451095:Genebody
chr3	12603289	12603838	550	278	16	60.3	6.98	23609:12598593-12625208:Genebody
chr3	14245492	14246001	510	198	14	51.49	6.98	-
chr3	39321587	39322180	594	398	16	55.89	6.98	1524:39304985-39321527:Upstream
chr3	45835809	45836478	670	259	19	67.31	6.98	54716:45796941-45838035:Genebody
chr3	145908516	145909213	698	335	17	52.62	6.98	57088:145910125-145968651:Downstream
chr3	167542869	167543556	688	242	19	65.54	6.98	5274:167453431-167543356:Genebody

chr3	187245255	187245859	605	421	16	54.85	6.98	-
chr3	189602007	189602530	524	327	14	50.13	6.98	8626:189349215-189615068:Genebody
chr4	5849200	5849664	465	199	14	56.21	6.98	1400:5822491-5890315:Genebody
chr4	30022717	30023292	576	339	15	51.42	6.98	-
chr4	40259096	40259648	553	260	15	53.59	6.98	-
chr4	67591989	67592707	719	262	22	81.68	6.98	-
chr4	86398724	86399375	652	400	17	56.62	6.98	83478:86396283-86923822:Genebody
chr4	94631838	94632360	523	273	14	50.22	6.98	2895:93225549-94693648:Genebody
chr4	99140253	99141042	790	369	22	74.37	6.98	-
chr4	104333566	104334117	552	194	15	53.69	6.98	-
chr4	115271801	115272476	676	481	18	60.49	6.98	-
chr4	123823309	123824166	858	431	24	65.53	6.98	11162:123813804-123843759:Genebody
chr4	148066703	148067269	567	224	15	52.25	6.98	-
chr4	187442350	187442929	580	390	16	57.24	6.98	-
chr5	18654977	18655479	503	304	14	52.19	6.98	-
chr5	25510871	25511381	511	190	14	51.39	6.98	-
chr5	52640127	52640756	630	199	16	52.59	6.98	-
chr5	65295313	65296053	741	348	20	66.67	6.98	55914:652223

								83-65376850:<br>Genebody
chr5	73744750	73745236	487	270	14	53.84	6.98	-
chr5	83866370	83866915	546	335	16	60.72	6.98	-
chr5	84180343	84181001	659	255	18	62.1	6.98	-
chr5	85714975	85715492	518	323	14	50.71	6.98	-
chr5	93765289	93765855	567	193	15	52.25	6.98	285600:93486<br>556-93954309<br>:Genebody
chr5	96664866	96665417	552	372	16	60.09	6.98	-
chr5	110781641	110782197	557	197	16	59.56	6.98	814:11056008<br>1-110820746:<br>Genebody
chr5	114155027	114155734	708	361	20	57.39	6.98	-
chr5	115358367	115359032	666	351	18	50.28	6.98	206338:11529<br>8150-1153632<br>98:Genebody
chr5	118047209	118047832	624	306	16	53.12	6.98	-
chr5	123844492	123845049	558	286	15	53.11	6.98	-
chr5	124640756	124641352	597	413	16	55.6	6.98	-
chr5	124850545	124851246	702	269	19	64.2	6.98	-
chr5	125914748	125915258	511	308	15	57.87	6.98	501:12587891<br>7-125931082:<br>Genebody
chr5	141812376	141813008	633	438	16	52.32	6.98	-
chr5	175794918	175795522	605	407	16	54.85	6.98	285598:17579<br>2501-1758005<br>03:Genebody

chr6	35065471	35065954	484	204	14	54.15	6.98	-
chr6	41238993	41239480	488	302	14	53.73	6.98	-
chr6	46799870	46800391	522	285	16	52.92	6.98	4224:46761093-46807519:Genebody
chr6	70943597	70944151	555	297	15	53.4	6.98	1297:70925743-70992911:Genebody
chr6	86678008	86678550	543	280	15	54.57	6.98	-
chr6	88955804	88956324	521	340	14	50.42	6.98	-
chr6	96261032	96261526	495	211	14	53	6.98	-
chr6	121950433	121951039	607	359	17	50.18	6.98	-
chr6	132671282	132671896	615	357	16	53.93	6.98	26002:132617194-132722664:Genebody
chr6	134628587	134629169	583	343	18	69.99	6.98	6446:134490384-134639196:Genebody
chr6	136885557	136886071	515	330	16	53.67	6.98	4217:136878187-137113656:Genebody
chr6	156558413	156558964	552	357	15	53.69	6.98	-
chr6	159846282	159846947	666	348	20	61.5	6.98	-
chr7	3043090	3043786	697	269	18	58.58	6.98	84433:2945768-3083579:Genebody
chr7	24238785	24239474	690	495	17	53.29	6.98	-
chr7	36624708	36625329	622	299	16	53.3	6.98	313:36552548

								-36764154:Genebody
chr7	55093105	55093772	668	317	17	55.19	6.98	1956:55086724-55224642:Genebody
chr7	85510403	85510996	594	397	16	55.89	6.98	-
chr7	86805443	86806024	582	223	15	50.87	6.98	9988:86781676-86825646:Genebody
chr8	63379017	63379708	692	376	18	59.03	6.98	286183:63161500-63903627:Genebody
chr8	76055406	76055909	504	239	14	52.09	6.98	-
chr8	120451322	120451990	669	284	17	55.1	6.98	-
chr8	134540316	134540896	581	408	15	50.96	6.98	6482:134467090-134584183:Genebody
chr9	12223933	12224518	586	196	15	50.51	6.98	-
chr9	13028899	13029509	611	337	17	60.5	6.98	-
chr9	24314376	24314897	522	340	14	50.32	6.98	-
chr9	68673909	68674580	672	272	17	54.84	6.98	-
chr9	70491837	70492542	706	263	17	51.97	6.98	-
chr9	86895442	86896025	584	394	15	50.69	6.98	64078:86893091-86983413:Genebody
chr9	92452567	92453109	543	362	15	54.57	6.98	-
chr9	98369884	98370490	607	279	16	54.67	6.98	-
chr9	99188865	99189445	581	212	17	63.57	6.98	-

chr9	117823462	117823981	520	194	14	50.51	6.98	3371:117782805-117880486:Genebody
chr9	123774651	123775318	668	395	18	61.24	6.98	727:123714615-123812554:Genebody
chr9	134259715	134260373	659	194	16	50.1	6.98	-
chr9_gl000199_random	117148	117658	511	313	14	51.39	6.98	-
chrUn_gl000212	45580	46133	554	360	15	53.49	6.98	NR_027278:24048-60768:Genebody
chrX	20135262	20135795	534	192	15	55.47	6.98	256714:20024832-20134756:Upstream
chrX	25108895	25109413	519	251	14	50.61	6.98	-
chrX	49021578	49022183	606	371	17	61	6.98	79917:49019180-49023836:Genebody
chr1	57381462	57381965	504	194	14	51.86	6.94	731:57320442-57383894:Genebody
chr1	58821498	58822118	621	423	16	53.14	6.94	-
chr1	89709245	89709875	631	248	16	52.25	6.94	-
chr1	175250261	175250763	503	302	14	51.96	6.94	-
chr1	197263650	197264142	493	299	15	59.59	6.94	23418:197237407-197447584:Genebody

chr1	232617649	232618146	498	327	14	52.47	6.94	57568:232533713-232651243:Genebody
chr10	26535022	26535711	690	215	19	65.05	6.94	2572:26505235-26590048:Genebody
chr10	28339367	28339834	468	193	14	55.65	6.94	143098:28339922-28571067:Downstream
chr10	34390439	34390944	506	195	14	51.66	6.94	-
chr10	35041628	35042276	649	182	19	69.16	6.94	56288:34398487-35104253:Genebody
chr10	58474611	58475271	661	344	20	74.43	6.94	-
chr10	107162850	107163428	579	198	16	57.09	6.94	-
chr11	4752630	4753221	592	406	16	55.83	6.94	-
chr11	11014651	11015234	584	196	15	50.46	6.94	-
chr11	23307507	23308286	780	416	21	68.83	6.94	-
chr11	34783825	34784594	770	330	19	57.87	6.94	-
chr11	108101477	108102100	624	276	16	52.87	6.94	472:108093558-108239826:Genebody
chr12	49310577	49311055	479	238	14	54.46	6.94	85478:49297892-49315358:Genebody
chr12	49924423	49925073	651	265	19	68.95	6.94	-
chr12	77201936	77202466	531	188	16	62.08	6.94	23390:77157853-77247474:

								Genebody
chr13	36065812	36066408	597	428	16	55.35	6.94	26960:35516423-36246872:Genebody
chr13	41004994	41005614	621	305	16	53.14	6.94	646982:40921272-41055143:Genebody
chr13	94532499	94533017	519	196	14	50.39	6.94	10082:93879077-95060267:Genebody
chr14	30363398	30364162	765	297	20	64.16	6.94	5587:30045688-30396899:Genebody
chr15	76803714	76804188	475	288	17	51.18	6.94	49855:76640528-77154285:Genebody
chr15	86335685	86336187	503	294	14	51.96	6.94	64410:86302560-86338189:Genebody
chr16	19123252	19123850	599	403	17	61.44	6.94	162073:19125253-19132948:Upstream
chr16	54994014	54994606	593	185	16	55.73	6.94	-
chr16	89643289	89643896	608	196	17	60.53	6.94	27132:89642175-89663653:Genebody
chr16	90057764	90058285	522	218	14	50.1	6.94	172:90038987-90063028:Ge

								nebody
chr17	4871310	4872135	826	318	22	70.64	6.94	9552:4862522-4871132:Upstream
chr17	6920075	6920678	604	312	16	54.7	6.94	124944:6918072-6920838:Genebody
chr17	15275221	15275695	475	218	14	54.89	6.94	-
chr17	75282730	75283243	514	307	14	50.87	6.94	10801:75277491-75496676:Genebody
chr18	107549	108860	1312	650	134	110.88	6.94	727758:109064-122217:Upstream
chr18	50751055	50751645	591	217	16	55.92	6.94	1630:49866541-51062273:Genebody
chr18	76786061	76786652	592	438	18	68.7	6.94	-
chr19	19249467	19250091	625	435	18	65.19	6.94	54929:19230430-19249267:Upstream
chr2	56555238	56555740	503	304	14	51.96	6.94	114800:56411257-56613308:Genebody
chr2	85246646	85247212	567	395	15	52.02	6.94	56888:85198230-85286594:Genebody
chr2	90147443	90147904	462	225	14	56.32	6.94	-

chr2	142209774	142210389	616	193	18	66.13	6.94	53353:140988995-142889270:Genebody
chr2	184870633	184871166	534	257	15	55.23	6.94	-
chr2	203176517	203177060	544	347	15	54.23	6.94	-
chr2	236553831	236554282	452	263	14	57.46	6.94	116987:236402735-237034114:Genebody
chr20	55626533	55627108	576	386	16	57.39	6.94	-
chr20	57462648	57463383	736	402	18	54.95	6.94	2778:57414794-57486249:Genebody
chr21	41272146	41272797	652	428	23	63.65	6.94	5121:41239346-41301320:Genebody
chr21	47054843	47055897	1055	664	38	99.04	6.94	-
chr3	47734396	47735019	624	176	18	65.3	6.94	6599:47627377-47823405:Genebody
chr3	100734261	100734821	561	384	15	52.58	6.94	-
chr3	141727260	141727872	613	270	16	53.86	6.94	7029:141663276-141747507:Genebody
chr4	19349472	19350051	580	385	15	50.82	6.94	-
chr4	39210148	39210654	507	240	14	51.56	6.94	57728:39184023-39287429:Genebody
chr4	102092643	102093262	620	187	17	59.36	6.94	5530:1019445

								86-102268628<br>:Genebody
chr4	137788421	137788990	570	192	16	57.98	6.94	-
chr4	153350461	153350950	490	173	14	53.29	6.94	55294:153242<br>410-15345617<br>2:Genebody
chr5	59874538	59875110	573	413	15	51.46	6.94	-
chr5	103904913	103905399	487	198	14	53.61	6.94	-
chr6	43039783	43040430	648	370	16	50.78	6.94	89953:430273<br>71-43042833:<br>Genebody
chr6	87164194	87164791	598	200	22	64.51	6.94	-
chr6	138340031	138340653	623	365	16	52.96	6.94	-
chr7	16899620	16900128	509	274	14	51.36	6.94	155465:16899<br>030-16921613<br>:Genebody
chr7	41452318	41452835	518	239	15	56.88	6.94	-
chr8	47984960	47985556	597	379	16	55.35	6.94	-
chr8	96045450	96046054	605	413	16	54.6	6.94	137682:96037<br>220-96070937<br>:Genebody
chr8	104674068	104674558	491	312	14	53.19	6.94	9699:1045129<br>75-105265451<br>:Genebody
chr9	7253154	7254017	864	417	24	79.29	6.94	-
chr9	110876285	110877008	724	435	22	50.98	6.94	-
chrX	140845794	140846398	605	164	20	80.85	6.94	-
chr1	208322239	208323113	875	529	34	78.33	6.92	5362:2081955

								89-208417665:Genebody
chr2	239394220	239395712	1493	528	51	87.32	6.92	-
chr5	3483902	3485040	1139	720	37	60.52	6.92	-
chr5	17517653	17518237	585	306	22	52.88	6.92	-
chr5	56105304	56106200	897	455	35	80.7	6.92	-
chr6	166032274	166033435	1162	437	42	84.58	6.92	10846:165740778-166075584:Genebody
chr1	54489746	54490435	690	281	20	53.25	6.91	-
chr10	58809766	58810379	614	291	21	66.85	6.91	-
chr13	99803793	99804306	514	187	17	54.66	6.91	-
chr18	58246807	58247461	655	221	21	62.26	6.91	-
chr19	14045133	14045963	831	400	23	56	6.91	79883:14042001-14049289:Genebody
chr19	58942131	58942846	716	338	20	50.87	6.91	7691:58944181-58951589:Downstream
chr2	43703023	43703547	525	230	17	53.42	6.91	63892:43457975-43823113:Genebody
chr20	10784052	10784713	662	320	22	67.25	6.91	-
chr5	15394809	15395524	716	327	20	50.87	6.91	-
chr6	76466684	76467325	642	278	20	57.99	6.91	4646:76458908-76629254:Genebody
chr6	147958066	147958842	777	342	21	50.62	6.91	-

chr7	111934306	111934821	516	206	17	54.43	6.91	11179:111846642-111983989:Genebody
chr8	118729173	118729761	589	185	24	89.11	6.91	-
chr10	3525292	3526223	932	458	35	88.98	6.9	-
chr2	102021080	102021650	571	293	20	51.9	6.9	731220:102013822-102091165:Genebody
chr1	221146166	221146745	580	325	16	51.08	6.88	-
chr10	111352556	111353252	697	515	21	69.34	6.88	-
chr11	58778874	58779708	835	427	20	51.35	6.88	-
chr11	70482491	70483133	643	300	18	56.78	6.88	22941:70313961-70507923:Genebody
chr11	84418416	84419085	670	248	18	54.26	6.88	1740:83166055-84634465:Genebody
chr11	122918434	122919012	579	381	16	51.18	6.88	-
chr12	12201737	12202252	516	286	15	51.43	6.88	-
chr13	48571226	48571814	589	198	16	50.24	6.88	8803:48516791-48575462:Genebody
chr14	22424367	22424916	550	189	16	54.04	6.88	-
chr15	68853901	68854696	796	439	21	59.9	6.88	-
chr17	47093432	47094040	609	273	18	60.15	6.88	10642:47074773-47133505:Genebody
chr17	55865685	55866228	544	245	18	67.35	6.88	-

chr19	12148339	12148951	613	177	17	53.79	6.88	-
chr2	115504276	115504901	626	457	18	58.43	6.88	57628:115199898-116602324:Genebody
chr20	7150968	7151701	734	356	20	59.82	6.88	-
chr20	33458747	33459360	614	196	19	65.8	6.88	2686:33432523-33460661:Genebody
chr20	49291656	49292256	601	402	18	60.98	6.88	-
chr3	15438126	15438807	682	380	18	53.18	6.88	-
chr3	71743877	71744389	513	197	15	51.74	6.88	317649:71728441-71774526:Genebody
chr3	110667908	110668495	588	196	16	50.33	6.88	-
chr3	140221819	140222520	702	503	21	68.83	6.88	64084:139654026-140286917:Genebody
chr4	33375192	33375852	661	172	21	73.23	6.88	-
chr4	173387952	173388573	622	429	18	58.83	6.88	442117:172734574-173961556:Genebody
chr5	21022008	21022739	732	407	20	60	6.88	-
chr5	148520284	148520750	467	254	14	50.4	6.88	22885:148521053-148639999:Upstream
chr5	176379563	176380138	576	377	16	51.47	6.88	51720:176332005-176433443:Genebody

chr6	6329183	6329814	632	268	17	52.02	6.88	-
chr6	22762245	22762834	590	370	16	50.15	6.88	-
chr7	54213312	54213772	461	215	14	51.05	6.88	-
chr8	17896723	17897220	498	281	16	53.32	6.88	-
chr8	106584926	106585514	589	377	17	56.13	6.88	23414:106331146-106816765:Genebody
chr8	120779357	120780052	696	394	18	51.96	6.88	6873:120743014-120845074:Genebody
chr8	122918719	122919343	625	432	18	58.53	6.88	-
chrY	58851984	58852555	572	215	16	51.85	6.88	-
chr1	183209351	183209899	549	182	16	54.14	6.87	3918:183155173-183210398:Genebody
chr11	61015263	61015793	531	295	16	56.02	6.87	5222:61008643-61018928:Genebody
chr12	106637305	106637942	638	444	18	57.26	6.87	10970:106631659-106641713:Genebody
chr14	66729738	66730270	533	199	16	55.81	6.87	-
chr16	15400515	15401066	552	303	16	53.83	6.87	-
chr2	240436955	240437622	668	333	18	54.44	6.87	-
chr4	83093740	83094298	559	248	16	53.13	6.87	-
chr12	40660880	40661503	624	189	21	59.93	6.86	120892:40618812-40763084:Genebody

chr15	89364649	89365427	779	470	23	54.77	6.86	176:89346673-89418585:Genebody
chr2	188745129	188745615	487	236	18	58.94	6.86	-
chr6	12623332	12624059	728	497	22	54.53	6.86	-
chr8	21252667	21253441	775	300	22	50.24	6.86	-
chr9	93489231	93489993	763	214	27	60.98	6.85	-
chr1	143479166	143479909	744	418	20	52.64	6.82	-
chr10	37344596	37345071	476	280	16	56.82	6.82	-
chr10	119974400	119974916	517	246	16	52.17	6.82	-
chr11	32988775	32989429	655	198	21	51.52	6.82	79832:32914791-33001812:Genebody
chr12	55496694	55497365	672	491	20	59.34	6.82	-
chr13	35259001	35259793	793	424	25	75.71	6.82	-
chr13	73944840	73945629	790	397	22	59.17	6.82	-
chr13	112933576	112934484	909	442	28	61.17	6.82	-
chr15	33791938	33792700	763	565	22	61.67	6.82	6263:33603176-34158302:Genebody
chr15	94494747	94495733	987	455	28	73.47	6.82	-
chr16	3779642	3780450	809	381	25	74.03	6.82	1387:3775057-3930121:Genebody
chr2	129697452	129698178	727	377	20	54.14	6.82	-
chr2	201206659	201207236	578	385	23	71.49	6.82	26010:201170603-201346982:Genebody

chr2	227495164	227496010	847	563	25	70.23	6.82	-
chr20	11245328	11246035	708	520	20	55.87	6.82	-
chr20	54517897	54518803	907	454	25	64.68	6.82	-
chr20	56639669	56640182	514	186	18	64.86	6.82	-
chr3	5582102	5582791	690	189	20	57.58	6.82	-
chr3	110558866	110559399	534	200	16	50.39	6.82	-
chr3	124172621	124173189	569	324	17	52.58	6.82	8997:1238135<br>57-124238698<br>:Genebody
chr3	173188224	173188952	729	281	20	53.96	6.82	22871:173116<br>243-17400111<br>6:Genebody
chr3	184654707	184655340	634	438	18	51.84	6.82	23355:184529<br>930-18477040<br>2:Genebody
chr4	8595053	8595766	714	362	21	60.83	6.82	8532:8594434<br>-8621486:Gen<br>ebody
chr4	162790949	162791637	689	285	21	63.34	6.82	56884:162305<br>050-16308518<br>6:Genebody
chr5	17525935	17528224	2290	799	93	100.64	6.82	-
chr5	33935284	33935956	673	478	20	59.24	6.82	51289:339364<br>90-33939023:<br>Upstream
chr5	57074229	57075038	810	369	21	52.23	6.82	-
chr7	135832620	135833335	716	421	21	60.64	6.82	-
chr9	5831001	5831647	647	464	19	56.16	6.82	79956:578457

								1-5833081:Genebody
chr9	121770986	121771469	484	297	16	55.87	6.82	-
chr9_gl000199_random	90987	93299	2313	860	280	209.55	6.81	-
chr9_gl000199_random	167576	169035	1460	966	83	128.91	6.8	-
chr10	1689490	1690190	701	200	30	72.73	6.79	105:1228073-1779718:Genebody
chr19	16410826	16411673	848	446	28	61.11	6.78	-
chr19	47473352	47473990	639	302	23	58.57	6.78	2909:47421932-47508323:Genebody
chr1	33887139	33887912	774	387	23	59.65	6.77	-
chr1	38800311	38800719	409	198	15	54.1	6.77	-
chr1	51150773	51151572	800	378	22	52.21	6.77	11124:50906934-51425936:Genebody
chr1	83561040	83561692	653	459	19	50.14	6.77	-
chr1	118126687	118127344	658	463	20	54.97	6.77	-
chr11	34512374	34513040	667	368	20	54.08	6.77	2001:34500341-34533346:Genebody
chr12	102454873	102455631	759	328	22	55.85	6.77	51019:102406717-102455899:Genebody
chr13	23651560	23652327	768	311	22	55.03	6.77	-

chr13	80036860	80037641	782	503	22	53.77	6.77	-
chr17	22244375	22245274	900	411	24	53.58	6.77	-
chr2	31486741	31487334	594	366	18	50.54	6.77	30845:31456879-31491259:Genebody
chr5	10850116	10850813	698	246	20	51.15	6.77	-
chr5	14521243	14521762	520	328	17	52.76	6.77	-
chr5	53555687	53556413	727	407	24	64.33	6.77	-
chr6	168610258	168611331	1074	877	35	88.83	6.77	-
chr7	7227162	7227849	688	377	21	57.38	6.77	56913:7222245-7283979:Genebody
chr7	94489818	94490712	895	487	26	63.91	6.77	-
chr9	68477602	68478479	878	473	23	50.6	6.77	-
chr1	65545320	65545932	613	186	16	52.34	6.76	-
chr1	103255360	103255980	621	199	16	51.62	6.76	-
chr1	112595353	112595969	617	433	16	51.98	6.76	-
chr1	193412073	193412725	653	454	17	54.65	6.76	-
chr1	221256055	221256609	555	363	16	57.94	6.76	-
chr10	32122328	32122957	630	447	16	50.82	6.76	94134:32095224-32217770:Genebody
chr10	126726183	126726933	751	408	20	63.55	6.76	1488:126676418-126849103:Genebody
chr11	24868130	24868784	655	200	19	66.69	6.76	338645:24518555-25104182:Genebody

chr11	34167060	34167672	613	390	16	52.34	6.76	55226:34127110-34168457:Genebody
chr12	62610086	62610761	676	457	18	58.5	6.76	-
chr12	70880093	70880663	571	172	16	56.32	6.76	-
chr12	80727896	80728520	625	174	16	51.26	6.76	283310:80603232-80772870:Genebody
chr12	93756437	93756988	552	373	15	51.99	6.76	-
chr13	29114081	29114639	559	197	15	51.32	6.76	-
chr15	65904285	65904969	685	190	19	63.71	6.76	81556:65871119-65903407:Upstream
chr15	84397446	84398036	591	389	16	54.38	6.76	57188:84322837-84708591:Genebody
chr16	28221831	28222446	616	190	18	64.37	6.76	23214:28109315-28223190:Genebody
chr16	67721865	67722436	572	379	15	50.11	6.76	81577:67708436-67753273:Genebody
chr16	85992988	85993611	624	313	16	51.35	6.76	-
chr18	39462684	39463317	634	400	16	50.48	6.76	-
chr18	70435758	70436305	548	361	15	52.37	6.76	81832:70414786-70532934:Genebody
chr19	23405955	23406527	573	190	16	56.12	6.76	-

chr19	44740077	44740805	729	307	18	53.87	6.76	7770:44716690-44741420:Genebody
chr2	124692910	124693410	501	289	14	50.78	6.76	-
chr2	147845751	147846303	553	231	15	51.89	6.76	-
chr21	17013282	17013747	466	235	14	54.46	6.76	-
chr21	19586664	19587247	584	307	16	55.05	6.76	-
chr22	47564635	47565194	560	304	15	51.23	6.76	25771:47158548-47569722:Genebody
chr22	48024034	48024769	736	413	21	71.06	6.76	400932:48016791-48027318:Genebody
chr3	49496876	49497496	621	285	16	51.62	6.76	-
chr3	59593450	59594012	563	312	16	57.12	6.76	-
chr3	149414229	149414788	560	332	15	51.23	6.76	25937:149235021-149421060:Genebody
chr3	168079077	168079793	717	449	18	54.88	6.76	93556:167967309-168548372:Genebody
chr3	182875478	182876063	586	403	19	67.58	6.76	27074:182840003-182880667:Genebody
chr4	39484260	39484828	569	195	16	50.39	6.76	401127:39481874-39483521:Downstream
chr4	42382736	42383331	596	169	17	60.08	6.76	-

chr5	14347057	14347601	545	389	15	52.67	6.76	7204:14143828-14509450:Genebody
chr5	24897215	24897911	697	296	18	56.61	6.76	-
chr5	72396473	72396970	498	323	14	51.08	6.76	-
chr6	34200845	34201396	552	272	15	51.99	6.76	-
chr6	114044948	114045557	610	397	17	58.69	6.76	-
chr6	137652384	137652980	597	192	17	59.98	6.76	-
chr7	104601986	104602493	508	277	15	56.45	6.76	-
chr7	156084747	156085382	636	308	17	56.2	6.76	-
chr8	134652347	134653002	656	265	17	54.38	6.76	-
chr9	24478219	24478742	524	235	15	54.77	6.76	-
chr11	19314111	19314848	738	423	22	52.25	6.73	-
chr14	97994192	97994825	634	443	20	52.19	6.73	-
chr18	71612535	71613345	811	321	27	70.71	6.73	-
chr2	200368066	200368554	489	200	17	51.68	6.73	-
chr3	127274189	127275011	823	373	25	59.17	6.73	-
chr6	55860437	55860987	551	189	18	50.25	6.73	-
chr8	25645026	25645743	718	267	22	54.14	6.73	-
chr1	23871589	23872165	577	200	18	62	6.71	-
chr10	47114301	47115103	803	267	21	57.59	6.71	-
chr11	33131660	33132169	510	324	15	50.74	6.71	1479:33106134-33183037:Genebody
chr12	4628570	4629266	697	284	21	67.56	6.71	57102:4596910-4647637:Genebody
chr12	66627867	66628639	773	291	21	60.22	6.71	11213:665829

								77-66648392:Genebody
chr13	36125927	36126868	942	531	26	72.81	6.71	100302239:36048405-36515382:Genebody
chr15	35580085	35580619	535	222	17	60.4	6.71	-
chr15	48746994	48747547	554	339	16	52.26	6.71	2200:48700503-48937985:Genebody
chr16	22268957	22269537	581	392	18	61.56	6.71	29904:22217591-22300065:Genebody
chr2	33789200	33789850	651	457	21	72.53	6.71	25780:33661415-33789797:Genebody
chr2	74338376	74338986	611	351	18	58.41	6.71	-
chr2	97125886	97126601	716	519	21	65.63	6.71	-
chr2	157789808	157790374	567	226	16	50.97	6.71	-
chr2	239899746	239900297	552	354	16	52.46	6.71	-
chr21	21121474	21122013	540	186	16	53.68	6.71	-
chr3	39159579	39160299	721	193	21	65.13	6.71	199223:39149151-39180394:Genebody
chr3	54256950	54257596	647	328	19	60.72	6.71	55799:54156692-55108582:Genebody
chr3	59962434	59962888	455	191	14	50.46	6.71	2272:5973503

								7-61237133:Genebody
chr3	151974415	151975003	589	274	17	54.68	6.71	-
chr5	34428783	34429460	678	334	19	57.69	6.71	-
chr5	86604215	86604986	772	312	21	60.31	6.71	5921:86564150-86687732:Genebody
chr5	94808454	94809125	672	209	18	52.59	6.71	9652:94800122-94890682:Genebody
chr5	129557239	129557845	607	242	17	52.93	6.71	-
chr5	135279578	135280212	635	189	17	50.33	6.71	-
chr5	142287795	142288352	558	198	16	51.86	6.71	23092:142150291-142608571:Genebody
chr6	81563759	81564392	634	336	18	56.12	6.71	-
chr6	149481516	149482216	701	292	18	50.06	6.71	-
chr7	33409343	33409902	560	198	16	51.66	6.71	27241:33169151-33645680:Genebody
chr7	68216748	68217378	631	455	19	62.36	6.71	-
chr7	94560230	94560814	585	233	17	55.08	6.71	55607:94536948-94925725:Genebody
chr7	145285823	145286654	832	447	20	50.01	6.71	-
chr8	30984	31868	885	454	23	61.4	6.71	-
chr8	29075346	29075833	488	235	15	53.08	6.71	23303:28924795-29120610:

								Genebody
chr8	50191783	50192415	633	418	19	62.15	6.71	-
chr8	71384645	71385179	535	185	16	54.2	6.71	-
chr8	145952668	145953264	597	196	19	66.03	6.71	90987:145946294-145980970:Genebody
chr14	95403691	95405041	1351	435	45	89.06	6.7	-
chr4	135565469	135566165	697	464	24	61.17	6.7	-
chr1	7776789	7777410	622	433	22	75.25	6.67	23261:6845383-7829763:Genebody
chr1	76618943	76619568	626	175	21	68.59	6.67	256435:76540388-77042890:Genebody
chr10	37306552	37307056	505	180	16	52.23	6.67	-
chr11	22275295	22275990	696	194	22	66.76	6.67	203859:22214721-22304913:Genebody
chr11	40298064	40298744	681	368	20	56.96	6.67	57689:40135752-40315664:Genebody
chr12	53932411	53933092	682	475	19	51.44	6.67	11016:53905844-53994805:Genebody
chr12	89784642	89785334	693	247	19	50.45	6.67	-
chr13	21788988	21789528	541	328	17	54.21	6.67	-
chr13	23592111	23592782	672	260	20	57.84	6.67	-
chr14	43414749	43415569	821	411	22	54.87	6.67	-

chr14	105327517	105328219	703	512	23	66.02	6.67	-
chr15	35119839	35120588	750	427	20	50.69	6.67	-
chr15	64689510	64690269	760	365	21	55	6.67	9325:64680019-64747500:Genebody
chr16	22972211	22972945	735	414	21	57.27	6.67	-
chr16	67684166	67684723	558	256	18	58.26	6.67	146206:67679029-67691470:Genebody
chr19	28393119	28393922	804	431	23	61.61	6.67	-
chr19	39110623	39111445	823	326	25	70.75	6.67	27335:39109721-39127595:Genebody
chr20	8136597	8137164	568	200	19	63.15	6.67	23236:8113295-8865545:Genebody
chr20	15926702	15927610	909	379	24	57.62	6.67	140733:13976145-16033839:Genebody
chr20	23195972	23196646	675	206	22	63.2	6.67	-
chr3	57842447	57843068	622	170	18	51.64	6.67	7871:57743173-57914894:Genebody
chr3	102617635	102618459	825	445	23	59.7	6.67	-
chr3	117820472	117821013	542	198	18	60.08	6.67	-
chr3	133627998	133628499	502	196	16	52.56	6.67	-
chr3	177941268	177941839	572	176	17	50.98	6.67	-
chr5	56629512	56630178	667	283	20	58.34	6.67	-

chr5	101818006	101818782	777	385	25	75.53	6.67	133482:101707653-101834720:Genebody
chr5	121026923	121027533	611	415	19	58.39	6.67	-
chr6	40200266	40200794	529	289	18	61.6	6.67	-
chr6	53520432	53520986	555	360	18	58.6	6.67	401265:53512699-53530506:Genebody
chr6	108541452	108542013	562	163	18	57.82	6.67	8724:108532717-108582464:Genebody
chr7	7949350	7949972	623	226	18	51.54	6.67	-
chr7	32763117	32763961	845	515	23	57.95	6.67	-
chr8	53044551	53045344	794	314	21	52.07	6.67	9705:53023391-53322439:Genebody
chr9	2560266	2560944	679	301	19	51.72	6.67	401491:2535655-2622373:Genebody
chr1	145323223	145324089	867	496	57	144.5	6.65	100132406:145293370-145368682:Genebody
chr9_gl000199_random	95518	96027	510	250	25	58.76	6.65	-
chr17	77039134	77039856	723	260	26	53.21	6.64	114897:77020250-77045869:Genebody

chr1	196506008	196506629	622	457	21	62.96	6.63	343450:196194912-196577499:Genebody
chr10	71891358	71891927	570	194	20	63.2	6.63	84883:71872029-71892690:Genebody
chr11	18383123	18384023	901	460	25	56.77	6.63	2965:18343815-18388590:Genebody
chr13	114175220	114176200	981	436	28	64.53	6.63	55002:114145307-114204542:Genebody
chr2	21087951	21088813	863	371	25	60.1	6.63	-
chr2	132779309	132779932	624	376	19	51.67	6.63	-
chr20	47085858	47086277	420	221	15	51.58	6.63	-
chr5	145399908	145400757	850	404	24	56.28	6.63	153769:145316125-145442877:Genebody
chr6	5136091	5136827	737	366	23	61.75	6.63	57128:5108653-5261172:Genebody
chr9	2228447	2228952	506	311	18	59.05	6.63	-
chr13	51147616	51148269	654	309	21	54.09	6.6	-
chr5	99477609	99478512	904	486	29	69.83	6.6	-
chr5	179186427	179187105	679	254	21	51.59	6.6	9794:179159850-179204285:Genebody
chr6	51200627	51201536	910	410	25	50.14	6.6	-

chr9	132230312	132231099	788	473	23	51.24	6.6	-
chrY	13437381	13438065	685	517	21	51.01	6.6	-
chr1	92564342	92564833	492	306	14	50.35	6.58	284697:92545861-92613395:Genebody
chr1	93716988	93717586	599	407	20	77.63	6.58	343099:93645919-93744266:Genebody
chr1	95549855	95550493	639	275	17	54.36	6.58	-
chr11	8052420	8053012	593	228	16	52.7	6.58	-
chr11	10575507	10576114	608	430	16	51.31	6.58	-
chr11	107546730	107547311	582	371	16	53.74	6.58	-
chr11	110584257	110584827	571	409	16	54.81	6.58	57569:110447765-110583451:Upstream
chr12	114426467	114427062	596	291	16	52.42	6.58	-
chr13	46119654	46120274	621	424	16	50.15	6.58	220081:46115431-46189874:Genebody
chr15	61008420	61008981	562	196	16	55.71	6.58	6095:60780484-61521502:Genebody
chr15	87929351	87929967	617	196	18	62.57	6.58	-
chr16	5291277	5291787	511	200	15	54.69	6.58	-
chr16	64592972	64593454	483	199	14	51.28	6.58	-
chr17	13960093	13960688	596	197	16	52.42	6.58	-
chr17	48307459	48308087	629	178	17	55.29	6.58	-
chr18	769001	769550	550	250	15	50.77	6.58	7525:721593-

								812327:Gene body
chr18	71111927	71112616	690	200	21	73.85	6.58	-
chr19	18131936	18132430	495	300	14	50.05	6.58	-
chr2	149790653	149791292	640	210	19	66.46	6.58	3800:149632818-149883273:Genebody
chr2	179313321	179313926	606	192	16	51.5	6.58	100302152:179246804-179541007:Genebody
chr2	217097900	217098563	664	186	19	64.01	6.58	-
chr20	24667227	24667912	686	200	20	67.97	6.58	-
chr20	40144015	40144688	674	288	17	51.27	6.58	84181:40031169-40247133:Genebody
chr21	44189864	44190372	509	303	15	54.9	6.58	5152:44073861-44195616:Genebody
chr4	8182228	8183045	818	349	33	94.19	6.58	-
chr4	12118762	12119353	592	263	16	52.79	6.58	-
chr4	130247992	130248613	622	189	17	55.94	6.58	-
chr4	152831934	152832426	493	255	14	50.25	6.58	-
chr4	169160437	169161123	687	272	18	55.86	6.58	55601:169137442-169239958:Genebody
chr4	182043346	182043962	617	394	16	50.5	6.58	-
chr6	22733562	22734108	547	299	15	51.06	6.58	-

chr6	48637854	48638855	1002	479	31	63.51	6.58	-
chr6	90813379	90814031	653	196	17	53.1	6.58	60468:90636247-91006562:Genebody
chr6	104505394	104505997	604	200	16	51.68	6.58	-
chr6	135374253	135374708	456	194	14	54.21	6.58	10767:135281516-135376036:Genebody
chr7	50560443	50561051	609	191	18	63.39	6.58	1644:50526135-50628768:Genebody
chr7	104837845	104838550	706	172	19	59.98	6.58	6733:104756823-104909477:Genebody
chr7	123127247	123127804	558	279	15	50.01	6.58	154865:123092237-123174718:Genebody
chr9	22804348	22805272	925	446	27	52.27	6.58	-
chr9	90327422	90327938	517	328	15	54.06	6.58	-
chr9	116381850	116382443	594	344	18	64.99	6.58	-
chrUn_gl000225	61422	62973	1552	466	56	79.95	6.58	-
chrUn_gl000225	54786	56380	1595	484	51	62.09	6.56	-
chr1	67983168	67983964	797	375	21	56.45	6.55	-
chr10	12178710	12179460	751	480	20	55.06	6.55	55176:12171639-12207403:Genebody

chr10	135132446	135133402	957	492	27	74.91	6.55	-
chr12	12662930	12663562	633	409	18	54.75	6.55	80824:12626215-12715448:Genebody
chr13	19093536	19094267	732	312	20	56.74	6.55	-
chr13	83700783	83701508	726	267	20	57.29	6.55	-
chr13	100224669	100225199	531	198	16	53.27	6.55	-
chr15	35663039	35663658	620	425	17	50.32	6.55	89978:35663170-35838404:Genebody
chr15	53052450	53053129	680	290	18	50.44	6.55	3175:53049352-53082209:Genebody
chr16	80569845	80570525	681	301	18	50.35	6.55	-
chr17	77069290	77069950	661	188	19	57.77	6.55	64772:77071018-77084679:Upstream
chr18	75249500	75250165	666	446	19	57.29	6.55	-
chr19	36361758	36362277	520	322	16	54.45	6.55	333:36359400-36370698:Genebody
chr2	81183067	81183737	671	406	18	51.23	6.55	-
chr2	137816693	137817308	616	420	18	56.41	6.55	80731:137748461-138435287:Genebody
chr2	177633165	177633721	557	215	18	62.71	6.55	-
chr2	229932057	229932604	548	357	18	63.74	6.55	55022:229888689-23013605

								7:Genebody
chr21	20348671	20349291	621	192	17	50.23	6.55	-
chr22	17332097	17332724	628	341	18	55.23	6.55	-
chr22	35563865	35564556	692	263	20	60.5	6.55	-
chr22	35779029	35779796	768	445	20	53.6	6.55	3162:35777059-35790205:Genebody
chr3	18992094	18992959	866	535	22	55.97	6.55	-
chr3	49094199	49094730	532	261	16	53.17	6.55	54870:49067143-49131504:Genebody
chr3	120437748	120438545	798	324	22	61.79	6.55	285282:120405529-120461384:Genebody
chr4	8530526	8531183	658	475	19	58.06	6.55	-
chr4	62396439	62396933	495	306	16	57.24	6.55	23284:62362838-62938167:Genebody
chr4	71723623	71724341	719	509	20	57.93	6.55	-
chr4	140814255	140814922	668	312	18	51.5	6.55	55534:140637545-141075233:Genebody
chr4	168456214	168456968	755	462	20	54.71	6.55	-
chr5	42631211	42631878	668	340	20	62.89	6.55	2690:42424025-42721925:Genebody
chr5	101314916	101315521	606	312	17	51.63	6.55	-
chr5	157393153	157393785	633	281	18	54.75	6.55	-

chr6	13015133	13015830	698	196	20	54.3	6.55	221692:12717832-13287528:Genebody
chr7	24941117	24941744	628	312	18	55.23	6.55	26031:24836164-25019760:Genebody
chr7	81208059	81208672	614	376	18	56.61	6.55	-
chr7	82717286	82717958	673	180	19	56.62	6.55	27445:82383320-82792197:Genebody
chr7	155224477	155225319	843	474	21	52.67	6.55	-
chr8	12231779	12232461	683	324	19	55.68	6.55	100133172:12219527-12268510:Genebody
chr8	79898919	79899529	611	171	17	51.16	6.55	-
chr1	27076116	27076941	826	409	22	52.93	6.52	8289:27022521-27108601:Genebody
chr1	111400388	111401181	794	390	21	50.61	6.52	-
chr11	59338422	59339133	712	368	20	52.61	6.52	-
chr11	103685608	103686345	738	459	23	66.42	6.52	-
chr12	15894681	15895357	677	446	19	50.54	6.52	2059:15773075-15942510:Genebody
chr12	82015836	82016502	667	352	19	51.46	6.52	8499:81653355-82153109:Genebody

chr12	92877087	92877694	608	197	18	51.69	6.52	-
chr13	40200301	40200891	591	301	18	53.39	6.52	-
chr14	48849989	48850693	705	455	21	58.63	6.52	-
chr14	96115398	96116006	609	237	19	57.19	6.52	27004:96117514-96139789:Upstream
chr16	59464093	59464873	781	407	21	51.7	6.52	-
chr18	57307136	57307867	732	200	20	50.83	6.52	147372:57098171-57364644:Genebody
chr20	39762338	39762844	507	255	18	62.9	6.52	-
chr22	20457673	20458336	664	198	20	57.18	6.52	85376:20456002-20461786:Genebody
chr22	26800922	26801728	807	403	22	54.54	6.52	-
chr3	2436795	2437575	781	517	23	62.14	6.52	152330:2142246-3099644:Genebody
chr3	110636422	110637206	785	383	21	51.37	6.52	-
chr4	134208294	134209213	920	505	26	65.15	6.52	-
chr4	140585050	140585823	774	318	21	52.3	6.52	4258:140586921-140625404:Upstream
chr5	2968234	2968789	556	360	17	51.35	6.52	-
chr5	87254506	87255093	588	412	18	53.7	6.52	-
chr5	91953334	91953918	585	395	20	65.76	6.52	-
chr6	14542994	14543814	821	345	22	53.35	6.52	-
chr7	79570132	79570714	583	195	18	54.22	6.52	-

chr7	115306046	115306597	552	342	17	51.76	6.52	-
chr8	144940193	144940996	804	391	24	65.34	6.52	83481:144939911-144947434:Genebody
chrX	16038783	16039557	775	430	21	52.22	6.52	-
chr3	195211573	195214243	2671	1616	111	68.45	6.51	-
chr1	38449554	38450206	653	183	20	52.81	6.5	10946:38422653-38455761:Genebody
chr1	52494921	52495733	813	481	23	53.14	6.5	51060:52485806-52521047:Genebody
chr13	36451087	36451699	613	272	19	51.52	6.5	100302239:36048405-36515382:Genebody
chr14	54668650	54669433	784	496	22	50.8	6.5	-
chr14	65327017	65327887	871	466	24	52.99	6.5	-
chr17	47517034	47517685	652	249	20	52.91	6.5	-
chr3	127361318	127361868	551	194	18	52.59	6.5	50512:127348038-127391652:Genebody
chr3	166414773	166415476	704	286	22	58.28	6.5	-
chr6	116717406	116718122	717	473	24	62.3	6.5	29940:116601282-116759440:Genebody
chr6	158051449	158052138	690	491	21	54.41	6.5	79683:157802556-15809497

								6:Genebody
chr7	39245343	39245984	642	334	20	53.91	6.5	11281:39017608-39504390:Genebody
chr1	221974116	221975032	917	561	27	57.25	6.48	-
chr10	134495882	134497110	1229	753	41	90.84	6.48	3632:134351352-134596983:Genebody
chr19	11359308	11359916	609	339	20	52.35	6.48	57572:11309972-11373157:Genebody
chr2	23331909	23332249	341	171	14	55.07	6.48	-
chr2	167550678	167551320	643	446	21	53.98	6.48	-
chr4	190574733	190575679	947	504	27	54.67	6.48	-
chr8	138674514	138675268	755	484	23	52.93	6.48	-
chr3	66594399	66595071	673	437	22	51.09	6.47	-
chr8	58118270	58119171	902	179	51	120.32	6.46	-
chr1	11396942	11397848	907	502	32	69.68	6.45	-
chr1	54085159	54085780	622	420	18	60.42	6.41	148979:53971905-54199877:Genebody
chr1	61072306	61072956	651	179	20	69.82	6.41	-
chr1	154132882	154133600	719	431	19	57.13	6.41	7170:154127779-154155725:Genebody
chr1	191851237	191851702	466	271	14	51.78	6.41	-
chr1	196111262	196111838	577	286	16	52.77	6.41	-
chr11	103533849	103534427	579	276	16	52.58	6.41	-

chr12	5835847	5836505	659	224	17	51.07	6.41	57101:5671816-6055398:Genebody
chr12	23038568	23039168	601	163	16	50.52	6.41	-
chr12	108902578	108903222	645	230	17	52.31	6.41	-
chr16	60919520	60920160	641	236	20	70.91	6.41	-
chr2	49475069	49475663	595	298	16	51.07	6.41	-
chr21	29331286	29331802	517	319	15	52.67	6.41	-
chr22	27932656	27933294	639	443	17	52.85	6.41	-
chr22	50692268	50692807	540	198	15	50.37	6.41	6300:50691330-50700089:Genebody
chr22	51216156	51216730	575	220	16	52.97	6.41	284942:51195513-51238064:Genebody
chr3	12232379	12233119	741	276	19	55.21	6.41	6854:12045861-12233530:Genebody
chr3	31926306	31926949	644	299	17	52.39	6.41	114884:31702317-32023342:Genebody
chr3	105350773	105351540	768	353	20	58.48	6.41	-
chr3	160401389	160401989	601	275	16	50.52	6.41	-
chr3	190066719	190067381	663	224	20	68.54	6.41	-
chr4	119993912	119994646	735	310	18	50.22	6.41	-
chr4	139788243	139788818	576	408	18	65.3	6.41	-
chr5	165399359	165399929	571	299	16	53.36	6.41	-
chr6	75563293	75563946	654	464	17	51.51	6.41	-

chr7	14805276	14805945	670	370	17	50.12	6.41	1607:14184674-14881075:Genebody
chr7	29253973	29254549	577	292	16	52.77	6.41	1124:29234120-29553943:Genebody
chr7	128278987	128279734	748	388	19	54.61	6.41	402483:128281294-128301052:Upstream
chr8	23140803	23141429	627	436	18	59.91	6.41	-
chr8	75801084	75801666	583	410	16	52.2	6.41	-
chr9	86425230	86425995	766	481	20	58.65	6.41	80318:86354336-86432752:Genebody
chr1	36973187	36973794	608	377	17	50.09	6.4	-
chr1	59948800	59949442	643	369	18	52.37	6.4	55277:59762624-60228400:Genebody
chr1	90701727	90702358	632	226	20	65.08	6.4	-
chr1	197792742	197793479	738	276	20	54.66	6.4	-
chr1	210682693	210683474	782	384	20	50.92	6.4	55733:210501595-210849638:Genebody
chr11	29040483	29041274	792	393	21	55.28	6.4	-
chr12	44263534	44264337	804	360	23	65.03	6.4	84216:44229961-44783540:Genebody
chr13	80097297	80098046	750	435	22	64.6	6.4	54602:800552

								58-80130205:Genebody
chr17	45595518	45596347	830	297	24	68.14	6.4	-
chr2	128477104	128477876	773	435	20	51.66	6.4	55339:128461807-128568745:Genebody
chr2	163712723	163713319	597	431	17	51.14	6.4	-
chr3	107372461	107373158	698	432	19	52.83	6.4	56987:107241782-107530047:Genebody
chr3	184261251	184261881	631	392	18	53.51	6.4	-
chr4	70977131	70977894	764	318	21	57.74	6.4	-
chr5	177021817	177022260	444	264	15	55.6	6.4	54732:177019212-177023097:Genebody
chr7	34000824	34001561	738	402	22	65.79	6.4	168667:33945111-34194111:Genebody
chr7	128463189	128464205	1017	317	28	67.58	6.4	64753:128432098-128462183:Downstream
chr7	134992135	134992647	513	342	17	60.06	6.4	-
chr9	139463684	139464456	773	396	22	62.4	6.4	-
chr10	38811433	38812102	670	437	23	72.23	6.38	-
chr12	7721042	7721671	630	298	19	53.68	6.38	-
chr16	62553607	62554133	527	334	17	53.2	6.38	-
chr17	72564263	72565165	903	451	26	64.91	6.38	-

chr19	32970630	32971439	810	410	22	52.8	6.38	147991:32896654-32976797:Genebody
chr21	24906096	24906606	511	189	17	55	6.38	-
chr21	47643811	47644455	645	431	21	63.34	6.38	4047:47608360-47648738:Genebody
chr22	25496253	25497105	853	414	23	54.13	6.38	85379:25423940-25593413:Genebody
chr3	187314800	187315469	670	183	21	60.67	6.38	-
chr4	37213215	37213787	573	174	18	53.96	6.38	-
chr4	169776347	169777037	691	321	20	53.16	6.38	23022:169418216-169849607:Genebody
chr6	99096462	99097116	655	264	19	51.25	6.38	-
chr7	66030948	66031941	994	670	31	77.39	6.38	493754:66018552-66043498:Genebody
chr8	5736597	5737406	810	402	22	52.8	6.38	-
chrUn_gl000224	7695	8823	1129	650	90	92.86	6.38	-
chr1	45550277	45550879	603	380	19	51.32	6.37	57643:45482075-45672250:Genebody
chr10	33917931	33918595	665	237	20	50.38	6.37	-
chr10	121029508	121030020	513	331	19	61.62	6.37	2869:120967196-121215130

								:Genebody
chr21	43855649	43856316	668	329	22	60.61	6.37	53347:43824018-43867772:Genebody
chr7	63467754	63468440	687	233	21	53.37	6.37	-
chr8	97096387	97097157	771	398	23	55.52	6.37	-
chrY	58879614	58880611	998	215	58	109.41	6.37	-
chr11	60694103	60694998	896	345	27	57.64	6.36	54972:60691912-60704630:Genebody
chr12	48250681	48251186	506	336	18	51.98	6.36	7421:48235321-48298814:Genebody
chr22	30648678	30649253	576	329	20	50.25	6.36	-
chr5	95980489	95981112	624	448	21	50.09	6.36	-
chrY	58847057	58848522	1466	455	80	105.17	6.36	-
chr2	5649290	5650671	1382	715	50	93.6	6.35	-
chr2	727992	729123	1132	417	51	129.4	6.34	-
chr1	21223200	21223704	505	341	15	58.51	6.28	8672:21132975-21503340:Genebody
chr1	35893208	35893791	584	391	15	50.69	6.28	-
chr1	62595284	62595925	642	197	17	57.54	6.28	10207:62208148-62629589:Genebody
chr1	78259865	78260448	584	396	15	50.69	6.28	374986:78245308-78344077:Genebody

chr1	79093840	79094537	698	450	18	58.49	6.28	10964:79086087-79111828:Genebody
chr1	92638029	92638668	640	328	16	51.71	6.28	23285:92632608-92650279:Genebody
chr1	101504278	101504843	566	216	16	58.64	6.28	-
chr1	143760513	143761036	524	356	16	63.12	6.28	-
chr1	154054952	154055601	650	184	16	50.86	6.28	91181:153965169-154127592:Genebody
chr1	236578766	236579408	643	449	16	51.45	6.28	128178:236557679-236648008:Genebody
chr1	240131973	240132561	589	219	16	56.37	6.28	-
chr10	20238338	20238850	513	186	14	51.19	6.28	84898:20105371-20569115:Genebody
chr10	32175258	32175919	662	474	17	55.72	6.28	94134:32095224-32217770:Genebody
chr10	59388422	59389001	580	289	15	51.05	6.28	-
chr10	61555393	61555899	507	161	15	58.3	6.28	8030:61548521-61666818:Genebody
chr10	64182296	64182816	521	194	14	50.42	6.28	22891:64133915-64240047:Genebody

chr10	86891742	86892313	572	171	16	58.04	6.28	-
chr10	90320156	90320738	583	380	16	56.95	6.28	55328:90033620-90343082:Genebody
chr10	98327643	98328291	649	344	18	63.07	6.28	56889:98277867-98346809:Genebody
chr10	104084469	104084990	522	177	14	50.32	6.28	8729:104005308-104142649:Genebody
chr11	18939241	18939828	588	416	15	50.33	6.28	-
chr11	23483089	23483606	518	331	14	50.71	6.28	-
chr11	70088693	70089303	611	209	16	54.3	6.28	-
chr11	73422135	73422785	651	359	16	50.77	6.28	5870:73386689-73472201:Genebody
chr11	82275661	82276235	575	395	15	51.51	6.28	-
chr11	90909566	90910214	649	207	16	50.94	6.28	-
chr11	105228123	105228643	521	334	14	50.42	6.28	-
chr12	7295584	7296291	708	518	18	57.61	6.28	9746:7282966-7311528:Genebody
chr12	44785955	44786568	614	431	16	54.02	6.28	-
chr12	121055990	121056573	584	187	16	56.85	6.28	-
chr13	32997717	32998411	695	498	19	64.86	6.28	90634:32974860-33002315:Genebody
chr13	38544348	38544862	515	200	14	51	6.28	-

chr13	109256013	109256630	618	168	16	53.66	6.28	23026:109248499-109860355:Genebody
chr14	19509123	19509631	509	275	14	51.59	6.28	-
chr14	34515191	34515837	647	471	17	57.08	6.28	-
chr14	55811942	55812508	567	188	15	52.25	6.28	55030:55738020-55820327:Genebody
chr15	57564523	57565160	638	351	16	51.89	6.28	6938:57210832-57580712:Genebody
chr15	71021160	71021713	554	255	15	53.49	6.28	55075:70946894-71055850:Genebody
chr16	924818	925371	554	199	15	53.49	6.28	64788:903634-1020984:Genebody
chr16	68501487	68502073	587	297	16	56.56	6.28	-
chr16	87867608	87868125	518	331	15	57.12	6.28	8140:87863629-87903100:Genebody
chr17	4559314	4559932	619	190	16	53.57	6.28	-
chr17	5745313	5745902	590	198	15	50.15	6.28	-
chr17	27163273	27163784	512	179	14	51.29	6.28	55731:27082995-27169841:Genebody
chr17	39124186	39124796	611	183	16	54.3	6.28	390792:39114668-39123144

								:Upstream
chr17	47178905	47179545	641	406	16	51.63	6.28	-
chr17	50675980	50676574	595	401	16	55.79	6.28	-
chr17	65995277	65995837	561	190	15	52.82	6.28	-
chr18	3082043	3082667	625	457	16	53.03	6.28	8736:3066805-3220106:Genebody
chr18	72334597	72335237	641	450	17	57.63	6.28	-
chr19	14964528	14965037	510	192	14	51.49	6.28	-
chr19	19773475	19774092	618	293	16	53.66	6.28	57130:19756011-19774503:Genebody
chr19	37322902	37323537	636	453	16	52.06	6.28	388536:37308332-37329284:Genebody
chr19	47620613	47621228	616	231	16	53.84	6.28	-
chr2	19225373	19226055	683	233	17	53.89	6.28	-
chr2	21982915	21983502	588	435	15	50.33	6.28	-
chr2	55860005	55860502	498	222	14	52.7	6.28	87178:55861197-55921011:Downstream
chr2	57567389	57567904	516	185	14	50.9	6.28	-
chr2	91651207	91651734	528	346	15	56.08	6.28	-
chr2	96464712	96465265	554	214	15	53.49	6.28	-
chr2	100466372	100466892	521	340	14	50.42	6.28	3899:100163717-100722045:Genebody
chr2	106724266	106724899	634	399	17	58.28	6.28	80146:106709

								759-10681079 5:Genebody
chr2	108999967	109000455	489	338	14	53.63	6.28	27233:108994 420-10900427 0:Genebody
chr2	149882901	149883418	518	210	14	50.71	6.28	-
chr2	153772306	153772980	675	263	18	60.59	6.28	-
chr2	228071141	228071747	607	193	16	54.67	6.28	1285:2280292 80-228179507 :Genebody
chr2	235847709	235848433	725	400	18	56.15	6.28	-
chr20	16513623	16514141	519	323	14	50.61	6.28	55614:162527 48-16554078: Genebody
chr20	18067282	18067864	583	197	15	50.78	6.28	-
chr20	37259192	37259822	631	198	16	52.5	6.28	343578:37230 576-37279295 :Genebody
chr20	56338981	56339563	583	323	16	56.95	6.28	-
chr21	37279011	37279663	653	189	16	50.6	6.28	-
chr3	24006994	24007544	551	193	15	53.78	6.28	9975:2398675 0-24022109:G enebody
chr3	43765840	43766325	486	336	14	53.94	6.28	51099:437323 74-43764216: Downstream
chr3	48932899	48933552	654	220	19	68.94	6.28	788:48894356 -48936402:Ge

								nebody
chr3	147148973	147149558	586	388	15	50.51	6.28	-
chr3	151455220	151455744	525	226	14	50.03	6.28	344752:151451703-151475554:Genebody
chr3	195816823	195817444	622	322	16	53.3	6.28	-
chr4	7978041	7978715	675	420	17	54.58	6.28	84448:7967037-8160559:Genebody
chr4	36265722	36266291	570	186	15	51.97	6.28	-
chr4	38744089	38744660	572	164	15	51.79	6.28	-
chr4	55274668	55275239	572	195	15	51.79	6.28	-
chr4	77012159	77012793	635	454	16	52.15	6.28	419:76932336-77033954:Genebody
chr4	105000336	105000937	602	325	16	55.13	6.28	-
chr4	107917260	107917849	590	302	16	56.27	6.28	27123:107842959-107957453:Genebody
chr4	116627932	116628509	578	395	15	51.24	6.28	-
chr4	118566611	118567174	564	356	15	52.54	6.28	-
chr4	130774089	130774662	574	424	15	51.6	6.28	-
chr4	131796176	131796803	628	437	16	52.76	6.28	-
chr4	137940108	137940617	510	198	14	51.49	6.28	-
chr5	53441147	53441815	669	194	18	61.15	6.28	54622:53180613-53606403:Genebody
chr5	129380911	129381501	591	264	15	50.07	6.28	337876:12924

								0522-129522326:Genebody
chr5	129776388	129776960	573	206	16	57.94	6.28	-
chr5	137438108	137438761	654	350	16	50.52	6.28	-
chr5	142421429	142422041	613	187	16	54.11	6.28	23092:142150291-142608571:Genebody
chr5	147555674	147556265	592	402	17	62.42	6.28	408187:147549295-147554961:Downstream
chr6	46594293	46594807	515	331	14	51	6.28	51302:46517444-46620523:Genebody
chr6	57852921	57853498	578	400	15	51.24	6.28	-
chr6	93618799	93619321	523	347	14	50.22	6.28	-
chr6	94083799	94084418	620	409	16	53.48	6.28	2045:93949741-94129300:Genebody
chr6	107613595	107614135	541	190	15	54.77	6.28	57107:107473760-107780779:Genebody
chr6	150432137	150432712	576	388	15	51.42	6.28	-
chr6	152349240	152349826	587	390	16	56.56	6.28	2099:152011630-152424406:Genebody
chr6	156408656	156409191	536	366	15	55.27	6.28	-
chr7	9660503	9661129	627	433	17	58.95	6.28	-

chr7	40837138	40837647	510	361	15	57.97	6.28	79783:40174574-40900357:Genebody
chr7	70553310	70553896	587	261	15	50.42	6.28	-
chr7	75518419	75518905	487	237	14	53.84	6.28	57414:75508316-75518244:Downstream
chr7	82429364	82429946	583	337	15	50.78	6.28	27445:82383320-82792197:Genebody
chr7	93461558	93462206	649	200	18	63.07	6.28	-
chr7	96482537	96483267	731	371	19	61.53	6.28	-
chr7	101223615	101224276	662	200	18	61.81	6.28	-
chr7	116850089	116850610	522	328	14	50.32	6.28	7982:116593380-116863955:Genebody
chr8	34826768	34827323	556	359	15	53.3	6.28	-
chr8	47286097	47286681	585	390	15	50.6	6.28	-
chr8	56182812	56183398	587	196	16	56.56	6.28	114786:56015016-56438708:Genebody
chr8	57470804	57471450	647	247	16	51.11	6.28	-
chr8	60302789	60303439	651	361	16	50.77	6.28	-
chr8	93157555	93158181	627	290	18	65.27	6.28	-
chr8	109196709	109197285	577	187	16	57.54	6.28	-
chr8	122955616	122956117	502	192	14	52.29	6.28	-
chr9	9788882	9789471	590	273	15	50.15	6.28	-
chr9	13907660	13908293	634	238	16	52.23	6.28	-

chr9	25114408	25115030	623	312	16	53.21	6.28	-
chrY	14198070	14198668	599	430	16	55.41	6.28	-
chr1	12080252	12080951	700	187	18	58.04	6.25	60672:12079511-12092106:Genebody
chr1	39395840	39396430	591	392	17	62.25	6.25	54933:39351478-39407456:Genebody
chr1	65627226	65627815	590	396	16	56.02	6.25	NM_203464:65613231-65697826:Genebody
chr1	71536494	71537244	751	347	22	62.82	6.25	9406:71528974-71546745:Genebody
chr1	80899889	80900498	610	373	22	60.42	6.25	-
chr1	80911551	80912275	725	430	24	77.2	6.25	-
chr1	85031472	85032206	735	358	21	58.82	6.25	64173:84971973-85031875:Genebody
chr1	101596608	101597185	578	299	15	51	6.25	-
chr1	155017966	155018622	657	395	16	50.02	6.25	149095:155006299-155023406:Genebody
chr1	193295780	193296685	906	351	26	56.52	6.25	-
chr1	206698496	206699089	594	199	16	55.64	6.25	83593:206680878-206762615:Genebody

chr10	3844646	3845206	561	298	19	54.61	6.25	-
chr10	21509616	21510319	704	523	19	50.87	6.25	-
chr10	57236774	57237238	465	266	14	50.61	6.25	-
chr10	69604348	69604861	514	177	14	50.87	6.25	-
chr10	73699290	73699887	598	253	17	55.24	6.25	-
chr10	110692414	110692898	485	275	16	55.75	6.25	-
chr10	111863247	111863829	583	212	15	50.55	6.25	120:111765725-111895320:Genebody
chr10	115419827	115420512	686	164	20	64.41	6.25	4892:115348583-115423805:Genebody
chr11	22203780	22204478	699	470	21	50.93	6.25	-
chr11	31509113	31509611	499	289	15	53.21	6.25	196294:31453948-31531169:Genebody
chr11	37946603	37947361	759	518	22	69.08	6.25	-
chr11	40047274	40047817	544	176	15	54.23	6.25	-
chr11	64650785	64651514	730	463	22	58.6	6.25	-
chr11	64985742	64986412	671	243	18	60.68	6.25	440044:64981310-64993511:Genebody
chr11	72434613	72435460	848	381	23	53.05	6.25	116985:72396114-72433403:Upstream
chr11	73870841	73871550	710	414	20	50.07	6.25	26005:73745479-73882064:Genebody

chr11	106212911	106213384	474	171	16	50.88	6.25	-
chr11	108899207	108900044	838	483	24	58.89	6.25	-
chr11	114016753	114017411	659	188	19	61.13	6.25	7704:113930430-114121394:Genebody
chr12	22410639	22411260	622	425	20	64.59	6.25	6489:22346325-22487648:Genebody
chr12	40433333	40434221	889	489	24	54.49	6.25	114134:40148824-40499661:Genebody
chr12	53436834	53437660	827	389	23	68.4	6.25	1975:53400061-53435992:Downstream
chr12	92561985	92562631	647	194	17	50.67	6.25	-
chr12	101374426	101375069	644	453	19	62.64	6.25	121601:101188373-101522419:Genebody
chr13	24804191	24804900	710	392	17	51.38	6.25	221178:24734860-24881212:Genebody
chr13	35698697	35699325	629	198	16	52.43	6.25	26960:35516423-36246872:Genebody
chr13	40979145	40979729	585	274	20	62.83	6.25	646982:40921272-41055143:Genebody
chr13	92248841	92249342	502	199	14	52.06	6.25	2262:9205093

								4-93519485:G<br>enebody
chr13	95479681	95480197	517	197	14	50.58	6.25	-
chr13	99745089	99745719	631	440	17	58.3	6.25	-
chr14	19393190	19393886	697	181	21	62.52	6.25	-
chr14	19979256	19979998	743	257	19	60.17	6.25	-
chr14	34403017	34403569	553	184	15	53.35	6.25	112399:34393<br>422-34420284<br>:Genebody
chr14	63800420	63801137	718	313	17	50.74	6.25	-
chr14	86377762	86378513	752	286	23	55.91	6.25	-
chr14	93419454	93420053	600	252	16	55.07	6.25	3705:9340325<br>8-93582263:G<br>enebody
chr14	96652932	96653578	647	384	16	50.87	6.25	-
chr15	46438686	46439423	738	364	20	59.45	6.25	-
chr16	11328344	11328972	629	397	17	52.29	6.25	-
chr16	27365506	27366104	599	276	19	55.53	6.25	3566:2732525<br>0-27366530:G<br>enebody
chr16	32062702	32063230	529	198	21	60.12	6.25	-
chr16	33664617	33665131	515	321	15	57.2	6.25	-
chr16	62134293	62134836	544	237	16	54.65	6.25	-
chr16	62757719	62758494	776	305	25	58.65	6.25	-
chr17	5681847	5682423	577	198	18	52.27	6.25	-
chr17	16738213	16738857	645	304	17	50.85	6.25	-
chr17	49336143	49336764	622	367	20	64.59	6.25	54799:492547<br>87-49337427:

								Genebody
chr17	49774502	49775086	585	180	15	50.37	6.25	56934:49707674-50236132:Genebody
chr18	15352121	15352818	698	510	19	57.4	6.25	-
chr18	30670531	30671082	552	158	17	60.01	6.25	374864:30517365-31020045:Genebody
chr18	40554854	40555541	688	489	20	57.77	6.25	6014:40323192-40695657:Genebody
chr18	71362058	71362609	552	245	15	53.45	6.25	-
chr18	73898989	73899682	694	395	17	52.7	6.25	-
chr19	17534201	17535037	837	415	24	53.04	6.25	93343:17530911-17536140:Genebody
chr19	18261197	18261809	613	341	22	59.82	6.25	23031:18208602-18262498:Genebody
chr19	20641072	20641577	506	278	14	51.66	6.25	-
chr19	56915910	56916676	767	395	23	60.32	6.25	147949:56915382-56936400:Genebody
chr2	9997183	9997781	599	195	18	50.04	6.25	9014:9983570-10074543:Genebody
chr2	58060375	58061054	680	185	20	65.02	6.25	-
chr2	82147420	82148058	639	351	18	51.37	6.25	-

chr2	90372432	90373467	1036	540	30	57.61	6.25	-
chr2	91869831	91870379	549	156	17	66.79	6.25	-
chr2	113652034	113652769	736	413	22	52.43	6.25	-
chr2	152991210	152991797	588	391	15	50.1	6.25	10254:152973315-153032506:Genebody
chr2	174379417	174380092	676	327	20	53.21	6.25	-
chr2	176617307	176618105	799	203	25	75.07	6.25	-
chr2	178520766	178521734	969	572	28	75.13	6.25	50940:178487978-178753466:Genebody
chr2	236765590	236766262	673	433	20	53.5	6.25	116987:236402735-237034114:Genebody
chr20	31905437	31906150	714	274	19	50	6.25	-
chr20	33892065	33892802	738	444	22	71.19	6.25	55245:33890370-33999945:Genebody
chr20	41131285	41131946	662	229	19	60.83	6.25	11122:40701392-41818557:Genebody
chr20	52364584	52365167	584	183	16	50.71	6.25	-
chr20	52559207	52559922	716	355	18	50.27	6.25	8537:52560078-52687304:Downstream
chr21	22319372	22320196	825	565	27	76.23	6.25	-
chr21	37723745	37724437	693	478	22	56.62	6.25	23515:37692486-37748943:

								Genebody
chr21	42185914	42186538	625	418	16	52.78	6.25	1826:41384342-42219039:Genebody
chr22	19418262	19418895	634	188	18	51.84	6.25	7290:19318223-19419219:Genebody
chr22	42868384	42869051	668	196	17	54.93	6.25	-
chr3	10915429	10916083	655	457	25	77.74	6.25	6538:10857916-10980144:Genebody
chr3	17329736	17330672	937	418	26	53.9	6.25	9779:17198654-17741512:Genebody
chr3	24537250	24537879	630	183	17	58.4	6.25	7068:24158646-24536266:Upstream
chr3	75559624	75560523	900	708	28	68.63	6.25	-
chr3	96650229	96651050	822	415	22	56.36	6.25	285220:96533424-97467786:Genebody
chr3	98427807	98428331	525	315	16	51.32	6.25	-
chr3	101957083	101957587	505	290	18	51.02	6.25	-
chr3	128452146	128452920	775	478	19	50.82	6.25	7879:128444978-128533641:Genebody
chr3	134952196	134952840	645	461	17	50.85	6.25	2047:134514098-134979305

								:Genebody
chr3	148819931	148820575	645	450	18	50.81	6.25	-
chr3	162397560	162398227	668	231	19	60.24	6.25	-
chr3	183506014	183506663	650	315	20	50.59	6.25	55689:183415605-183530413:Genebody
chr3	194257728	194258242	515	193	16	52.39	6.25	-
chr4	23756055	23756696	642	473	17	57.27	6.25	-
chr4	24165794	24166448	655	391	16	50.19	6.25	-
chr4	26104084	26104714	631	198	17	52.11	6.25	-
chr4	32614319	32614950	632	436	17	52.02	6.25	-
chr4	72173611	72174509	899	514	25	52.45	6.25	8671:72053002-72437803:Genebody
chr4	89764462	89765255	794	473	22	52.72	6.25	10144:89647105-89978323:Genebody
chr4	98927537	98928274	738	327	21	65.23	6.25	285555:98480026-99064391:Genebody
chr4	119891330	119891919	590	240	18	50.94	6.25	171024:119809995-119959668:Genebody
chr4	150327101	150327854	754	455	19	52.52	6.25	-
chr4	152189182	152189730	549	276	18	50.46	6.25	-
chr4	173233030	173233662	633	392	16	52.08	6.25	442117:172734574-173961556:Genebody

chr4	182161658	182162125	468	146	14	50.29	6.25	-
chr5	1222161	1223127	967	436	34	76.26	6.25	340024:1201709-1225228:Genebody
chr5	59057926	59058682	757	307	26	65.01	6.25	5144:58264865-59189621:Genebody
chr5	81418368	81419107	740	449	20	52.99	6.25	83734:81267843-81551211:Genebody
chr5	131006498	131007312	815	342	21	58.27	6.25	96459:130977406-131132756:Genebody
chr5	134131891	134132516	626	265	20	58.26	6.25	9879:134094460-134166809:Genebody
chr5	144081449	144082105	657	249	16	50.02	6.25	-
chr5	158024321	158024839	519	191	15	51.12	6.25	-
chr6	34495586	34496154	569	379	20	77.64	6.25	29993:34433904-34502998:Genebody
chr6	78823948	78824545	598	402	16	55.26	6.25	-
chr6	119908830	119909388	559	368	18	54.19	6.25	-
chr6	130420787	130421559	773	401	21	55.37	6.25	84456:130339733-130462584:Genebody
chr6	164348304	164349218	915	500	29	63.84	6.25	-
chr6	168405960	168406493	534	182	16	55.7	6.25	-

chr7	57187478	57188479	1002	562	30	60.52	6.25	90827:57187327-57207571:Genebody
chr7	84290968	84291540	573	195	15	51.46	6.25	-
chr7	102218931	102219467	537	248	26	59.97	6.25	10156:102220093-102257205:Downstream
chr7	136714953	136715700	748	430	23	62.19	6.25	-
chr8	7404846	7405529	684	432	17	53.54	6.25	-
chr8	16226995	16227504	510	260	17	53.89	6.25	-
chr8	47651561	47652261	701	199	22	55.81	6.25	-
chr8	50752521	50753161	641	250	17	51.2	6.25	-
chr8	102144054	102144648	595	405	18	55.69	6.25	-
chr9	514402	515089	688	197	17	53.2	6.25	23189:504702-746103:Genebody
chr9	9591634	9592284	651	338	17	50.32	6.25	5789:8314246-10612723:Genebody
chr9	38138671	38139202	532	243	16	55.91	6.25	-
chr9	80160237	80160934	698	381	18	51.79	6.25	9630:80037995-80263232:Genebody
chr9	82137596	82138240	645	200	18	50.81	6.25	-
chr9	92331697	92332563	867	481	26	60.01	6.25	100129066:92254697-92334674:Genebod

								y
chr9	122517739	122518318	580	301	16	51.08	6.25	-
chr9	127270730	127271224	495	191	14	52.78	6.25	2516:127243514-127269699:Upstream
chr9	131947607	131948097	491	245	16	54.08	6.25	-
chr3	49170200	49171418	1219	638	58	81.35	6.19	3913:49158547-49170599:Upstream
chr9_gl000199_random	49384	50365	982	459	76	101.06	6.18	-
chr11	43436560	43437491	932	384	31	70.26	6.14	55761:43380434-43516482:Genebody
chr14	52764372	52765125	754	567	25	64.57	6.13	-
chr16	1504252	1505001	750	480	24	59.79	6.13	1186:1494934-1525085:Genebody
chr20	30182195	30182862	668	217	22	57.87	6.13	-
chr6	45498217	45498918	702	335	24	64.87	6.13	860:45296053-45518818:Genebody
chr6	146221425	146222245	821	322	27	68.01	6.13	257218:146205946-146285233:Genebody
chr18	40897401	40898059	659	464	22	64.51	6.12	-
chr2	69195868	69196461	594	198	19	54.76	6.12	-
chr2	227302502	227303095	594	191	19	54.76	6.12	-

chr20	42192940	42193663	724	532	23	63.08	6.12	10110:42187685-42214272:Genebody
chr6	128342769	128343376	608	311	20	58.84	6.12	5796:128289924-128841819:Genebody
chr8	54991674	54992430	757	468	23	59.77	6.12	10434:54958938-55014577:Genebody
chr9	92073142	92074101	960	524	28	66.4	6.12	10507:91975705-92094611:Genebody
chr10	1099866	1100675	810	289	22	55.82	6.11	-
chr13	28321572	28322383	812	288	23	60.88	6.11	-
chr13	33674876	33675405	530	331	17	55.41	6.11	-
chr13	55831012	55831635	624	187	18	51.45	6.11	-
chr15	80395326	80395988	663	474	19	53.2	6.11	54469:80352020-80430710:Genebody
chr17	20651238	20651963	726	460	20	52.77	6.11	-
chr17	32479451	32480031	581	192	17	50.09	6.11	40:31340107-32483825:Genebody
chr17	75241392	75242877	1486	915	64	95.36	6.11	-
chr2	4635650	4636243	594	225	18	54.42	6.11	-
chr2	45057319	45058109	791	472	21	52.32	6.11	-
chr2	65145731	65146338	608	197	18	53.01	6.11	-
chr2	192027432	192028188	757	444	20	50.1	6.11	-

chr3	76262791	76263548	758	463	21	55.18	6.11	-
chr5	78375925	78376587	663	277	19	53.2	6.11	23743:78365546-78385897:Genebody
chr6	170521291	170522003	713	374	22	64.98	6.11	-
chr7	91689902	91690768	867	387	23	56.08	6.11	10142:91570188-91739986:Genebody
chr8	139756608	139757322	715	428	20	53.76	6.11	169044:139600478-139926236:Genebody
chr1	158476315	158477178	864	455	23	63.19	6.1	-
chr1	233919768	233920420	653	401	18	54.32	6.1	-
chr10	82923750	82924467	718	263	19	54.04	6.1	-
chr10	101823322	101824063	742	405	20	57.44	6.1	1369:101802064-101841642:Genebody
chr12	48316502	48317099	598	220	17	53.79	6.1	-
chr12	88107083	88107653	571	380	16	50.59	6.1	-
chr16	81461878	81462496	619	246	17	51.79	6.1	-
chr17	17639108	17639744	637	337	18	55.83	6.1	10743:17584786-17714763:Genebody
chr18	68358369	68359134	766	398	24	72.44	6.1	-
chr2	21263504	21264275	772	438	20	54.83	6.1	338:21224301-21266945:Genebody
chr20	7995695	7996294	600	198	17	53.6	6.1	56255:796171

								5-8000393:Genebody
chr20	57162996	57163529	534	184	16	54.31	6.1	-
chr22	16123054	16123685	632	387	20	68.39	6.1	-
chr3	25446775	25447456	682	321	18	51.7	6.1	-
chr3	120863208	120863887	680	382	18	51.87	6.1	9515:120627049-121143607:Genebody
chr4	34059807	34060366	560	319	18	63.9	6.1	-
chr4	37977491	37978164	674	387	18	52.41	6.1	23216:37892719-38140793:Genebody
chr6	41484810	41485464	655	465	18	54.13	6.1	-
chr10	701342	702381	1040	554	36	51.73	6.09	22982:320131-735608:Genebody
chr1	81113327	81113931	605	423	16	53.07	6.08	-
chr1	113644060	113644538	479	180	14	53.06	6.08	9860:113615830-113667342:Genebody
chr1	244806333	244806847	515	350	16	62.27	6.08	-
chr10	62351267	62351961	695	400	20	68.92	6.08	-
chr12	53586294	53586907	614	154	17	58.3	6.08	283337:53574534-53584653:Downstream
chr13	114922847	114923393	547	320	15	52.47	6.08	-
chr14	39884268	39884937	670	299	17	53.15	6.08	254170:39866877-39901704

								:Genebody
chr16	12959667	12960261	595	200	17	60.18	6.08	-
chr18	1421014	1421640	627	195	16	51.09	6.08	-
chr2	28748354	28748873	520	193	15	55.18	6.08	151056:28718937-28866651:Genebody
chr2	82430362	82430944	583	413	16	55.15	6.08	-
chr2	92057273	92057875	603	253	16	53.25	6.08	-
chr2	125544975	125545651	677	319	18	58.41	6.08	129684:124782863-125672861:Genebody
chr22	29728587	29729093	507	191	14	50.18	6.08	162:29723670-29784572:Genebody
chr3	2125862	2126491	630	234	16	50.82	6.08	-
chr3	157437476	157437989	514	331	15	55.81	6.08	-
chr4	88690522	88691166	645	192	17	55.38	6.08	-
chr4	91118026	91118745	720	425	19	60.44	6.08	401145:91048683-92523369:Genebody
chr5	99224089	99224719	631	420	16	50.74	6.08	-
chr5	118001923	118002585	663	278	18	59.71	6.08	-
chr6	125156062	125156620	559	296	15	51.32	6.08	-
chr7	67680139	67680752	614	424	16	52.25	6.08	-
chr7	126399605	126400290	686	199	18	57.59	6.08	2918:126078651-126883569:Genebody
chr8	35422012	35422618	607	330	16	52.88	6.08	137970:35092

								974-35652180:Genebody
chr9	19298197	19298888	692	388	17	51.29	6.08	55667:19290748-19374138:Genebody
chr9	34202685	34203286	602	199	16	53.34	6.08	51271:34179002-34252520:Genebody
chr1	121482560	121485530	2971	2664	1261	643.72	6.07	-
chr8	58125276	58126030	755	328	36	55.72	6.07	-
chrY	58821964	58822367	404	200	27	63.41	6.07	-
chr1	228531199	228532398	1200	497	42	78.8	6.06	84033:228395860-228548950:Genebody
chr10	24147769	24148335	567	230	21	57.67	6.03	56243:23983674-24836771:Genebody
chr13	112629334	112630039	706	232	27	73.27	6.03	-
chr18	76308974	76310335	1362	506	39	62.1	6.03	-
chr4	146504902	146505488	587	283	20	50.13	6.03	-
chr6	57240500	57241016	517	188	19	52.89	6.03	5558:57182421-57513375:Genebody
chr6	170021056	170022293	1238	726	41	81.38	6.03	253769:169857306-170102159:Genebody
chr12	74392231	74393138	908	382	26	53.42	6.02	-
chr19	45535905	45536737	833	532	31	81.05	6.02	5971:4550471

								1-45541452:Genebody
chr2	68819204	68819910	707	446	22	52.6	6.02	-
chr2	238053110	238053724	615	176	20	51.72	6.02	-
chr3	158997647	158998177	531	337	19	55.7	6.02	29970:158991543-159615139:Genebody
chr3	172886005	172886714	710	433	22	52.31	6.02	-
chr6	87484858	87485622	765	308	24	56.89	6.02	-
chr7	56439199	56439888	690	279	31	67.83	6.01	-
chr7	61240544	61241286	743	268	34	75.06	6.01	-
chr11	19453905	19454657	753	393	30	59.95	6	89797:19372270-20143146:Genebody
chr13	35355576	35356404	829	476	24	56.64	6	-
chr17	21903966	21905305	1340	828	95	93.34	6	284124:21904061-21913070:Genebody
chr5	58154743	58155629	887	457	25	56.42	6	-
chr6	64386518	64387295	778	225	23	56.29	6	23469:64356430-64424405:Genebody
chr6	76399254	76399788	535	337	20	66.17	6	26054:76311621-76427993:Genebody
chr7	40151357	40152013	657	242	23	68.94	6	-
chr8	95952058	95952522	465	199	16	50.97	6	94241:95938200-95961615:

								Genebody
chr1	5006170	5006832	663	487	21	62.92	5.98	-
chr1	11680864	11681478	615	417	18	51	5.98	-
chr1	52188264	52188870	607	194	21	69.24	5.98	114883:52082763-52254136:Genebody
chr1	192631797	192632600	804	417	23	59.98	5.98	-
chr1	213658780	213659404	625	433	18	50.04	5.98	-
chr12	4153218	4153939	722	237	20	51.71	5.98	-
chr12	10236538	10237373	836	390	22	52.11	5.98	51267:10223081-10251605:Genebody
chr12	123323410	123324304	895	356	24	57.14	5.98	9026:123320038-123347507:Genebody
chr16	71523830	71524518	689	460	20	54.74	5.98	7567:71507975-71523254:Upstream
chr18	9092697	9093282	586	198	18	53.91	5.98	-
chr18	39893819	39894366	548	332	17	52.18	5.98	-
chr18	58075536	58076067	532	182	17	53.9	5.98	-
chr2	27225543	27226133	591	430	18	53.39	5.98	22924:27193524-27250086:Genebody
chr3	55820116	55820836	721	270	20	51.8	5.98	26059:55542335-56502391:Genebody
chr3	57720813	57721511	699	390	20	53.8	5.98	-

chr3	128402470	128403125	656	410	19	52.49	5.98	-
chr3	173836343	173837176	834	442	28	84.88	5.98	22871:173116243-174001116:Genebody
chr3	197501050	197501697	648	197	21	64.54	5.98	84248:197476423-197511317:Genebody
chr4	3590926	3591824	899	542	25	61.85	5.98	-
chr4	19772122	19772992	871	442	23	54.17	5.98	-
chr5	32347733	32348221	489	278	16	52.8	5.98	-
chr5	133955075	133955539	465	199	16	55.62	5.98	51128:133936840-133968533:Genebody
chr6	88418211	88418954	744	464	21	54.95	5.98	-
chr8	119763022	119763674	653	439	20	58.29	5.98	-
chr9	33393123	33393868	746	475	21	54.77	5.98	364:33384948-33402517:Genebody
chr1	21983197	21983927	731	549	21	62.45	5.95	5909:21922708-21995856:Genebody
chr1	66084567	66085078	512	337	16	55.32	5.95	3953:65886247-66096093:Genebody
chr1	150923255	150924032	778	409	20	52.76	5.95	9869:150898814-150937220:Genebody
chr1	162721322	162721881	560	198	16	50.33	5.95	4921:1626022

								27-162750237 :Genebody
chr10	100159965	100160893	929	543	30	56.85	5.95	84795:100143 322-10017497 8:Genebody
chr10	111518193	111518951	759	286	22	65.45	5.95	-
chr11	40441308	40441859	552	360	16	51.12	5.95	-
chr11	42986420	42987199	780	375	20	52.6	5.95	-
chr11	121802405	121802908	504	318	16	56.21	5.95	-
chr12	24905554	24906208	655	195	18	52.68	5.95	-
chr12	55505551	55506132	582	184	17	53.97	5.95	-
chr12	106091289	106091907	619	281	17	50.41	5.95	-
chr14	98831749	98832448	700	275	19	54.12	5.95	-
chr16	69365506	69366059	554	379	16	50.92	5.95	64146:693625 23-69364498: Upstream
chr17	11787740	11788326	587	151	17	53.47	5.95	1770:1150174 7-11873063:G enebody
chr17	57317827	57318495	669	495	19	57	5.95	284161:57297 827-57350381 :Genebody
chr17	63978662	63979294	633	244	19	60.57	5.95	201134:63631 658-64187987 :Genebody
chr18	2886292	2886925	634	455	18	54.65	5.95	84034:284702 7-2914088:Ge nebody

chr2	134306493	134307074	582	197	18	59.94	5.95	344148:133429372-134326031:Genebody
chr2	140968067	140968723	657	323	18	52.49	5.95	-
chr2	193966534	193967381	848	479	23	62.79	5.95	-
chr2	215947680	215948277	598	416	17	52.39	5.95	26154:215796266-216003151:Genebody
chr2	233410344	233410896	553	197	16	51.02	5.95	1146:233404436-233411038:Genebody
chr21	16921254	16921798	545	274	16	51.83	5.95	-
chr21	33684147	33685007	861	322	22	56.37	5.95	56246:33664123-33684597:Genebody
chr3	93884239	93884793	555	351	16	50.82	5.95	-
chr4	30534979	30535656	678	506	18	50.61	5.95	-
chr4	142863775	142864556	782	537	20	52.43	5.95	-
chr4	188877335	188878047	713	323	20	58.49	5.95	-
chr5	75553030	75553942	913	411	30	58.35	5.95	22987:75379304-75621416:Genebody
chr6	20754464	20755132	669	199	19	57	5.95	54901:20534687-21232632:Genebody
chr6	28316342	28316964	623	424	17	50.04	5.95	64288:28292516-28321972:Genebody

chr6	159333481	159334148	668	369	18	51.5	5.95	-
chr7	24244154	24244663	510	336	16	55.54	5.95	-
chr7	65890935	65891683	749	496	26	56.4	5.95	-
chr7	148681155	148681764	610	174	19	63	5.95	-
chr8	38784690	38785310	621	440	18	55.92	5.95	59339:38758752-38831428:Genebody
chr8	116171077	116171675	599	402	17	52.3	5.95	-
chr16	46949300	46949974	675	236	23	54.6	5.93	84706:46918307-46965200:Genebody
chr17	41399858	41401589	1732	771	226	346.69	5.93	-
chr6	12883960	12884612	653	256	22	51.98	5.93	221692:12717832-13287528:Genebody
chr7	148954996	148955825	830	460	26	53.52	5.93	-
chr1	86093325	86093879	555	163	15	50.29	5.92	-
chr1	145399947	145400436	490	245	14	50.56	5.92	-
chr1	167746404	167747031	628	333	17	55.38	5.92	9019:167691186-167761155:Genebody
chr10	8855249	8855924	676	373	17	51.1	5.92	-
chr10	27060991	27061533	543	384	15	51.45	5.92	10006:27035526-27150016:Genebody
chr10	130011412	130011977	566	191	16	55.31	5.92	-
chr13	67008941	67009523	583	392	16	53.65	5.92	5101:66876966-67804468:G

								enebody
chr14	28229282	28229817	536	199	15	52.13	5.92	-
chr15	71098061	71098725	665	218	19	63.91	5.92	-
chr16	65705614	65706294	681	200	19	62.34	5.92	-
chr17	72348647	72349318	672	428	17	51.44	5.92	124602:72322350-72351958:Genebody
chr19	12691098	12691709	612	192	16	50.95	5.92	57474:12686919-12721623:Genebody
chr19	17683324	17683925	602	302	16	51.86	5.92	79709:17666510-17693965:Genebody
chr19	21555796	21556341	546	209	15	51.15	5.92	148203:21541734-21571383:Genebody
chr2	156745742	156746255	514	372	16	60.83	5.92	-
chr2	174326428	174326985	558	149	16	56.12	5.92	-
chr3	36039757	36040350	594	197	16	52.6	5.92	-
chr3	66515058	66515669	612	414	16	50.95	5.92	26018:66429221-66550845:Genebody
chr3	119230282	119230935	654	438	17	53.01	5.92	51300:119217367-119243123:Genebody
chr4	21647442	21648133	692	376	18	55.42	5.92	80333:20730238-21699318:Genebody

chr4	33148479	33149053	575	209	16	54.42	5.92	-
chr4	69072421	69073000	580	227	16	53.94	5.92	100130017:69048009-69078188:Genebody
chr4	104297468	104298244	777	532	20	59.44	5.92	-
chr4	135147864	135148523	660	231	19	64.41	5.92	-
chr4	139524208	139524854	647	309	17	53.63	5.92	-
chr4	163092529	163093135	607	445	16	51.4	5.92	-
chr5	14649149	14649734	586	259	16	53.36	5.92	-
chr5	138783990	138784547	558	191	15	50.01	5.92	641700:138784244-138842320:Genebody
chr6	39077721	39078325	605	406	16	51.59	5.92	55776:39071839-39082865:Genebody
chr7	143870776	143871384	609	195	16	51.22	5.92	-
chr7	157539091	157539786	696	456	19	60.91	5.92	5799:157331750-158380482:Genebody
chr9	4104259	4104869	611	422	16	51.04	5.92	169792:3824127-4152183:Genebody
chr9	125540899	125541528	630	453	17	55.19	5.92	-
chr1	1887607	1888476	870	461	25	50.72	5.91	85452:1884751-1935276:Genebody
chr1	59195319	59195988	670	198	25	65.62	5.91	-

chr1	88417542	88418227	686	197	23	58.52	5.91	-
chr10	105507801	105508559	759	287	26	66.28	5.91	9644:105353783-105615164:Genebody
chr12	66026467	66027350	884	415	28	63.55	5.91	-
chr12	78605557	78606381	825	496	24	50.03	5.91	89795:78225068-78606788:Genebody
chr14	106035631	106036206	576	350	22	65.91	5.91	-
chr15	67349243	67350102	860	403	25	51.58	5.91	-
chr15	88137486	88138683	1198	197	35	63.48	5.91	-
chr10	42384118	42386170	2053	1107	471	197.93	5.9	-
chr5	49439018	49441499	2482	1820	137	60.02	5.9	-
chr17	9976301	9977203	903	499	25	53.57	5.88	8522:9813925-10101868:Genebody
chr17_gl000205_random	38022	38985	964	428	33	88.48	5.88	-
chr2	47105352	47106179	828	345	30	60.22	5.88	-
chr20	11349563	11350051	489	195	17	52.8	5.88	-
chr7	106239752	106240986	1235	304	37	77.27	5.88	-
chr9	96331175	96332040	866	416	24	51.98	5.88	-
chr9	138069311	138070073	763	569	23	56.27	5.88	-
chr4	49274415	49275281	867	384	30	61.51	5.86	-
chr1	248838365	248839404	1040	843	32	78.3	5.85	-
chr11	50348493	50349066	574	223	19	59.62	5.85	-
chr11	83216079	83216885	807	356	22	53.05	5.85	1740:83166055-83393468:G

								enebody
chr13	79950503	79951230	728	243	23	65.82	5.85	64062:79894099-79979923:Genebody
chr14	87302246	87303052	807	315	23	58.13	5.85	-
chr16	47186267	47186991	725	171	21	55.25	5.85	-
chr16	73376600	73377371	772	366	21	51.05	5.85	-
chr2	69282521	69283048	528	194	17	53.09	5.85	84168:69240275-69373494:Genebody
chr2	184128215	184128860	646	196	19	52.11	5.85	-
chr3	76648207	76648906	700	409	20	52.32	5.85	-
chr3	122941182	122942022	841	649	27	76.39	5.85	26984:122920773-122992980:Genebody
chr4	132353606	132354457	852	466	23	54.21	5.85	-
chr4	161964474	161965306	833	457	24	60.94	5.85	-
chr5	83722514	83723592	1079	696	28	55.34	5.85	-
chr5	154921397	154922528	1132	407	32	70.12	5.85	-
chr7	52005835	52006498	664	269	19	50.41	5.85	-
chr8	11131154	11131696	543	194	17	51.47	5.85	-
chr8	49269702	49270484	783	333	21	50.12	5.85	-
chr9	83115127	83115724	598	412	19	56.98	5.85	-
chr11	78989987	78990711	725	530	26	62.9	5.83	26011:78364328-79151695:Genebody
chr16	33358196	33359169	974	472	29	52.66	5.83	-
chr18	33709171	33710042	872	435	29	62.02	5.83	25800:336884

								94-33709357:<br>Upstream
chr2	58707181	58708032	852	346	26	50.25	5.83	-
chr21	15427533	15428573	1041	633	33	59.8	5.83	-
chrUn_gl000<br>225	183669	184997	1329	776	62	106.21	5.83	-
chrUn_gl000<br>228	116445	120099	3655	2044	279	62.99	5.82	-
chrY	13709436	13710410	975	634	38	73.23	5.82	-
chr1	208019879	208020405	527	334	16	52.39	5.81	-
chr10	6181696	6182301	606	196	18	55.97	5.81	-
chr10	30950869	30951757	889	507	23	57.56	5.81	-
chr10	125749967	125750688	722	497	21	61.66	5.81	-
chr11	40891219	40891886	668	378	18	50.1	5.81	-
chr11	118275069	118276038	970	711	28	71.66	5.81	10632:118272<br>103-11828056<br>1:Genebody
chr12	2655261	2655984	724	344	19	50.56	5.81	775:2162415-<br>2807115:Gen<br>ebody
chr12	75415996	75416645	650	477	19	57.34	5.81	-
chr15	57788219	57788755	537	248	17	57.33	5.81	84952:576687<br>04-57842920:<br>Genebody
chr16	55150378	55151047	670	198	19	55.4	5.81	-
chr16	84902326	84902975	650	329	18	51.72	5.81	83716:848535<br>86-84943116:<br>Genebody

chr2	98506602	98507259	658	196	18	50.99	5.81	23505:98372802-98612354:Genebody
chr2	134430740	134431356	617	376	18	54.87	5.81	-
chr2	187039632	187040332	701	385	19	52.56	5.81	-
chr2	202771918	202772592	675	263	19	54.93	5.81	-
chr20	39725080	39725707	628	360	18	53.8	5.81	7150:39657461-39753124:Genebody
chr3	36690495	36691043	549	387	16	50.13	5.81	-
chr3	162145375	162146019	645	194	19	57.83	5.81	-
chr3	169832919	169833783	865	395	26	76.05	5.81	80012:169805367-169899537:Genebody
chr5	111662310	111663037	728	223	20	55.55	5.81	64097:111498314-111755010:Genebody
chr7	123443269	123444137	869	448	23	59.23	5.81	-
chr9	136376588	136377890	1303	713	54	79.47	5.81	-
chrX	61682645	61683618	974	613	43	76.41	5.81	-
chr10	82415874	82416889	1016	502	31	63.86	5.8	-
chr11	58963583	58964518	936	456	28	57.22	5.8	23220:58939811-58976060:Genebody
chr11	70566594	70567217	624	174	22	58.8	5.8	22941:70313961-70935808:Genebody
chr12	70599451	70600007	557	359	19	50.44	5.8	-

chr13	21071616	21072635	1020	505	32	68.17	5.8	51084:20977808-21100012:Genebody
chr19	52976852	52978129	1278	835	36	59.72	5.8	147660:52956828-53020130:Genebody
chr2	9329524	9330446	923	436	28	58.38	5.8	-
chr20	60266273	60267295	1023	762	31	58.7	5.8	1002:59827558-60512299:Genebody
chr22	27488827	27489720	894	462	30	70.86	5.8	-
chr6	4464651	4465252	602	406	20	50.76	5.8	-
chr9	27158959	27159599	641	244	21	51.76	5.8	7010:27109146-27230171:Genebody
chr14	69140095	69140956	862	347	34	56.79	5.79	-
chr4	68264050	68264889	840	468	32	51.1	5.79	-
chr17	22251262	22253637	2376	1961	229	96.96	5.78	-
chr1	145325536	145326282	747	264	38	59.78	5.77	100132406:145293370-145368682:Genebody
chr1	185588856	185589473	618	263	20	53.84	5.77	-
chr12	52934049	52934582	534	222	15	50.96	5.77	-
chr12	119565804	119566363	560	382	16	54.45	5.77	84530:119419395-119600857:Genebody
chr13	42891730	42892354	625	433	17	54.14	5.77	11215:428462

								88-42897402:<br>Genebody
chr13	96360015	96360659	645	183	23	67.34	5.77	5611:9632940<br>1-96447241:G<br>enebody
chr14	54564560	54565147	588	339	16	51.73	5.77	-
chr14	62960967	62961557	591	336	16	51.45	5.77	-
chr15	97443151	97443887	737	292	20	61.25	5.77	-
chr16	4778869	4779335	467	278	14	51.67	5.77	124401:47465<br>12-4784163:G<br>enebody
chr17	57290353	57291033	681	284	21	52.66	5.77	55181:572873<br>70-57292610:<br>Genebody
chr19	42644851	42645395	545	355	19	56.45	5.77	-
chr19	45310614	45311245	632	161	18	59.42	5.77	4059:4531233<br>7-45323610:U<br>pstream
chr2	71466934	71467815	882	465	27	63.56	5.77	-
chr2	71995273	71995860	588	179	17	57.74	5.77	-
chr2	88768687	88769774	1088	614	37	69.47	5.77	-
chr2	145525129	145525731	603	271	16	50.34	5.77	-
chr20	17543414	17543953	540	344	16	56.5	5.77	-
chr20	53731567	53732166	600	403	17	56.54	5.77	-
chr21	41889352	41889957	606	186	16	50.07	5.77	1826:4138434<br>2-42219039:G<br>enebody
chr3	130913846	130914443	598	196	16	50.8	5.77	79858:130745

								693-131069308:Genebody
chr3	196978867	196979488	622	423	17	54.42	5.77	1739:196769431-197025447:Genebody
chr4	123281182	123281897	716	526	19	57.4	5.77	84162:123091757-123283913:Genebody
chr4	151243174	151243774	601	175	16	50.52	5.77	987:151185597-151936879:Genebody
chr4	176885558	176886218	661	391	17	50.89	5.77	2823:176554088-176923648:Genebody
chr5	111534657	111535199	543	192	15	50.08	5.77	64097:111498314-111755010:Genebody
chr6	27270824	27271442	619	382	17	54.7	5.77	-
chr6	146317955	146318431	477	156	14	50.6	5.77	-
chr7	26208182	26208868	687	324	21	52.08	5.77	9603:26191846-26226754:Genebody
chr7	48813659	48814359	701	379	18	53.05	5.77	-
chr7	73080247	73080840	594	396	16	51.17	5.77	155382:73082173-73086439:Upstream
chr7	89355748	89356430	683	379	19	60.45	5.77	-
chr8	50030288	50030929	642	167	17	52.57	5.77	-

chr8	57250628	57251254	627	391	20	52.9	5.77	-
chr9	23031353	23031886	534	287	15	50.96	5.77	-
chr9	106786786	106787384	599	319	16	50.7	5.77	-
chr7	54657020	54657967	948	759	31	57.89	5.74	-
chr9	140047025	140048407	1383	451	53	88.29	5.74	2902:140033608-140063214:Genebody
chr1	104491908	104492733	826	406	22	50.03	5.73	-
chr1	156640737	156641219	483	136	16	51.12	5.73	10763:156638556-156647189:Genebody
chr10	72758923	72759797	875	510	23	50.84	5.73	-
chr10	89852413	89853049	637	439	19	51.67	5.73	-
chr12	22057473	22058063	591	237	18	50.84	5.73	10060:21950325-22089628:Genebody
chr13	83165972	83167078	1107	301	33	75.22	5.73	-
chr14	70034152	70034928	777	388	22	54.22	5.73	56936:70036531-70037918:Downstream
chr14	83203551	83204353	803	393	22	51.95	5.73	-
chr16	2056631	2057209	579	366	18	52.06	5.73	90850:2047767-2059763:Genebody
chr19	58920796	58921509	714	323	23	60.19	5.73	201514:58920062-58929690:Genebody
chr2	9840118	9840940	823	296	23	55.18	5.73	-

chr2	53280039	53280597	559	200	18	54.19	5.73	-
chr2	113621476	113621943	468	288	16	52.87	5.73	-
chr2	122108384	122109021	638	197	19	51.58	5.73	23332:122095353-122407052:Genebody
chr22	45953777	45954393	617	239	20	59.23	5.73	2192:45898718-45954591:Genebody
chr5	92928310	92929237	928	426	24	51.36	5.73	7025:92919042-92929778:Genebody
chr6	23895277	23895966	690	235	20	51.89	5.73	-
chr6	76426958	76427554	597	316	18	50.24	5.73	26054:76311621-76427993:Genebody
chr6	161350678	161351348	671	413	20	53.7	5.73	-
chr9	119877406	119877928	523	288	17	52.42	5.73	23245:119187503-120177317:Genebody
chrUn_gl000224	73194	73803	610	430	20	60	5.73	-
chr1	2688045	2689040	996	423	28	50.76	5.7	-
chr1	85815733	85816501	769	365	25	57.32	5.7	23576:85784168-85930889:Genebody
chr2	17540151	17540952	802	559	28	63.83	5.7	-
chr2	235908449	235909345	897	523	26	50.23	5.7	23677:235860627-23596435

								6:Genebody
chr7	94544790	94545451	662	356	24	63.78	5.7	55607:94536948-94925725:Genebody
chrUn_gl000212	16186	16943	758	258	25	58.43	5.7	-
chr1	22948682	22949306	625	305	18	52.7	5.68	-
chr1	232468596	232469398	803	477	24	68.94	5.68	-
chr10	95094060	95094924	865	410	22	52.82	5.68	26509:95066186-95242074:Genebody
chr10	114257499	114258030	532	182	16	50.59	5.68	143187:114206755-114578501:Genebody
chr10	127831374	127832194	821	444	35	88.71	5.68	8038:127702901-128077127:Genebody
chr11	41409393	41409947	555	378	17	54.03	5.68	-
chr12	6730438	6731065	628	450	20	63.94	5.68	57121:6728000-6740815:Genebody
chr12	105572910	105573457	548	372	18	60.8	5.68	55198:105567074-105630008:Genebody
chr13	89578416	89579023	608	402	18	54.37	5.68	-
chr19	37150139	37150708	570	375	17	52.48	5.68	92283:37128283-37157739:Genebody

chr2	39561893	39562588	696	442	19	51.58	5.68	8491:39476422-39664219:Genebody
chr2	81215489	81216351	863	407	22	52.98	5.68	-
chr20	61075441	61076153	713	313	19	50.09	5.68	-
chr22	49300477	49301172	696	459	21	62.62	5.68	-
chr3	74439911	74440561	651	278	18	50.26	5.68	5067:74311721-74570343:Genebody
chr3	135605142	135605901	760	510	22	61.96	5.68	-
chr4	80300668	80301337	670	510	20	59.54	5.68	-
chr4	85879635	85880169	535	343	16	50.28	5.68	23001:85590696-85887544:Genebody
chr4	154133136	154133739	604	386	19	60.59	5.68	23321:154074269-154260474:Genebody
chr6	7430033	7430696	664	191	19	54.52	5.68	-
chr6	170697980	170698573	594	197	18	55.79	5.68	84498:170615843-170714236:Genebody
chr9	114814858	114815394	537	191	16	50.08	5.68	64420:114803061-114937556:Genebody
chr4_gl000193_random	82953	83811	859	624	52	65.33	5.67	-
chr1	197087441	197088168	728	252	22	51.88	5.66	259266:197053257-1971158

								24:Genebody
chr10	107096186	107097127	942	527	26	52.04	5.66	-
chr11	10540915	10541752	838	418	26	61.21	5.66	50862:10533224-10562774:Genebody
chr13	60314092	60314898	807	386	24	54.31	5.66	81624:60239724-60738119:Genebody
chr21	40506050	40506938	889	459	25	51.86	5.66	-
chr7	153506493	153507240	748	424	22	50.04	5.66	-
chr8	16997948	16998626	679	459	21	51.59	5.66	-
chr8	79792130	79792923	794	310	24	55.51	5.66	-
chr1	94171183	94171848	666	413	27	67.18	5.65	-
chr12	133779507	133780547	1041	677	31	52.68	5.65	10795:133757994-133783695:Genebody
chr17	63201425	63202216	792	397	26	53.31	5.65	8787:63133455-63206778:Genebody
chr9_gl000199_random	159724	161173	1450	1019	84	97.67	5.64	-
chr15	80209676	80210254	579	394	17	57.15	5.63	400410:80191182-80216077:Genebody
chr21	32844425	32845003	579	180	16	51.18	5.63	7074:32490735-32931290:Genebody
chr1	46820653	46821263	611	194	17	53.98	5.62	387338:46806

								389-46830690 :Genebody
chr1	51819697	51820268	572	414	16	51.85	5.62	2060:5181993 4-51887793:G enebody
chr1	228344391	228344925	535	308	16	55.59	5.62	57165:228337 552-22834752 5:Genebody
chr10	31258881	31259607	727	241	19	54.81	5.62	220929:31133 566-31288446 :Genebody
chr10	85321037	85321656	620	444	18	59.03	5.62	-
chr11	69520678	69521307	630	194	17	52.2	5.62	9965:6951300 6-69519106:U pstream
chr12	121146650	121147218	569	395	18	64.46	5.62	84747:121147 830-12116144 2:Upstream
chr13	57317697	57318291	595	400	17	55.54	5.62	-
chr13	95654282	95654870	589	178	16	50.24	5.62	-
chr14	21231003	21231578	576	317	16	51.47	5.62	-
chr14	24764856	24765454	599	154	17	55.14	5.62	115817:24759 805-24768666 :Genebody
chr14	44107713	44108343	631	200	17	52.11	5.62	-
chr16	79439781	79440552	772	387	22	67.81	5.62	-
chr19	33539926	33540675	750	403	19	52.86	5.62	85415:334694 98-33555824:

								Genebody
chr2	192889159	192889758	600	185	17	55.04	5.62	23671:192814748-193059644:Genebody
chr2	239492746	239493379	634	168	17	51.84	5.62	-
chr21	18618223	18618890	668	493	19	60.24	5.62	-
chr21	35139178	35139647	470	288	14	50.08	5.62	6453:35014783-35210801:Genebody
chr22	38604368	38604949	582	189	16	50.9	5.62	23764:38597938-38612515:Genebody
chr3	78583358	78584000	643	486	17	51.03	5.62	-
chr3	116074538	116075126	589	421	16	50.24	5.62	4045:115521210-116164385:Genebody
chr3	131529422	131529984	563	376	16	52.73	5.62	131034:131253583-131753844:Genebody
chr3	144176870	144177499	630	192	17	52.2	5.62	-
chr3	152707336	152707901	566	217	16	52.44	5.62	-
chr4	143007654	143008283	630	450	17	52.2	5.62	8821:142949183-143767604:Genebody
chr5	3076351	3076869	519	347	16	57.32	5.62	-
chr5	106317699	106318386	688	398	18	52.66	5.62	-
chr5	154059908	154060435	528	183	15	50.22	5.62	-
chr7	139446102	139446807	706	523	19	56.68	5.62	28996:139246

								316-13947769 3:Genebody
chr8	81044507	81045097	591	188	16	50.05	5.62	7163:8094710 4-81083836:G enebody
chr10	92740277	92741182	906	511	27	66.3	5.61	-
chr10	95429900	95430431	532	338	17	50.26	5.61	118924:95427 640-95462329 :Genebody
chr10	103598379	103599183	805	481	23	55.29	5.61	30819:103585 731-10359961 1:Genebody
chr11	18013630	18014375	746	338	21	50.56	5.61	26297:178095 98-18034637: Genebody
chr12	1646071	1646699	629	363	20	56.58	5.61	-
chr12	123601897	123602785	889	406	25	57.8	5.61	-
chr13	21957497	21958182	686	352	20	50.94	5.61	253832:21950 509-22033423 :Genebody
chr14	32489435	32490280	846	495	24	56.63	5.61	-
chr15	28390422	28391651	1230	345	34	67.66	5.61	8924:2835618 9-28567295:G enebody
chr17	65883628	65884192	565	234	20	63.8	5.61	2186:6582177 9-65980493:G enebody
chr2	137688293	137689302	1010	373	28	57.3	5.61	-

chr21	45146235	45146909	675	282	21	57.27	5.61	8566:45138977-45182187:Genebody
chr4	97604169	97604985	817	335	24	59.25	5.61	-
chr5	53592116	53592744	629	165	19	51.18	5.61	-
chr5	113490522	113491330	809	503	23	54.93	5.61	-
chr6	15751672	15752347	676	508	21	57.17	5.61	-
chr8	93645718	93646638	921	414	25	55.1	5.61	-
chr8	95066756	95067658	903	405	27	66.58	5.61	-
chr11	434835	435695	861	360	29	66.11	5.6	338440:417929-442011:Genebody
chr16	86960924	86961723	800	384	25	53.01	5.6	-
chr2	168839029	168839895	867	505	27	56.01	5.6	27347:168810530-169104105:Genebody
chr22	33681955	33682752	798	230	25	53.19	5.6	9215:33669062-34316416:Genebody
chr5	149508570	149509587	1018	676	30	51.82	5.6	5159:149493402-149535422:Genebody
chr1	78299176	78299755	580	159	15	51.05	5.58	374986:78245308-78344077:Genebody
chr10	46166599	46167244	646	447	16	51.2	5.58	93550:46111039-46168251:Genebody

chr10	50199397	50199949	553	361	15	53.59	5.58	-
chr10	64537997	64538577	581	417	16	57.15	5.58	-
chr10	105745838	105746405	568	375	15	52.16	5.58	9748:105727469-105787342:Genebody
chr11	28075943	28076615	673	239	17	54.75	5.58	81930:28042162-28129746:Genebody
chr12	79603792	79604444	653	195	16	50.6	5.58	6857:79257772-79845787:Genebody
chr13	112943410	112944000	591	398	15	50.07	5.58	-
chr14	27955672	27956241	570	371	15	51.97	5.58	-
chr14	76652632	76653252	621	440	16	53.39	5.58	55668:76618258-76669133:Genebody
chr15	58726591	58727216	626	434	16	52.94	5.58	3990:58724174-58861072:Genebody
chr15	79475715	79476305	591	164	15	50.07	5.58	-
chr16	73561408	73562031	624	357	16	53.12	5.58	-
chr17	64244173	64244650	478	195	14	54.79	5.58	-
chr18	28146908	28147559	652	232	17	56.62	5.58	-
chr18	51029306	51029812	507	357	14	51.79	5.58	1630:49866541-51062273:Genebody
chr19	48636398	48637048	651	268	16	50.77	5.58	3978:48618702-48673560:G

								enebody
chr2	22404809	22405415	607	412	16	54.67	5.58	-
chr2	51961623	51962144	522	177	14	50.32	5.58	-
chr2	113186135	113186783	649	463	16	50.94	5.58	727851:113125946-113191222:Genebody
chr2	144868640	144869222	583	183	15	50.78	5.58	79712:144703582-144994906:Genebody
chr2	160722363	160722935	573	386	15	51.7	5.58	4065:160659868-160761262:Genebody
chr2	165833228	165833874	647	219	16	51.11	5.58	-
chr2	207508716	207509308	593	399	16	55.98	5.58	200726:207507141-207514173:Genebody
chr22	43881066	43881581	516	159	14	50.9	5.58	758:43808019-43902799:Genebody
chr3	80354275	80354793	519	322	14	50.61	5.58	-
chr3	89009169	89009743	575	383	15	51.51	5.58	-
chr3	135455976	135456533	558	252	15	53.11	5.58	-
chr3	190126705	190127316	612	181	16	54.21	5.58	10686:190105660-190129931:Genebody
chr4	11830364	11830936	573	192	15	51.7	5.58	-
chr4	79651861	79652497	637	407	16	51.97	5.58	-
chr4	113197058	113197643	586	412	15	50.51	5.58	92610:113196

								782-113207059:Genebody
chr4	148184055	148184583	529	181	15	55.98	5.58	-
chr4	158966390	158966954	565	387	15	52.44	5.58	-
chr5	74443870	74444493	624	225	16	53.12	5.58	256006:74364122-74532703:Genebody
chr5	108366606	108367173	568	179	16	58.44	5.58	2241:108083522-108523373:Genebody
chr6	9071534	9072117	584	147	15	50.69	5.58	-
chr6	27315870	27316515	646	384	17	57.17	5.58	-
chr6	84325124	84325746	623	156	16	53.21	5.58	9892:84262606-84419127:Genebody
chr6	103575358	103575966	609	425	16	54.48	5.58	-
chr7	21722943	21723520	578	176	15	51.24	5.58	8701:21582832-21941455:Genebody
chr7	98173502	98174018	517	200	14	50.8	5.58	-
chr7	102254042	102254618	577	380	16	57.54	5.58	10156:102220093-102257205:Genebody
chr7	105052325	105052933	609	415	16	54.48	5.58	-
chr8	106708204	106708792	589	268	15	50.24	5.58	23414:106331146-106816765:Genebody
chr9	23003599	23004178	580	188	15	51.05	5.58	-

chr9	27589386	27590082	697	273	19	64.67	5.58	-
chr9	101909287	101909863	577	260	15	51.33	5.58	7046:101867411-101916471:Genebody
chr1	1150826	1151423	598	195	16	55.26	5.56	7293:1146706-1149512:Upstream
chr1	6655027	6655602	576	351	19	50.62	5.56	9903:6650784-6662929:Genebody
chr1	21364550	21365123	574	411	15	51.37	5.56	8672:21132975-21503340:Genebody
chr1	45312248	45312922	675	513	20	57.54	5.56	-
chr1	107238659	107239479	821	438	22	54.87	5.56	-
chr1	116367362	116367977	616	163	16	53.59	5.56	-
chr1	209853798	209854471	674	372	21	50.85	5.56	-
chr1	229284702	229285799	1098	547	34	74.02	5.56	-
chr10	53005199	53005791	593	216	16	55.73	5.56	5592:52750944-54055274:Genebody
chr10	134248373	134249481	1109	516	58	137.79	5.56	-
chr11	30769759	30770469	711	364	20	54.13	5.56	-
chr11	121609290	121609954	665	476	19	53.01	5.56	-
chr12	83327843	83328617	775	579	23	64.37	5.56	160335:83080933-83528063:Genebody
chr12	105955216	105955849	634	391	16	51.99	5.56	-

chr13	82901149	82901880	732	534	22	50.24	5.56	-
chr15	36843530	36844194	665	462	18	61.25	5.56	-
chr18	7584946	7585638	693	188	19	50.45	5.56	5797:7567313-8406858:Genebody
chr18	20655180	20656018	839	524	24	50.14	5.56	-
chr18	69632386	69632964	579	185	19	61.89	5.56	-
chr2	18743115	18744166	1052	688	29	65.85	5.56	57665:18735990-18741959:Upstream
chr2	194321080	194321889	810	397	21	50.75	5.56	-
chr20	37992910	37993673	764	516	21	54.64	5.56	-
chr3	38199722	38200326	605	291	20	52.77	5.56	-
chr3	52417370	52418120	751	289	20	50.6	5.56	25981:52350334-52434512:Genebody
chr3	68754990	68755582	593	398	16	55.73	5.56	-
chr4	37639332	37639936	605	185	18	53.31	5.56	768211:37592421-37687999:Genebody
chr4	53743343	53744083	741	378	20	51.46	5.56	152579:53739151-54232242:Genebody
chr4_gl000194_random	2270	2993	724	304	20	52.95	5.56	-
chr5	4664914	4665436	523	176	14	50	5.56	-
chr5	57096579	57097364	786	191	28	75.54	5.56	-
chr5	88373395	88374062	668	372	19	52.73	5.56	-

chr6	45780275	45780955	681	292	19	51.53	5.56	-
chr6	70757415	70758127	713	426	17	51.14	5.56	1310:70576447-70922157:Genebody
chr7	22098016	22098557	542	374	15	54.43	5.56	-
chr7	151125987	151126910	924	281	33	71.08	5.56	155051:151127056-151137099:Downstream
chr8	26362592	26363160	569	199	15	51.83	5.56	10687:26362195-26371483:Genebody
chr8	81557814	81558729	916	403	29	62.08	5.56	619279:81550768-81787016:Genebody
chr9	101974392	101974934	543	197	15	54.33	5.56	-
chr10	38817061	38818220	1160	192	51	70.23	5.53	-
chr10	81085550	81086509	960	418	33	73.81	5.51	-
chr11	13363796	13364816	1021	506	33	67.71	5.51	406:13299324-13408810:Genebody
chr13	22544990	22545946	957	687	31	55.52	5.51	-
chr14	65467570	65468457	888	352	27	52.76	5.51	2342:65453506-65529369:Genebody
chr4	3912377	3913475	1099	502	36	74.16	5.51	-
chr10	32834988	32835827	840	338	24	55.66	5.5	221016:32735040-32863490

								:Genebody
chr12	103513417	103514137	721	384	21	51.48	5.5	-
chr13	70572804	70573692	889	603	29	71.39	5.5	57626:70274725-70682625:Genebody
chr17	41842562	41843322	761	429	22	52.83	5.5	1845:41843489-41856368:Downstream
chr2	107029823	107030575	753	250	24	63.95	5.5	653489:107021135-107084801:Genebody
chr2	218620166	218620667	502	326	17	52.43	5.5	729582:218148747-218621316:Genebody
chr3	187094709	187095455	747	531	22	54.12	5.5	-
chr4	171342064	171342609	546	354	18	53.14	5.5	-
chr4	185420641	185421351	711	534	22	57.58	5.5	-
chr6	3806244	3806924	681	200	21	55.3	5.5	-
chr8	122213187	122214479	1293	197	37	74.47	5.5	-
chr11	42365178	42365762	585	269	17	55.08	5.49	-
chr12	94869190	94869884	695	356	18	50.57	5.49	-
chr12	98275166	98275803	638	453	18	55.74	5.49	-
chr13	81098154	81098860	707	229	19	55.02	5.49	-
chr14	19818982	19819959	978	527	37	56.62	5.49	-
chr15	32028230	32028792	563	200	16	51.36	5.49	-
chr21	26903492	26904158	667	198	18	53.04	5.49	-
chr4	39627532	39628093	562	365	16	51.46	5.49	201895:39552549-39640481

								:Genebody
chr5	149616963	149617657	695	406	18	50.57	5.49	815:14959905 3-149669403: Genebody
chr7	57737560	57738360	801	509	22	63.27	5.49	-
chr7	133217998	133218624	627	179	17	51.05	5.49	60412:132937 822-13375051 1:Genebody
chr8	98508731	98509244	514	342	15	50.33	5.49	-
chr9	26729631	26730326	696	174	18	50.49	5.49	-
chr8	102635298	102636903	1606	1286	55	96.23	5.47	79977:102504 667-10268195 0:Genebody
chr10	35838071	35838975	905	531	26	52.27	5.45	219771:35535 952-35860845 :Genebody
chr12	126347305	126347869	565	200	19	50.67	5.45	-
chr16	11557154	11558043	890	647	30	67.9	5.45	-
chr3	104872694	104873543	850	408	26	57.13	5.45	-
chr3	148519874	148520596	723	248	25	64.97	5.45	-
chr6	44678595	44679568	974	550	31	69.47	5.45	-
chr6	72106156	72106861	706	509	24	61.57	5.45	-
chr9	5175075	5175819	745	395	23	52.58	5.45	11172:516386 4-5185618:Ge nebody
chr1	143754139	143754971	833	469	22	52.35	5.43	-
chr10	3850631	3851370	740	337	21	55.32	5.43	-
chr12	44445129	44445864	736	483	20	50.49	5.43	84216:442299

								61-44783540:Genebody
chr12	95069496	95070181	686	489	20	55.03	5.43	-
chr14	75824294	75824889	596	261	18	52.88	5.43	-
chr14	92878074	92878562	489	163	16	52.8	5.43	123041:92788924-92962594:Genebody
chr15	77328080	77328661	582	232	18	54.32	5.43	9051:77287464-77329671:Genebody
chr16	6014369	6015065	697	502	21	59.42	5.43	-
chr16	21545523	21546247	725	284	20	51.45	5.43	-
chr17	29228373	29229024	652	165	19	52.87	5.43	79736:29226000-29233286:Genebody
chr19	11591939	11592741	803	364	24	65.44	5.43	1995:11562142-11591803:Upstream
chr2	196589627	196590449	823	542	22	53.18	5.43	57181:196521531-196602425:Genebody
chr21	24557017	24557585	569	181	17	50.03	5.43	-
chr4	170928030	170928644	615	383	18	51	5.43	9848:170907749-170947429:Genebody
chr5	40225632	40226230	599	399	18	52.58	5.43	-
chr5	149972436	149973046	611	349	18	51.39	5.43	-
chr6	10660573	10661380	808	552	24	64.95	5.43	-

chr6	26395285	26396289	1005	568	33	54.49	5.43	10385:26383353-26395098:Downstream
chr6	68748387	68749075	689	492	20	54.74	5.43	-
chr8	37356201	37357600	1400	562	55	60.91	5.43	-
chr8	51677218	51677831	614	423	18	51.1	5.43	54212:50824596-51705427:Genebody
chr8	113085980	113087036	1057	261	30	68.5	5.43	-
chr9	130908450	130909245	796	376	23	60.72	5.43	-
chr11	2492614	2493707	1094	662	43	66.9	5.42	3784:2466220-2870339:Genebody
chr19	46871735	46872637	903	436	27	50.15	5.42	5536:46850293-46894103:Genebody
chr4	1384245	1385235	991	479	31	59.86	5.42	285464:1385339-1389782:Upstream
chr5	132802366	132803325	960	459	34	77.01	5.42	23105:132532151-132948223:Genebody
chr6	170037190	170038129	940	526	30	55.61	5.42	253769:169857306-170102159:Genebody
chrX	61710569	61711504	936	739	97	150.35	5.42	-
chr1	69799939	69800526	588	174	16	54.66	5.41	-
chr10	71265369	71265911	543	368	15	52.86	5.41	23555:712112

								25-71267423:<br>Genebody
chr11	27591529	27592127	599	309	16	53.62	5.41	497258:27528<br>398-27699348<br>:Genebody
chr13	48739631	48740230	600	353	16	53.53	5.41	-
chr17	74180966	74181563	598	405	16	53.72	5.41	114804:74138<br>534-74236390<br>:Genebody
chr2	65455575	65456187	613	192	16	52.34	5.41	10097:654548<br>28-65498385:<br>Genebody
chr4	70904690	70905260	571	293	15	50.2	5.41	-
chr5	136983483	136984097	615	213	16	52.16	5.41	26249:136953<br>189-13707177<br>9:Genebody
chr16	2886720	2887532	813	465	24	56.61	5.39	-
chr3	12567335	12568176	842	249	27	69.14	5.39	80746:125259<br>30-12574819:<br>Genebody
chr5	125231953	125232540	588	199	20	58.4	5.39	-
chr7	21449705	21450421	717	198	22	55.59	5.39	-
chr18	68719979	68720912	934	330	32	62.42	5.38	-
chr21	30519571	30521057	1487	478	55	80.54	5.38	56911:304528<br>72-30548200:<br>Genebody
chr1	145257810	145258558	749	404	30	66.61	5.36	388677:14520<br>9110-1452859

								11:Genebody
chr10	45739724	45740279	556	383	16	50.73	5.36	-
chr11	92580173	92581014	842	487	25	51.81	5.36	120114:92085261-92629633:Genebody
chr14	47706968	47707559	592	186	17	52.98	5.36	161357:47308829-47812438:Genebody
chr14	97647634	97648854	1221	539	41	92.13	5.36	-
chr15	22040580	22041500	921	598	32	56.93	5.36	-
chr19	29184110	29184842	733	500	25	62.44	5.36	-
chr2	110328777	110329396	620	401	17	50.32	5.36	151011:110300377-110371783:Genebody
chr2	116167123	116167613	491	186	15	51.48	5.36	57628:115199898-116602324:Genebody
chr2	159295532	159296179	648	368	18	53.33	5.36	130940:159027868-159313265:Genebody
chr3	12183137	12184050	914	493	26	50.15	5.36	6854:12045861-12227226:Genebody
chr3	133738113	133738664	552	363	16	51.12	5.36	6578:133651541-133748920:Genebody
chr4	57483195	57483798	604	196	17	51.82	5.36	-
chr4	128336552	128337227	676	220	19	56.33	5.36	-

chr6	50956914	50957489	576	429	17	54.58	5.36	-
chr6	162255083	162255715	633	205	18	54.75	5.36	5071:161768590-163148834:Genebody
chr8	69477371	69477970	600	412	17	52.2	5.36	116328:69350147-69731256:Genebody
chr8	135656366	135657308	943	519	27	52.14	5.36	57623:135490032-135708801:Genebody
chr9	19971043	19972067	1025	220	30	54.15	5.36	-
chr14	20423850	20424763	914	337	28	52.2	5.33	-
chr14	42242384	42243569	1186	380	34	50.73	5.33	145581:42076763-42373750:Genebody
chr17	77408000	77409199	1200	441	36	61.65	5.33	146713:77085427-77478563:Genebody
chr2	16830390	16831310	921	509	29	55.93	5.33	81553:16730731-16847134:Genebody
chr9	70835727	70837206	1480	370	41	56.06	5.33	-
chr1	217526116	217526887	772	421	21	51.05	5.32	-
chr11	12903540	12904064	525	366	17	53.42	5.32	7003:12695968-12966283:Genebody
chr13	44621964	44622616	653	424	20	56.86	5.32	-
chr2	84856029	84856752	724	309	20	50.17	5.32	1768:8474357

								8-85046711:Genebody
chr2	85355583	85356243	661	286	20	56.05	5.32	-
chr2	174376186	174376678	493	185	16	51.15	5.32	-
chr3	31749686	31750351	666	164	19	50.22	5.32	114884:31702317-32023342:Genebody
chr5	42619142	42619805	664	200	19	50.41	5.32	2690:42424025-42721925:Genebody
chr5	128168321	128168919	599	177	19	56.87	5.32	-
chr7	39741947	39742551	605	431	18	50.7	5.32	5898:39663161-39747715:Genebody
chr7	50856372	50856909	538	163	17	52.01	5.32	2887:50657759-50861159:Genebody
chr20	60662733	60663574	842	462	28	52.46	5.3	-
chr11	120733486	120734223	738	407	22	52.25	5.29	2900:120531027-120856969:Genebody
chr13	33913127	33913803	677	460	21	53.05	5.29	-
chr13	106357418	106358295	878	484	25	54.22	5.29	-
chr17	19346423	19347320	898	534	26	57.22	5.29	-
chr11	34822136	34823211	1076	580	35	75.32	5.26	-
chr12	115921345	115922577	1233	409	36	61.48	5.26	-
chr14	31911653	31912264	612	345	16	50.95	5.26	-
chr19	22654276	22655467	1192	759	34	56.35	5.26	-

chr22	17747632	17748232	601	413	16	51.95	5.26	-
chr9	89418064	89418911	848	577	32	56.96	5.26	-
chr9	106782310	106784204	1895	1150	109	156.03	5.25	-
chr14	21819742	21820747	1006	536	30	51.46	5.24	57096:21756135-21819458:Downstream
chr19	14317659	14319098	1440	840	90	141.23	5.24	22859:14258550-14316997:Upstream
chr8	39970126	39971096	971	470	29	50.25	5.24	-
chr1	242496921	242497518	598	441	17	51.04	5.23	200150:242252271-242687998:Genebody
chr10	89583936	89584629	694	505	19	53.19	5.23	142913:89578069-89605365:Genebody
chr13	19076030	19076578	549	237	17	56.02	5.23	-
chr13	70062519	70063222	704	273	19	52.29	5.23	-
chr17	49055002	49055606	605	407	17	50.37	5.23	9043:49039535-49198226:Genebody
chr22	46665146	46665691	546	356	16	50.43	5.23	55020:46663860-46689903:Genebody
chr3	108499752	108500411	660	303	18	50.81	5.23	-
chr6	165971378	165971966	589	397	17	51.91	5.23	10846:165740778-166075584:Genebody

chr8	138367811	138368540	730	499	20	55.37	5.23	-
chr8	75497804	75498713	910	581	30	53.36	5.22	-
chr12	14410776	14411658	883	515	23	50.21	5.21	-
chr14	24836093	24836769	677	468	20	53.12	5.21	4776:24836144-24848808:Genebody
chr14	107104342	107105051	710	414	20	50.07	5.21	-
chr16	65910224	65910931	708	335	22	60.79	5.21	-
chr18	43633645	43634409	765	520	23	60.51	5.21	9050:43563502-43652250:Genebody
chr19	45391177	45391987	811	452	24	61.38	5.21	5819:45349392-45392485:Genebody
chr2	67740309	67740998	690	302	22	62.66	5.21	-
chr2	134676377	134676958	582	213	18	51.75	5.21	-
chr2	176772115	176772972	858	384	23	52.22	5.21	-
chr3	62441260	62441978	719	236	21	54.39	5.21	8618:62384020-62861064:Genebody
chr5	118992852	118993905	1054	411	28	55.53	5.21	-
chr6	5355950	5356600	651	182	20	55.67	5.21	10667:5261583-5771816:Genebody
chr7	4288162	4289024	863	424	32	60.28	5.21	221935:3341079-4308629:Genebody
chr7	100431647	100432353	707	196	20	50.34	5.21	-

chr1	40993190	40994071	882	371	25	52.45	5.19	-
chr11	5046215	5046843	629	388	20	51.47	5.19	-
chr15	27137828	27138580	753	382	24	59.48	5.19	2558:27111865-27194356:Genebody
chr2	222004779	222005976	1198	194	30	62.65	5.19	-
chr5	110869034	110869883	850	482	24	50.55	5.19	-
chr8	63056838	63057501	664	178	22	58.29	5.19	-
chr9	34635410	34636078	669	419	21	52.58	5.19	10280:34634719-34637768:Genebody
chr11	123453488	123454030	543	347	20	55.16	5.17	57476:123396527-123493518:Genebody
chr13	22464287	22465366	1080	524	38	87.72	5.17	-
chr5	92642015	92642761	747	263	25	58.22	5.17	-
chr5	136521757	136522755	999	396	33	71.54	5.17	6695:136310987-136835018:Genebody
chr5	172200862	172202202	1341	482	37	55.71	5.17	-
chr21	10200797	10201665	869	497	30	51.76	5.15	-
chr21	10903778	10904707	930	256	33	56.42	5.14	-
chr17	2416673	2417248	576	393	16	52.87	5.13	79066:2319349-2415200:Upstream
chr17	1884856	1885634	779	482	21	54.85	5.11	146760:1837972-1928178:Genebody

chr2	7203484	7204119	636	465	18	51.65	5.11	-
chr2	76362759	76363614	856	478	25	69.36	5.11	-
chr5	166636585	166637115	531	332	16	50.7	5.11	-
chr9	98459131	98459892	762	463	20	51.11	5.11	-
chr1	228259506	228260402	897	440	26	62.05	5.1	-
chr1	247912722	247913388	667	498	21	58.09	5.1	-
chr14	22600485	22601170	686	244	20	50.94	5.1	-
chr15	101621759	101622733	975	538	26	55.43	5.1	-
chr16	69149204	69149949	746	492	22	55.62	5.1	3038:69140159-69152617:Genebody
chr3	184152447	184153081	635	444	19	50.59	5.1	-
chr4	147253678	147254388	711	417	21	53.75	5.1	84068:147175136-147443123:Genebody
chr1	231478741	231479335	595	425	20	53.85	5.09	83932:231473681-231489988:Genebody
chr11	80565326	80566142	817	497	24	52.05	5.09	-
chr18	28740459	28741607	1149	425	32	56.09	5.09	1823:28709215-28742819:Genebody
chr2	192287681	192288386	706	487	23	57.76	5.09	4430:192110106-192290115:Genebody
chr9	83870583	83871248	666	217	22	56.76	5.09	-
chr9	112865030	112865793	764	228	23	52.1	5.09	445815:112542576-1129347

								89:Genebody
chr11	29306521	29307098	578	408	20	50.03	5.08	-
chr11	65485783	65486707	925	655	32	64.78	5.08	10524:65479488-65487074:Genebody
chr12	126811514	126812345	832	190	27	51.5	5.08	-
chr17	54820665	54821978	1314	338	42	67.57	5.08	-
chr2	75042935	75044130	1196	260	35	56.95	5.08	-
chr22	39924481	39925408	928	389	30	62.7	5.08	91582:39925098-39928860:Downstream
chr6	170707355	170709727	2373	547	160	114.28	5.08	84498:170615843-170714236:Genebody
chr8	87774284	87774862	579	198	22	60.42	5.08	-
chr17	965458	967717	2260	480	162	161.8	5.03	29:906759-1012324:Genebody
chrUn_gl000220	117117	118701	1585	787	407	119.27	5.02	-
chr1	51358865	51359486	622	232	17	52.94	5	11124:50906934-51425936:Genebody
chr10	29398925	29399919	995	704	31	62.74	5	-
chr11	18739033	18740094	1062	863	35	66.77	5	283284:18725851-18747777:Genebody
chr11	24893383	24893962	580	387	16	51.08	5	338645:24518

								555-25104182<br>:Genebody
chr11	88214443	88214957	515	322	17	50.96	5	-
chr11	98922499	98923492	994	189	28	65.66	5	53942:988918<br>70-100227472<br>:Genebody
chr11	111327083	111328015	933	351	28	51.88	5	-
chr13	94543711	94544412	702	519	21	53.25	5	10082:938790<br>77-95060267:<br>Genebody
chr14	75618320	75619022	703	514	22	58.38	5	10972:755981<br>70-75643349:<br>Genebody
chr16	73280762	73282370	1609	1024	77	93.52	5	-
chr17	51123279	51124035	757	424	23	51.45	5	-
chr17	80201828	80202831	1004	467	30	61.94	5	1453:8020224<br>1-80231573:G<br>enebody
chr2	2184019	2184982	964	700	35	73.08	5	23040:179288<br>6-2335045:Ge<br>nebody
chr2	49660273	49661167	895	328	25	55.74	5	-
chr2	101521292	101522063	772	353	23	56.85	5	4862:1014366<br>12-101613287<br>:Genebody
chr2	183718665	183719837	1173	775	33	65.66	5	2487:1836980<br>04-183731498<br>:Genebody

chr2	223341388	223342222	835	487	33	86.48	5	130367:223289321-223423616:Genebody
chr2	240512084	240512996	913	452	26	59.01	5	-
chr20	61432798	61433516	719	183	22	50.21	5	55257:61427804-61431943:Downstream
chr21	22847002	22847606	605	423	19	59.03	5	4685:22370632-22911214:Genebody
chr22	28027167	28028310	1144	463	34	52.14	5	-
chr4	6058911	6059569	659	377	20	52.22	5	152789:6027925-6202318:Genebody
chr5	171908095	171908792	698	250	19	50.01	5	-
chr6	42243173	42243882	710	279	23	56.01	5	55809:42192668-42419783:Genebody
chr6	143058841	143060126	1286	435	39	73.92	5	-
chr7	4643110	4643933	824	487	33	79.5	5	-
chr7	102182600	102183436	837	359	29	52.45	5	548644:102178365-102213068:Genebody
chr7	154728565	154729231	667	480	21	50.33	5	100132707:154720226-154738558:Genebody
chr8	122967558	122968509	952	403	29	54.55	5	-

chr9	89181312	89181994	683	183	19	51.35	5	-
chr10	42598748	42600562	1815	1017	239	80.71	4.97	-
chr21	11087344	11089258	1915	647	91	68.42	4.96	85316:11020841-11098925:Genebody
chr1	148852862	148853783	922	346	31	64.93	4.92	-
chr12	8272389	8273224	836	402	28	53.04	4.92	-
chr12	45433452	45434444	993	530	34	64	4.92	440097:45408538-45444882:Genebody
chr13	46077768	46078553	786	222	29	64.75	4.92	83548:46039070-46110759:Genebody
chr15	72549595	72550803	1209	860	38	57.12	4.92	56965:72533522-72563628:Genebody
chr16	29230536	29231690	1155	836	40	70.08	4.92	-
chr2	218856496	218857424	929	566	32	57.13	4.92	-
chr20	18002862	18003853	992	609	31	54	4.92	58495:18004795-18038521:Downstream
chr6	26760762	26761912	1151	367	35	57.42	4.92	-
chrX	9377473	9378297	825	212	29	60.62	4.92	-
chr20	57198201	57198898	698	470	22	50.99	4.91	-
chr8	42222278	42222885	608	196	20	50.13	4.91	5423:42196029-42229313:Genebody
chr1	54102790	54103841	1052	198	28	50.91	4.9	148979:53971

								905-54199877<br>:Genebody
chr1	111875804	111876415	612	181	19	50.4	4.9	-
chr1	182337090	182337752	663	466	20	50.57	4.9	-
chr18	33933907	33934738	832	364	24	54.9	4.9	80206:338777<br>01-34360018:<br>Genebody
chr20	29450535	29451367	833	515	24	54.81	4.9	-
chr5	14199943	14200659	717	173	21	50.54	4.9	7204:1414382<br>8-14509450:G<br>enebody
chr5	120664008	120664804	797	503	24	58.1	4.9	-
chr5	172213567	172214342	776	595	25	60.12	4.9	-
chr8	144234273	144235284	1012	625	29	58.5	4.9	-
chr1	227493867	227494512	646	233	19	53.45	4.89	8476:2271775<br>66-227505826<br>:Genebody
chr13	92459973	92460994	1022	288	29	66.45	4.89	2262:9205093<br>4-93519485:G<br>enebody
chr18	60857736	60858442	707	474	20	53.07	4.89	596:60790578<br>-60986613:Ge<br>nebody
chr22	43262798	43263608	811	348	23	59.35	4.89	-
chr5	41511720	41512466	747	269	21	54.68	4.89	345557:41307<br>047-41510730<br>:Upstream
chr7	120749934	120750549	616	426	18	50.91	4.89	79974:120628

								750-12093749<br>7:Genebody
chr1	22904935	22906284	1350	864	52	77.26	4.88	2046:2289000<br>3-22916103:G<br>enebody
chr21	10792691	10793790	1100	695	49	54.13	4.86	-
chr7_gl00019<br>5_random	119681	120515	835	253	31	58.88	4.86	-
chr17_gl0002<br>05_random	78481	79636	1156	437	47	56.2	4.85	-
chr5	21496088	21497336	1249	890	57	82.22	4.85	728411:21459<br>588-21497305<br>:Genebody
chr13	110861919	110862460	542	361	22	61.6	4.84	1282:1108013<br>10-110959496<br>:Genebody
chr5	136363132	136363794	663	469	23	52.35	4.84	6695:1363109<br>87-136835018<br>:Genebody
chr17	29034760	29036046	1287	379	68	94.86	4.82	-
chr2	1559005	1560064	1060	522	34	72.1	4.82	-
chr2	180357736	180358519	784	259	25	55.84	4.82	151126:18030<br>6719-1804273<br>15:Genebody
chr1	38048922	38049742	821	455	26	59.36	4.81	29889:380324<br>13-38061586:<br>Genebody
chr11	95777313	95777958	646	459	20	50.99	4.81	84441:957114

								39-96076344:<br>Genebody
chr2	160222919	160223907	989	651	29	58.8	4.81	29994:160175<br>491-16047305<br>9:Genebody
chr2	170163566	170164544	979	705	29	59.65	4.81	4036:1699836<br>19-170219122<br>:Genebody
chr3	80607732	80608423	692	454	21	51.6	4.81	-
chr11	90415581	90416072	492	185	16	51.27	4.79	-
chr12	56105815	56106595	781	308	22	55.32	4.79	3679:5607835<br>5-56106089:U<br>pstream
chr13	36336645	36338093	1449	1059	57	63.6	4.79	100302239:36<br>048405-36515<br>382:Genebod<br>y
chr14	49988818	49989852	1035	732	28	58.69	4.79	-
chr6	23740038	23740799	762	190	21	51.91	4.79	-
chr11	9726228	9727735	1508	231	50	75.73	4.78	23075:968562<br>7-9774505:Ge<br>nebody
chr2	1461297	1462633	1337	491	55	78.69	4.78	7173:1417232<br>-1546498:Gen<br>ebody
chr20	16168134	16169717	1584	501	64	86.61	4.78	-
chrY	58874431	58875601	1171	632	46	58.71	4.78	-
chr1	249134746	249135604	859	509	29	59.1	4.76	79894:249132

								529-24914371<br>3:Genebody
chr14	47760733	47761541	809	351	26	50.47	4.76	161357:47308<br>829-47812438<br>:Genebody
chr16	46416153	46418111	1959	813	195	110.84	4.76	-
chr21	30226856	30227466	611	193	17	51.16	4.76	-
chr3	16218602	16219545	944	408	30	55.47	4.76	117248:16216<br>183-16271252<br>:Genebody
chr7	90436	91334	899	407	30	59.7	4.76	-
chr9	93603225	93604601	1377	895	51	67.68	4.76	6850:9356401<br>1-93660841:G<br>enebody
chr15	89226787	89227358	572	296	20	51.78	4.74	-
chr17	41626170	41627263	1094	746	33	58.37	4.74	-
chr3	934657	935463	807	294	25	52.36	4.74	-
chr3	47652865	47653614	750	464	25	57.91	4.74	6599:4762737<br>7-47823405:G<br>enebody
chr4	24874009	24875191	1183	387	34	55.45	4.74	91050:248093<br>91-24914580:<br>Genebody
chr4	36347522	36348554	1033	297	33	68.24	4.74	401124:36283<br>243-36347377<br>:Downstream
chr4	52767016	52767905	890	395	27	53.93	4.74	23142:527092<br>75-52783001:

								Genebody
chr4	65287956	65288929	974	380	28	53.93	4.72	-
chr4	168328397	168329162	766	562	23	53.23	4.72	-
chr5	98328063	98328772	710	179	22	53.6	4.72	-
chr5	169967397	169968298	902	709	26	55.38	4.72	30820:169780880-170163634:Genebody
chr7	2437695	2438692	998	383	29	56.47	4.72	-
chr7	21313804	21314605	802	493	23	50.01	4.72	-
chr12	121432387	121433231	845	319	27	50.3	4.69	6927:121416548-121440312:Genebody
chr13	49896049	49897100	1052	751	29	60.33	4.69	81617:49882786-49975735:Genebody
chr18	54987963	54988598	636	215	19	51.77	4.69	-
chr2	186234379	186235443	1065	573	32	52.01	4.69	-
chr7	38215207	38215805	599	456	22	52.32	4.69	-
chr9	68396328	68397336	1009	195	37	58.36	4.67	-
chr11	60254007	60254668	662	193	21	52.03	4.63	-
chr7	120342390	120342899	510	176	18	52.61	4.63	3751:119913721-120390387:Genebody
chr1	44062354	44063231	878	341	32	68.26	4.62	5792:43996546-44089342:Genebody
chr4_gl000193_random	86141	87185	1045	347	66	86.69	4.61	-

chr21	10084954	10087637	2684	753	132	91.89	4.6	-
chr9	92755557	92756410	854	426	25	60.92	4.59	-
chr1	41889960	41890967	1008	666	36	59.72	4.58	-
chr12	46875377	46876453	1077	365	32	52.57	4.58	-
chr16	87000197	87001086	890	200	27	51.27	4.58	-
chr8	55770755	55771678	924	425	31	51.29	4.58	-
chr1	48004766	48005620	855	430	25	52.01	4.55	-
chr1	217251646	217252778	1133	875	33	60.01	4.55	2104:216676587-217262976:Genebody
chr1	235319759	235320720	962	283	28	56.45	4.55	23029:235294498-235324571:Genebody
chr15	32938922	32939825	904	618	26	52.35	4.55	6447:32933869-32989298:Genebody
chr6	41157406	41158589	1184	819	36	51.44	4.55	79865:41157551-41168925:Genebody
chr14	59554542	59555711	1170	652	35	55.9	4.51	-
chr2	156598354	156599352	999	198	31	71.07	4.5	-
chr2	222546103	222546783	681	261	20	50.12	4.5	-
chr22	23012121	23012798	678	236	20	50.4	4.5	-
chr22	29471541	29472304	764	539	22	52.56	4.5	83999:29469065-29542803:Genebody
chr4	46866770	46867774	1005	190	28	56.07	4.5	170712:46736848-46911252

								:Genebody
chr4	57393866	57394590	725	469	23	61.47	4.5	-
chr4	86149435	86150438	1004	396	28	56.15	4.5	-
chr11	29418662	29420102	1441	442	52	75.19	4.49	-
chr8	43836914	43838176	1263	614	81	63.38	4.49	-
chr1	145215509	145216370	862	472	27	51.21	4.44	388677:145209110-145285911:Genebody
chr16	26823833	26824902	1070	430	34	58.54	4.44	-
chr16	33045990	33047112	1123	189	41	85.04	4.44	-
chr14	20132481	20133960	1480	1113	60	71.17	4.43	-
chr12	51212679	51213285	607	410	19	50.91	4.41	466:51157818-51214906:Genebody
chrUn_gl000234	2632	4214	1583	301	47	58.57	4.41	-
chr15	98544223	98545456	1234	712	46	53.83	4.4	-
chr9	2241295	2242480	1186	468	44	51.47	4.4	-
chr10	6446997	6448012	1016	749	31	57.76	4.39	-
chr12	4276369	4277441	1073	867	31	53.1	4.39	-
chr13	87471660	87472685	1026	725	30	52.61	4.39	-
chr2	226041428	226042093	666	220	22	52.99	4.39	-
chr17	27013374	27014443	1070	596	33	57.07	4.37	6830:26989301-27029248:Genebody
chr3	9714065	9715199	1135	361	34	55.72	4.37	64419:9691116-9744078:Genebody

chr15	92468784	92469819	1036	188	31	64.15	4.33	28232:92396937-92709137:Genebody
chr20	34448198	34449240	1043	314	28	50.07	4.33	51230:34359922-34538288:Genebody
chrUn_gl000225	31856	32893	1038	603	41	76.41	4.33	-
chr1	143476038	143476817	780	453	24	50.25	4.31	-
chr4	48527241	48528441	1201	387	39	75.61	4.31	285527:48499379-48782316:Genebody
chr4	183848637	183849636	1000	613	29	53.29	4.31	-
chr5	179143338	179144582	1245	407	35	54.81	4.31	821:179125929-179158637:Genebody
chr10	135499151	135500882	1732	1068	77	99.57	4.29	-
chr22	50294966	50296648	1683	1049	55	76.31	4.29	79087:50296855-50312106:Downstream
chrUn_gl000241	40475	41603	1129	696	38	58.07	4.29	-
chrUn_gl000232	24043	25337	1295	885	42	50.84	4.28	-
chr1	17051837	17053165	1329	235	45	66.37	4.23	-
chrY	58842332	58843754	1423	559	82	55.41	4.22	-
chr19	20727226	20728523	1298	870	50	60.76	4.21	100129842:20720798-20748

								626:Genebody
chr10	310583	311723	1141	496	39	62.03	4.17	-
chr21	18096491	18097473	983	763	28	51.74	4.17	-
chr7	43237456	43238645	1190	588	41	56.85	4.17	23072:43152197-43602938:Genebody
chr9	13063452	13064289	838	523	28	60.69	4.17	-
chrY	58835928	58838001	2074	1491	118	63.29	4.17	-
chr18	45747044	45748789	1746	365	83	100.34	4.13	-
chr18	77341498	77343109	1612	1246	61	69.91	4.12	-
chr16	82061701	82063173	1473	1039	52	66.91	4.11	-
chrUn_gl000214	63958	66385	2428	992	193	104.25	4.11	-
chr15	75015990	75017307	1318	1092	46	74.25	4.1	1543:75011883-75017877:Genebody
chr17	71691724	71692795	1072	196	32	51.56	4.1	-
chr19	6131982	6133173	1192	286	34	50.29	4.1	-
chr6	84816952	84817924	973	638	30	60.06	4.09	-
chr7	45895307	45896488	1182	879	32	60.43	4.09	-
chr16	74446385	74447415	1031	445	39	61.61	4.06	497190:74442530-74455368:Genebody
chr17	76049923	76051262	1340	384	52	81.71	4.06	57690:76000317-76104916:Genebody
chr8	143610181	143611391	1211	762	39	53.15	4.05	575:14354537

								6-143626367:<br>Genebody
chr10	90179981	90180743	763	196	25	51.57	4.03	55328:900336<br>20-90343082:<br>Genebody
chr13	107937475	107938584	1110	672	33	51.18	4.03	728215:10782<br>0879-1085194<br>60:Genebody
chr17	34066392	34067515	1124	397	33	50.1	4.03	91608:340586<br>78-34070539:<br>Genebody
chr3	192504944	192505764	821	395	25	53.69	4.02	-
chrUn_gl000<br>214	112318	113499	1182	314	43	65.67	4	-
chr4	57438130	57439076	947	613	28	50.44	3.95	-
chr5	1545137	1546520	1384	615	43	52.43	3.93	-
chr15	20481383	20482549	1167	183	35	51.74	3.91	-
chr7	65261412	65262698	1287	380	38	54.02	3.91	-
chr11	34504757	34506026	1270	773	45	62.26	3.9	2001:3450034<br>1-34533346:G<br>enebody
chr4	186625433	186626750	1318	864	38	50.28	3.85	8470:1865065<br>98-186697066<br>:Genebody
chr17	21906119	21907095	977	603	58	56.36	3.81	284124:21904<br>061-21913070<br>:Genebody
chr21	11180653	11182108	1456	796	84	53.55	3.78	-

chr10	624455	626545	2091	1368	71	53.42	3.76	22982:320131-735608:Genebody
chr4	115942101	115943569	1469	857	48	50.74	3.7	64579:115748930-116035032:Genebody
chr7	61967107	61971345	4239	2020	2111	131.6	3.68	-
chr7	103020986	103022596	1611	1184	63	65.54	3.65	375611:102993176-103086624:Genebody
chr1	17564997	17567283	2287	821	143	89.04	3.59	29943:17531620-17572501:Genebody
chr4	154865	155964	1100	883	34	54.55	3.57	255403:53276-156488:Genebody
chr8	58121880	58123056	1177	362	87	82.5	3.49	-
chr7	61890769	61893846	3078	1889	421	162.54	3.39	-
chrY	28815060	28818129	3070	2323	338	147.62	3.38	-
chrUn_gl000225	76356	78045	1690	791	65	52.45	3.33	-
chr7	155750139	155752764	2626	946	135	52.77	3.22	-
chrX	61718379	61720399	2021	563	150	63.29	3.22	-
chr16	46410216	46413735	3520	1953	267	51.86	3.08	-
chrUn_gl000224	2745	5450	2706	1062	586	114.38	2.86	-
chr6	58776135	58780163	4029	3056	3340	294.99	2.13	-

Supplementary Table S11. Probe design for MLPA.

name	sequence	base count
**1p36.33-LPO1**	GGGTTCCCTAAGGGTTGGAGTCCAGGTTCTTGTGCTGAGAGG	42
**1p36.33-RPO1**	CTCCTTGGATCAGTGGGTTTATGGTTCTAGATTGGATCTTGCTGGCAC	48
**1p36.33-LPO2**	GGGTTCCCTAAGGGTTGGAGTTTGGATTTTCCTGCAGGGTTTCATTG	47
**1p36.33-RPO2**	GAACACCAGGTTCCAGGGCCGTAGATCTAGATTGGATCTTGCTGGCAC	48
**1p36.33-LPO3**	GGGTTCCCTAAGGGTTGGACTAAAATTCCCATTTGGCTCATTTAAACATGTTTC	54
**1p36.33-RPO3**	CTCGCTGAGATTTCCGGCACTTTTCTAGATTGGATCTTGCTGGCAC	46
**1q31.3-LPO4**	GGGTTCCCTAAGGGTTGGAGTCACTGTTGAAAGGAAGCTGAGCAGTCC	48
**1q31.3-RPO4**	ATGAGCATTCAACTAACCTTTCTGTCAATGGTACTCTAGATTGGATCTTGCTGGCAC	57
**1q31.3-LPO5**	GGGTTCCCTAAGGGTTGGACACATCAAAACCACATTCAACAATAGTTTCTATTAGC	56
**1q31.3-RPO5**	CATGATCCTGGAATATTTTGTCTCCATCAGTTCTAGATTGGATCTTGCTGGCAC	54
**1q31.3-LPO6**	GGGTTCCCTAAGGGTTGGAGTCTTCACGACATTCTATTTTGTGCAGAGTATCATGG	56
**1q31.3-RPO6**	GAGTTCAATGTGTCAGGAACAATAGGAATTGTAACCTCTAGATTGGATCTTGCTGGCAC	59
**7q36.3-LPO7**	GGGTTCCCTAAGGGTTGGACAAGAAGGATAGATGCTATATAATTTCATCTAGACAACAC	59
**7q36.3-RPO7**	CTGCATAACCAACTCTTGTGTCCTATGGGATAACTAACTCTAGATTGGATCTTGCTGGCAC	61
**7q36.3-LPO8**	GGGTTCCCTAAGGGTTGGACTGGAAAAATGCAAAGCATTTCTATAAAGTAGTTAAGG	57
**7q36.3-RPO8**	CTTTACATGTATATCAAATAGAAATGGAACCTATACATTCAAGACTCTAGATTGGATCTTGCTGGCAC	68
**17p13.1-LPO9**	GGGTTCCCTAAGGGTTGGATCGTCATTGATATCATTGGAGCACTGGCCTTGATTATAGTCCTC	63
**17p13.1-RPO9**	CACTTTCTGCTCCTAAGCACTGCACACACACAGCTGCTAGGCCTTCTAGATTGGATCTTGCTGGCAC	67
**17p13.1-LPO10**	GGGTTCCCTAAGGGTTGGAAAGTTAGGGCTGAAGAGACTAACTTATAAGAGAATTTATACATATC	65
**17p13.1-RPO10**	CTAAGGTAACCACACCTACAGTAGTTTGCTATTGTCATTTCCATACATCTAGATTGGATCTTGCTGGCAC	70
**17p13.1-LPO11**	GGGTTCCCTAAGGGTTGGATAAGGTAGTAGTAGCCACTACAATGATCAAAACAGAAAGAAAGTAAC	66
**17p13.1-RPO11**	GAAATCTACATACGTAATCAAGATTGACTGGTTTTCACGATCTGTAAGGGATCTAGATTGGATCTTGCTGGCAC	74
**4p16.1-LPO12**	GGGTTCCCTAAGGGTTGGAGACCTTAGCACAGTCCACGAAGAGGACAAGGTTACTAGATGTCAAAGTGCCACG	73
**4p16.1-RPO12**	GTGAAAAGGATTAACAAATGTATGTGGTACATGCAGGTAGGGAAACGACTCTAGATTGGATCTTGCTGGCAC	72
**4p16.1-LPO13**	GGGTTCCCTAAGGGTTGGAGACCTGGACCTGACCTGGGAAGAATTTCTGACAATGAACATGCACGTCTTCTCTCC	75
**4p16.1-RPO13**	CAATGACAGTGGTAAAGATGTAATCGCCATCAGCAACCCTTACTGAGCGTACTCTAGATTGGATCTTGCTGGCAC	75

**4p16.1-LPO14**	GGGTTCCCTAAGGGTTGGACGGATCATGGACCTGTCTTGCCTTGTATCCACTTTATATGCCAACGTGTTCGCTTCCAG	78
**4p16.1-RPO14**	CCACAATTCCAGACTTTGAGAGCATGCTGTTAACTGATCCTGACGTAGCTAGGCTCTAGATTGGATCTTGCTGGCAC	77
**5q21.3-LPO15**	GGGTTCCCTAAGGGTTGGACGAATACCTGTTACTGCAGCCCAGAATGAAACTTTAAGCTGTGTTGGTGAACCCAGACCTC	80
**5q21.3-RPO15**	CTCTACTGTGCATTGTAATCAATTAAATCCTGGCAACAATTAGGAAGGCTTGATACATCTAGATTGGATCTTGCTGGCAC	80
**5q21.3-LPO16**	GGGTTCCCTAAGGGTTGGAGGTAAAGCCCATACATGGAAGGAAGCATTTACTGCACAGTCAAGTGTATGGTAAGCAATATGAC	83
**5q21.3-RPO16**	CTCATAGAAAGAAGAACTAAGAACCCAAAACCCTGGTTTGAAGCCTATCTTAATACATGTCTAGATTGGATCTTGCTGGCAC	82
**5q21.3-LPO17**	GGGTTCCCTAAGGGTTGGAATCTAATGGGAAGCAATATCATATATAAAACATCTAGGAACCCTTGTTAAGTTGGTCAGTCAGTTC	85
**5q21.3-RPO17**	CCAAATTGCCACAAGACATCAAACAGCAAAGTTAGCTAAACTCGGGAAAATAACGCCTGTGTTCTAGATTGGATCTTGCTGGCAC	85
**5q34-LPO18**	GGGTTCCCTAAGGGTTGGACCATTCTGCAAATCTCTTGACACATTATAAGGACCCATGAATACACAGTCTCTGGAATGGAAGAAAGAG	88
**5q34-RO18**	GTGATCTGCATTCAGGATGTGATTTAATTGCGCCATATAAGACCTCCACTATCTACCCTTGGAATCTAGATTGGATCTTGCTGGCAC	87
**5q34-LPO19**	GGGTTCCCTAAGGGTTGGACCTACTTCGTTCAACAAGGAGAGCAACAAGGCCACTAGAGAGATGACTACAGAAATCCTGTTGAGAAGATG	90
**5q34-RO19**	GTAACTGTGACTAGAGGACTATGACCAGTCTAAACACACTCAGAGCGAAGTGAGTTGCAGTGGTCAGTCTAGATTGGATCTTGCTGGCAC	90
**5q34-LPO20**	GGGTTCCCTAAGGGTTGGAATTACAACGATGTTTCATATCATTAGGCTCAAACCAAGTAAATCCCAGAATCCTAAGCTGATGACACATCCTG	92
**5q34-RPO20**	GGTTTGCACTAATCATAATTTGCAATCTCCATCATTGTGCTGAGAAGGTTCCTGGGTAGCGGAATCAATCTCTAGATTGGATCTTGCTGGCAC	93
**8q24.23-LPO21**	GGGTTCCCTAAGGGTTGGACATTCAGATTAACTATGACTTTGACCAAAACTGCTTCTGTGGAATGGTAAG	70

**8q24.23-MPO21**	GGCAAATGCCTGAGTGCTTGACTGGCTTGGGTTTAAGGGAAATAGAAGTAAC	52
**8q24.23-RPO21**	GAGGGAAAAGGAATATAAACTACTCTGTGAGGAACTTTGTGGTGCTCTAGATTGGATCTTGCTGGCAC	68
**8q24.23-LPO22**	GGGTTCCCTAAGGGTTGGAGTAATAGCAGGAAGCTGTTAGCACCCCTGGACCTGAAGGTCAAAGAGGTGGAGCTG TTG	78
**8q24.23-MPO22**	CAGAAATAGGACCAATAGCCAACTGGAGGAACTGAGGACTTCAGGTGG	48
**8q24.23-RPO22**	GCCAAACTGTGATCCGATCCAGTTGGACAAAGATGGTGCATTTCTGTCTAGATTGGATCTTGCTGGCAC	69
**8q24.23-LPO23**	GGGTTCCCTAAGGGTTGGAGTCAACAGGATAGGAACTCTTACTTCTTGACTCTATAACTTGAGCTCTTAAC	71
**8q24.23-MPO23**	CTTTGTTCTCAACATTGTGTGCCCATGAGTTTAGAGAGAAAGGCAAGCGTGG	52
**8q24.23-RPO23**	CTTATAGAAGCTATGAAATCAATAAGCCGTGAGTGTCAACTTTGATAACAAATGTCTAGATTGGATCTTGCTGGCAC	77
**2q32.1-LPO24**	GGGTTCCCTAAGGGTTGGAGGACTTGGACTTAAGCTAATTTTCCCTTCATGTTTATAGACCTTAGCTAGGTCTCTTG G	78
**2q32.1-MO24**	GGCAATAAGGGTAGAATCATGCCCAACATGTATGTTTAGTTTTACAG	47
**2q32.1-RPO24**	CCATTTGTTTGTGAATTCATGCAATGGTGTGCTGGAACTGGCTTACACGACGTAACGTCTAGATTGGATCTTGCTGG CAC	80
**2q32.1-LPO25**	GGGTTCCCTAAGGGTTGGAGGACTTGGACTGTCCAGCAGTATGAACTGGGAATTTAAAAATCACAGCTTAAGTACA GAAG	80
**2q32.1-MPO25**	GAGGAATGATGTCACCCAGCTTTAAATTTAGGAGAAAATAATTTAGTAGC	50
**2q32.1-RPO25**	CTTTAGTTTTAATTTAGGTTATTGCCATTTATTACGATGATACATTATAACTCAACGTCTAGATTGGATCTTGCTGGCA C	80
**16q24.2-LPO26**	GGGTTCCCTAAGGGTTGGAGGACTTGGACTGGACTTGGACTGGACTTTGGCACGTGAGCCTCACCTGATCTTGACC ATCTCAG	83
**16q24.2-MPO26**	CTAAAACACTGAACCACCCTTGGACTCTTACTACACCCACCAGCAGCAGACGG	53
**16q24.2-RPO26**	AGAAGAACTGTATAGGGTGCAAAAGCGCCAACTCAACGAACTCAACGAACTCAACGTCTAGATTGGATCTTGCTGG CAC	79
**16q24.2-LPO27**	GGGTTCCCTAAGGGTTGGAGGACTTGGACTGGACTTGGACTGGACTTTGGAGGAGGCACGAGAGGCCCTCAGCTA CGACCCAGTGCC	87
**16q24.2-MPO27**	CGGTGCTGCGGCTGGAGGAGGTGTTGGAGGCAGACACCCACCAGC	45
**16q24.2-RPO27**	GCTCCATCTCACCCTGGAGATACCGGTGAGGACCTAGAAACTCAACGAACTCAACGAACTCAACGTCTAGATTGGA	88

	TCTTGCTGGCAC	
**20p12.1-LPO28**	GGGTTCCCTAAGGGTTGGAGGACTTGGACTGGAGTGACTGGCTGTAGCAAGAGATGACAGCTGACCTTTGGATACTAAACTAG	83
**20p12.1-MPO28**	CCATAAAACTGAAAAATCATGCCATGATACCTTGTTGAATCACTTCTTAAGTAAAGGTTAAC	62
**20p12.1-RPO28**	TTACATACATTACATTCTGATTGTTTAAACAGAAACAAGTGTAACTCAACGAACTCATCTAGATTGGATCTTGCTGGCAC	80
**20p12.1-LPO29**	GGGTTCCCTAAGGGTTGGAGGACTTGGACTCTCGATTTTATACTCTCCCTTGCTACCATAAGCAATTCATTATCACAGTCTGTC	84
**20p12.1-MPO29**	CGTGTTACTCTCGCATCTCCTTCTCTGTATTCATTGACTTGACCCCTGCTGAGCCCAAACTACCATC	67
**20p12.1-RPO29**	ACTATTCACCTAGACTGTTGCGATATCCTTCTAAATGGTCTCTACTCAACGAACTCATCTAGATTGGATCTTGCTGGCAC	80
**20p12.1-LPO30**	GGGTTCCCTAAGGGTTGGAGACTTGGACTAATCGTGACCTGCTCTGGTTTATCATTTAAGGGATCCCCTTCACATCCCAAATC	83
**20p12.1-MPO30**	CTCCCACATTAGCCCCTTCTCCCATCATTTAGCTTTTTACCTGTGCTATAACTTGGTTCTATCCCTTAC	69
**20p12.1-RPO30**	TAGTATCTCATTTCTGGGAGGAGAGGACTCTGTCCAACTTGTTAGTGTTCTCAACGAACTCATCTAGATTGGATCTTGCTGGCAC	85

Supplementary Table S12. The sequence of specific primers for *L3MBTL4*, *LTBP1* and *GAPDH* in quantitative real-time PCR.

L3mbtl4 (species: rat)
forward: 5'-AGGCAGTGGACAGGAGGAAC-3'<br>reverse: 5'-CATCGCACCAGTAATCAAAGC-3'<br>product size: 120bp
Ltbp1 (species: rat)
forward: 5'- GGCCAGAGGGTGAAAGAAGT -3'<br>reverse: 5'- GGTGGGAGTATGTGGAATGG -3'<br>product size: 100bp
Gapdh (species: rat)
forward: 5'-CCGTATTCAGCATTCTATGCTCT-3'<br>reverse: 5'-TGTTTTTGTAAGTATCTTGGTGCCT-3'<br>product size: 115bp
L3MBTL4 (species: human)
forward: 5'-CATTCCGATTTGTTGTCAGG-3'<br>reverse: 5'-ATGGTGTCCCTCCACTTCTG-3'<br>product size: 177bp
GAPDH (species: human)

forward: 5'-CTCTGCTCCTCCTGTTCGAC-3'
reverse: 5'-GCGCCCAATACGACCAAATC-3'
product size: 121bp

Supplementary Table S13. The gene specific primers for genotype identification of *L3MBTL4* transgenic rats.

L3MBTL4
forward: 5'-GTGCCACAGCGAACGAATG-3'<br>reverse: 5'-TGGGATGCAGCACAAGC-3'<br>product size: 376bp
GAPDH
forward: 5'-AGCAACAGGGTGGTGGA-3'<br>reverse: 5'-CAGCGAACTTTATTGATGGTAT-3'<br>product size: 240bp

Supplementary Table S14. The primer of q-PCR to determine the enrichment of *LTBP1* peak regions in immunoprecipitated DNA fragments.

***LTBP1* (species: human)**
forward: 5'- GCAGTGACTCACGCCTGTAA -3'<br>reverse: 5'- CCATCATGCCTGGCTAATTT -3'<br>product size: 138bp

Supplementary Figures and Supplementary Figure Legends

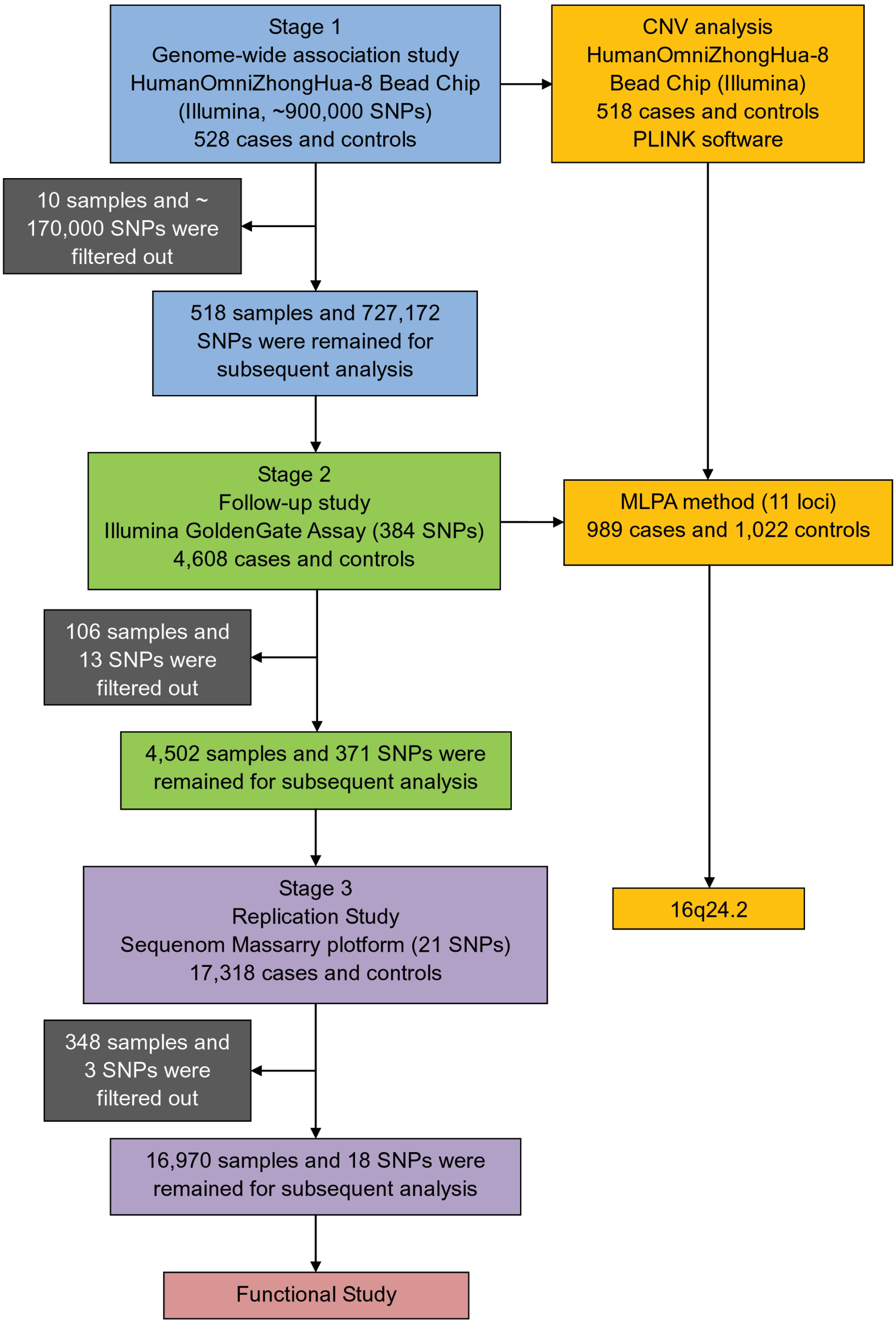

Supplementary Figure S1. A flow chart of our study. SNPs, single nucleotide polymorphisms. CNV, copy number variation. MLPA, multiplex ligation-dependent probe amplification.

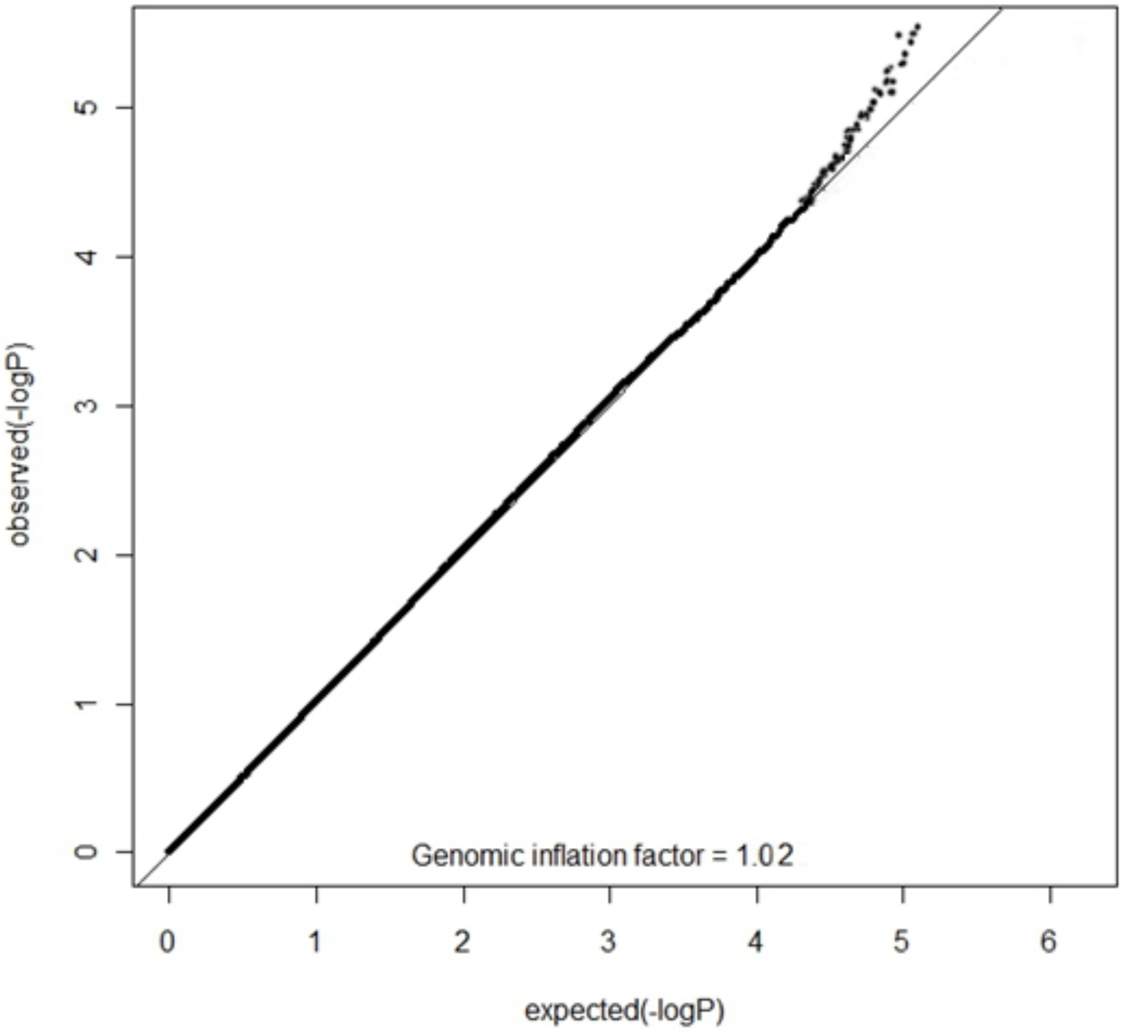

Supplementary Figure S2. QQ plot of genome-wide association study. The QQ plot is a graphical representation of the deviation of the observed P values from the null hypothesis: the observed P values for each SNP are sorted from largest to smallest and plotted against expected values from a theoretical χ2-distribution. If the observed values correspond to the expected values, all points are on or near the middle line between the x-axis and the y-axis (null hypothesis: light gray line in Fig). If some observed P values are clearly more significant than expected under the null hypothesis, points will move towards the y-axis.

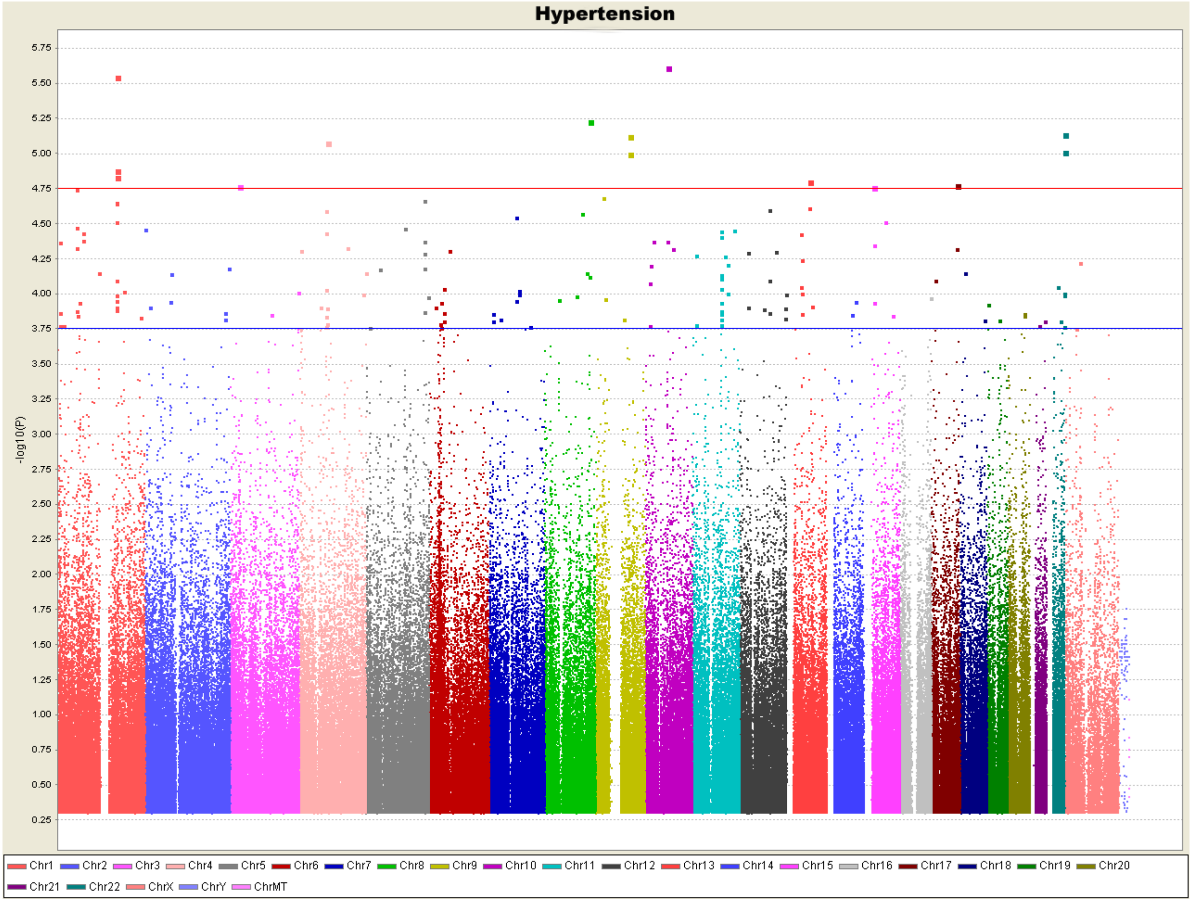
Hypertension
-log10(P)
5.75
5.50
5.25
5.00
4.75
4.50
4.25
4.00
3.75
3.50
3.25
3.00
2.75
2.50
2.25
2.00
1.75
1.50
1.25
1.00
0.75
0.50
0.25
Chr1 Chr2 Chr3 Chr4 Chr5 Chr6 Chr7 Chr8 Chr9 Chr10 Chr11 Chr12 Chr13 Chr14 Chr15 Chr16 Chr17 Chr18 Chr19 Chr20
Chr21 Chr22 ChrX ChrY ChrMT

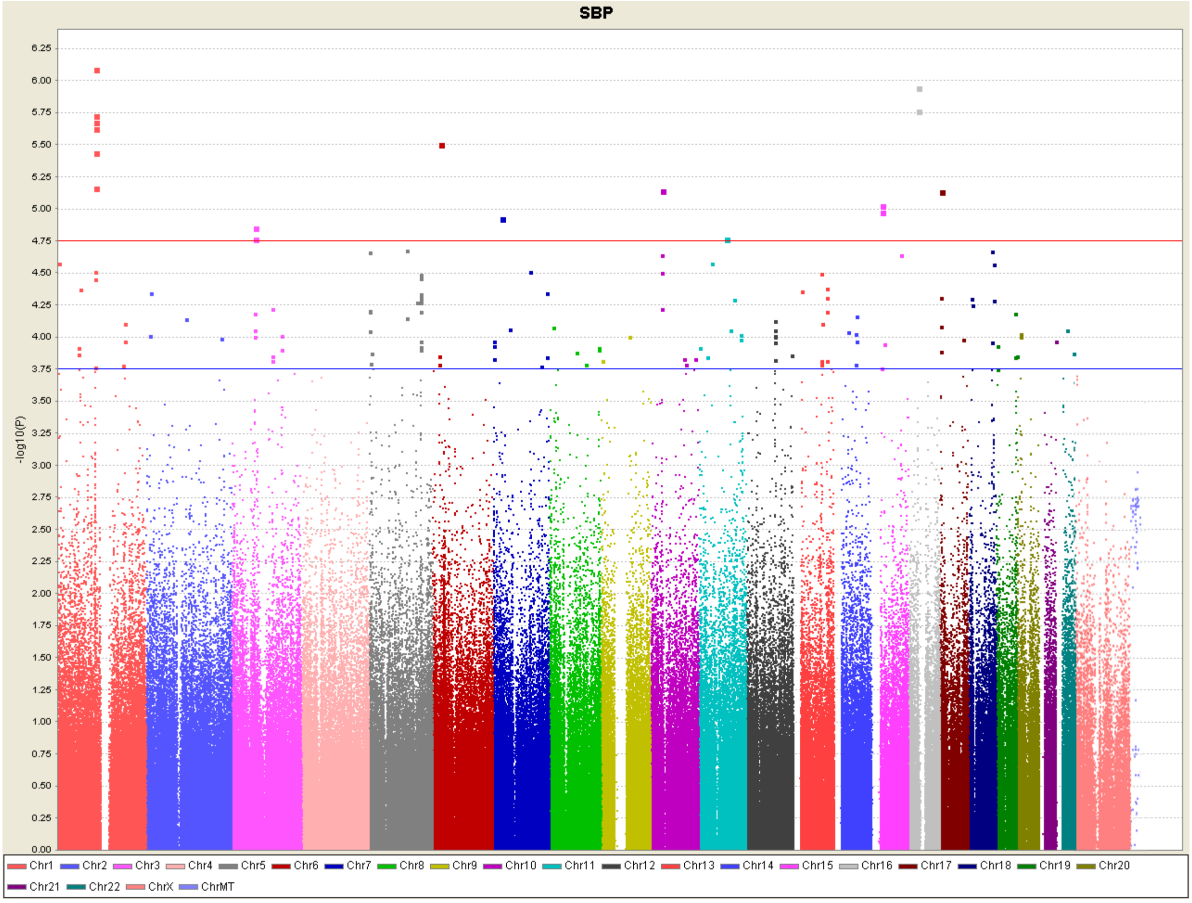
SBP
-log10(P)
6.25
6.00
5.75
5.50
5.25
5.00
4.75
4.50
4.25
4.00
3.75
3.50
3.25
3.00
2.75
2.50
2.25
2.00
1.75
1.50
1.25
1.00
0.75
0.50
0.25
0.00
Chr1 Chr2 Chr3 Chr4 Chr5 Chr6 Chr7 Chr8 Chr9 Chr10 Chr11 Chr12 Chr13 Chr14 Chr15 Chr16 Chr17 Chr18 Chr19 Chr20
Chr21 Chr22 ChrX ChrMT

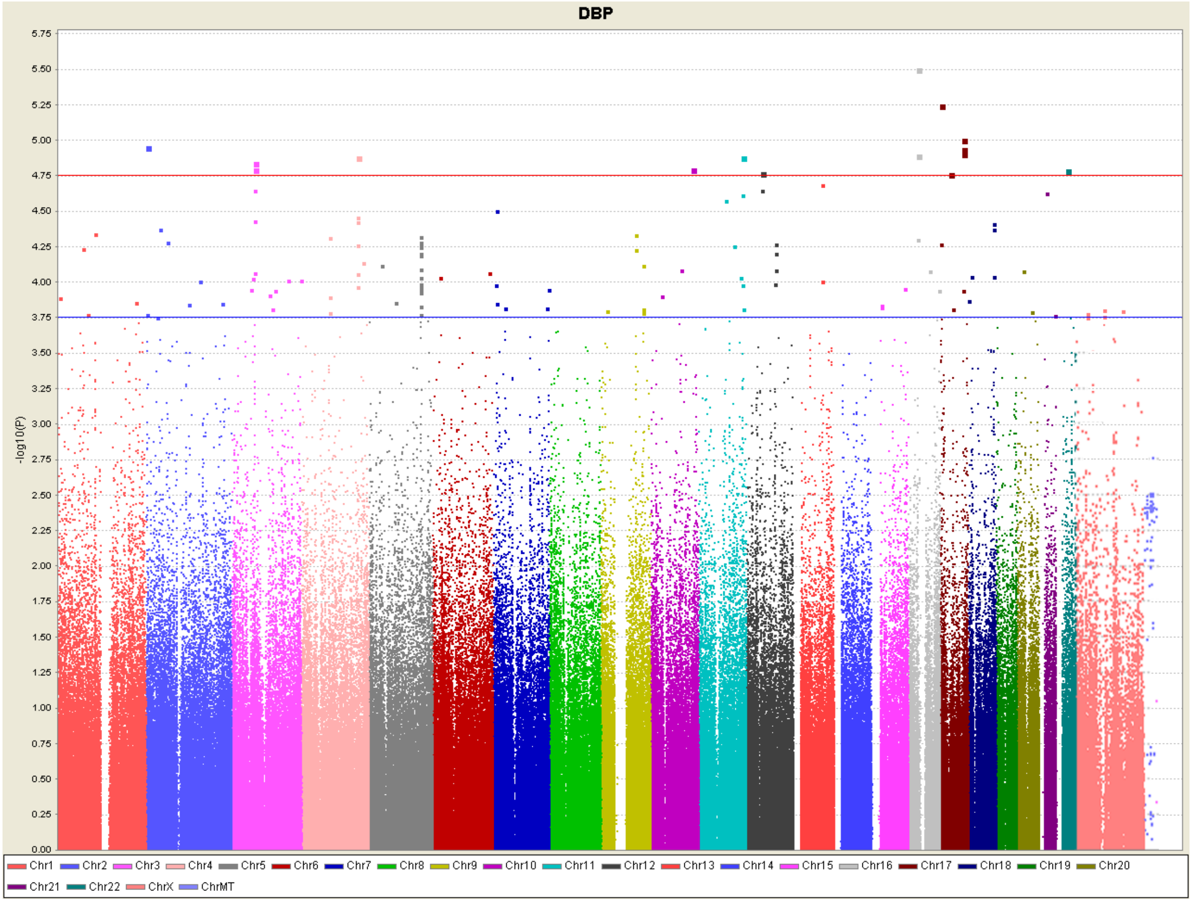

Supplementary Figure S3. Manhattan plot of genome-wide association study of stage 1 (the GWAS scan). For each tested marker, the significance id displayed on the y-axis as the -log_{10} of the P value. The -log_{10} results are ordered along the x-axis by chromosome. Each colored bar represents a different chromosome. Supplemental Figure 3a, 3b, and 3c represent Manhattan plot for hypertension, SBP, and DBP respectively.

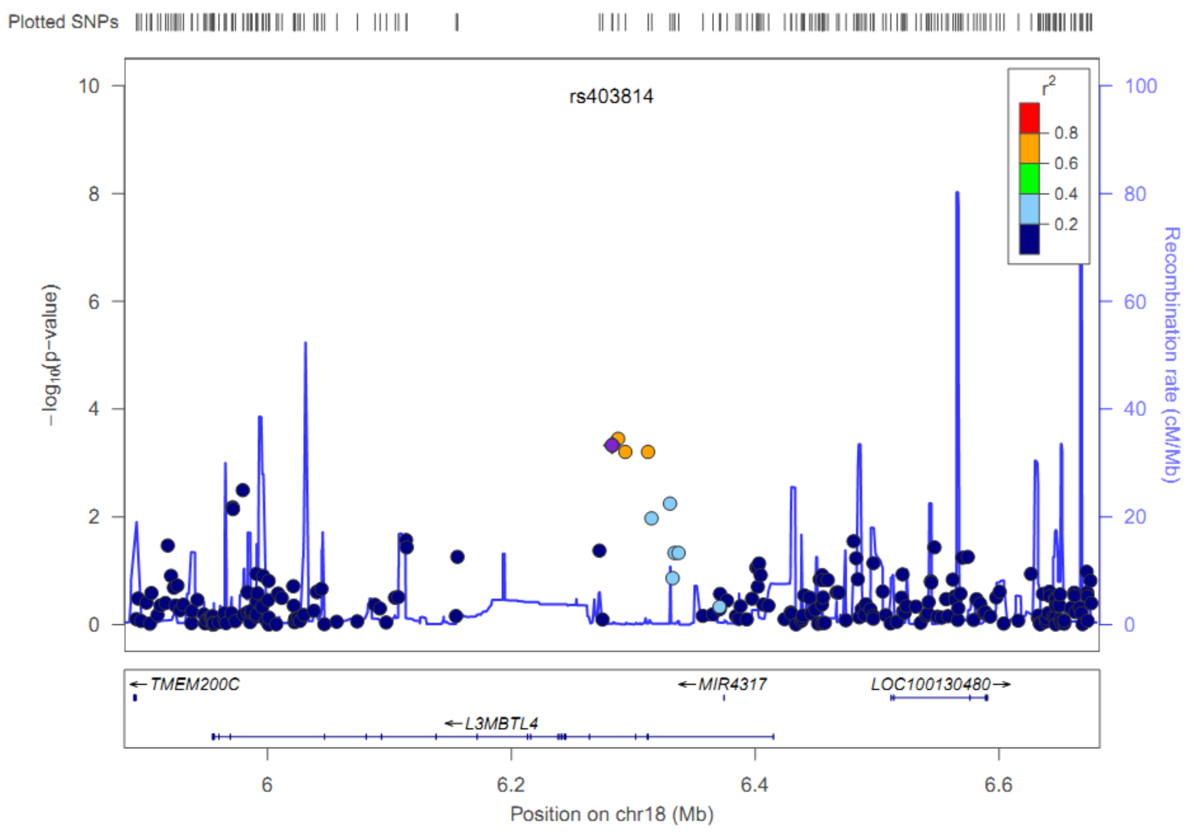

Supplementary Figure S4. Regional association plot of SNP rs403814. A regional association plot focusing on the 18p11.31 region of significant association signals from stage 1 is shown. Blue lines on bottom represent genes and the light blue line shows the recombination rate (secondary y axis) based on hg19/1000 Genomes ASN data. SNP rs403814 (purple diamond) shows association with hypertension (p=0.0005).

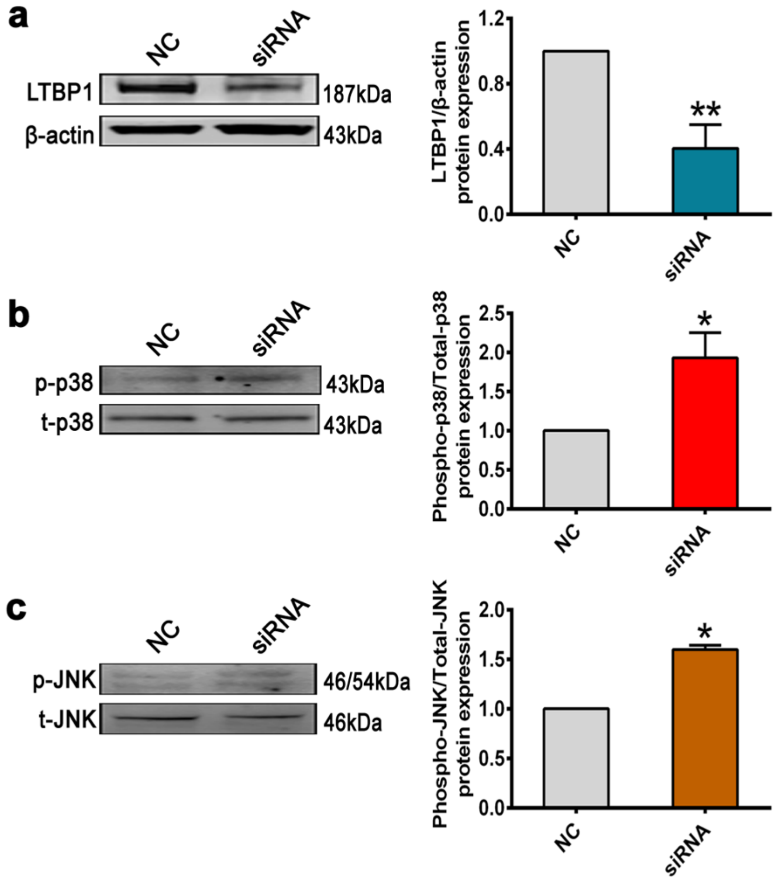

Supplementary Figure S5. The phosphorylation of p38MAPK and JNK is elevated by siRNA targeting LTBP1. (a) Western Blot analysis validate suppressed expression of LTBP1 by siRNA. n=4. **(b-c)** Transfection with siRNA specific to LTBP1 impacted the phosphorylation levels of p38MAPK and JNK. n=4 for **(b)** and n=3 for **(c)**. *p<0.05, **p<0.01 compared to NC. All data represent mean±s.e.m.

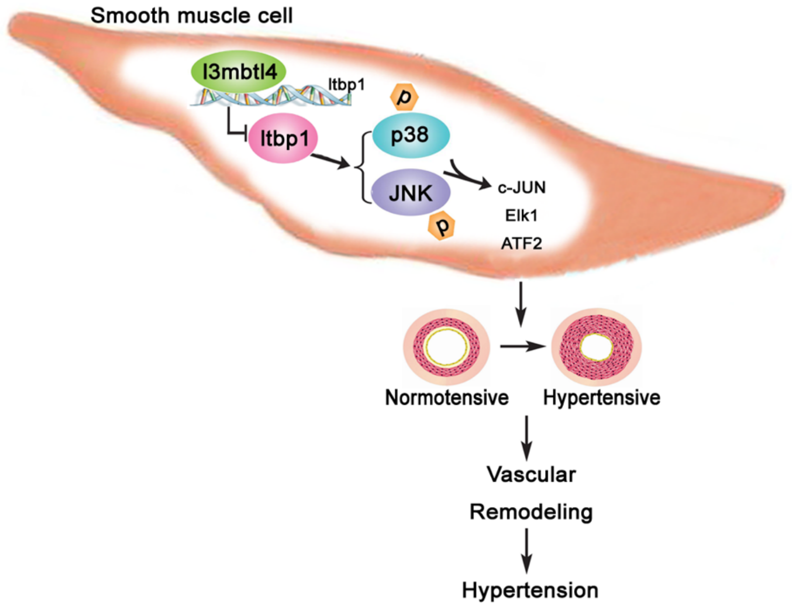

Supplementary Figure S6. Proposed mechanism underlying L3MBTL4-induced vascular remodeling and hypertension.

Supplementary References

1 Liu, L. S. [2010 Chinese guidelines for the management of hypertension]. *Zhonghua xin xue guan bing za zhi* **39**, 579-615 (2011).

2 Willer, C. J., Li, Y. & Abecasis, G. R. METAL: fast and efficient meta-analysis of genomewide association scans. *Bioinformatics (Oxford, England)* **26**, 2190-2191, doi:10.1093/bioinformatics/btq340 (2010).

3 Pruim, R. J. *et al.* LocusZoom: regional visualization of genome-wide association scan results. *Bioinformatics (Oxford, England)* **26**, 2336-2337, doi:10.1093/bioinformatics/btq419 (2010).

4 Purcell, S. *et al.* PLINK: a tool set for whole-genome association and population-based linkage analyses. *American journal of human genetics* **81**, 559-575, doi:10.1086/519795 (2007).
